# Functional Improvement after Photothrombotic Stroke in Rats Is Associated with Different Patterns of Dendritic Plasticity after G-CSF Treatment and G-CSF Treatment Combined with Concomitant or Sequential Constraint-Induced Movement Therapy

**DOI:** 10.1371/journal.pone.0146679

**Published:** 2016-01-11

**Authors:** Katrin Frauenknecht, Kai Diederich, Petra Leukel, Henrike Bauer, Wolf-Rüdiger Schäbitz, Clemens J. Sommer, Jens Minnerup

**Affiliations:** 1 Institute of Neuropathology, University Medical Center of the Johannes Gutenberg University, Mainz, Germany; 2 Department of Neurology, University of Münster, Münster, Germany; 3 Neurology, Bethel, EVKB, Bielefeld, Germany; School of Pharmacy, Texas Tech University HSC, UNITED STATES

## Abstract

We have previously shown that granulocyte-colony stimulating factor (G-CSF) treatment alone, or in combination with constraint movement therapy (CIMT) either sequentially or concomitantly, results in significantly improved sensorimotor recovery after photothrombotic stroke in rats in comparison to untreated control animals. CIMT alone did not result in any significant differences compared to the control group (Diederich et al., Stroke, 2012;43:185–192). Using a subset of rat brains from this former experiment the present study was designed to evaluate whether dendritic plasticity would parallel improved functional outcomes. Five treatment groups were analyzed (n = 6 each) (i) ischemic control (saline); (ii) CIMT (CIMT between post-stroke days 2 and 11); (iii) G-CSF (10 μg/kg G-CSF daily between post-stroke days 2 and 11); (iv) combined concurrent group (CIMT plus G-CSF) and (v) combined sequential group (CIMT between post-stroke days 2 and 11; 10 μg/kg G-CSF daily between post-stroke days 12 and 21, respectively). After impregnation of rat brains with a modified Golgi-Cox protocol layer V pyramidal neurons in the peri-infarct cortex as well as the corresponding contralateral cortex were analyzed. Surprisingly, animals with a similar degree of behavioral recovery exhibited quite different patterns of dendritic plasticity in both peri-lesional and contralesional areas. The cause for these patterns is not easily to explain but puts the simple assumption that increased dendritic complexity after stroke necessarily results in increased functional outcome into perspective.

## Introduction

Stroke is the leading cause of long-term disability in adulthood [1]. Apart from thrombolysis or mechanical removal of the thromboembolic material occluding the vessel lumen no specific therapy is available yet [2, 3]. The effectivity of neuroprotective strategies for the treatment of ischemic stroke strongly depends on a prompt initiation of treatment which is one of the many problems preventing successful translation into the clinic up to now. In contrast, neurorestorative therapies have the attractive advantage of an extended time window to beneficially modulate brain regeneration after stroke. In rodents, positive effects have been described even when treatment was started with a delay of one month [4]. To further enhance the benefit of restorative therapies a combination of divergent therapeutic approaches to potentiate the success rate seems a promising strategy. However, there exist few studies which systematically addressed this issue.

In the last years the hematopoietic growth factor granulocyte-colony stimulating factor (G-CSF) has been shown to exert both neuroprotective and neuroregenerative effects in experimental models of ischemic stroke [5, 6]. Several mechanisms responsible for the neurorestorative post-ischemic effect of G-CSF are under discussion, including enhancement of neurogenesis and mobilization of bone marrow stem cells [7, 8]. Different training strategies are an alternative approach to improve recovery after stroke [9]. One concept is the so called constrained-induced movement therapy, where the unaffected arm is immobilized thus forcing the use of the affected arm resulting in improved motor function of the paretic arm weeks after unilateral stroke [10, 11].

We have previously shown that the combined treatment with the hematopoietic growth factor G-CSF and constraint-induced movement therapy did not further enhance neurological outcome compared to treatment with G-CSF alone [6]. In this study photothrombotic ischemia was induced in the right frontal cortex of rats resulting in impaired function of the contralateral forelimb as determined by the cylinder test and the adhesive tape removal test. Both motor and somatosensory recovery were significantly improved at 28 days after stroke in the G-CSF treated group and in the two groups with a combination of concomitant or sequential treatment with G-CSF and CIMT while CIMT only just narrowly failed the significance level [6]. Since both therapies are thought to enhance the spontaneously occurring post-stroke plasticity our present study was designed to evaluate whether dendritic plasticity would parallel the functional outcome. The Golgi-Cox method has been described as a powerful tool in a variety of animal models including cerebral ischemia to compare behavior and structural neuronal changes [12–19]. Using this method we could recently demonstrate that G-CSF deficiency in mice causes a reduction of the dendritic length and branching of hippocampal CA1 neurons which was associated with impaired learning and memory function [20]. However, the influence of G-CSF or CIMT on dendritic plasticity in the peri-ischemic or contralateral homotopic cortex has not been analyzed so far. To our knowledge only one study exists demonstrating increased mushroom-type spines on the apical dendrites of layer V pyramidal neurons adjacent to the infarct in a mouse model of chronic stroke and a combination therapy of G-CSF with stem cell factor [21]. Therefore, rat brains of our former experiment [6] were subjected to a modified Golgi-Cox method to visualize dendritic morphology of perilesional layer V pyramidal neurons in the various treatment groups. Since plastic changes are known to occur not only in the peri-infarct cortex but also in homologous areas of the contralateral side as shown for example by an increase of high-molecular weight neurofilament (NFH) positive axons in these regions [18] additionally we chose this area for Golgi Cox analysis.

## Material and Methods

### Experimental Groups

As recently described [6], all animal procedures were carried out according to the guidelines of the German animal protection law and approved by the responsible ethics committee of the University of Münster and the North Rhine-Westphalia State Environment Agency. All efforts were made to minimize the number of used animals and their suffering. Experiments were performed on adult male Wistar rats (Charles River, Sulzfeld, Germany; 280−320g body weight) which had free access to food and water throughout the experiments. Animals were randomly assigned to the following experimental groups (n = 20 each): (i) control group (ischemia, treatment with 0.5 ml saline 0.9% starting 48 hours after ischemia until day 11 after ischemia); (ii) (CIMT) group (ischemia, CIMT starting 48 hours after ischemia until day 11 after ischemia); (iii) G-CSF group (ischemia, treatment with 10μg/kg G-CSF as daily subcutaneous injection starting 48 hours after ischemia until day 11 after ischemia); (iv) combined concurrent treatment group (ischemia, treatment with 10μg/kg G-CSF as daily subcutaneous injection and CIMT starting 48 hours after ischemia until day 11 after ischemia); (v) combined sequential treatment group (ischemia, CIMT starting 48 hours after ischemia until day 11 after ischemia, subsequent treatment with 10μg/kg G-CSF as daily subcutaneous injection starting on day 12 until day 21after ischemia). Randomization was carried out by the computer software “Research Randomizer” (Urbaniak, G. C., & Plous, S. (2011), Research Randomizer (Version 3.0) Retrieved on April 22, 2011, from http://www.randomizer.org/). To ensure blinding, behavioral assessment and treatment administration were performed by different investigators. A total of 107 animals has been used in this study in which 7 animals died during surgeries. A subset of brains has been used for the recently published receptor autoradiographic analyses and brain volume quantifications [6]. Infarct volumes and the remaining cortical tissue did not significantly differ between the various experimental groups [6]. At the determined endpoint of the experiments, animals were deeply anesthetized using an overdose mixture of ketamine hydrochloride (20.38 mg/ml) and xylazine hydrochloride (5.38 mg/ml).

### Photothrombotic Ischemia

Photothrombotic ischemia was induced as described in the previously published study [6]. Briefly, animals were anesthetized with an intraperitoneal injection of ketamine hydrochloride (100mg/kg body weight; Ketanest) and xylazine hydrochloride (8mg/kg body weight; Ceva GmbH), and anesthesia was maintained if necessary. The left femoral vein was cannulated with PE-50 tube for Rose Bengal infusion. During the experiment, rectal temperature was maintained at 37°C by a thermostat-controlled heating pad (Föhr Medical Instruments). Animals were placed in a stereotactic frame, and the scalp was incised for exposure of the skull surface. For illumination, a laser spot of 8 mm in diameter (G Laser Technologies) was stereotactically placed onto the skull of the right hemisphere 0.5 mm anterior to the bregma and 4 mm lateral from the midline and then the skull was laser-illuminated for 20 minutes. During the first four minutes of illumination, the dye Rose Bengal (0.3 mL/kg body weight, 10 mg/mL saline) was injected intravenously. After surgery, the catheter was removed and the animals were allowed to recover from anesthesia.

### Constraint-Induced Movement Therapy

Forty-eight hours after induction of ischemia, CIMT-treated animals were fitted with one-sleeve plaster casts, as recently described [6]. Animals were anesthetized with an intraperitoneal injection of ketamine hydrochloride (100 mg/kg body weight; Ketanest) and xylazine hydrochloride (8 mg/kg body weight; Ceva GmbH). The upper torso and the ipsilateral right forelimb were wrapped in soft felt and, then, the forelimb was positioned in a naturally retracted position against the animal’s sternum. A single plaster of Paris strip was wrapped around the immobilized limb and upper torso.

### Functional Testing

In all animals, cylinder test and the adhesive tape removal test were performed before ischemia (baseline) as well as on days 1, 12, 21, and 28 after ischemia by an investigator blinded to the experimental groups.

### Brain Tissue Processing–Golgi-Cox Method

**Fig 1:**
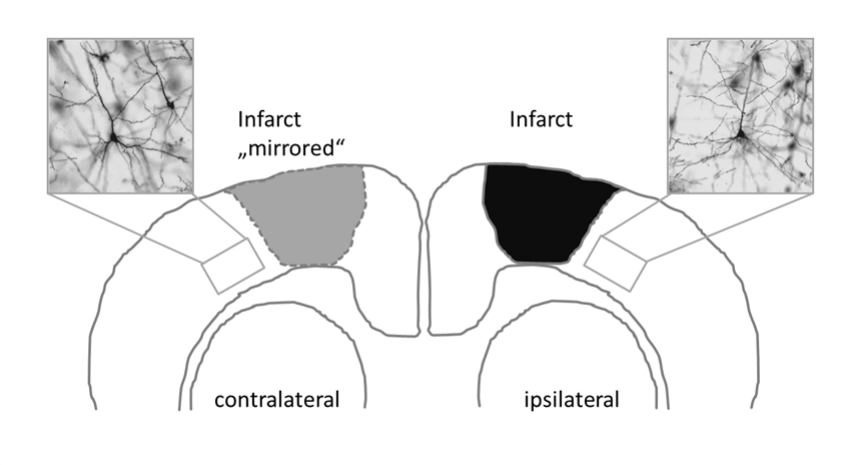
Topographic localization of the photothrombotic infarct. Note that infarct was mirrored on the contralateral side to analyze Golgi Cox-impregnated cortical layer V pyramidal neurons in the ipsilateral peri-infarct cortex and in the corresponding contralateral cortex (Insets: representative Golgi Cox-impregnated cells).

For Golgi-Cox analysis, 6 rats per group were used. At the determined endpoint of the experiment rats were deeply anesthetized using a mixture of ketamine hydrochloride (20.38 mg/ml) and xylazine hydrochloride (5.38 mg/ml). Subsequently, mice were transcardially perfused with 0.9% saline followed by PBS. Brains were removed and were then coded with numbers before sectioning to ensure that the experimenter was blinded to treatment. The brains were processed according to current protocols with minor modifications [22, 23]. In brief, the brains were first stored in the dark for 14 days in 20 ml Golgi-Cox solution. The Golgi-Cox solution was replaced with 30% sucrose and brains were then stored at 4°C for at least 2 days until sectioning. Coronal sections of 200 μm thickness were serially cut with a vibratome (VT1000S, LEICA, Germany). Sections were then collected in serial order on 2% gelatinized slides and were rinsed with distilled water and were then placed into ammonium hydroxide in the dark for 30 min. After additional rinsing with distilled water, sections were immersed for 30 min with Kodak Fix for Film diluted 1:1 with distilled water and subsequently washed with distilled water followed by dehydration in ascending alcohol series (1 min: 50%; 1 min: 70%, 1 min: 95%, 5 min twice: 100%), followed by 10 min in a solution of 33% ethanol, 33% HemoDe (limonene) and 33% chloroform, followed by 15 min twice in HemoDe. The slides were covered with cover slips using Permount. For evaluation of neuron morphology, a LEICA DM6000B microscope and a computer-based system (Neurolucida; MicroBrightField) was used to generate three-dimensional neuron tracings. Visualization and Analysis of neuron morphology were performed using NeuroExplorer (MicroBrightField). Slices were checked for the photothrombotic lesion. Layer V pyramidal neurons of ipsilateral adjacent peri-infarct area (control n = 31; CIMT n = 32, G-CSF n = 30, CIMT+G-CSF n = 27, CIMT/G-CSF n = 26) as well as of the mirrored contralateral cortex (control n = 49; CIMT n = 49, G-CSF n = 50, CIMT+G-CSF n = 42, CIMT/G-CSF n = 35) (Fig 1) with dark and well filled impregnation were randomly selected when they showed a prominent, single apical tree extending from the apex of the soma toward the pial surface of the cortex, at least two basilar dendritic trees extending from the base of the soma. The neurons were chosen for further tracing when located within the middle of the thickness of the section, when distinct from other neurons and when soma and branches where unobscured by other neuron branches or vessels. Analysis was performed blinded to group identity, using a camera lucida and a 40x objective. Dendritic parameters (e.g., apical and basilar dendritic length, mean number of apical and basilar dendrites, branch order, mean number of branch order dendrites, dendrite length per branch order) were calculated for each neuron and for each animal. 3D Sholl analysis was used to obtain dendritic complexity. As previously described [24] concentric spheres of increasing radius (10 μm increments) were placed around the center of the cell body until dendrites were completely captured. The number of dendritic intersections was counted and expressed as total number of intersections as well as the number of intersections per concentric sphere. The results were analyzed for apical and basilar dendrites, separately and were presented as means ± SEM.

### Statistical Analysis

Values are presented as mean ± SEM. The normal distribution of the data was verified by Shapiro-Wilk test. Due to the normal distribution all experimental groups were compared pair-wise and significant group effects were confirmed by one-way analysis of variance (ANOVA) and Bonferroni error protection to correct for multiple comparisons. Significant group effects between different groups were confirmed by independent t-test. A p-value <0.05 was considered statistically significant. Analysis was performed using the general statistics module of Analyse-it™ forMicrosoft Excel (Analyse-it Software, Ltd., Leeds, UK).

## Results

### Functional Testing

As recently published, both motor and somatosensory recovery was significantly increased at 28 days after stroke in the G-CSF treated group and in the two groups with a combination of concomitant or sequential treatment with G-CSF and CIMT compared to untreated ischemic controls. CIMT only did not result in any significant differences compared to the control group [6].

### Golgi Cox Method

#### Dendritic number and length

**Fig 2:**
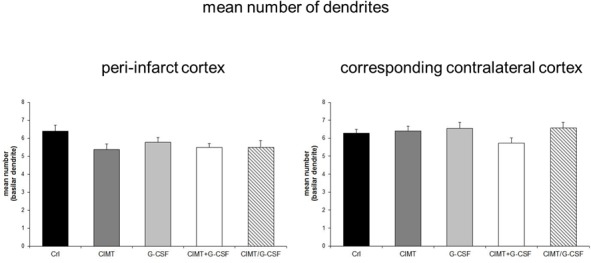
Quantitative analysis of Golgi Cox-impregnated layer V pyramidal neurons in the peri-infarct cortex and in the corresponding contralateral cortex. There were no differences in the number of basilar dendrites between the various experimental groups neither in the peri-infarct cortex nor in the corresponding contralateral cortex (Values are expressed as mean number of basilar dendrites ± SEM).

**Fig 3:**
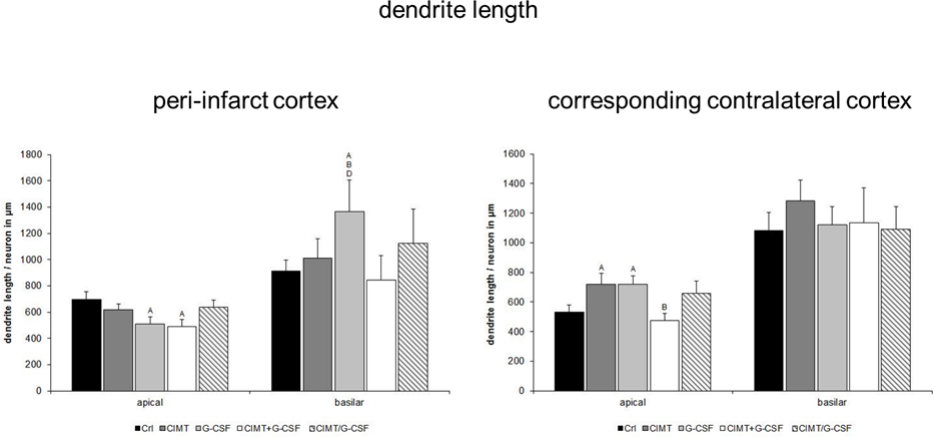
Dendritic length of Golgi Cox-impregnated layer V pyramidal neurons in the peri-infarct cortex and in the corresponding contralateral cortex. Quantitative analysis yielded a decrease in the mean length of the apical dendrites and an increase of the basilar dendrites in the peri-infarct cortex of the G-CSF group which was associated with significantly improved functional outcome in this group as recently shown [6]. However, the combined G-CSF and CIMT groups with a similar degree of functional recovery [6] lacked respective changes in the basilar dendritic length. In the corresponding contralateral cortex, a significant increase of the mean dendritic length of the apical dendrites of both G-CSF and CIMT treated rats without alterations of the basilar dendrites was seen (A, B, D, significant difference compared with control, CIMT, CIMT+G-CSF, respectively; values are expressed as length/neuron in μm ± SEM; p<0.05).

**Fig 4:**
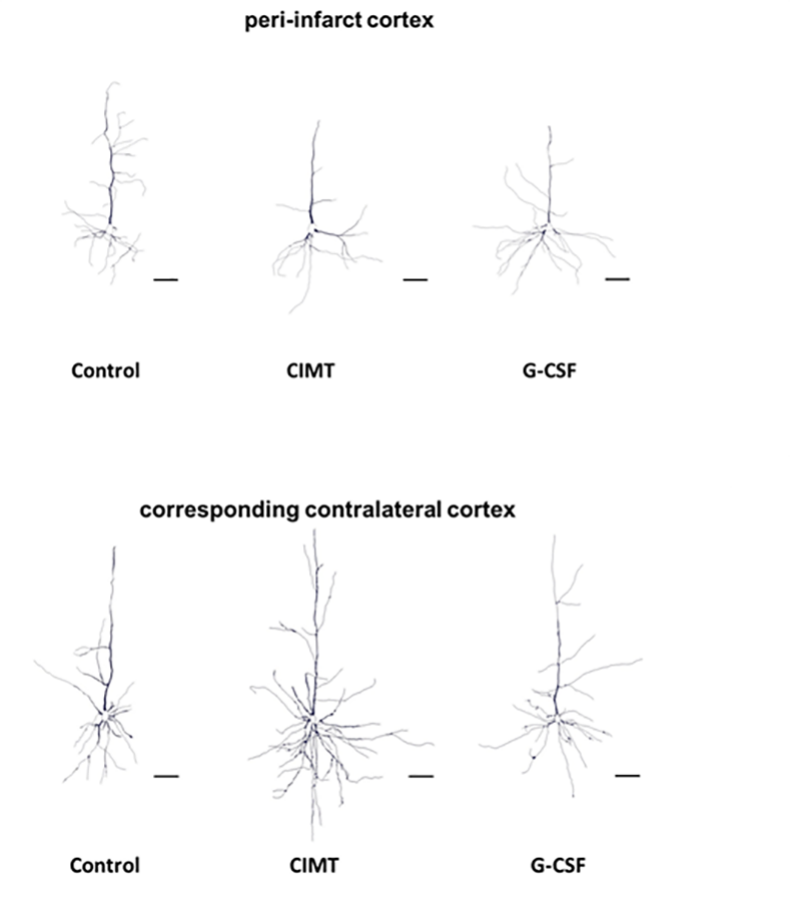
Representative pictures of Golgi Cox-impregnated layer V pyramidal neurons of the control, CIMT and G-CSF-treated group in the peri-infarct cortex (A) and the corresponding contralateral cortex (B), respectively. Note the increase of the basilar dendritic arbor in the peri-infarct cortex of G-CSF treated rats. Scale bar: 50 μm.

Dendritic plasticity of layer V pyramidal neurons was analyzed in the peri-infarct cortex and the corresponding contralateral cortex in each treatment group (Fig 1) (peri-infarct cortex: control n = 31; CIMT n = 32, G-CSF n = 30, CIMT+G-CSF n = 27, CIMT/G-CSF n = 26; corresponding contralateral cortex: control n = 49; CIMT n = 49, G-CSF n = 50, CIMT+G-CSF n = 42, CIMT/G-CSF n = 35). There were no differences in the number of basilar dendrites between the various experimental groups neither in the peri-infarct cortex nor in the corresponding homotopic contralateral cortex (Fig 2). However, in the G-CSF only group the mean length of the apical dendrite was significantly decreased compared to control and CIMT only group while the length of the basilar dendrites was significantly increased in the peri-infarct cortex (Figs 3 and 4). With the exception of a decrease of the length of the apical dendrite in the peri-infarct cortex of the concomitant CIMT + G-CSF group no further differences were present (Fig 3). On the corresponding contralateral cortex the length of the apical dendrites was increased both in the CIMT and the G-CSF group compared to control and the concurrent CIMT + G-CSF group. The basilar dendrites on the contralateral side did not show any significant differences of mean length by the different treatment paradigms (Figs 3 and 4).

#### Dendritic arborization–intersection and branch order analysis

**Fig 5:**
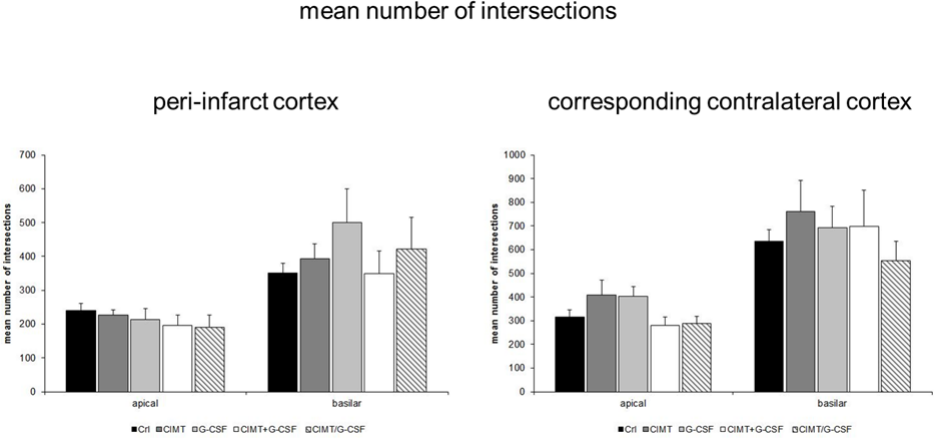
Intersection analysis of Golgi Cox-impregnated layer V pyramidal neurons in the peri-infarct cortex and in the corresponding contralateral cortex. No significant differences in the mean number of intersections of apical and basilar dendrites between the various experimental groups were detectable (Values are expressed as mean number of intersections ± SEM).

**Fig 6:**
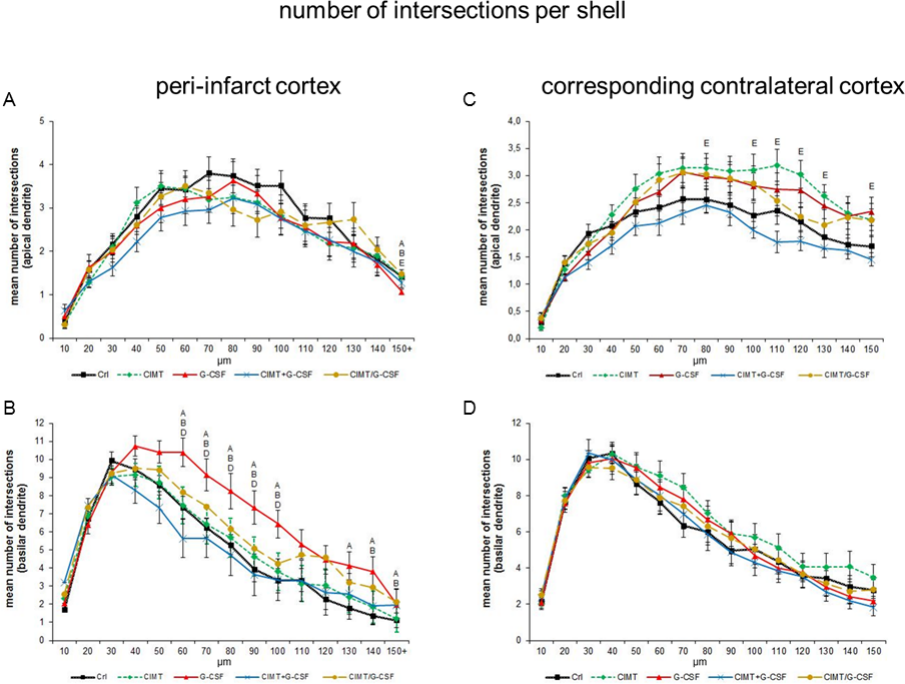
Sholl analysis of Golgi Cox-impregnated layer V pyramidal neurons in the peri-infarct cortex and in the corresponding contralateral cortex. Note that corresponding to the increased length of basilar dendrites (c.f. Fig 3) a significantly increased number of intersections of higher order in the G-CSF group of the peri-infarct cortex were detectable (B) which paralleled the functional outcome as recently published [6] (A, B, D, E significant difference compared with control, CIMT, CIMT+G-CSF, CIMT/G-CSF, respectively. Values are expressed as mean number of intersections / μm ± SEM; p<0.05).

*Intersection analysis*. Regarding the branching of traced neurons there were no significant changes in the mean number of intersections neither in the peri-infarct cortex nor in the corresponding contralateral cortex when comparing all treatment groups (Fig 5). Further analysis of the apical and basilar dendritic tree on each side revealed a complex pattern. The most obvious changes were a significantly increase in the number of intersections of the basilar dendrites 60–100 μm from the soma in the peri-infarct cortex of G-CSF treated rats compared to control (Fig 6).

*Branch order analysis*. The branch order analysis of apical and basilar dendrites in the peri-infarct and corresponding contralateral cortex revealed only a few treatment specific pattern. The number of basilar dendrites of third, 4^th^ and 5^th^ order was increased in the peri-infarct cortex of G-CSF treated rats compared to control. Furthermore, the mean length of these dendrites was also increased in those of 3rd and 5^th^ order in the G-CSF only group but also 2nd to 5^th^ order dendrites of the sequential CIMT and G-CSF group (not shown). In the corresponding contralateral cortex the mean number of apical dendrites per branch order was unchanged in the sole treatment groups while for the mean length of these dendrites a heterogeneous pattern of single significant alterations throughout all groups and branch orders was visible (not shown). There were no significant differences between the various experimental groups concerning the mean number of basilar dendrites per branch order in the corresponding contralateral cortex, while 3rd order basilar dendrites of G-CSF treated rats were decreased. Basilar dendrites further showed an increase in the mean length of 4^th^ and 5^th^ order dendrites in the concomitant CIMT/G-CSF group (not shown).

## Discussion

As previously shown, G-CSF treatment alone, or in combination with CIMT either sequentially or concomitantly, resulted in significantly improved sensorimotor recovery after photothrombotic stroke in rats in comparison to untreated ischemic control animals, while CIMT alone did not result in any significant differences compared to untreated ischemic controls [6]. This failure of a significant benefit in the CIMT only group was associated with a shift from excitation toward inhibition in peri-ischemic and remote cortical regions [6]. Using brains from this former experiment the present study was designed to evaluate whether improved neurological outcome would be paralleled by an increase in dendritic plasticity of layer V pyramidal neurons in the peri-infarct and/or the respective contralateral homotopic cortex.

Surprisingly, a complex and different pattern of dendritic plasticity in the various treatment groups became apparent which can be summarized as follows: (i) a decrease in the mean length of the apical dendrites and an increase of the basilar dendrites in the peri-infarct cortex of the G-CSF group which was associated with a significantly increased number of intersections of higher order; (ii) a significant increase of the mean dendritic length of the apical dendrites in the contralateral cortex of both G-CSF and CIMT treated rats without alterations of the basilar dendrites; (iii) a heterogeneous pattern in the groups with combined treatment.

One crucial issue when looking for recovery-associated structural changes after ischemic stroke is the selection of regions to be analyzed. Both in the peri-ischemic as well as in the contralateral homotopic cortex plastic changes have been identified which are thought to represent the structural correlate of spontaneous post-stroke recovery frequently observed in rodents and also in humans [12, 25–28]. Although there is consensus that these regions are potential targets for therapeutic intervention to enhance the spontaneously occurring regenerative processes it is contentiously discussed which of both is more relevant. Theoretically, the surviving peri-infarct cortex represents a particularly ideal area for plastic processes due to pronounced changes in the post-stroke milieu with dramatic changes in the expression of growth promoting or inhibiting factors. However, studies analyzing peri-infarct dendritic plasticity are limited and the results are partly heterogeneous. The bulk of evidence, however, suggests that ischemia indeed induces increased peri-lesional dendritic plasticity which may be enhanced by various therapies. The comparability of studies on dendritic plasticity after stroke is further complicated by the fact that some studies analyzed apical dendrites, whereas other studies focused on basilar dendrites which may result in completely different findings.

### Basilar Dendrites

In a permanent MCAO model in rats Golgi Cox staining revealed increased perifocal basilar dendrites after 56 days, while at 7 days, number and diameter of basilar dendrites was decreased [18]. The hypothesis that this spontaneously occurring plasticity may attributable to therapeutic intervention is supported from a number of studies. Using Golgi Cox staining, Gonzalesz et al. [29] could show that chronic low-dose administration of nicotine after focal cortical ischemia in rats enhanced basilar dendritic branching of layer 5 pyramidal neurons in the adjacent anterior cingulate cortex which was associated with increased functional outcome. Vice versa, in a rat model of motor cortex injury using a unilateral suction lesion rats with blockade of the endogenous bFGF expression resulted in worse recovery compared to the control treated group. Impaired function was associated with an atrophy of the basilar dendritic arbor in layer V pyramidal cells of the neighboring cortex analyzed by the Golgi Cox method [30]. Our results with increased length and enhanced branching of basilar dendrites in the G-CSF group with significantly enhanced neurological outcome [6] are largely in line with these studies. However, one has to note that this association was completely lost when looking at the groups with combined therapy consisting of CIMT and concomitant or sequential G-CSF application which exhibited significantly improved functional outcome [6], but did not show respective changes in basilar dendrites (Figs 3 and 6). The heterogeneity of these findings is reminiscent to a study [13] which thoroughly compared behavioral outcome and dendritic plasticity in three models of permanent ischemia and a cortical aspiration model in rats resulting in sensorimotor loss related to damage in the territory of the MCA. Not so surprisingly, the neurological impairment due to motor cortex injury was similar across the different models. However, there were striking differences in dendritic plasticity. While in the devascularization group the lesions were associated with increased dendritic arborization, spine density was increased in the aspiration group [13]. Obviously, there are completely different ways and different patterns of synaptic reorganization in which the brain can respond to injury to enhance recovery.

### Apical Dendrites

Using in vivo two-photon microscopy, Mostany and Portera-Cailliau [28] did not find any large-scale dendritic plasticity of layer V pyramidal neurons. Instead, a two-step pruning process with an initial decrease in apical dendritic length followed by loss of dendritic branches has been observed. This “pruning” of apical dendrites in the peri-infarct regions is considered as an active process rather than a passive degradation to avoid circuit dysfunction since the progressive contortion of structures over time could have a negative impact on recovery [27]. This would be true particularly for dendrites trying to maintain connections with presynaptic terminals that may have arisen from distant regions of the brain e.g. transcallosal projections from the intact hemisphere [31]. This view is supported by the fact that the apical dendrites are connected primarily to axons from remote or even contralateral neurons in contrast to the basilar dendrites which connect with surrounding neurons [18, 32]. This would also be a plausible explanation for the increase in dendritic length of the apical dendrite in the homotopic contralateral cortex in the G-CSF and CIMT group. Although not investigated in this study a significant correlation between interhemispheric axonal sprouting originating from the peri-infarct motor cortex after ischemic stroke in the motor cortex and functional improvement has been convincingly demonstrated in a study in rats by Liu and colleagues [33].

In conclusion, animals with a similar degree of behavioral recovery exhibited quite different patterns of dendritic plasticity in both peri-lesional and contralesional cortical areas. A reasonable explanation would be that completely different therapeutic strategies like a pharmacotherapy with the growth factor G-CSF and CIMT which is directed at function as in our previous study are highly likely to trigger different mechanisms eventually resulting in a different distribution of plastic changes. The situation is further complicated by a combination of these strategies either in a concomitant or sequential application. Although we were absolutely aware of this issue, the experimental design of our study was primarily chosen to possibly identify a therapy superior to the others. In a second step we looked whether specific patterns of dendritic plasticity would be associated with improved neurological outcome. It proved that this is only partly the case for the single therapy groups. Another explanation for lack of an association between function and dendritic morphology is the highly underestimated and frequently ignored problem to differentiate between recovery and compensatory movements (for an excellent introduction into this issue see [34]). Ideally, post-stroke therapies would enhance the spontaneously occurring recovery which is thought to take place in particular in the peri-ischemic brain but will also involve functionally connected regions of the contralateral hemisphere [25]. However, a clear distinction from plastic changes induced by compensatory motor patterns is frequently not possible.

## Supporting Information

S1 Table Neuron summary ipsilateral layer V. (PDF)

S2 Table Neuron summary contralateral layer V. (PDF)
