# Supplementary material for: Functional Improvement after Photothrombotic Stroke in Rats Is Associated with Different Patterns of Dendritic Plasticity after G-CSF Treatment and G-CSF Treatment Combined with Concomitant or Sequential Constraint-Induced Movement Therapy
Source: PLoS One. 2016 Jan 11;11(1):e0146679. doi: 10.1371/journal.pone.0146679 (PMC4713830; doi:10.1371/journal.pone.0146679)
Supplement: S1 Table — (PDF) [file pone.0146679.s001.pdf]

Data Figure 2

Basilar dendrites peri-infarct cortex

Group	number		Qty	Length (µm)	mean length (µm)
Control	215-9-1i	basilar	7	669,9	95,7
Control	215-11-1i	basilar	7	789,1	112,7
Control	215-15-1i	basilar	10	959,4	95,9
Control	215-16-1i	basilar	6	1048	174,7
Control	215-18-1i	basilar	6	866,4	144,4
Control	216-6-1i	basilar	6	1763,5	293,9
Control	216-8-1i	basilar	5	1154,9	231
Control	216-12-1i	basilar	6	303,1	50,5
Control	216-18-1i	basilar	6	402	67
Control	216-19-1i	basilar	6	348,1	58
Control	216-22-1i	basilar	4	615,2	153,8
Control	219-10-1i	basilar	4	989	247,2
Control	219-14-1i	basilar	6	1446,3	241
Control	219-15-1i	basilar	6	1038,9	173,1
Control	219-17-1i	basilar	5	986	197,2
Control	219-21-1i	basilar	6	1628,3	271,4
Control	297-19-1i	basilar	6	1084,7	180,8
Control	297-19-2i	basilar	5	512,6	102,5
Control	297-20-1i	basilar	9	1479,8	164,4
Control	297-22-1i	basilar	6	657,5	109,6
Control	297-22-2i	basilar	6	1138,8	189,8
Control	298-15-1i	basilar	8	1141,8	142,7
Control	298-15-2i	basilar	6	592,5	98,7
Control	298-16-1i	basilar	3	288,1	96
Control	298-17-1i	basilar	5	948	189,6
Control	298-18-1i	basilar	5	924,4	184,9
Control	308-17-1i	basilar	7	1291,7	184,5
Control	308-18-1i	basilar	10	1277,8	127,8
Control	308-18-2i	basilar	12	932	77,7
Control	308-21-1i	basilar	7	603,3	86,2
Control	308-21-2i	basilar	7	959,8	137,1
CIMT	211-10-1i	basilar	6	2019,4	336,6
CIMT	211-13-1i	basilar	7	1115,3	159,3
CIMT	211-13-2i	basilar	3	729	243
CIMT	211-14-1i	basilar	5	934,2	186,8
CIMT	211-14-2i	basilar	6	566,9	94,5
CIMT	211-15-1i	basilar	6	794,3	132,4
CIMT	211-15-2i	basilar	4	1000	250
CIMT	212-13-1i	basilar	4	542,4	135,6
CIMT	212-13-2i	basilar	4	621	155,3
CIMT	212-14-1i	basilar	6	538,6	89,8
CIMT	212-14-2i	basilar	8	967,3	120,9
CIMT	212-15-1i	basilar	3	491,1	163,7
CIMT	218-11-1i	basilar	5	738,5	147,7
CIMT	218-14-1i	basilar	7	831,8	118,8
CIMT	218-16-1i	basilar	5	1740,8	348,2
CIMT	218-17-1i	basilar	3	1068,2	356,1
CIMT	218-19-1i	basilar	3	1209,1	403
CIMT	299-14-1i	basilar	6	854,1	142,4
CIMT	299-14-2i	basilar	6	884,6	147,4

CIMT	299-15-1i	basilar	5	962,1	192,4
CIMT	299-16-1i	basilar	3	550,8	183,6
CIMT	299-17-1i	basilar	5	314,4	62,9
CIMT	300-16-1i	basilar	5	823,2	164,6
CIMT	300-20-1i	basilar	7	1169,8	167,1
CIMT	300-22-1i	basilar	4	1550	387,5
CIMT	300-24-1i	basilar	5	1030,3	206,1
CIMT	300-24-2i	basilar	5	1261,4	252,3
CIMT	309-15-1i	basilar	6	874,3	145,7
CIMT	309-15-2i	basilar	10	1016	101,6
CIMT	309-16-1i	basilar	5	653,3	130,7
CIMT	309-16-2i	basilar	10	2040,5	204,1
CIMT	309-17-1i	basilar	5	1333,6	266,7
CIMT+G-CSF	213-08-1i	basilar	5	907,8	181,6
CIMT+G-CSF	213-12-1i	basilar	6	386,5	64,4
CIMT+G-CSF	213-14-1i	basilar	6	280,6	46,8
CIMT+G-CSF	213-17-1i	basilar	7	1153,4	164,8
CIMT+G-CSF	213-18-1i	basilar	8	845,6	105,7
CIMT+G-CSF	214-14-1i	basilar	7	609,2	87
CIMT+G-CSF	214-14-2i	basilar	7	590,6	84,4
CIMT+G-CSF	214-17-1i	basilar	5	710,8	142,2
CIMT+G-CSF	214-18-1i	basilar	6	927	154,5
CIMT+G-CSF	214-19-1i	basilar	5	679,8	136
CIMT+G-CSF	217-6-1i	basilar	5	1211	242,2
CIMT+G-CSF	217-8-1i	basilar	4	1413,2	353,3
CIMT+G-CSF	217-10-1i	basilar	5	544,5	108,9
CIMT+G-CSF	217-16-1i	basilar	5	2581,7	516,3
CIMT+G-CSF	217-17-1i	basilar	7	2179,9	311,4
CIMT+G-CSF	304-17-1i	basilar	4	434,4	108,6
CIMT+G-CSF	304-19-1i	basilar	4	438,2	109,6
CIMT+G-CSF	304-19-2i	basilar	4	529,9	132,5
CIMT+G-CSF	304-20-1i	basilar	5	558,1	111,6
CIMT+G-CSF	304-21-1i	basilar	4	1378,9	344,7
CIMT+G-CSF	304-21-2i	basilar	5	388,6	77,7
CIMT+G-CSF	304-22-1i	basilar	6	644,4	107,4
CIMT+G-CSF	305-17-1i	basilar	6	765,5	127,6
CIMT+G-CSF	305-17-2i	basilar	4	850,7	212,7
CIMT+G-CSF	305-18-1i	basilar	7	379,1	54,2
CIMT+G-CSF	305-19-1i	basilar	5	710,7	142,1
CIMT+G-CSF	305-19-2i	basilar	6	787,8	131,3
CIMT/G-CSF	306-17-1i	basilar	6	542	90,3
CIMT/G-CSF	306-17-2i	basilar	8	1246,1	155,8
CIMT/G-CSF	306-18-1i	basilar	4	670,8	167,7
CIMT/G-CSF	306-18-2i	basilar	6	910,5	151,8
CIMT/G-CSF	306-18-3i	basilar	5	725,6	145,1
CIMT/G-CSF	311-16-1i	basilar	10	1288,8	128,9
CIMT/G-CSF	311-17-1i	basilar	6	3458,7	576,4
CIMT/G-CSF	311-17-2i	basilar	7	3068,7	438,4
CIMT/G-CSF	311-18-1i	basilar	5	823,8	164,8
CIMT/G-CSF	311-19-1i	basilar	8	2073,3	259,2
CIMT/G-CSF	313-14.1i	basilar	5	806,9	161,4
CIMT/G-CSF	313-15-1i	basilar	4	950,4	237,6
CIMT/G-CSF	313-17-1i	basilar	5	972,5	194,5
CIMT/G-CSF	313-19-1i	basilar	6	535,8	89,3
CIMT/G-CSF	313-19-2i	basilar	6	995,6	165,9
CIMT/G-CSF	314-16-1i	basilar	7	1711,2	244,5

CIMT/G-CSF	314-16-2i	basilar	3	515,9	172
CIMT/G-CSF	314-17-1i	basilar	4	1853,9	463,5
CIMT/G-CSF	314-17-2i	basilar	5	1149,1	229,8
CIMT/G-CSF	314-18-1i	basilar	3	198,3	66,1
CIMT/G-CSF	315-11-1i	basilar	8	597,9	74,7
CIMT/G-CSF	315-11-2i	basilar	8	757,5	94,7
CIMT/G-CSF	315-13-1i	basilar	3	1584,5	528,2
CIMT/G-CSF	315-13-2i	basilar	4	664,7	166,2
CIMT/G-CSF	315-14-1i	basilar	3	213,9	71,3
CIMT/G-CSF	315-14-2i	basilar	4	542	135,5
G-CSF	220-7-1i	basilar	5	1430,4	286,1
G-CSF	220-16-1i	basilar	5	785,8	157,2
G-CSF	220-17-1i	basilar	7	1139,9	162,8
G-CSF	220-18-1i	basilar	6	1246,1	207,7
G-CSF	220-19-1i	basilar	5	1608,7	321,7
G-CSF	221-6-1i	basilar	6	1636	272,7
G-CSF	221-13-1i	basilar	5	1336,2	267,2
G-CSF	221-16-1i	basilar	7	3276,6	468,1
G-CSF	221-19-1i	basilar	5	3331,9	666,4
G-CSF	221-22-1i	basilar	5	1655,7	331,1
G-CSF	222-8-1i	basilar	9	2017,7	224,2
G-CSF	222-16-1i	basilar	6	809,3	134,9
G-CSF	222-17-1i	basilar	6	1864,4	310,7
G-CSF	222-21-1i	basilar	5	1975,2	395
G-CSF	222-23-1i	basilar	8	1425,8	178,2
G-CSF	301-18-1i	basilar	3	289,3	96,4
G-CSF	301-19-1i	basilar	5	407,9	81,6
G-CSF	301-20-1i	basilar	8	724,6	90,6
G-CSF	301-20-2i	basilar	6	1701,3	283,6
G-CSF	301-20-3i	basilar	5	1976,4	395,3
G-CSF	302-16-1i	basilar	6	399,2	66,5
G-CSF	302-16-2i	basilar	5	187,4	37,5
G-CSF	302-17-1i	basilar	6	301,8	50,3
G-CSF	302-17-2i	basilar	5	724,4	144,9
G-CSF	302-18-1i	basilar	5	577,3	115,5
G-CSF	303-15-1i	basilar	4	992,9	248,2
G-CSF	303-16-1i	basilar	10	2119,3	211,9
G-CSF	303-16-2i	basilar	7	1038,5	148,4
G-CSF	303-17-1i	basilar	4	987	246,7
G-CSF	303-17-2i	basilar	4	692,9	173,2

Data Figure 3

Dendrite length per neuron peri-infarct cortex

Group	animal No	length basilar	number of neurons	length per neuron basilar per neuron	length apical	length per neuron apical per neuron
Control	215	4332,8	5	866,6	2819,2	563,8
Control	216	4586,8	6	764,5	3497,3	582,9
Control	219	6088,5	5	1217,7	3835,5	767,1
Control	297	4873,4	5	974,7	4077,6	815,5
Control	298	3894,8	6	649,1	4292,9	715,5
Control	308	5064,6	5	1012,9	3709,3	741,9
CIMT	211	7159,1	5	1431,8	3418,1	683,6
CIMT	212	3160,4	7	451,5	2708,8	387,0
CIMT	218	5588,4	5	1117,7	3088,8	617,8
CIMT	299	3566	5	713,2	3055,9	611,2
CIMT	300	5834,7	5	1166,9	2711,2	542,2
CIMT	309	5917,7	5	1183,5	4265,6	853,1
CIMT+G-CSF	213	3973,9	6	662,3	2515,2	419,2
CIMT+G-CSF	214	4017,4	5	803,5	2418,6	483,7
CIMT+G-CSF	217	7930,3	7	1132,9	4743,1	677,6
CIMT+G-CSF	304	4372,5	5	874,5	2002,1	400,4
CIMT+G-CSF	305	3673,8	5	734,8	2297,8	459,6
CIMT/G-CSF	306	4095	5	819,0	2687,7	537,5
CIMT/G-CSF	311	10713,3	5	2142,7	4456,8	891,4
CIMT/G-CSF	313	4261,2	5	852,2	2940,7	588,1
CIMT/G-CSF	314	5428,4	5	1085,7	3384,5	676,9
CIMT/G-CSF	315	4360,5	6	726,8	2911,7	485,3
G-CSF	220	6210,9	5	1242,2	2499,2	499,8
G-CSF	221	11236,4	5	2247,3	2719,5	543,9
G-CSF	222	8092,4	5	1618,5	3145,2	629,0
G-CSF	301	5099,5	4	1274,9	1694,7	423,7
G-CSF	302	3190,1	5	638	1821,2	364,2
G-CSF	303	5830,6	5	1166,1	2974,7	594,9

Data Figure 5

Intersections per Group

Group	number	No of neurons	Qty basilar	Qty per neuron basilar	Qty apical	Qty per neuron apical
Control	215	5	312	62,4	150	30,0
Control	216	6	328	54,7	226	37,7
Control	219	5	495	99,0	241	48,2
Control	297	5	339	67,8	257	51,4
Control	298	6	293	48,8	304	50,7
Control	308	5	337	67,4	259	51,8
CIMT	211	5	523	104,6	220	44,0
CIMT	212	7	246	35,1	204	29,1
CIMT	299	5	271	54,2	222	44,4
CIMT	300	5	437	87,4	213	42,6
CIMT	309	5	456	91,2	196	39,2
CIMT	218	5	423	84,6	301	60,2
CIMT+G-CSF	213	6	282	47,0	184	30,7
CIMT+G-CSF	214	5	263	52,6	252	50,4
CIMT+G-CSF	217	7	609	87,0	220	31,4
CIMT+G-CSF	304	5	342	68,4	240	48,0
CIMT+G-CSF	305	5	254	50,8	85	17,0
CIMT/G-CSF	306	5	295	59,0	204	40,8
CIMT/G-CSF	311	5	786	157,2	154	30,8
CIMT/G-CSF	313	5	297	59,4	155	31,0
CIMT/G-CSF	314	5	403	80,6	327	65,4
CIMT/G-CSF	315	6	327	54,5	110	18,3
G-CSF	220	5	459	91,8	137	27,4
G-CSF	221	5	880	176,0	166	33,2
G-CSF	222	5	653	130,6	361	72,2
G-CSF	301	4	401	100,3	204	51,0
G-CSF	302	5	176	35,2	224	44,8
G-CSF	303	5	435	87,0	190	38,0

Group	Number	No of neurons	Qty total	Qty per neuron total
Control	215	5	462	92,4
Control	216	6	554	92,3
Control	219	5	736	147,2
Control	297	5	596	119,2
Control	298	6	597	99,5
Control	308	5	596	119,2
CIMT	211	5	743	148,6
CIMT	212	7	450	64,3
CIMT	299	5	493	98,6
CIMT	300	5	650	130,0
CIMT	309	5	652	130,4
CIMT	218	5	724	144,8
CIMT+G-CSF	213	6	466	77,7
CIMT+G-CSF	214	5	515	103,0
CIMT+G-CSF	217	7	829	118,4
CIMT+G-CSF	304	5	582	116,4
CIMT+G-CSF	305	5	339	67,8
CIMT/G-CSF	306	5	499	99,8
CIMT/G-CSF	311	5	940	188,0
CIMT/G-CSF	313	5	452	90,4

CIMT/G-CSF	314	5	730	146,0
CIMT/G-CSF	315	6	437	72,8
G-CSF	220	5	596	119,2
G-CSF	221	5	1046	209,2
G-CSF	222	5	1014	202,8
G-CSF	301	4	605	151,3
G-CSF	302	5	400	80,0
G-CSF	303	5	625	125,0

Data Figure 6

apical dendrite Sholl 10µm

Group	number	Radius(µm)	Intersections	Length(µm)
Control	215-11-1i	10	0	0
Control	215-15-1i	10	1	3,1
Control	215-16-1i	10	1	2
Control	215-18-1i	10	1	2,2
Control	215-9-1i	10	1	1,3
Control	216-12-1i	10	1	1,6
Control	216-18-1i	10	0	0
Control	216-19-1i	10	0	0
Control	216-22-1i	10	0	0
Control	216-6-1i	10	0	0
Control	216-8-1i	10	1	0,1
Control	219-10-1i	10	0	0
Control	219-14-1i	10	0	0
Control	219-15-1i	10	0	0
Control	219-17-1i	10	0	0
Control	219-21-1i	10	0	0
Control	297-19-1i	10	0	0
Control	297-19-2i	10	0	0
Control	297-20-1i	10	0	0
Control	297-22-1i	10	0	0
Control	297-22-2i	10	0	0
Control	298-15-1i	10	1	1,8
Control	298-15-2i	10	1	3,5
Control	298-16-1i	10	0	0
Control	298-17-1i	10	1	0,2
Control	298-18-1i	10	0	0
Control	308-17-1i	10	0	0
Control	308-18-1i	10	1	4
Control	308-18-2i	10	0	0
Control	308-21-1i	10	0	0
Control	308-21-2i	10	2	2,5
CIMT	211-10-1i	10	0	0
CIMT	211-13-1i	10	1	1,2
CIMT	211-13-2i	10	1	2,7
CIMT	211-14-1i	10	0	0
CIMT	211-14-2i	10	0	0
CIMT	211-15-1i	10	0	0
CIMT	211-15-2i	10	0	0
CIMT	212-13-1i	10	1	0,7

CIMT	212-13-2i	10	0	0
CIMT	212-14-1i	10	0	0
CIMT	212-14-2i	10	1	1,7
CIMT	212-15-1i	10	1	3,7
CIMT	218-11-1i	10	1	2,5
CIMT	218-14-1i	10	0	0
CIMT	218-16-1i	10	0	0
CIMT	218-17-1i	10	0	0
CIMT	218-19-1i	10	0	0
CIMT	299-14-1i	10	1	0,7
CIMT	299-14-2i	10	0	0
CIMT	299-15-1i	10	1	0
CIMT	299-16-1i	10	0	0
CIMT	299-17-1i	10	0	0
CIMT	300-16-1i	10	1	2,5
CIMT	300-20-1i	10	0	0
CIMT	300-22-1i	10	0	0
CIMT	300-24-1i	10	0	0
CIMT	300-24-2i	10	0	0
CIMT	309-15-1i	10	0	0
CIMT	309-15-2i	10	0	0
CIMT	309-16-1i	10	1	7,1
CIMT	309-16-2i	10	0	0
CIMT	309-17-1i	10	0	0
CIMT+G-CSF	213-12-1i	10	1	2,4
CIMT+G-CSF	213-14-1i	10	3	12,2
CIMT+G-CSF	213-17-1i	10	1	0,9
CIMT+G-CSF	213-18-1i	10	0	0
CIMT+G-CSF	213-8-1i	10	0	0
CIMT+G-CSF	214-14-1i	10	0	0
CIMT+G-CSF	214-14-2i	10	0	0
CIMT+G-CSF	214-17-1i	10	0	0
CIMT+G-CSF	214-18-1i	10	1	2,2
CIMT+G-CSF	214-19-1i	10	0	0
CIMT+G-CSF	217-10-1i	10	1	3,2
CIMT+G-CSF	217-16-1i	10	0	0
CIMT+G-CSF	217-17-1i	10	0	0
CIMT+G-CSF	217-6-1i	10	0	0
CIMT+G-CSF	217-8-1i	10	1	1,3
CIMT+G-CSF	304-17-1i	10	1	8
CIMT+G-CSF	304-19-1i	10	2	0,1
CIMT+G-CSF	304-19-2i	10	0	0
CIMT+G-CSF	304-20-1i	10	1	5
CIMT+G-CSF	304-21-1i	10	0	0
CIMT+G-CSF	304-21-2i	10	1	0,9
CIMT+G-CSF	304-22-1i	10	1	6,7
CIMT+G-CSF	305-17-1i	10	1	0,6
CIMT+G-CSF	305-17-2i	10	1	1,1
CIMT+G-CSF	305-18-1i	10	0	0
CIMT+G-CSF	305-19-1i	10	1	2,2
CIMT+G-CSF	305-19-2i	10	0	0
CIMT/G-CSF	306-17-1i	10	1	1,9
CIMT/G-CSF	306-17-2i	10	0	0
CIMT/G-CSF	306-18-1i	10	0	0
CIMT/G-CSF	306-18-2i	10	1	1,4
CIMT/G-CSF	306-18-3i	10	0	0

CIMT/G-CSF	311-16-1i	10	0	0
CIMT/G-CSF	311-17-1i	10	0	0
CIMT/G-CSF	311-17-2i	10	0	0
CIMT/G-CSF	311-18-1i	10	0	0
CIMT/G-CSF	311-19-1i	10	0	0
CIMT/G-CSF	313-14.1i	10	1	8,5
CIMT/G-CSF	313-15-1i	10	0	0
CIMT/G-CSF	313-17-1i	10	0	0
CIMT/G-CSF	313-19-1i	10	1	1,9
CIMT/G-CSF	313-19-2i	10	1	0,4
CIMT/G-CSF	314-16-1i	10	1	2,1
CIMT/G-CSF	314-16-2i	10	0	0
CIMT/G-CSF	314-17-1i	10	0	0
CIMT/G-CSF	314-17-2i	10	0	0
CIMT/G-CSF	314-18-1i	10	1	1,9
CIMT/G-CSF	315-11-1i	10	0	0
CIMT/G-CSF	315-11-2i	10	0	0
CIMT/G-CSF	315-13-1i	10	0	0
CIMT/G-CSF	315-13-2i	10	0	0
CIMT/G-CSF	315-14-1i	10	0	0
CIMT/G-CSF	315-14-2i	10	1	0,8
G-CSF	220-16-1i	10	0	0
G-CSF	220-17-1i	10	1	0,5
G-CSF	220-18-1i	10	0	0
G-CSF	220-19-1i	10	0	0
G-CSF	220-7-1i	10	0	0
G-CSF	221-13-1i	10	0	0
G-CSF	221-16-1i	10	0	0
G-CSF	221-19-1i	10	0	0
G-CSF	221-22-1i	10	0	0
G-CSF	221-6-1i	10	1	1,2
G-CSF	222-16-1i	10	0	0
G-CSF	222-17-1i	10	0	0
G-CSF	222-21-1i	10	0	0
G-CSF	222-23-1i	10	1	0,6
G-CSF	222-8-1i	10	0	0
G-CSF	301-18-1i	10	5	12
G-CSF	301-19-1i	10	1	0,4
G-CSF	301-20-1i	10	0	0
G-CSF	301-20-2i	10	0	0
G-CSF	301-20-3i	10	0	0
G-CSF	302-16-1i	10	0	0
G-CSF	302-16-2i	10	1	3,8
G-CSF	302-17-1i	10	1	4,6
G-CSF	302-17-2i	10	1	1,7
G-CSF	302-18-1i	10	1	0,4
G-CSF	303-15-1i	10	0	0
G-CSF	303-16-1i	10	0	0
G-CSF	303-16-2i	10	1	1,2
G-CSF	303-17-1i	10	1	0
G-CSF	303-17-2i	10	0	0

apical dendrite Sholl 20µm

Group	number	Radius(µm)	Intersections	Length(µm)
Control	215-11-1i	20	1	13,3
Control	215-15-1i	20	1	10,6
Control	215-16-1i	20	2	15,5
Control	215-18-1i	20	2	17,8
Control	215-9-1i	20	4	85,9
Control	216-12-1i	20	1	27,4
Control	216-18-1i	20	1	29,7
Control	216-19-1i	20	1	7,6
Control	216-22-1i	20	1	15,4
Control	216-6-1i	20	0	0
Control	216-8-1i	20	2	28,2
Control	219-10-1i	20	1	5,4
Control	219-14-1i	20	1	9,2
Control	219-15-1i	20	2	15,9
Control	219-17-1i	20	1	5,3
Control	219-21-1i	20	1	10,1
Control	297-19-1i	20	1	7,9
Control	297-19-2i	20	1	9
Control	297-20-1i	20	1	9,3
Control	297-22-1i	20	2	31,5
Control	297-22-2i	20	6	18,7
Control	298-15-1i	20	2	21,1
Control	298-15-2i	20	1	10,3
Control	298-16-1i	20	1	6,6
Control	298-17-1i	20	3	23,8
Control	298-18-1i	20	1	1,1
Control	308-17-1i	20	1	11,1
Control	308-18-1i	20	1	11,8
Control	308-18-2i	20	1	10,4
Control	308-21-1i	20	2	22,5
Control	308-21-2i	20	3	39,7
CIMT	211-10-1i	20	0	0
CIMT	211-13-1i	20	1	10,5
CIMT	211-13-2i	20	1	10,6
CIMT	211-14-1i	20	1	8,9
CIMT	211-14-2i	20	1	10,3
CIMT	211-15-1i	20	1	9,8
CIMT	211-15-2i	20	1	8,7
CIMT	212-13-1i	20	1	10,8
CIMT	212-13-2i	20	1	8,6
CIMT	212-14-1i	20	2	14
CIMT	212-14-2i	20	2	14,1
CIMT	212-15-1i	20	1	10,3
CIMT	218-11-1i	20	2	30,8
CIMT	218-14-1i	20	1	9,7
CIMT	218-16-1i	20	0	0
CIMT	218-17-1i	20	1	6,9
CIMT	218-19-1i	20	1	9,6
CIMT	299-14-1i	20	1	11,8
CIMT	299-14-2i	20	2	21,6
CIMT	299-15-1i	20	1	11,2
CIMT	299-16-1i	20	1	10,6

CIMT	299-17-1i	20	1	8,3
CIMT	300-16-1i	20	1	10,6
CIMT	300-20-1i	20	2	22,3
CIMT	300-22-1i	20	1	10
CIMT	300-24-1i	20	1	9,4
CIMT	300-24-2i	20	2	25,2
CIMT	309-15-1i	20	1	9,9
CIMT	309-15-2i	20	3	28,5
CIMT	309-16-1i	20	2	16,5
CIMT	309-16-2i	20	2	9,5
CIMT	309-17-1i	20	2	19,4
CIMT+G-CSF	213-12-1i	20	3	35,3
CIMT+G-CSF	213-14-1i	20	1	27,4
CIMT+G-CSF	213-17-1i	20	1	10,2
CIMT+G-CSF	213-18-1i	20	1	9,7
CIMT+G-CSF	213-8-1i	20	3	19,9
CIMT+G-CSF	214-14-1i	20	1	8,7
CIMT+G-CSF	214-14-2i	20	2	17,8
CIMT+G-CSF	214-17-1i	20	1	11,5
CIMT+G-CSF	214-18-1i	20	3	28,5
CIMT+G-CSF	214-19-1i	20	1	1,8
CIMT+G-CSF	217-10-1i	20	1	10,6
CIMT+G-CSF	217-16-1i	20	0	0
CIMT+G-CSF	217-17-1i	20	1	8,6
CIMT+G-CSF	217-6-1i	20	2	19,4
CIMT+G-CSF	217-8-1i	20	2	12,5
CIMT+G-CSF	304-17-1i	20	1	14,3
CIMT+G-CSF	304-19-1i	20	1	11
CIMT+G-CSF	304-19-2i	20	1	12
CIMT+G-CSF	304-20-1i	20	1	11,6
CIMT+G-CSF	304-21-1i	20	1	10,1
CIMT+G-CSF	304-21-2i	20	1	11,3
CIMT+G-CSF	304-22-1i	20	1	10,1
CIMT+G-CSF	305-17-1i	20	1	13,5
CIMT+G-CSF	305-17-2i	20	1	11,7
CIMT+G-CSF	305-18-1i	20	1	10,5
CIMT+G-CSF	305-19-1i	20	1	10,2
CIMT+G-CSF	305-19-2i	20	1	11,8
CIMT/G-CSF	306-17-1i	20	1	10,2
CIMT/G-CSF	306-17-2i	20	1	11
CIMT/G-CSF	306-18-1i	20	1	11
CIMT/G-CSF	306-18-2i	20	1	11,3
CIMT/G-CSF	306-18-3i	20	1	10,4
CIMT/G-CSF	311-16-1i	20	1	11
CIMT/G-CSF	311-17-1i	20	1	8,7
CIMT/G-CSF	311-17-2i	20	1	5,6
CIMT/G-CSF	311-18-1i	20	2	15,9
CIMT/G-CSF	311-19-1i	20	1	9,8
CIMT/G-CSF	313-14.1i	20	2	19,8
CIMT/G-CSF	313-15-1i	20	4	33,5
CIMT/G-CSF	313-17-1i	20	2	21,5
CIMT/G-CSF	313-19-1i	20	1	12,4
CIMT/G-CSF	313-19-2i	20	2	29,6
CIMT/G-CSF	314-16-1i	20	2	15,8
CIMT/G-CSF	314-16-2i	20	5	63,9
CIMT/G-CSF	314-17-1i	20	1	11,8

CIMT/G-CSF	314-17-2i	20	1	9,5
CIMT/G-CSF	314-18-1i	20	1	11,6
CIMT/G-CSF	315-11-1i	20	1	8,4
CIMT/G-CSF	315-11-2i	20	2	19,9
CIMT/G-CSF	315-13-1i	20	1	10
CIMT/G-CSF	315-13-2i	20	2	24,5
CIMT/G-CSF	315-14-1i	20	1	17,7
CIMT/G-CSF	315-14-2i	20	2	13,2
G-CSF	220-16-1i	20	2	15,7
G-CSF	220-17-1i	20	2	13,3
G-CSF	220-18-1i	20	1	11,1
G-CSF	220-19-1i	20	0	0
G-CSF	220-7-1i	20	2	21,3
G-CSF	221-13-1i	20	2	16,6
G-CSF	221-16-1i	20	1	7,7
G-CSF	221-19-1i	20	1	7,3
G-CSF	221-22-1i	20	1	9,1
G-CSF	221-6-1i	20	1	10,3
G-CSF	222-16-1i	20	1	9,9
G-CSF	222-17-1i	20	1	9,5
G-CSF	222-21-1i	20	1	9,9
G-CSF	222-23-1i	20	3	14,4
G-CSF	222-8-1i	20	1	10,3
G-CSF	301-18-1i	20	9	131,3
G-CSF	301-19-1i	20	1	10,8
G-CSF	301-20-1i	20	1	8,5
G-CSF	301-20-2i	20	1	12,5
G-CSF	301-20-3i	20	1	12,8
G-CSF	302-16-1i	20	1	10,7
G-CSF	302-16-2i	20	4	33
G-CSF	302-17-1i	20	1	10,6
G-CSF	302-17-2i	20	1	10,8
G-CSF	302-18-1i	20	1	10,1
G-CSF	303-15-1i	20	1	8,6
G-CSF	303-16-1i	20	2	31,7
G-CSF	303-16-2i	20	2	23,4
G-CSF	303-17-1i	20	1	16
G-CSF	303-17-2i	20	2	40

apical dendrite Sholl 30µm

Group	number	Radius(µm)	Intersections	Length(µm)
Control	215-11-1i	30	1	10,3
Control	215-15-1i	30	1	10,6
Control	215-16-1i	30	2	31
Control	215-18-1i	30	3	42,5
Control	215-9-1i	30	4	53,3
Control	216-12-1i	30	2	26
Control	216-18-1i	30	1	11,1
Control	216-19-1i	30	1	10,1
Control	216-22-1i	30	2	22
Control	216-6-1i	30	4	15,1
Control	216-8-1i	30	2	27,9
Control	219-10-1i	30	2	26,4
Control	219-14-1i	30	1	12,6

Control	219-15-1i	30	2	33
Control	219-17-1i	30	1	32,8
Control	219-21-1i	30	2	17,5
Control	297-19-1i	30	2	26
Control	297-19-2i	30	1	10,5
Control	297-20-1i	30	1	10,5
Control	297-22-1i	30	2	27
Control	297-22-2i	30	7	98,5
Control	298-15-1i	30	2	24,7
Control	298-15-2i	30	2	31,8
Control	298-16-1i	30	1	13,5
Control	298-17-1i	30	2	26,8
Control	298-18-1i	30	3	37,1
Control	308-17-1i	30	3	28,4
Control	308-18-1i	30	1	14,4
Control	308-18-2i	30	2	25
Control	308-21-1i	30	5	79,1
Control	308-21-2i	30	2	35,6
CIMT	211-10-1i	30	2	1,9
CIMT	211-13-1i	30	1	10,1
CIMT	211-13-2i	30	3	49,9
CIMT	211-14-1i	30	2	32,4
CIMT	211-14-2i	30	1	10,5
CIMT	211-15-1i	30	1	10,3
CIMT	211-15-2i	30	1	10,9
CIMT	212-13-1i	30	1	14,6
CIMT	212-13-2i	30	2	26,2
CIMT	212-14-1i	30	4	77,1
CIMT	212-14-2i	30	2	30
CIMT	212-15-1i	30	1	10,4
CIMT	218-11-1i	30	3	31,5
CIMT	218-14-1i	30	1	11
CIMT	218-16-1i	30	1	9,1
CIMT	218-17-1i	30	1	10,3
CIMT	218-19-1i	30	2	16,4
CIMT	299-14-1i	30	1	10,2
CIMT	299-14-2i	30	3	44,7
CIMT	299-15-1i	30	3	42,2
CIMT	299-16-1i	30	1	10,2
CIMT	299-17-1i	30	2	52,8
CIMT	300-16-1i	30	2	12
CIMT	300-20-1i	30	3	49,1
CIMT	300-22-1i	30	2	13,8
CIMT	300-24-1i	30	3	50,6
CIMT	300-24-2i	30	2	21,5
CIMT	309-15-1i	30	3	59
CIMT	309-15-2i	30	4	60,1
CIMT	309-16-1i	30	5	80,2
CIMT	309-16-2i	30	1	11,8
CIMT	309-17-1i	30	3	27,1
CIMT+G-CSF	213-12-1i	30	3	50,3
CIMT+G-CSF	213-14-1i	30	2	23,6
CIMT+G-CSF	213-17-1i	30	2	11,6
CIMT+G-CSF	213-18-1i	30	1	12,9
CIMT+G-CSF	213-8-1i	30	1	15,2
CIMT+G-CSF	214-14-1i	30	4	107,8

CIMT+G-CSF	214-14-2i	30	4	74,3
CIMT+G-CSF	214-17-1i	30	2	29
CIMT+G-CSF	214-18-1i	30	2	45,2
CIMT+G-CSF	214-19-1i	30	1	13,8
CIMT+G-CSF	217-10-1i	30	2	22,5
CIMT+G-CSF	217-16-1i	30	0	0
CIMT+G-CSF	217-17-1i	30	1	10,3
CIMT+G-CSF	217-6-1i	30	2	27,5
CIMT+G-CSF	217-8-1i	30	4	53,6
CIMT+G-CSF	304-17-1i	30	1	13,5
CIMT+G-CSF	304-19-1i	30	1	12,1
CIMT+G-CSF	304-19-2i	30	1	10,4
CIMT+G-CSF	304-20-1i	30	1	10,6
CIMT+G-CSF	304-21-1i	30	1	10,3
CIMT+G-CSF	304-21-2i	30	2	22
CIMT+G-CSF	304-22-1i	30	1	10,1
CIMT+G-CSF	305-17-1i	30	1	10,8
CIMT+G-CSF	305-17-2i	30	1	10,5
CIMT+G-CSF	305-18-1i	30	1	10,5
CIMT+G-CSF	305-19-1i	30	1	10,3
CIMT+G-CSF	305-19-2i	30	1	18
CIMT/G-CSF	306-17-1i	30	1	11,4
CIMT/G-CSF	306-17-2i	30	1	11
CIMT/G-CSF	306-18-1i	30	1	11
CIMT/G-CSF	306-18-2i	30	1	13,7
CIMT/G-CSF	306-18-3i	30	1	12,3
CIMT/G-CSF	311-16-1i	30	3	38,8
CIMT/G-CSF	311-17-1i	30	1	10,4
CIMT/G-CSF	311-17-2i	30	2	11,7
CIMT/G-CSF	311-18-1i	30	3	38,9
CIMT/G-CSF	311-19-1i	30	3	33,7
CIMT/G-CSF	313-14.1i	30	2	24,1
CIMT/G-CSF	313-15-1i	30	2	46,8
CIMT/G-CSF	313-17-1i	30	1	29,5
CIMT/G-CSF	313-19-1i	30	1	12,7
CIMT/G-CSF	313-19-2i	30	3	39,1
CIMT/G-CSF	314-16-1i	30	3	30,6
CIMT/G-CSF	314-16-2i	30	8	102,1
CIMT/G-CSF	314-17-1i	30	1	10,9
CIMT/G-CSF	314-17-2i	30	1	11,4
CIMT/G-CSF	314-18-1i	30	1	11,4
CIMT/G-CSF	315-11-1i	30	2	33,2
CIMT/G-CSF	315-11-2i	30	2	45,2
CIMT/G-CSF	315-13-1i	30	1	10,6
CIMT/G-CSF	315-13-2i	30	2	34,3
CIMT/G-CSF	315-14-1i	30	4	61,5
CIMT/G-CSF	315-14-2i	30	2	24
G-CSF	220-16-1i	30	5	67,1
G-CSF	220-17-1i	30	2	21,2
G-CSF	220-18-1i	30	1	11,2
G-CSF	220-19-1i	30	2	4,8
G-CSF	220-7-1i	30	3	32,3
G-CSF	221-13-1i	30	1	14,6
G-CSF	221-16-1i	30	1	10,4
G-CSF	221-19-1i	30	1	10,6
G-CSF	221-22-1i	30	1	11,3

G-CSF	221-6-1i	30	1	11,2
G-CSF	222-16-1i	30	2	25
G-CSF	222-17-1i	30	1	10,7
G-CSF	222-21-1i	30	2	16,4
G-CSF	222-23-1i	30	2	36,4
G-CSF	222-8-1i	30	1	10,3
G-CSF	301-18-1i	30	11	177,3
G-CSF	301-19-1i	30	1	10,2
G-CSF	301-20-1i	30	1	10,4
G-CSF	301-20-2i	30	2	48,1
G-CSF	301-20-3i	30	2	14,3
G-CSF	302-16-1i	30	1	11
G-CSF	302-16-2i	30	2	45,8
G-CSF	302-17-1i	30	1	11,1
G-CSF	302-17-2i	30	1	10,4
G-CSF	302-18-1i	30	2	25,1
G-CSF	303-15-1i	30	1	11,7
G-CSF	303-16-1i	30	3	37,2
G-CSF	303-16-2i	30	2	34,3
G-CSF	303-17-1i	30	2	30,2
G-CSF	303-17-2i	30	2	24,5

apical dendrite Sholl 40µm

Group	number	Radius(µm)	Intersections	Length(µm)
Control	215-11-1i	40	1	11,8
Control	215-15-1i	40	2	30,9
Control	215-16-1i	40	2	30,1
Control	215-18-1i	40	3	39,2
Control	215-9-1i	40	3	47,1
Control	216-12-1i	40	3	30,5
Control	216-18-1i	40	1	20,5
Control	216-19-1i	40	2	17,9
Control	216-22-1i	40	2	23,5
Control	216-6-1i	40	5	115
Control	216-8-1i	40	6	110,7
Control	219-10-1i	40	3	39,2
Control	219-14-1i	40	1	11,5
Control	219-15-1i	40	4	41,6
Control	219-17-1i	40	3	18,8
Control	219-21-1i	40	2	32,5
Control	297-19-1i	40	2	25,4
Control	297-19-2i	40	4	28,9
Control	297-20-1i	40	1	10
Control	297-22-1i	40	1	14,9
Control	297-22-2i	40	4	64,5
Control	298-15-1i	40	3	27,7
Control	298-15-2i	40	3	31,6
Control	298-16-1i	40	2	26,8
Control	298-17-1i	40	2	23,1
Control	298-18-1i	40	4	46,8
Control	308-17-1i	40	4	47,3
Control	308-18-1i	40	1	10,5
Control	308-18-2i	40	4	25,4
Control	308-21-1i	40	5	72,6

Control	308-21-2i	40	4	58,7
CIMT	211-10-1i	40	7	111,2
CIMT	211-13-1i	40	2	39,7
CIMT	211-13-2i	40	3	39,3
CIMT	211-14-1i	40	1	20,5
CIMT	211-14-2i	40	1	10,6
CIMT	211-15-1i	40	1	12,2
CIMT	211-15-2i	40	1	10,5
CIMT	212-13-1i	40	2	16,1
CIMT	212-13-2i	40	5	48
CIMT	212-14-1i	40	7	128
CIMT	212-14-2i	40	4	41,7
CIMT	212-15-1i	40	1	10,4
CIMT	218-11-1i	40	4	66,7
CIMT	218-14-1i	40	1	11,1
CIMT	218-16-1i	40	1	14,4
CIMT	218-17-1i	40	1	10,3
CIMT	218-19-1i	40	3	42,1
CIMT	299-14-1i	40	3	46,9
CIMT	299-14-2i	40	3	43,6
CIMT	299-15-1i	40	4	53,1
CIMT	299-16-1i	40	1	11,2
CIMT	299-17-1i	40	3	36,9
CIMT	300-16-1i	40	4	36,3
CIMT	300-20-1i	40	5	51,4
CIMT	300-22-1i	40	4	37,4
CIMT	300-24-1i	40	2	33,4
CIMT	300-24-2i	40	2	20,6
CIMT	309-15-1i	40	3	38,8
CIMT	309-15-2i	40	6	132,1
CIMT	309-16-1i	40	4	65,5
CIMT	309-16-2i	40	3	37,5
CIMT	309-17-1i	40	8	118,5
CIMT+G-CSF	213-12-1i	40	5	54,7
CIMT+G-CSF	213-14-1i	40	1	41,4
CIMT+G-CSF	213-17-1i	40	2	50,9
CIMT+G-CSF	213-18-1i	40	2	26,5
CIMT+G-CSF	213-8-1i	40	4	26
CIMT+G-CSF	214-14-1i	40	3	40,7
CIMT+G-CSF	214-14-2i	40	6	84,1
CIMT+G-CSF	214-17-1i	40	3	27,5
CIMT+G-CSF	214-18-1i	40	2	26,1
CIMT+G-CSF	214-19-1i	40	1	13,2
CIMT+G-CSF	217-10-1i	40	2	26,9
CIMT+G-CSF	217-16-1i	40	2	22,9
CIMT+G-CSF	217-17-1i	40	1	10,4
CIMT+G-CSF	217-6-1i	40	3	56
CIMT+G-CSF	217-8-1i	40	3	64
CIMT+G-CSF	304-17-1i	40	1	10,7
CIMT+G-CSF	304-19-1i	40	2	43,8
CIMT+G-CSF	304-19-2i	40	2	18,3
CIMT+G-CSF	304-20-1i	40	2	27,8
CIMT+G-CSF	304-21-1i	40	2	13,9
CIMT+G-CSF	304-21-2i	40	2	23,9
CIMT+G-CSF	304-22-1i	40	2	19,1
CIMT+G-CSF	305-17-1i	40	1	13,2

CIMT+G-CSF	305-17-2i	40	1	13,5
CIMT+G-CSF	305-18-1i	40	2	33,3
CIMT+G-CSF	305-19-1i	40	2	40,2
CIMT+G-CSF	305-19-2i	40	1	17,1
CIMT/G-CSF	306-17-1i	40	1	12,9
CIMT/G-CSF	306-17-2i	40	2	65,2
CIMT/G-CSF	306-18-1i	40	3	47,7
CIMT/G-CSF	306-18-2i	40	2	32,1
CIMT/G-CSF	306-18-3i	40	2	29,1
CIMT/G-CSF	311-16-1i	40	3	47,8
CIMT/G-CSF	311-17-1i	40	2	12,5
CIMT/G-CSF	311-17-2i	40	2	24,1
CIMT/G-CSF	311-18-1i	40	3	61
CIMT/G-CSF	311-19-1i	40	4	106,6
CIMT/G-CSF	313-14.1i	40	2	21,9
CIMT/G-CSF	313-15-1i	40	2	22,7
CIMT/G-CSF	313-17-1i	40	2	14,6
CIMT/G-CSF	313-19-1i	40	2	32
CIMT/G-CSF	313-19-2i	40	3	37,2
CIMT/G-CSF	314-16-1i	40	3	38,4
CIMT/G-CSF	314-16-2i	40	6	95,1
CIMT/G-CSF	314-17-1i	40	2	31,8
CIMT/G-CSF	314-17-2i	40	2	33,2
CIMT/G-CSF	314-18-1i	40	3	60
CIMT/G-CSF	315-11-1i	40	3	41,5
CIMT/G-CSF	315-11-2i	40	1	65,2
CIMT/G-CSF	315-13-1i	40	2	19,2
CIMT/G-CSF	315-13-2i	40	2	26,6
CIMT/G-CSF	315-14-1i	40	5	116,5
CIMT/G-CSF	315-14-2i	40	4	45,4
G-CSF	220-16-1i	40	5	71,7
G-CSF	220-17-1i	40	1	13
G-CSF	220-18-1i	40	1	11,2
G-CSF	220-19-1i	40	3	61,4
G-CSF	220-7-1i	40	2	40,5
G-CSF	221-13-1i	40	1	10,7
G-CSF	221-16-1i	40	3	36,4
G-CSF	221-19-1i	40	2	14,6
G-CSF	221-22-1i	40	2	23,9
G-CSF	221-6-1i	40	1	10
G-CSF	222-16-1i	40	2	25,7
G-CSF	222-17-1i	40	1	10,2
G-CSF	222-21-1i	40	2	29,6
G-CSF	222-23-1i	40	6	67,5
G-CSF	222-8-1i	40	1	10,4
G-CSF	301-18-1i	40	12	155,3
G-CSF	301-19-1i	40	2	21,5
G-CSF	301-20-1i	40	2	23,7
G-CSF	301-20-2i	40	2	35
G-CSF	301-20-3i	40	3	64,1
G-CSF	302-16-1i	40	1	10,2
G-CSF	302-16-2i	40	2	44,1
G-CSF	302-17-1i	40	1	10,4
G-CSF	302-17-2i	40	1	10,5
G-CSF	302-18-1i	40	2	47,8
G-CSF	303-15-1i	40	3	27,7

G-CSF	303-16-1i	40	3	33
G-CSF	303-16-2i	40	3	30,3
G-CSF	303-17-1i	40	4	38,4
G-CSF	303-17-2i	40	4	39,9

apical dendrite Sholl 50µm

Group	number	Radius(µm)	Intersections	Length(µm)
Control	215-11-1i	50	2	45,8
Control	215-15-1i	50	2	27,6
Control	215-16-1i	50	3	45,1
Control	215-18-1i	50	2	44,8
Control	215-9-1i	50	4	53,3
Control	216-12-1i	50	3	34,4
Control	216-18-1i	50	1	11,8
Control	216-19-1i	50	2	26,1
Control	216-22-1i	50	2	24,4
Control	216-6-1i	50	7	107,4
Control	216-8-1i	50	6	123,6
Control	219-10-1i	50	3	43,8
Control	219-14-1i	50	1	10
Control	219-15-1i	50	6	58,4
Control	219-17-1i	50	1	47,8
Control	219-21-1i	50	3	37,5
Control	297-19-1i	50	3	31,5
Control	297-19-2i	50	2	33,4
Control	297-20-1i	50	2	22,5
Control	297-22-1i	50	1	13,1
Control	297-22-2i	50	4	56,6
Control	298-15-1i	50	4	51,7
Control	298-15-2i	50	9	80,9
Control	298-16-1i	50	1	18,4
Control	298-17-1i	50	5	48,5
Control	298-18-1i	50	5	56,2
Control	308-17-1i	50	4	53,6
Control	308-18-1i	50	1	11
Control	308-18-2i	50	4	82,7
Control	308-21-1i	50	10	96,8
Control	308-21-2i	50	4	45,2
CIMT	211-10-1i	50	6	147,7
CIMT	211-13-1i	50	4	33,5
CIMT	211-13-2i	50	3	33,6
CIMT	211-14-1i	50	1	10,1
CIMT	211-14-2i	50	1	10,4
CIMT	211-15-1i	50	1	11,6
CIMT	211-15-2i	50	3	57,5
CIMT	212-13-1i	50	3	43,4
CIMT	212-13-2i	50	5	77
CIMT	212-14-1i	50	6	106,6
CIMT	212-14-2i	50	4	65,1
CIMT	212-15-1i	50	4	42,5
CIMT	218-11-1i	50	4	45,9
CIMT	218-14-1i	50	1	10,5
CIMT	218-16-1i	50	1	11,4
CIMT	218-17-1i	50	1	10,2

CIMT	218-19-1i	50	4	51,7
CIMT	299-14-1i	50	4	81,2
CIMT	299-14-2i	50	4	55,7
CIMT	299-15-1i	50	5	78,9
CIMT	299-16-1i	50	3	49,9
CIMT	299-17-1i	50	2	58,2
CIMT	300-16-1i	50	5	68,9
CIMT	300-20-1i	50	4	56,9
CIMT	300-22-1i	50	5	97,6
CIMT	300-24-1i	50	3	28,4
CIMT	300-24-2i	50	2	21,1
CIMT	309-15-1i	50	3	39,9
CIMT	309-15-2i	50	4	63,3
CIMT	309-16-1i	50	5	77,2
CIMT	309-16-2i	50	3	38,2
CIMT	309-17-1i	50	8	103
CIMT+G-CSF	213-12-1i	50	4	58,4
CIMT+G-CSF	213-14-1i	50	2	19,9
CIMT+G-CSF	213-17-1i	50	2	39,1
CIMT+G-CSF	213-18-1i	50	2	27,7
CIMT+G-CSF	213-8-1i	50	3	55,9
CIMT+G-CSF	214-14-1i	50	3	57,4
CIMT+G-CSF	214-14-2i	50	5	130
CIMT+G-CSF	214-17-1i	50	3	38,6
CIMT+G-CSF	214-18-1i	50	3	41,7
CIMT+G-CSF	214-19-1i	50	1	10,6
CIMT+G-CSF	217-10-1i	50	5	104,5
CIMT+G-CSF	217-16-1i	50	5	61
CIMT+G-CSF	217-17-1i	50	2	28,3
CIMT+G-CSF	217-6-1i	50	5	73,8
CIMT+G-CSF	217-8-1i	50	7	139,8
CIMT+G-CSF	304-17-1i	50	1	11,6
CIMT+G-CSF	304-19-1i	50	1	14,4
CIMT+G-CSF	304-19-2i	50	2	24,8
CIMT+G-CSF	304-20-1i	50	2	25,6
CIMT+G-CSF	304-21-1i	50	4	33,3
CIMT+G-CSF	304-21-2i	50	2	30,8
CIMT+G-CSF	304-22-1i	50	1	23,7
CIMT+G-CSF	305-17-1i	50	1	17,2
CIMT+G-CSF	305-17-2i	50	1	11,8
CIMT+G-CSF	305-18-1i	50	3	38,4
CIMT+G-CSF	305-19-1i	50	3	32,3
CIMT+G-CSF	305-19-2i	50	2	59,7
CIMT/G-CSF	306-17-1i	50	2	18,9
CIMT/G-CSF	306-17-2i	50	3	73,6
CIMT/G-CSF	306-18-1i	50	4	54,6
CIMT/G-CSF	306-18-2i	50	2	37,9
CIMT/G-CSF	306-18-3i	50	3	52,3
CIMT/G-CSF	311-16-1i	50	1	36,2
CIMT/G-CSF	311-17-1i	50	4	90,5
CIMT/G-CSF	311-17-2i	50	2	21,7
CIMT/G-CSF	311-18-1i	50	2	34,1
CIMT/G-CSF	311-19-1i	50	6	71,1
CIMT/G-CSF	313-14.1i	50	2	21,7
CIMT/G-CSF	313-15-1i	50	2	21,5
CIMT/G-CSF	313-17-1i	50	2	34,7

CIMT/G-CSF	313-19-1i	50	3	31,1
CIMT/G-CSF	313-19-2i	50	3	55,6
CIMT/G-CSF	314-16-1i	50	3	36,6
CIMT/G-CSF	314-16-2i	50	6	68
CIMT/G-CSF	314-17-1i	50	2	28,2
CIMT/G-CSF	314-17-2i	50	7	110,1
CIMT/G-CSF	314-18-1i	50	4	85,9
CIMT/G-CSF	315-11-1i	50	3	36,3
CIMT/G-CSF	315-11-2i	50	1	11
CIMT/G-CSF	315-13-1i	50	4	66,4
CIMT/G-CSF	315-13-2i	50	3	42
CIMT/G-CSF	315-14-1i	50	6	108,8
CIMT/G-CSF	315-14-2i	50	5	57,4
G-CSF	220-16-1i	50	4	74,4
G-CSF	220-17-1i	50	2	40
G-CSF	220-18-1i	50	5	83,1
G-CSF	220-19-1i	50	2	42,7
G-CSF	220-7-1i	50	2	22,1
G-CSF	221-13-1i	50	3	43,1
G-CSF	221-16-1i	50	3	47
G-CSF	221-19-1i	50	4	35,5
G-CSF	221-22-1i	50	2	48,3
G-CSF	221-6-1i	50	2	18,5
G-CSF	222-16-1i	50	4	57,4
G-CSF	222-17-1i	50	2	18,7
G-CSF	222-21-1i	50	2	29,6
G-CSF	222-23-1i	50	4	82,3
G-CSF	222-8-1i	50	2	20,8
G-CSF	301-18-1i	50	11	164,7
G-CSF	301-19-1i	50	2	29,7
G-CSF	301-20-1i	50	2	25
G-CSF	301-20-2i	50	2	28,6
G-CSF	301-20-3i	50	2	36,4
G-CSF	302-16-1i	50	1	10,3
G-CSF	302-16-2i	50	2	26,8
G-CSF	302-17-1i	50	3	37,3
G-CSF	302-17-2i	50	1	12,2
G-CSF	302-18-1i	50	1	26,3
G-CSF	303-15-1i	50	4	54,6
G-CSF	303-16-1i	50	3	58
G-CSF	303-16-2i	50	4	74,3
G-CSF	303-17-1i	50	4	62,2
G-CSF	303-17-2i	50	5	51,7

apical dendrite Sholl 60µm

Group	number	Radius(µm)	Intersections	Length(µm)
Control	215-11-1i	60	3	64,9
Control	215-15-1i	60	2	24,2
Control	215-16-1i	60	4	45,8
Control	215-18-1i	60	3	33
Control	215-9-1i	60	4	53,6
Control	216-12-1i	60	3	36,1
Control	216-18-1i	60	1	10,4
Control	216-19-1i	60	3	39,4

Control	216-22-1i	60	2	13,6
Control	216-6-1i	60	5	86,2
Control	216-8-1i	60	3	66,4
Control	219-10-1i	60	4	97,7
Control	219-14-1i	60	1	11,3
Control	219-15-1i	60	4	77,4
Control	219-17-1i	60	1	10,9
Control	219-21-1i	60	5	84,5
Control	297-19-1i	60	3	55,8
Control	297-19-2i	60	3	54,1
Control	297-20-1i	60	2	25,9
Control	297-22-1i	60	2	23,7
Control	297-22-2i	60	6	63
Control	298-15-1i	60	4	68,6
Control	298-15-2i	60	7	96,7
Control	298-16-1i	60	1	11,2
Control	298-17-1i	60	3	42,8
Control	298-18-1i	60	5	56,4
Control	308-17-1i	60	4	47,9
Control	308-18-1i	60	3	74,6
Control	308-18-2i	60	4	93,7
Control	308-21-1i	60	6	120,4
Control	308-21-2i	60	5	71,2
CIMT	211-10-1i	60	6	76
CIMT	211-13-1i	60	4	72,7
CIMT	211-13-2i	60	4	55,7
CIMT	211-14-1i	60	2	15,9
CIMT	211-14-2i	60	1	11,5
CIMT	211-15-1i	60	1	11,5
CIMT	211-15-2i	60	2	38,8
CIMT	212-13-1i	60	3	39,8
CIMT	212-13-2i	60	6	85,1
CIMT	212-14-1i	60	6	75,5
CIMT	212-14-2i	60	3	40
CIMT	212-15-1i	60	2	34,1
CIMT	218-11-1i	60	5	44,2
CIMT	218-14-1i	60	1	16,4
CIMT	218-16-1i	60	1	11,9
CIMT	218-17-1i	60	2	44,9
CIMT	218-19-1i	60	3	54,8
CIMT	299-14-1i	60	4	56,1
CIMT	299-14-2i	60	3	78,3
CIMT	299-15-1i	60	5	64,2
CIMT	299-16-1i	60	3	43,4
CIMT	299-17-1i	60	2	23,1
CIMT	300-16-1i	60	4	59,7
CIMT	300-20-1i	60	4	48,5
CIMT	300-22-1i	60	4	61,6
CIMT	300-24-1i	60	3	34,5
CIMT	300-24-2i	60	5	43
CIMT	309-15-1i	60	4	62,8
CIMT	309-15-2i	60	3	40,5
CIMT	309-16-1i	60	7	127,2
CIMT	309-16-2i	60	2	36,3
CIMT	309-17-1i	60	5	106,2
CIMT+G-CSF	213-12-1i	60	5	96,5

CIMT+G-CSF	213-14-1i	60	1	37,8
CIMT+G-CSF	213-17-1i	60	6	78
CIMT+G-CSF	213-18-1i	60	3	54,6
CIMT+G-CSF	213-8-1i	60	5	92
CIMT+G-CSF	214-14-1i	60	1	37,5
CIMT+G-CSF	214-14-2i	60	3	81,8
CIMT+G-CSF	214-17-1i	60	3	33,4
CIMT+G-CSF	214-18-1i	60	4	45
CIMT+G-CSF	214-19-1i	60	1	10,7
CIMT+G-CSF	217-10-1i	60	5	69,9
CIMT+G-CSF	217-16-1i	60	3	43,5
CIMT+G-CSF	217-17-1i	60	3	39,8
CIMT+G-CSF	217-6-1i	60	5	88,6
CIMT+G-CSF	217-8-1i	60	6	84,5
CIMT+G-CSF	304-17-1i	60	0	8,8
CIMT+G-CSF	304-19-1i	60	2	26,1
CIMT+G-CSF	304-19-2i	60	3	28,1
CIMT+G-CSF	304-20-1i	60	2	23,8
CIMT+G-CSF	304-21-1i	60	4	54,9
CIMT+G-CSF	304-21-2i	60	1	18,3
CIMT+G-CSF	304-22-1i	60	1	10,4
CIMT+G-CSF	305-17-1i	60	1	10,9
CIMT+G-CSF	305-17-2i	60	1	10,4
CIMT+G-CSF	305-18-1i	60	3	27,3
CIMT+G-CSF	305-19-1i	60	3	66,2
CIMT+G-CSF	305-19-2i	60	4	54,7
CIMT/G-CSF	306-17-1i	60	2	59,5
CIMT/G-CSF	306-17-2i	60	3	55,1
CIMT/G-CSF	306-18-1i	60	4	52,5
CIMT/G-CSF	306-18-2i	60	2	26,7
CIMT/G-CSF	306-18-3i	60	3	43,4
CIMT/G-CSF	311-16-1i	60	5	50,9
CIMT/G-CSF	311-17-1i	60	4	148,4
CIMT/G-CSF	311-17-2i	60	3	31,2
CIMT/G-CSF	311-18-1i	60	1	18,7
CIMT/G-CSF	311-19-1i	60	6	74,8
CIMT/G-CSF	313-14.1i	60	2	22,3
CIMT/G-CSF	313-15-1i	60	2	40,7
CIMT/G-CSF	313-17-1i	60	3	46,2
CIMT/G-CSF	313-19-1i	60	3	38,7
CIMT/G-CSF	313-19-2i	60	3	36,6
CIMT/G-CSF	314-16-1i	60	4	38,8
CIMT/G-CSF	314-16-2i	60	8	109,5
CIMT/G-CSF	314-17-1i	60	1	46,9
CIMT/G-CSF	314-17-2i	60	4	78,8
CIMT/G-CSF	314-18-1i	60	5	74,6
CIMT/G-CSF	315-11-1i	60	2	28,6
CIMT/G-CSF	315-11-2i	60	1	11,3
CIMT/G-CSF	315-13-1i	60	7	75,5
CIMT/G-CSF	315-13-2i	60	2	32,1
CIMT/G-CSF	315-14-1i	60	5	112,1
CIMT/G-CSF	315-14-2i	60	6	84,9
G-CSF	220-16-1i	60	6	91,9
G-CSF	220-17-1i	60	4	50,7
G-CSF	220-18-1i	60	4	76,3
G-CSF	220-19-1i	60	2	24,7

G-CSF	220-7-1i	60	2	25,3
G-CSF	221-13-1i	60	3	49,9
G-CSF	221-16-1i	60	3	69,4
G-CSF	221-19-1i	60	5	69,5
G-CSF	221-22-1i	60	4	83,2
G-CSF	221-6-1i	60	2	23,8
G-CSF	222-16-1i	60	4	65,3
G-CSF	222-17-1i	60	2	29,8
G-CSF	222-21-1i	60	2	25,8
G-CSF	222-23-1i	60	3	50,8
G-CSF	222-8-1i	60	2	23,5
G-CSF	301-18-1i	60	9	138,2
G-CSF	301-19-1i	60	2	19,2
G-CSF	301-20-1i	60	2	26,4
G-CSF	301-20-2i	60	1	27,8
G-CSF	301-20-3i	60	2	21
G-CSF	302-16-1i	60	1	10,3
G-CSF	302-16-2i	60	3	27,3
G-CSF	302-17-1i	60	3	43,5
G-CSF	302-17-2i	60	1	13,5
G-CSF	302-18-1i	60	1	14,2
G-CSF	303-15-1i	60	4	54
G-CSF	303-16-1i	60	6	61,1
G-CSF	303-16-2i	60	5	72,9
G-CSF	303-17-1i	60	3	39,4
G-CSF	303-17-2i	60	5	96,5

apical dendrite Sholl 70µm

Group	number	Radius(µm)	Intersections	Length(µm)
Control	215-11-1i	70	2	55,4
Control	215-15-1i	70	2	21,5
Control	215-16-1i	70	4	67,4
Control	215-18-1i	70	4	36,4
Control	215-9-1i	70	3	48,8
Control	216-12-1i	70	4	40,8
Control	216-18-1i	70	1	10,3
Control	216-19-1i	70	3	34,9
Control	216-22-1i	70	3	73,5
Control	216-6-1i	70	4	111,2
Control	216-8-1i	70	4	80,1
Control	219-10-1i	70	5	73,3
Control	219-14-1i	70	1	66,6
Control	219-15-1i	70	3	40,6
Control	219-17-1i	70	1	20,3
Control	219-21-1i	70	7	114,7
Control	297-19-1i	70	3	34,6
Control	297-19-2i	70	4	35,3
Control	297-20-1i	70	2	27,9
Control	297-22-1i	70	1	27,5
Control	297-22-2i	70	10	170
Control	298-15-1i	70	5	77,2
Control	298-15-2i	70	7	84,5
Control	298-16-1i	70	2	14
Control	298-17-1i	70	4	54,8

Control	298-18-1i	70	6	61,3
Control	308-17-1i	70	4	55,9
Control	308-18-1i	70	3	40,8
Control	308-18-2i	70	5	82,4
Control	308-21-1i	70	6	93
Control	308-21-2i	70	5	119,7
CIMT	211-10-1i	70	5	79,7
CIMT	211-13-1i	70	2	85,8
CIMT	211-13-2i	70	4	46,8
CIMT	211-14-1i	70	1	23,2
CIMT	211-14-2i	70	1	10,9
CIMT	211-15-1i	70	1	10,4
CIMT	211-15-2i	70	2	24,3
CIMT	212-13-1i	70	4	41,9
CIMT	212-13-2i	70	5	133,4
CIMT	212-14-1i	70	3	55,6
CIMT	212-14-2i	70	2	29,9
CIMT	212-15-1i	70	2	22,4
CIMT	218-11-1i	70	3	59,4
CIMT	218-14-1i	70	2	28,1
CIMT	218-16-1i	70	1	10,6
CIMT	218-17-1i	70	5	107,8
CIMT	218-19-1i	70	3	45,9
CIMT	299-14-1i	70	4	44
CIMT	299-14-2i	70	2	31,3
CIMT	299-15-1i	70	6	71,5
CIMT	299-16-1i	70	5	67,8
CIMT	299-17-1i	70	2	37,1
CIMT	300-16-1i	70	3	47
CIMT	300-20-1i	70	4	43,7
CIMT	300-22-1i	70	3	40,6
CIMT	300-24-1i	70	3	35,6
CIMT	300-24-2i	70	5	73
CIMT	309-15-1i	70	4	55,5
CIMT	309-15-2i	70	3	44,9
CIMT	309-16-1i	70	3	56,9
CIMT	309-16-2i	70	4	40,6
CIMT	309-17-1i	70	5	72,1
CIMT+G-CSF	213-12-1i	70	4	50,9
CIMT+G-CSF	213-14-1i	70	1	10,4
CIMT+G-CSF	213-17-1i	70	7	115,5
CIMT+G-CSF	213-18-1i	70	4	82,7
CIMT+G-CSF	213-8-1i	70	3	77,8
CIMT+G-CSF	214-14-1i	70	1	10,5
CIMT+G-CSF	214-14-2i	70	1	18,1
CIMT+G-CSF	214-17-1i	70	4	48,7
CIMT+G-CSF	214-18-1i	70	4	48,4
CIMT+G-CSF	214-19-1i	70	2	40,2
CIMT+G-CSF	217-10-1i	70	4	53,8
CIMT+G-CSF	217-16-1i	70	3	48
CIMT+G-CSF	217-17-1i	70	4	40,5
CIMT+G-CSF	217-6-1i	70	5	77,2
CIMT+G-CSF	217-8-1i	70	5	88,6
CIMT+G-CSF	304-19-1i	70	2	27,4
CIMT+G-CSF	304-19-2i	70	3	36,1
CIMT+G-CSF	304-20-1i	70	1	19

CIMT+G-CSF	304-21-1i	70	4	47
CIMT+G-CSF	304-21-2i	70	1	10,5
CIMT+G-CSF	304-22-1i	70	1	10,5
CIMT+G-CSF	305-17-1i	70	3	42,3
CIMT+G-CSF	305-17-2i	70	1	10,2
CIMT+G-CSF	305-18-1i	70	1	51,3
CIMT+G-CSF	305-19-1i	70	3	45,1
CIMT+G-CSF	305-19-2i	70	5	85,7
CIMT/G-CSF	306-17-1i	70	2	25,3
CIMT/G-CSF	306-17-2i	70	3	62,2
CIMT/G-CSF	306-18-1i	70	3	53,3
CIMT/G-CSF	306-18-2i	70	2	26,1
CIMT/G-CSF	306-18-3i	70	2	30,1
CIMT/G-CSF	311-16-1i	70	4	90,6
CIMT/G-CSF	311-17-1i	70	5	68,3
CIMT/G-CSF	311-17-2i	70	4	69,3
CIMT/G-CSF	311-18-1i	70	1	10,2
CIMT/G-CSF	311-19-1i	70	9	110,1
CIMT/G-CSF	313-14.1i	70	2	22,1
CIMT/G-CSF	313-15-1i	70	2	23,5
CIMT/G-CSF	313-17-1i	70	2	44,4
CIMT/G-CSF	313-19-1i	70	3	36,5
CIMT/G-CSF	313-19-2i	70	3	61,2
CIMT/G-CSF	314-16-1i	70	4	50,5
CIMT/G-CSF	314-16-2i	70	7	112,2
CIMT/G-CSF	314-17-1i	70	1	12
CIMT/G-CSF	314-17-2i	70	6	144,2
CIMT/G-CSF	314-18-1i	70	3	73,2
CIMT/G-CSF	315-11-1i	70	3	33
CIMT/G-CSF	315-11-2i	70	1	13
CIMT/G-CSF	315-13-1i	70	4	109,5
CIMT/G-CSF	315-13-2i	70	2	24,4
CIMT/G-CSF	315-14-1i	70	5	84,4
CIMT/G-CSF	315-14-2i	70	4	69,4
G-CSF	220-16-1i	70	5	69,6
G-CSF	220-17-1i	70	3	45,2
G-CSF	220-18-1i	70	7	68
G-CSF	220-19-1i	70	2	27
G-CSF	220-7-1i	70	2	22
G-CSF	221-13-1i	70	4	53,1
G-CSF	221-16-1i	70	4	69,7
G-CSF	221-19-1i	70	9	91,4
G-CSF	221-22-1i	70	2	34,3
G-CSF	221-6-1i	70	2	23,3
G-CSF	222-16-1i	70	5	55,7
G-CSF	222-17-1i	70	2	51,9
G-CSF	222-21-1i	70	2	22,5
G-CSF	222-23-1i	70	4	55,8
G-CSF	222-8-1i	70	2	25,4
G-CSF	301-18-1i	70	8	106,7
G-CSF	301-19-1i	70	3	57,7
G-CSF	301-20-1i	70	2	32,9
G-CSF	301-20-2i	70	1	10,7
G-CSF	301-20-3i	70	3	41,4
G-CSF	302-16-1i	70	1	11,5
G-CSF	302-16-2i	70	2	28,2

G-CSF	302-17-1i	70	1	27
G-CSF	302-17-2i	70	1	11,1
G-CSF	302-18-1i	70	1	10,6
G-CSF	303-15-1i	70	4	52,4
G-CSF	303-16-1i	70	5	93,5
G-CSF	303-16-2i	70	4	101
G-CSF	303-17-1i	70	3	36,8
G-CSF	303-17-2i	70	4	63,9

apical dendrite Sholl 80µm

Group	number	Radius(µm)	Intersections	Length(µm)
Control	215-11-1i	80	2	28,6
Control	215-15-1i	80	2	21
Control	215-16-1i	80	3	52,9
Control	215-18-1i	80	4	54,6
Control	215-9-1i	80	2	34
Control	216-12-1i	80	3	43,7
Control	216-18-1i	80	1	16,7
Control	216-19-1i	80	2	33
Control	216-22-1i	80	2	31,8
Control	216-6-1i	80	3	45,4
Control	216-8-1i	80	6	105,8
Control	219-10-1i	80	4	72,1
Control	219-14-1i	80	1	10,5
Control	219-15-1i	80	2	34,7
Control	219-17-1i	80	3	14,2
Control	219-21-1i	80	7	126,8
Control	297-19-1i	80	3	36,6
Control	297-19-2i	80	2	68,4
Control	297-20-1i	80	3	35,1
Control	297-22-1i	80	2	26,7
Control	297-22-2i	80	11	143,5
Control	298-15-1i	80	3	58,7
Control	298-15-2i	80	8	98,2
Control	298-16-1i	80	4	49,8
Control	298-17-1i	80	6	56,7
Control	298-18-1i	80	5	57,9
Control	308-17-1i	80	5	80,1
Control	308-18-1i	80	3	42,7
Control	308-18-2i	80	5	77,4
Control	308-21-1i	80	5	70
Control	308-21-2i	80	4	74,9
CIMT	211-10-1i	80	5	58,2
CIMT	211-13-1i	80	2	35,3
CIMT	211-13-2i	80	2	31,7
CIMT	211-14-1i	80	1	11
CIMT	211-14-2i	80	1	12,7
CIMT	211-15-1i	80	1	11
CIMT	211-15-2i	80	4	76,3
CIMT	212-13-1i	80	4	81,3
CIMT	212-13-2i	80	4	70,5
CIMT	212-14-1i	80	4	52,4
CIMT	212-14-2i	80	3	38,5
CIMT	212-15-1i	80	1	52,3

CIMT	218-11-1i	80	2	31,4
CIMT	218-14-1i	80	4	93,6
CIMT	218-16-1i	80	1	10,4
CIMT	218-17-1i	80	5	74,3
CIMT	218-19-1i	80	2	31,9
CIMT	299-14-1i	80	4	54,1
CIMT	299-14-2i	80	3	55,5
CIMT	299-15-1i	80	6	72,7
CIMT	299-16-1i	80	5	75,7
CIMT	299-17-1i	80	2	25,7
CIMT	300-16-1i	80	2	23,4
CIMT	300-20-1i	80	2	25,6
CIMT	300-22-1i	80	3	36,5
CIMT	300-24-1i	80	4	88,2
CIMT	300-24-2i	80	5	78,8
CIMT	309-15-1i	80	5	67,8
CIMT	309-15-2i	80	2	49,5
CIMT	309-16-1i	80	5	110,4
CIMT	309-16-2i	80	5	92,7
CIMT	309-17-1i	80	5	61,5
CIMT+G-CSF	213-12-1i	80	4	53,7
CIMT+G-CSF	213-14-1i	80	2	12,7
CIMT+G-CSF	213-17-1i	80	5	92,7
CIMT+G-CSF	213-18-1i	80	4	65,4
CIMT+G-CSF	213-8-1i	80	6	94,9
CIMT+G-CSF	214-14-1i	80	2	27,6
CIMT+G-CSF	214-14-2i	80	2	52,3
CIMT+G-CSF	214-17-1i	80	3	36,6
CIMT+G-CSF	214-18-1i	80	4	55,3
CIMT+G-CSF	214-19-1i	80	2	40
CIMT+G-CSF	217-10-1i	80	6	96,6
CIMT+G-CSF	217-16-1i	80	3	63,6
CIMT+G-CSF	217-17-1i	80	4	51,9
CIMT+G-CSF	217-6-1i	80	6	89,3
CIMT+G-CSF	217-8-1i	80	7	103,1
CIMT+G-CSF	304-19-1i	80	2	44,4
CIMT+G-CSF	304-19-2i	80	3	34,5
CIMT+G-CSF	304-20-1i	80	0	1,3
CIMT+G-CSF	304-21-1i	80	4	70,2
CIMT+G-CSF	304-21-2i	80	0	0,2
CIMT+G-CSF	304-22-1i	80	1	15,1
CIMT+G-CSF	305-17-1i	80	2	59,5
CIMT+G-CSF	305-17-2i	80	4	62,6
CIMT+G-CSF	305-18-1i	80	1	10,5
CIMT+G-CSF	305-19-1i	80	3	54,3
CIMT+G-CSF	305-19-2i	80	4	73,6
CIMT/G-CSF	306-17-1i	80	3	43,8
CIMT/G-CSF	306-17-2i	80	2	79,9
CIMT/G-CSF	306-18-1i	80	5	87,7
CIMT/G-CSF	306-18-2i	80	2	27
CIMT/G-CSF	306-18-3i	80	1	11,8
CIMT/G-CSF	311-16-1i	80	3	74,3
CIMT/G-CSF	311-17-1i	80	4	77,4
CIMT/G-CSF	311-17-2i	80	6	90,2
CIMT/G-CSF	311-18-1i	80	1	10,4
CIMT/G-CSF	311-19-1i	80	9	151,8

CIMT/G-CSF	313-14.1i	80	2	23,4
CIMT/G-CSF	313-15-1i	80	1	21,1
CIMT/G-CSF	313-17-1i	80	2	27,8
CIMT/G-CSF	313-19-1i	80	2	27,6
CIMT/G-CSF	313-19-2i	80	2	44,4
CIMT/G-CSF	314-16-1i	80	2	46,5
CIMT/G-CSF	314-16-2i	80	5	84,2
CIMT/G-CSF	314-17-1i	80	1	13,8
CIMT/G-CSF	314-17-2i	80	4	64,4
CIMT/G-CSF	314-18-1i	80	2	25,3
CIMT/G-CSF	315-11-1i	80	3	46,9
CIMT/G-CSF	315-11-2i	80	1	12,4
CIMT/G-CSF	315-13-1i	80	4	53
CIMT/G-CSF	315-13-2i	80	1	13,7
CIMT/G-CSF	315-14-1i	80	4	63,1
CIMT/G-CSF	315-14-2i	80	5	64,9
G-CSF	220-16-1i	80	2	37,2
G-CSF	220-17-1i	80	3	30,9
G-CSF	220-18-1i	80	6	137,7
G-CSF	220-19-1i	80	3	38
G-CSF	220-7-1i	80	2	22,5
G-CSF	221-13-1i	80	4	57
G-CSF	221-16-1i	80	11	121,2
G-CSF	221-19-1i	80	8	108,4
G-CSF	221-22-1i	80	3	38,6
G-CSF	221-6-1i	80	3	34,1
G-CSF	222-16-1i	80	6	65,1
G-CSF	222-17-1i	80	2	26,6
G-CSF	222-21-1i	80	7	108,7
G-CSF	222-23-1i	80	4	53,1
G-CSF	222-8-1i	80	4	46,3
G-CSF	301-18-1i	80	7	163,1
G-CSF	301-19-1i	80	3	44,1
G-CSF	301-20-1i	80	2	26,2
G-CSF	301-20-2i	80	1	10,1
G-CSF	301-20-3i	80	3	40
G-CSF	302-16-1i	80	1	12
G-CSF	302-16-2i	80	2	21,4
G-CSF	302-17-1i	80	1	10,4
G-CSF	302-17-2i	80	1	12,6
G-CSF	302-18-1i	80	1	13,3
G-CSF	303-15-1i	80	4	47,8
G-CSF	303-16-1i	80	6	73,2
G-CSF	303-16-2i	80	3	45,6
G-CSF	303-17-1i	80	2	26,9
G-CSF	303-17-2i	80	4	61,9

apical dendrite Sholl 90µm

Group	number	Radius(µm)	Intersections	Length(µm)
Control	215-11-1i	90	3	69,9
Control	215-15-1i	90	2	24,9
Control	215-16-1i	90	3	34,1
Control	215-18-1i	90	5	61,1
Control	215-9-1i	90	4	52,3

Control	216-12-1i	90	2	22,5
Control	216-18-1i	90	2	28,3
Control	216-19-1i	90	1	24,2
Control	216-22-1i	90	2	21,7
Control	216-6-1i	90	3	33
Control	216-8-1i	90	5	88,2
Control	219-10-1i	90	1	20,5
Control	219-14-1i	90	5	89
Control	219-15-1i	90	3	26,2
Control	219-17-1i	90	1	19,9
Control	219-21-1i	90	2	27,8
Control	297-19-1i	90	3	37,7
Control	297-19-2i	90	3	56,9
Control	297-20-1i	90	3	70,1
Control	297-22-1i	90	4	65,4
Control	297-22-2i	90	2	28,8
Control	298-15-1i	90	9	179,1
Control	298-15-2i	90	3	44,6
Control	298-16-1i	90	6	101
Control	298-17-1i	90	3	100,5
Control	298-18-1i	90	8	107,3
Control	308-17-1i	90	1	11,3
Control	308-18-1i	90	5	70,9
Control	308-18-2i	90	4	59,3
Control	308-21-1i	90	8	74,8
Control	308-21-2i	90	3	70
CIMT	211-10-1i	90	3	79,8
CIMT	211-13-1i	90	3	79,8
CIMT	211-13-2i	90	3	33,4
CIMT	211-14-1i	90	1	12,7
CIMT	211-14-2i	90	1	10,2
CIMT	211-15-1i	90	3	22,9
CIMT	211-15-2i	90	1	12,8
CIMT	212-13-1i	90	2	50,7
CIMT	212-13-2i	90	3	57
CIMT	212-14-1i	90	3	59,4
CIMT	212-14-2i	90	3	36,8
CIMT	212-15-1i	90	3	35,7
CIMT	218-11-1i	90	5	102,4
CIMT	218-14-1i	90	2	32,2
CIMT	218-16-1i	90	5	65,3
CIMT	218-17-1i	90	1	12,6
CIMT	218-19-1i	90	4	53,7
CIMT	299-14-1i	90	5	59,9
CIMT	299-14-2i	90	3	37,2
CIMT	299-15-1i	90	1	14,5
CIMT	299-16-1i	90	4	56,2
CIMT	299-17-1i	90	4	65,3
CIMT	300-16-1i	90	2	31,3
CIMT	300-20-1i	90	1	34
CIMT	300-22-1i	90	5	60,1
CIMT	300-24-1i	90	3	31,2
CIMT	300-24-2i	90	6	73,4
CIMT	309-15-1i	90	3	46,7
CIMT	309-15-2i	90	5	61,5
CIMT	309-16-1i	90	4	60,3

CIMT	309-16-2i	90	3	67,5
CIMT	309-17-1i	90	5	75,6
CIMT+G-CSF	213-12-1i	90	2	20,2
CIMT+G-CSF	213-14-1i	90	3	53,3
CIMT+G-CSF	213-17-1i	90	2	22,4
CIMT+G-CSF	213-18-1i	90	4	106,7
CIMT+G-CSF	213-8-1i	90	2	24,6
CIMT+G-CSF	214-14-1i	90	2	69,4
CIMT+G-CSF	214-14-2i	90	2	23,2
CIMT+G-CSF	214-17-1i	90	3	25
CIMT+G-CSF	214-18-1i	90	3	33,6
CIMT+G-CSF	214-19-1i	90	4	49,1
CIMT+G-CSF	217-10-1i	90	6	138,2
CIMT+G-CSF	217-16-1i	90	4	111,1
CIMT+G-CSF	217-17-1i	90	4	59,6
CIMT+G-CSF	217-6-1i	90	7	111,9
CIMT+G-CSF	217-8-1i	90	7	77,4
CIMT+G-CSF	304-19-1i	90	3	39,4
CIMT+G-CSF	304-19-2i	90	2	25,9
CIMT+G-CSF	304-21-1i	90	3	40,7
CIMT+G-CSF	304-22-1i	90	2	77,3
CIMT+G-CSF	305-17-1i	90	1	11,2
CIMT+G-CSF	305-17-2i	90	2	24,4
CIMT+G-CSF	305-18-1i	90	3	87,5
CIMT+G-CSF	305-19-1i	90	1	10,2
CIMT+G-CSF	305-19-2i	90	2	23,7
CIMT/G-CSF	306-17-1i	90	4	52,3
CIMT/G-CSF	306-17-2i	90	2	33
CIMT/G-CSF	306-18-1i	90	1	23,1
CIMT/G-CSF	306-18-2i	90	5	66,2
CIMT/G-CSF	306-18-3i	90	1	20,7
CIMT/G-CSF	311-16-1i	90	5	66,4
CIMT/G-CSF	311-17-1i	90	2	33,2
CIMT/G-CSF	311-17-2i	90	6	115,3
CIMT/G-CSF	311-18-1i	90	6	62,3
CIMT/G-CSF	311-19-1i	90	2	19,3
CIMT/G-CSF	313-14.1i	90	9	129
CIMT/G-CSF	313-15-1i	90	2	27,4
CIMT/G-CSF	313-17-1i	90	1	10,7
CIMT/G-CSF	313-19-1i	90	2	25,3
CIMT/G-CSF	313-19-2i	90	1	21,7
CIMT/G-CSF	314-16-1i	90	3	26,3
CIMT/G-CSF	314-16-2i	90	2	23
CIMT/G-CSF	314-17-1i	90	5	76
CIMT/G-CSF	314-17-2i	90	1	10,7
CIMT/G-CSF	314-18-1i	90	2	23
CIMT/G-CSF	315-11-1i	90	2	26,3
CIMT/G-CSF	315-11-2i	90	1	27,3
CIMT/G-CSF	315-13-1i	90	1	14,1
CIMT/G-CSF	315-13-2i	90	3	39,9
CIMT/G-CSF	315-14-1i	90	0	2,4
CIMT/G-CSF	315-14-2i	90	2	26,9
G-CSF	220-16-1i	90	4	65,6
G-CSF	220-17-1i	90	2	24,7
G-CSF	220-18-1i	90	3	30,5
G-CSF	220-19-1i	90	5	69,8

G-CSF	220-7-1i	90	3	43
G-CSF	221-13-1i	90	3	48,8
G-CSF	221-16-1i	90	3	47,2
G-CSF	221-19-1i	90	5	247,3
G-CSF	221-22-1i	90	9	138,6
G-CSF	221-6-1i	90	2	57,4
G-CSF	222-16-1i	90	2	35,7
G-CSF	222-17-1i	90	7	132,3
G-CSF	222-21-1i	90	3	24,4
G-CSF	222-23-1i	90	5	87,3
G-CSF	222-8-1i	90	4	61,8
G-CSF	301-18-1i	90	5	62,1
G-CSF	301-19-1i	90	9	160,9
G-CSF	301-20-1i	90	3	56,6
G-CSF	301-20-2i	90	1	17
G-CSF	301-20-3i	90	1	10,2
G-CSF	302-16-1i	90	3	37,7
G-CSF	302-16-2i	90	1	10,6
G-CSF	302-17-1i	90	2	24,7
G-CSF	302-17-2i	90	1	12,3
G-CSF	302-18-1i	90	1	13,9
G-CSF	303-15-1i	90	1	12
G-CSF	303-16-1i	90	4	50,5
G-CSF	303-16-2i	90	6	86,9
G-CSF	303-17-1i	90	1	18,8
G-CSF	303-17-2i	90	1	12

apical dendrite Sholl 100µm

Group	number	Radius(µm)	Intersections	Length(µm)
Control	215-11-1i	100	3	44,9
Control	215-15-1i	100	1	31
Control	215-16-1i	100	3	82,1
Control	215-18-1i	100	4	50
Control	215-9-1i	100	2	22,6
Control	216-12-1i	100	2	21,1
Control	216-18-1i	100	1	10,6
Control	216-19-1i	100	2	25,5
Control	216-22-1i	100	2	57,4
Control	216-6-1i	100	5	78,5
Control	216-8-1i	100	6	125,2
Control	219-10-1i	100	2	63
Control	219-14-1i	100	4	50
Control	219-15-1i	100	1	10,4
Control	219-17-1i	100	2	47,5
Control	219-21-1i	100	2	41,8
Control	297-19-1i	100	7	84,4
Control	297-19-2i	100	5	66,9
Control	297-20-1i	100	4	60
Control	297-22-1i	100	3	38
Control	297-22-2i	100	4	99,7
Control	298-15-1i	100	3	40,8
Control	298-15-2i	100	8	91,4
Control	298-16-1i	100	4	55,6
Control	298-17-1i	100	8	114,1

Control	298-18-1i	100	4	54,5
Control	308-17-1i	100	3	52
Control	308-18-1i	100	2	57,7
Control	308-18-2i	100	6	101,4
Control	308-21-1i	100	2	24,7
Control	308-21-2i	100	4	63
CIMT	211-10-1i	100	4	84,3
CIMT	211-13-1i	100	3	34,8
CIMT	211-13-2i	100	0	15,2
CIMT	211-14-1i	100	1	10,6
CIMT	211-14-2i	100	3	49,1
CIMT	211-15-1i	100	1	28,5
CIMT	211-15-2i	100	2	59,7
CIMT	212-13-1i	100	4	93
CIMT	212-13-2i	100	3	53,8
CIMT	212-14-1i	100	3	36,9
CIMT	212-14-2i	100	2	48
CIMT	212-15-1i	100	2	30,6
CIMT	218-11-1i	100	2	28,3
CIMT	218-14-1i	100	3	55,1
CIMT	218-16-1i	100	1	10,6
CIMT	218-17-1i	100	3	41,1
CIMT	218-19-1i	100	1	10,3
CIMT	299-14-1i	100	2	23,7
CIMT	299-14-2i	100	1	10,9
CIMT	299-15-1i	100	5	46,5
CIMT	299-16-1i	100	2	43
CIMT	299-17-1i	100	2	44,1
CIMT	300-16-1i	100	2	47,2
CIMT	300-20-1i	100	4	63,2
CIMT	300-22-1i	100	2	26,3
CIMT	300-24-1i	100	7	106,6
CIMT	300-24-2i	100	4	50,3
CIMT	309-15-1i	100	5	60,6
CIMT	309-15-2i	100	4	89,1
CIMT	309-16-1i	100	2	26,5
CIMT	309-16-2i	100	5	56,4
CIMT	309-17-1i	100	5	67,8
CIMT+G-CSF	213-12-1i	100	1	52,4
CIMT+G-CSF	213-14-1i	100	2	47,8
CIMT+G-CSF	213-17-1i	100	2	40,6
CIMT+G-CSF	213-18-1i	100	4	33,6
CIMT+G-CSF	213-8-1i	100	2	15,7
CIMT+G-CSF	214-14-1i	100	1	27,5
CIMT+G-CSF	214-14-2i	100	1	32,4
CIMT+G-CSF	214-17-1i	100	2	32,6
CIMT+G-CSF	214-18-1i	100	3	69,1
CIMT+G-CSF	214-19-1i	100	3	43,3
CIMT+G-CSF	217-10-1i	100	3	42,6
CIMT+G-CSF	217-16-1i	100	4	82,8
CIMT+G-CSF	217-17-1i	100	6	104,3
CIMT+G-CSF	217-6-1i	100	8	112,2
CIMT+G-CSF	217-8-1i	100	2	37
CIMT+G-CSF	304-19-1i	100	2	28
CIMT+G-CSF	304-19-2i	100	3	38
CIMT+G-CSF	304-21-1i	100	1	14,9

CIMT+G-CSF	304-22-1i	100	1	10,4
CIMT+G-CSF	305-17-1i	100	2	21,4
CIMT+G-CSF	305-17-2i	100	5	73,3
CIMT+G-CSF	305-18-1i	100	1	10,6
CIMT+G-CSF	305-19-1i	100	4	29,5
CIMT+G-CSF	305-19-2i	100	3	40
CIMT/G-CSF	306-17-1i	100	3	64,6
CIMT/G-CSF	306-17-2i	100	1	11,1
CIMT/G-CSF	306-18-1i	100	4	82,6
CIMT/G-CSF	306-18-2i	100	1	12,5
CIMT/G-CSF	306-18-3i	100	2	17,2
CIMT/G-CSF	311-16-1i	100	2	29
CIMT/G-CSF	311-17-1i	100	7	116,7
CIMT/G-CSF	311-17-2i	100	6	72,8
CIMT/G-CSF	311-18-1i	100	2	31,7
CIMT/G-CSF	311-19-1i	100	7	103,7
CIMT/G-CSF	313-14.1i	100	2	23,1
CIMT/G-CSF	313-15-1i	100	2	30,4
CIMT/G-CSF	313-17-1i	100	2	27,4
CIMT/G-CSF	313-19-1i	100	1	10,7
CIMT/G-CSF	313-19-2i	100	3	47,8
CIMT/G-CSF	314-16-1i	100	6	39,2
CIMT/G-CSF	314-16-2i	100	5	60,5
CIMT/G-CSF	314-17-1i	100	1	10,6
CIMT/G-CSF	314-17-2i	100	3	25,9
CIMT/G-CSF	314-18-1i	100	2	27,1
CIMT/G-CSF	315-11-1i	100	1	13,3
CIMT/G-CSF	315-11-2i	100	1	11,6
CIMT/G-CSF	315-13-1i	100	3	35,9
CIMT/G-CSF	315-14-1i	100	2	36,8
CIMT/G-CSF	315-14-2i	100	4	57,8
G-CSF	220-16-1i	100	1	19,1
G-CSF	220-17-1i	100	3	30,9
G-CSF	220-18-1i	100	5	75,6
G-CSF	220-19-1i	100	4	51,1
G-CSF	220-7-1i	100	1	23
G-CSF	221-13-1i	100	2	37
G-CSF	221-16-1i	100	3	57,7
G-CSF	221-19-1i	100	8	107,7
G-CSF	221-22-1i	100	2	27,8
G-CSF	221-6-1i	100	2	29,6
G-CSF	222-16-1i	100	9	105,2
G-CSF	222-17-1i	100	3	57,7
G-CSF	222-21-1i	100	4	51,7
G-CSF	222-23-1i	100	5	49,4
G-CSF	222-8-1i	100	2	50,6
G-CSF	301-18-1i	100	4	119,8
G-CSF	301-19-1i	100	3	49,7
G-CSF	301-20-1i	100	1	10,7
G-CSF	301-20-2i	100	1	10,2
G-CSF	301-20-3i	100	3	33,9
G-CSF	302-16-1i	100	1	11,5
G-CSF	302-16-2i	100	1	14,8
G-CSF	302-17-1i	100	0	0,8
G-CSF	302-17-2i	100	1	10,1
G-CSF	302-18-1i	100	1	10,2

G-CSF	303-15-1i	100	6	61,2
G-CSF	303-16-1i	100	4	56,5
G-CSF	303-16-2i	100	1	13
G-CSF	303-17-1i	100	1	11
G-CSF	303-17-2i	100	1	37,2

apical dendrite Sholl 110µm

Group	number	Radius(µm)	Intersections	Length(µm)
Control	215-11-1i	110	1	35,1
Control	215-15-1i	110	1	11,5
Control	215-16-1i	110	2	36,5
Control	215-18-1i	110	3	53
Control	215-9-1i	110	1	20,1
Control	216-12-1i	110	1	15,9
Control	216-18-1i	110	0	3,8
Control	216-19-1i	110	1	29,7
Control	216-22-1i	110	2	21,4
Control	216-6-1i	110	5	85,4
Control	216-8-1i	110	4	79,4
Control	219-10-1i	110	2	23,8
Control	219-14-1i	110	4	52,9
Control	219-15-1i	110	1	10,6
Control	219-17-1i	110	2	40,9
Control	219-21-1i	110	2	34,6
Control	297-19-1i	110	3	68,7
Control	297-19-2i	110	4	118,2
Control	297-20-1i	110	3	47,1
Control	297-22-1i	110	2	34,3
Control	297-22-2i	110	4	51,8
Control	298-15-1i	110	2	31,2
Control	298-15-2i	110	4	154,6
Control	298-16-1i	110	5	64,9
Control	298-17-1i	110	8	102,4
Control	298-18-1i	110	4	43,5
Control	308-17-1i	110	1	44,8
Control	308-18-1i	110	2	25,9
Control	308-18-2i	110	6	103,6
Control	308-21-1i	110	2	26,9
Control	308-21-2i	110	4	56,6
CIMT	211-10-1i	110	5	56,7
CIMT	211-13-1i	110	2	43,7
CIMT	211-14-1i	110	2	29,3
CIMT	211-14-2i	110	1	32,7
CIMT	211-15-1i	110	1	11,5
CIMT	211-15-2i	110	1	14
CIMT	212-13-1i	110	3	55,8
CIMT	212-13-2i	110	3	34,7
CIMT	212-14-1i	110	3	34,8
CIMT	212-14-2i	110	1	38,3
CIMT	212-15-1i	110	2	23
CIMT	218-11-1i	110	2	25
CIMT	218-14-1i	110	2	34,4
CIMT	218-16-1i	110	1	11,2
CIMT	218-17-1i	110	3	39,5

CIMT	218-19-1i	110	2	23,8
CIMT	299-14-1i	110	2	20,8
CIMT	299-14-2i	110	2	26,5
CIMT	299-15-1i	110	2	73,6
CIMT	299-16-1i	110	2	21,3
CIMT	299-17-1i	110	2	24,5
CIMT	300-16-1i	110	1	18
CIMT	300-20-1i	110	3	38,3
CIMT	300-22-1i	110	2	25,7
CIMT	300-24-1i	110	7	83,9
CIMT	300-24-2i	110	3	37,4
CIMT	309-15-1i	110	5	66
CIMT	309-15-2i	110	3	56,2
CIMT	309-16-1i	110	1	12,4
CIMT	309-16-2i	110	5	68,5
CIMT	309-17-1i	110	3	51,4
CIMT+G-CSF	213-12-1i	110	2	20,6
CIMT+G-CSF	213-14-1i	110	1	16,3
CIMT+G-CSF	213-17-1i	110	1	15,4
CIMT+G-CSF	213-18-1i	110	4	46,3
CIMT+G-CSF	213-8-1i	110	1	13,9
CIMT+G-CSF	214-14-1i	110	1	12
CIMT+G-CSF	214-14-2i	110	0	15,8
CIMT+G-CSF	214-17-1i	110	3	32,6
CIMT+G-CSF	214-18-1i	110	3	37
CIMT+G-CSF	214-19-1i	110	2	51,1
CIMT+G-CSF	217-10-1i	110	3	39,4
CIMT+G-CSF	217-16-1i	110	4	49,4
CIMT+G-CSF	217-17-1i	110	6	66,8
CIMT+G-CSF	217-6-1i	110	8	133,3
CIMT+G-CSF	217-8-1i	110	2	23,5
CIMT+G-CSF	304-19-1i	110	2	25,8
CIMT+G-CSF	304-19-2i	110	2	31,1
CIMT+G-CSF	304-21-1i	110	1	10,3
CIMT+G-CSF	304-22-1i	110	1	10,3
CIMT+G-CSF	305-17-1i	110	2	27,4
CIMT+G-CSF	305-17-2i	110	3	52,3
CIMT+G-CSF	305-18-1i	110	1	11,2
CIMT+G-CSF	305-19-1i	110	3	48,1
CIMT+G-CSF	305-19-2i	110	3	35,1
CIMT/G-CSF	306-17-1i	110	3	74,2
CIMT/G-CSF	306-17-2i	110	1	10,2
CIMT/G-CSF	306-18-1i	110	4	51,7
CIMT/G-CSF	306-18-2i	110	1	15,3
CIMT/G-CSF	306-18-3i	110	1	38,2
CIMT/G-CSF	311-16-1i	110	1	23,2
CIMT/G-CSF	311-17-1i	110	9	114,8
CIMT/G-CSF	311-17-2i	110	8	148
CIMT/G-CSF	311-18-1i	110	2	31,7
CIMT/G-CSF	311-19-1i	110	6	75
CIMT/G-CSF	313-14.1i	110	1	18,7
CIMT/G-CSF	313-15-1i	110	2	32,2
CIMT/G-CSF	313-17-1i	110	1	28
CIMT/G-CSF	313-19-1i	110	1	10,5
CIMT/G-CSF	313-19-2i	110	4	83,2
CIMT/G-CSF	314-16-1i	110	3	61

CIMT/G-CSF	314-16-2i	110	2	38,7
CIMT/G-CSF	314-17-1i	110	2	33,9
CIMT/G-CSF	314-17-2i	110	2	32
CIMT/G-CSF	314-18-1i	110	2	28,2
CIMT/G-CSF	315-11-1i	110	2	22,8
CIMT/G-CSF	315-11-2i	110	2	35,4
CIMT/G-CSF	315-13-1i	110	0	8,8
CIMT/G-CSF	315-14-1i	110	2	24,8
CIMT/G-CSF	315-14-2i	110	3	45,3
G-CSF	220-16-1i	110	2	21,6
G-CSF	220-17-1i	110	2	24,2
G-CSF	220-18-1i	110	6	84,8
G-CSF	220-19-1i	110	2	86,2
G-CSF	220-7-1i	110	1	10,5
G-CSF	221-13-1i	110	2	21,1
G-CSF	221-16-1i	110	3	47,5
G-CSF	221-19-1i	110	11	130,5
G-CSF	221-22-1i	110	2	25,2
G-CSF	221-6-1i	110	1	15,2
G-CSF	222-16-1i	110	4	96,2
G-CSF	222-17-1i	110	2	36,7
G-CSF	222-21-1i	110	3	33
G-CSF	222-23-1i	110	4	55,2
G-CSF	222-8-1i	110	2	23,2
G-CSF	301-18-1i	110	4	64,5
G-CSF	301-19-1i	110	2	33,2
G-CSF	301-20-1i	110	1	10
G-CSF	301-20-2i	110	1	12,4
G-CSF	301-20-3i	110	3	33,6
G-CSF	302-16-1i	110	1	10,3
G-CSF	302-16-2i	110	2	67,8
G-CSF	302-17-2i	110	1	12,3
G-CSF	302-18-1i	110	1	13,2
G-CSF	303-15-1i	110	5	105,9
G-CSF	303-16-1i	110	4	46
G-CSF	303-16-2i	110	1	11,3
G-CSF	303-17-1i	110	0	3,9
G-CSF	303-17-2i	110	1	12,2

apical dendrite Sholl 120µm

Group	number	Radius(µm)	Intersections	Length(µm)
Control	215-11-1i	120	1	12,3
Control	215-15-1i	120	1	11,8
Control	215-16-1i	120	2	29,6
Control	215-18-1i	120	2	31
Control	215-9-1i	120	1	19,5
Control	216-12-1i	120	1	10,7
Control	216-19-1i	120	1	11
Control	216-22-1i	120	2	22
Control	216-6-1i	120	5	69,1
Control	216-8-1i	120	4	57,5
Control	219-10-1i	120	3	53,6
Control	219-14-1i	120	5	58,1
Control	219-15-1i	120	2	37,1

Control	219-17-1i	120	3	36,5
Control	219-21-1i	120	1	25,3
Control	297-19-1i	120	2	101,5
Control	297-19-2i	120	2	29,1
Control	297-20-1i	120	3	33,7
Control	297-22-1i	120	2	25,3
Control	297-22-2i	120	2	43,2
Control	298-15-1i	120	1	18,3
Control	298-15-2i	120	4	79,7
Control	298-16-1i	120	6	168,1
Control	298-17-1i	120	9	113,6
Control	298-18-1i	120	2	29,4
Control	308-17-1i	120	1	11,6
Control	308-18-1i	120	3	68,2
Control	308-18-2i	120	5	77,1
Control	308-21-1i	120	3	29,8
Control	308-21-2i	120	4	52,6
CIMT	211-10-1i	120	5	61,5
CIMT	211-13-1i	120	2	25
CIMT	211-14-1i	120	1	20,3
CIMT	211-14-2i	120	1	48,5
CIMT	211-15-1i	120	1	10,5
CIMT	211-15-2i	120	1	10,3
CIMT	212-13-1i	120	2	34,3
CIMT	212-13-2i	120	2	23,8
CIMT	212-14-1i	120	2	31,4
CIMT	212-14-2i	120	1	10,5
CIMT	212-15-1i	120	2	28
CIMT	218-11-1i	120	1	15,8
CIMT	218-14-1i	120	2	31,6
CIMT	218-16-1i	120	1	11,3
CIMT	218-17-1i	120	4	67,9
CIMT	218-19-1i	120	2	27,6
CIMT	299-14-1i	120	2	22,7
CIMT	299-14-2i	120	2	23,9
CIMT	299-15-1i	120	1	10,9
CIMT	299-16-1i	120	2	25,7
CIMT	299-17-1i	120	4	44,4
CIMT	300-16-1i	120	1	10,8
CIMT	300-20-1i	120	2	28,4
CIMT	300-22-1i	120	0	17,8
CIMT	300-24-1i	120	4	73,7
CIMT	300-24-2i	120	0	19,9
CIMT	309-15-1i	120	6	78,4
CIMT	309-15-2i	120	4	48,6
CIMT	309-16-1i	120	1	10,3
CIMT	309-16-2i	120	5	67,6
CIMT	309-17-1i	120	3	34,8
CIMT+G-CSF	213-12-1i	120	0	14
CIMT+G-CSF	213-14-1i	120	2	20,2
CIMT+G-CSF	213-17-1i	120	1	10,2
CIMT+G-CSF	213-18-1i	120	0	21,7
CIMT+G-CSF	213-8-1i	120	1	11,1
CIMT+G-CSF	214-14-1i	120	1	13,5
CIMT+G-CSF	214-17-1i	120	2	32,4
CIMT+G-CSF	214-18-1i	120	4	35,3

CIMT+G-CSF	214-19-1i	120	0	23,7
CIMT+G-CSF	217-10-1i	120	6	40,6
CIMT+G-CSF	217-16-1i	120	4	49,3
CIMT+G-CSF	217-17-1i	120	8	80,5
CIMT+G-CSF	217-6-1i	120	7	109,2
CIMT+G-CSF	217-8-1i	120	2	21,9
CIMT+G-CSF	304-19-1i	120	1	22,7
CIMT+G-CSF	304-19-2i	120	1	18,4
CIMT+G-CSF	304-21-1i	120	0	4,6
CIMT+G-CSF	304-22-1i	120	1	11,2
CIMT+G-CSF	305-17-1i	120	2	23,2
CIMT+G-CSF	305-17-2i	120	2	48,6
CIMT+G-CSF	305-18-1i	120	1	10,4
CIMT+G-CSF	305-19-1i	120	3	35,6
CIMT+G-CSF	305-19-2i	120	3	33,1
CIMT/G-CSF	306-17-1i	120	3	40,1
CIMT/G-CSF	306-17-2i	120	1	10,8
CIMT/G-CSF	306-18-1i	120	3	70,5
CIMT/G-CSF	306-18-2i	120	1	10,6
CIMT/G-CSF	306-18-3i	120	2	28,7
CIMT/G-CSF	311-16-1i	120	2	65,2
CIMT/G-CSF	311-17-1i	120	8	102,9
CIMT/G-CSF	311-17-2i	120	8	107,3
CIMT/G-CSF	311-18-1i	120	2	25,9
CIMT/G-CSF	311-19-1i	120	7	108,4
CIMT/G-CSF	313-14.1i	120	0	3,4
CIMT/G-CSF	313-15-1i	120	1	22,6
CIMT/G-CSF	313-17-1i	120	1	11
CIMT/G-CSF	313-19-1i	120	1	11,1
CIMT/G-CSF	313-19-2i	120	2	37,3
CIMT/G-CSF	314-16-1i	120	3	48,1
CIMT/G-CSF	314-16-2i	120	2	22,6
CIMT/G-CSF	314-17-1i	120	2	26,3
CIMT/G-CSF	314-17-2i	120	3	49,9
CIMT/G-CSF	314-18-1i	120	5	53,2
CIMT/G-CSF	315-11-1i	120	1	12,7
CIMT/G-CSF	315-11-2i	120	2	31,9
CIMT/G-CSF	315-14-1i	120	2	23,4
CIMT/G-CSF	315-14-2i	120	2	33,3
G-CSF	220-16-1i	120	1	22,4
G-CSF	220-17-1i	120	1	12,9
G-CSF	220-18-1i	120	3	80,9
G-CSF	220-19-1i	120	1	27,3
G-CSF	220-7-1i	120	1	10,6
G-CSF	221-13-1i	120	2	21,5
G-CSF	221-16-1i	120	2	36,4
G-CSF	221-19-1i	120	9	174,3
G-CSF	221-22-1i	120	2	47,6
G-CSF	221-6-1i	120	1	11
G-CSF	222-16-1i	120	3	36,3
G-CSF	222-17-1i	120	2	27,8
G-CSF	222-21-1i	120	2	29
G-CSF	222-23-1i	120	3	46,2
G-CSF	222-8-1i	120	3	24,9
G-CSF	301-18-1i	120	4	57
G-CSF	301-19-1i	120	2	26,2

G-CSF	301-20-1i	120	1	10,8
G-CSF	301-20-2i	120	1	10,4
G-CSF	301-20-3i	120	3	31,4
G-CSF	302-16-1i	120	1	12,8
G-CSF	302-16-2i	120	1	21,1
G-CSF	302-17-2i	120	1	11,2
G-CSF	302-18-1i	120	1	11,7
G-CSF	303-15-1i	120	5	68,6
G-CSF	303-16-1i	120	3	42
G-CSF	303-16-2i	120	3	52
G-CSF	303-17-2i	120	0	8,6

apical dendrite Sholl 140µm

Group	number	Radius(µm)	Intersections	Length(µm)
Control	215-11-1i	140	1	12,8
Control	215-15-1i	140	0	12,1
Control	215-16-1i	140	2	22,8
Control	215-18-1i	140	0	11,1
Control	215-9-1i	140	0	11,5
Control	216-12-1i	140	1	11,9
Control	216-19-1i	140	1	10,2
Control	216-22-1i	140	1	20,4
Control	216-6-1i	140	2	31,8
Control	216-8-1i	140	3	33,7
Control	219-10-1i	140	4	128,2
Control	219-14-1i	140	2	42,8
Control	219-15-1i	140	1	25,9
Control	219-17-1i	140	2	27,1
Control	219-21-1i	140	0	12,2
Control	297-19-1i	140	1	11,5
Control	297-19-2i	140	4	85,5
Control	297-20-1i	140	3	144,4
Control	297-22-1i	140	2	21,8
Control	298-15-2i	140	2	23,3
Control	298-16-1i	140	2	77,1
Control	298-17-1i	140	8	85,7
Control	298-18-1i	140	1	17,9
Control	308-17-1i	140	1	10,9
Control	308-18-1i	140	1	57,5
Control	308-18-2i	140	3	40,8
Control	308-21-1i	140	2	31,7
Control	308-21-2i	140	1	10,5
CIMT	211-10-1i	140	5	48,9
CIMT	211-13-1i	140	3	56
CIMT	211-14-1i	140	1	12,2
CIMT	211-15-1i	140	2	48,7
CIMT	211-15-2i	140	0	24
CIMT	212-13-1i	140	3	22,9
CIMT	212-13-2i	140	2	13,5
CIMT	212-14-1i	140	1	14,2
CIMT	212-14-2i	140	2	20,1
CIMT	212-15-1i	140	3	41,6
CIMT	218-11-1i	140	1	10,5
CIMT	218-14-1i	140	1	19,1

CIMT	218-16-1i	140	3	80,5
CIMT	218-17-1i	140	2	21,9
CIMT	218-19-1i	140	1	11,3
CIMT	299-14-1i	140	0	14,2
CIMT	299-14-2i	140	1	24
CIMT	299-15-1i	140	1	16,5
CIMT	299-16-1i	140	2	59,3
CIMT	299-17-1i	140	2	21,1
CIMT	300-20-1i	140	0	6,5
CIMT	300-24-1i	140	4	47
CIMT	309-15-1i	140	4	70,1
CIMT	309-15-2i	140	1	10,8
CIMT	309-16-1i	140	2	10,9
CIMT	309-16-2i	140	2	37,8
CIMT	309-17-1i	140	2	61,3
CIMT+G-CSF	213-14-1i	140	0	8,7
CIMT+G-CSF	213-17-1i	140	2	11
CIMT+G-CSF	213-8-1i	140	0	7,3
CIMT+G-CSF	214-14-1i	140	3	54,9
CIMT+G-CSF	214-17-1i	140	2	28
CIMT+G-CSF	217-10-1i	140	2	30,1
CIMT+G-CSF	217-16-1i	140	3	41,9
CIMT+G-CSF	217-17-1i	140	3	52,4
CIMT+G-CSF	217-6-1i	140	4	60,6
CIMT+G-CSF	217-8-1i	140	3	40,9
CIMT+G-CSF	304-19-1i	140	1	11
CIMT+G-CSF	304-19-2i	140	0	4,3
CIMT+G-CSF	305-17-1i	140	1	11,7
CIMT+G-CSF	305-17-2i	140	0	11
CIMT+G-CSF	305-18-1i	140	0	10,9
CIMT+G-CSF	305-19-1i	140	3	36,5
CIMT+G-CSF	305-19-2i	140	3	48,4
CIMT/G-CSF	306-17-1i	140	3	45,9
CIMT/G-CSF	306-17-2i	140	1	10,9
CIMT/G-CSF	306-18-1i	140	1	39,9
CIMT/G-CSF	306-18-2i	140	1	12
CIMT/G-CSF	306-18-3i	140	2	21,6
CIMT/G-CSF	311-16-1i	140	2	43,2
CIMT/G-CSF	311-17-1i	140	5	88,2
CIMT/G-CSF	311-17-2i	140	3	48,8
CIMT/G-CSF	311-18-1i	140	1	10,2
CIMT/G-CSF	311-19-1i	140	6	95
CIMT/G-CSF	313-15-1i	140	2	27,4
CIMT/G-CSF	313-17-1i	140	1	18,8
CIMT/G-CSF	313-19-1i	140	2	30,6
CIMT/G-CSF	313-19-2i	140	2	43,8
CIMT/G-CSF	314-16-1i	140	2	24,2
CIMT/G-CSF	314-16-2i	140	1	16,7
CIMT/G-CSF	314-17-1i	140	2	22,5
CIMT/G-CSF	314-17-2i	140	3	70,2
CIMT/G-CSF	314-18-1i	140	1	40,6
CIMT/G-CSF	315-11-1i	140	1	19,7
CIMT/G-CSF	315-11-2i	140	3	60,9
CIMT/G-CSF	315-14-1i	140	2	23
CIMT/G-CSF	315-14-2i	140	0	3,4
G-CSF	220-17-1i	140	1	10,4

G-CSF	220-18-1i	140	1	10,8
G-CSF	220-19-1i	140	3	68,4
G-CSF	220-7-1i	140	1	11
G-CSF	221-13-1i	140	1	11
G-CSF	221-16-1i	140	3	39,1
G-CSF	221-19-1i	140	6	76,8
G-CSF	221-22-1i	140	2	25,8
G-CSF	221-6-1i	140	1	10,5
G-CSF	222-16-1i	140	1	21,7
G-CSF	222-17-1i	140	1	18,5
G-CSF	222-21-1i	140	2	22,2
G-CSF	222-23-1i	140	1	11,8
G-CSF	222-8-1i	140	1	21,4
G-CSF	301-18-1i	140	1	58,5
G-CSF	301-19-1i	140	1	13,1
G-CSF	301-20-1i	140	1	11,5
G-CSF	301-20-2i	140	1	14,5
G-CSF	301-20-3i	140	4	62,8
G-CSF	302-16-1i	140	1	14,2
G-CSF	302-16-2i	140	0	13,2
G-CSF	302-17-2i	140	1	10,4
G-CSF	302-18-1i	140	1	10,3
G-CSF	303-15-1i	140	2	21
G-CSF	303-16-1i	140	3	34,9
G-CSF	303-16-2i	140	3	57,7

apical dendrite Sholl 150µm +

Group	number	Radius(µm)	Intersections	Length(µm)
Control	215-11-1i	150	1	12,3
Control	215-16-1i	150	2	26,8
Control	216-12-1i	150	1	13,7
Control	216-19-1i	150	0	10,4
Control	216-22-1i	150	1	12
Control	216-6-1i	150	2	25,3
Control	216-8-1i	150	3	39,3
Control	219-10-1i	150	2	80,7
Control	219-14-1i	150	2	20,3
Control	219-15-1i	150	2	37
Control	219-17-1i	150	2	24
Control	297-19-1i	150	3	20,5
Control	297-19-2i	150	2	38,5
Control	297-20-1i	150	2	29,9
Control	297-22-1i	150	2	22
Control	298-15-2i	150	1	25
Control	298-16-1i	150	1	18,9
Control	298-17-1i	150	4	62,3
Control	298-18-1i	150	1	12
Control	308-17-1i	150	1	15,5
Control	308-18-1i	150	1	18
Control	308-18-2i	150	4	107,7
Control	308-21-1i	150	2	22,6
Control	308-21-2i	150	1	11,7
Control	215-11-1i	160	0	1,9
Control	215-16-1i	160	1	27,8

Control	216-12-1i	160	1	11,2
Control	216-22-1i	160	1	10,2
Control	216-6-1i	160	2	28,1
Control	216-8-1i	160	2	25,9
Control	219-10-1i	160	2	27,6
Control	219-14-1i	160	2	20,9
Control	219-15-1i	160	2	48,7
Control	219-17-1i	160	1	13,1
Control	297-19-1i	160	2	81,6
Control	297-19-2i	160	1	15,3
Control	297-20-1i	160	1	12,7
Control	297-22-1i	160	2	20,8
Control	298-15-2i	160	1	10,5
Control	298-16-1i	160	1	13
Control	298-17-1i	160	5	51,1
Control	298-18-1i	160	0	11,4
Control	308-17-1i	160	1	21,4
Control	308-18-1i	160	1	10,3
Control	308-18-2i	160	2	56,2
Control	308-21-1i	160	1	20
Control	308-21-2i	160	1	10,2
Control	215-16-1i	170	0	1
Control	216-12-1i	170	1	11
Control	216-22-1i	170	1	10,2
Control	216-6-1i	170	1	22,1
Control	216-8-1i	170	2	27,6
Control	219-10-1i	170	3	29,5
Control	219-14-1i	170	4	48,2
Control	219-15-1i	170	0	10,1
Control	219-17-1i	170	1	11,7
Control	297-19-1i	170	4	55,5
Control	297-19-2i	170	1	10,3
Control	297-20-1i	170	2	16,8
Control	297-22-1i	170	2	22,4
Control	298-15-2i	170	1	10,4
Control	298-16-1i	170	1	24,6
Control	298-17-1i	170	6	60,2
Control	308-17-1i	170	1	10,9
Control	308-18-1i	170	1	10,1
Control	308-18-2i	170	2	21,8
Control	308-21-1i	170	0	1,5
Control	308-21-2i	170	1	10,8
Control	216-12-1i	180	1	10,2
Control	216-22-1i	180	1	11,9
Control	216-6-1i	180	1	11
Control	216-8-1i	180	3	39,2
Control	219-10-1i	180	2	56,9
Control	219-14-1i	180	1	37,8
Control	219-17-1i	180	1	10,8
Control	297-19-1i	180	5	87
Control	297-19-2i	180	1	10,1
Control	297-20-1i	180	1	29
Control	297-22-1i	180	2	25,3
Control	298-15-2i	180	1	10,3
Control	298-16-1i	180	2	18,5
Control	298-17-1i	180	5	71,6

Control	308-17-1i	180	2	30
Control	308-18-1i	180	1	12,8
Control	308-18-2i	180	2	60,2
Control	308-21-2i	180	1	10,8
Control	216-12-1i	190	2	20,1
Control	216-22-1i	190	1	10,7
Control	216-6-1i	190	1	10,3
Control	216-8-1i	190	2	29,7
Control	219-10-1i	190	4	77,6
Control	219-14-1i	190	2	18,5
Control	219-17-1i	190	1	48,1
Control	297-19-1i	190	4	70,2
Control	297-19-2i	190	1	10,2
Control	297-20-1i	190	0	9,7
Control	297-22-1i	190	3	34,1
Control	298-15-2i	190	0	8,7
Control	298-16-1i	190	2	28,4
Control	298-17-1i	190	4	73
Control	308-17-1i	190	2	33
Control	308-18-1i	190	1	10,6
Control	308-18-2i	190	2	22,3
Control	308-21-2i	190	1	12,6
Control	216-12-1i	200	0	13,4
Control	216-22-1i	200	1	11
Control	216-6-1i	200	0	6,1
Control	216-8-1i	200	0	20,8
Control	219-10-1i	200	1	53,9
Control	219-14-1i	200	2	34,5
Control	219-17-1i	200	1	10,7
Control	297-19-1i	200	2	49,9
Control	297-19-2i	200	0	8
Control	297-22-1i	200	3	32,2
Control	298-16-1i	200	1	11,6
Control	298-17-1i	200	4	51,7
Control	308-17-1i	200	1	29,9
Control	308-18-1i	200	1	11,1
Control	308-18-2i	200	0	13
Control	308-21-2i	200	0	0,1
Control	216-22-1i	210	1	11
Control	219-10-1i	210	2	27,4
Control	219-14-1i	210	2	26,3
Control	219-17-1i	210	1	11
Control	297-19-1i	210	3	38,8
Control	297-22-1i	210	1	26,1
Control	298-16-1i	210	1	11,7
Control	298-17-1i	210	2	27,6
Control	308-17-1i	210	0	13,7
Control	308-18-1i	210	1	20
Control	216-22-1i	220	0	2,8
Control	219-10-1i	220	1	23,4
Control	219-14-1i	220	2	22,9
Control	219-17-1i	220	1	11,6
Control	297-19-1i	220	3	38,5
Control	297-22-1i	220	2	21,3
Control	298-16-1i	220	1	16,8
Control	298-17-1i	220	2	23

Control	308-18-1i	220	1	14,1
Control	219-10-1i	230	1	10,1
Control	219-14-1i	230	2	24
Control	219-17-1i	230	1	10,1
Control	297-19-1i	230	2	33,9
Control	297-22-1i	230	0	36,5
Control	298-16-1i	230	0	3,1
Control	298-17-1i	230	2	22,7
Control	308-18-1i	230	1	13,9
Control	219-10-1i	240	1	10
Control	219-14-1i	240	1	19
Control	219-17-1i	240	1	11,7
Control	297-19-1i	240	1	30,2
Control	298-17-1i	240	1	28,7
Control	308-18-1i	240	1	11,6
Control	219-10-1i	250	1	10,4
Control	219-14-1i	250	1	10,6
Control	219-17-1i	250	1	10,7
Control	297-19-1i	250	1	10,1
Control	298-17-1i	250	1	11,4
Control	308-18-1i	250	1	20,1
Control	219-10-1i	260	1	13,5
Control	219-14-1i	260	1	11,9
Control	219-17-1i	260	1	10,3
Control	297-19-1i	260	1	11,5
Control	298-17-1i	260	0	1,9
Control	308-18-1i	260	0	2,2
Control	219-10-1i	270	1	10,4
Control	219-14-1i	270	1	10,3
Control	219-17-1i	270	1	10,5
Control	297-19-1i	270	1	10,6
Control	219-10-1i	280	1	10,1
Control	219-14-1i	280	1	12,8
Control	219-17-1i	280	1	11
Control	297-19-1i	280	1	13,3
Control	219-10-1i	290	1	10
Control	219-14-1i	290	1	10,8
Control	219-17-1i	290	0	1,4
Control	297-19-1i	290	2	22,7
Control	219-10-1i	300	1	10
Control	219-14-1i	300	1	10,5
Control	297-19-1i	300	0	11,7
Control	219-10-1i	310	1	11,8
Control	219-14-1i	310	0	5,1
Control	219-10-1i	320	1	10,4
Control	219-10-1i	330	1	11
Control	219-10-1i	340	0	4,9
CIMT	211-10-1i	150	2	116,6
CIMT	211-13-1i	150	4	36,5
CIMT	211-14-1i	150	1	11,2
CIMT	211-15-1i	150	2	37,3
CIMT	212-13-1i	150	2	37,9
CIMT	212-13-2i	150	3	46,7
CIMT	212-14-1i	150	1	10,2
CIMT	212-14-2i	150	1	17,6
CIMT	212-15-1i	150	2	26,9

CIMT	218-11-1i	150	1	11,3
CIMT	218-14-1i	150	1	10,5
CIMT	218-16-1i	150	3	46,4
CIMT	218-17-1i	150	2	22,5
CIMT	218-19-1i	150	1	10,1
CIMT	299-14-2i	150	0	4,7
CIMT	299-15-1i	150	0	2,6
CIMT	299-16-1i	150	2	28,1
CIMT	299-17-1i	150	3	40,9
CIMT	300-24-1i	150	3	57,6
CIMT	309-15-1i	150	2	40,6
CIMT	309-15-2i	150	0	2,5
CIMT	309-16-1i	150	2	32
CIMT	309-16-2i	150	1	30
CIMT	309-17-1i	150	1	20,6
CIMT	211-10-1i	160	2	31,4
CIMT	211-13-1i	160	3	61,3
CIMT	211-14-1i	160	2	12
CIMT	211-15-1i	160	2	27,7
CIMT	212-13-1i	160	1	17,1
CIMT	212-13-2i	160	1	26,1
CIMT	212-14-1i	160	0	7,2
CIMT	212-14-2i	160	1	11
CIMT	212-15-1i	160	3	35,7
CIMT	218-11-1i	160	1	11,7
CIMT	218-14-1i	160	1	10,3
CIMT	218-16-1i	160	3	51,1
CIMT	218-17-1i	160	2	21,1
CIMT	218-19-1i	160	1	10,5
CIMT	299-16-1i	160	1	13,6
CIMT	299-17-1i	160	1	27,7
CIMT	300-24-1i	160	2	54
CIMT	309-15-1i	160	2	24,9
CIMT	309-16-1i	160	3	79
CIMT	309-16-2i	160	0	4,4
CIMT	309-17-1i	160	0	15,4
CIMT	211-10-1i	170	2	26,8
CIMT	211-13-1i	170	4	44,6
CIMT	211-14-1i	170	1	17,7
CIMT	211-15-1i	170	2	24
CIMT	212-13-1i	170	1	11,5
CIMT	212-13-2i	170	1	14
CIMT	212-14-2i	170	1	16,9
CIMT	212-15-1i	170	2	53,6
CIMT	218-11-1i	170	1	10,2
CIMT	218-14-1i	170	1	10,2
CIMT	218-16-1i	170	3	62,1
CIMT	218-17-1i	170	2	21,1
CIMT	218-19-1i	170	1	10,9
CIMT	299-16-1i	170	1	10,5
CIMT	299-17-1i	170	1	10,2
CIMT	300-24-1i	170	2	25
CIMT	309-15-1i	170	2	23,8
CIMT	309-16-1i	170	6	50,8
CIMT	211-10-1i	180	2	21,6
CIMT	211-13-1i	180	0	1,5

CIMT	211-14-1i	180	1	10,6
CIMT	211-15-1i	180	2	24,8
CIMT	212-13-1i	180	0	1,1
CIMT	212-13-2i	180	0	8,6
CIMT	212-14-2i	180	0	4,9
CIMT	212-15-1i	180	1	21,4
CIMT	218-11-1i	180	1	10,4
CIMT	218-14-1i	180	1	10,1
CIMT	218-16-1i	180	2	53
CIMT	218-17-1i	180	2	20,2
CIMT	218-19-1i	180	1	10,7
CIMT	299-16-1i	180	1	11,8
CIMT	299-17-1i	180	1	10,2
CIMT	300-24-1i	180	1	19,9
CIMT	309-15-1i	180	2	35,4
CIMT	309-16-1i	180	3	72,2
CIMT	211-10-1i	190	2	26,9
CIMT	211-14-1i	190	0	14,1
CIMT	211-15-1i	190	2	23,6
CIMT	212-15-1i	190	1	11,2
CIMT	218-11-1i	190	1	12,3
CIMT	218-14-1i	190	1	10,6
CIMT	218-16-1i	190	2	27,7
CIMT	218-17-1i	190	2	22,1
CIMT	218-19-1i	190	1	10,8
CIMT	299-16-1i	190	0	4,5
CIMT	299-17-1i	190	5	18,5
CIMT	300-24-1i	190	2	24,8
CIMT	309-15-1i	190	2	72,8
CIMT	309-16-1i	190	2	29
CIMT	211-10-1i	200	0	14,8
CIMT	211-15-1i	200	2	26,3
CIMT	212-15-1i	200	1	12,7
CIMT	218-11-1i	200	1	12,4
CIMT	218-14-1i	200	1	14,6
CIMT	218-16-1i	200	2	26,9
CIMT	218-17-1i	200	1	13,2
CIMT	218-19-1i	200	1	14,1
CIMT	299-17-1i	200	2	37,8
CIMT	300-24-1i	200	1	18,1
CIMT	309-15-1i	200	2	27,9
CIMT	309-16-1i	200	2	29,9
CIMT	211-15-1i	210	1	26,2
CIMT	212-15-1i	210	0	7,3
CIMT	218-11-1i	210	1	11,8
CIMT	218-14-1i	210	0	2,2
CIMT	218-16-1i	210	2	25,8
CIMT	218-17-1i	210	1	10,3
CIMT	218-19-1i	210	1	11,6
CIMT	299-17-1i	210	1	25,3
CIMT	300-24-1i	210	1	10,3
CIMT	309-15-1i	210	2	28,6
CIMT	309-16-1i	210	2	25,5
CIMT	211-15-1i	220	1	10,2
CIMT	218-11-1i	220	0	1,1
CIMT	218-16-1i	220	2	25,5

CIMT	218-17-1i	220	1	10,6
CIMT	218-19-1i	220	0	1,5
CIMT	299-17-1i	220	3	33,4
CIMT	300-24-1i	220	1	10,5
CIMT	309-15-1i	220	2	33,6
CIMT	309-16-1i	220	2	24,1
CIMT	211-15-1i	230	1	10,5
CIMT	218-16-1i	230	2	23,1
CIMT	218-17-1i	230	1	11,6
CIMT	299-17-1i	230	3	35,7
CIMT	300-24-1i	230	0	7
CIMT	309-15-1i	230	2	28,8
CIMT	309-16-1i	230	2	26,4
CIMT	211-15-1i	240	0	2,2
CIMT	218-16-1i	240	2	21,3
CIMT	218-17-1i	240	1	11,2
CIMT	299-17-1i	240	2	35,3
CIMT	309-15-1i	240	1	44,5
CIMT	309-16-1i	240	2	28,2
CIMT	218-16-1i	250	2	24,5
CIMT	218-17-1i	250	1	11,4
CIMT	299-17-1i	250	2	22,5
CIMT	309-15-1i	250	2	19,9
CIMT	309-16-1i	250	2	24,1
CIMT	218-16-1i	260	2	26,7
CIMT	218-17-1i	260	1	10,2
CIMT	299-17-1i	260	2	22,3
CIMT	309-15-1i	260	0	40,3
CIMT	309-16-1i	260	1	31
CIMT	218-16-1i	270	2	26
CIMT	218-17-1i	270	2	28,1
CIMT	299-17-1i	270	3	44,3
CIMT	309-16-1i	270	2	15,1
CIMT	218-16-1i	280	2	23,1
CIMT	218-17-1i	280	1	18,3
CIMT	299-17-1i	280	2	30,5
CIMT	309-16-1i	280	3	34,8
CIMT	218-16-1i	290	1	14
CIMT	218-17-1i	290	1	10,5
CIMT	299-17-1i	290	1	19,7
CIMT	309-16-1i	290	1	28,6
CIMT	218-16-1i	300	1	10,9
CIMT	218-17-1i	300	1	10,4
CIMT	299-17-1i	300	1	10,1
CIMT	309-16-1i	300	0	6,9
CIMT	218-16-1i	310	1	10,4
CIMT	218-17-1i	310	1	10,2
CIMT	299-17-1i	310	1	12,2
CIMT	218-16-1i	320	1	10,5
CIMT	218-17-1i	320	1	11,5
CIMT	299-17-1i	320	1	10,2
CIMT	218-16-1i	330	1	10,9
CIMT	218-17-1i	330	1	10,4
CIMT	299-17-1i	330	1	10,1
CIMT	218-16-1i	340	0	1,8
CIMT	218-17-1i	340	1	10,4

CIMT	299-17-1i	340	1	10,3
CIMT	218-17-1i	350	1	20
CIMT	299-17-1i	350	1	11,3
CIMT	218-17-1i	360	1	11,7
CIMT	299-17-1i	360	1	10,2
CIMT	218-17-1i	370	1	10,2
CIMT	299-17-1i	370	0	2,9
CIMT	218-17-1i	380	1	10,3
CIMT	218-17-1i	390	1	10,5
CIMT	218-17-1i	400	1	11,2
CIMT	218-17-1i	410	1	10,6
CIMT	218-17-1i	420	0	0,9
CIMT+G-CSF	213-17-1i	150	1	56,7
CIMT+G-CSF	214-14-1i	150	1	26,3
CIMT+G-CSF	214-17-1i	150	0	12,1
CIMT+G-CSF	217-10-1i	150	1	18,7
CIMT+G-CSF	217-16-1i	150	3	37,2
CIMT+G-CSF	217-17-1i	150	1	31,1
CIMT+G-CSF	217-6-1i	150	5	56
CIMT+G-CSF	217-8-1i	150	2	29,7
CIMT+G-CSF	304-19-1i	150	1	12,3
CIMT+G-CSF	305-17-1i	150	0	8,8
CIMT+G-CSF	305-19-1i	150	1	26,7
CIMT+G-CSF	305-19-2i	150	1	12,3
CIMT+G-CSF	213-17-1i	160	1	10,8
CIMT+G-CSF	214-14-1i	160	2	21,5
CIMT+G-CSF	217-10-1i	160	1	10,7
CIMT+G-CSF	217-16-1i	160	3	33,4
CIMT+G-CSF	217-17-1i	160	1	10,7
CIMT+G-CSF	217-6-1i	160	5	59,2
CIMT+G-CSF	217-8-1i	160	3	29,6
CIMT+G-CSF	304-19-1i	160	0	1,5
CIMT+G-CSF	305-19-1i	160	0	1,4
CIMT+G-CSF	305-19-2i	160	0	4,3
CIMT+G-CSF	213-17-1i	170	0	5
CIMT+G-CSF	214-14-1i	170	1	14,6
CIMT+G-CSF	217-10-1i	170	1	11,4
CIMT+G-CSF	217-16-1i	170	3	35
CIMT+G-CSF	217-17-1i	170	1	14,3
CIMT+G-CSF	217-6-1i	170	3	53,7
CIMT+G-CSF	217-8-1i	170	1	19,3
CIMT+G-CSF	214-14-1i	180	1	15,9
CIMT+G-CSF	217-10-1i	180	1	10,8
CIMT+G-CSF	217-16-1i	180	2	23,4
CIMT+G-CSF	217-17-1i	180	1	10
CIMT+G-CSF	217-6-1i	180	2	35,5
CIMT+G-CSF	217-8-1i	180	1	10,4
CIMT+G-CSF	214-14-1i	190	4	12,7
CIMT+G-CSF	217-10-1i	190	1	10,5
CIMT+G-CSF	217-16-1i	190	1	19,5
CIMT+G-CSF	217-17-1i	190	1	10,6
CIMT+G-CSF	217-6-1i	190	2	21,8
CIMT+G-CSF	217-8-1i	190	1	11,7
CIMT+G-CSF	214-14-1i	200	2	27,7
CIMT+G-CSF	217-10-1i	200	1	10,5
CIMT+G-CSF	217-16-1i	200	0	8,8

CIMT+G-CSF	217-17-1i	200	1	10,2
CIMT+G-CSF	217-6-1i	200	2	21,6
CIMT+G-CSF	217-8-1i	200	1	10,1
CIMT+G-CSF	214-14-1i	210	1	30
CIMT+G-CSF	217-10-1i	210	1	10,3
CIMT+G-CSF	217-17-1i	210	1	12,4
CIMT+G-CSF	217-6-1i	210	1	11,7
CIMT+G-CSF	217-8-1i	210	1	10,5
CIMT+G-CSF	214-14-1i	220	1	11,2
CIMT+G-CSF	217-10-1i	220	1	10,6
CIMT+G-CSF	217-17-1i	220	0	0,7
CIMT+G-CSF	217-6-1i	220	0	14,3
CIMT+G-CSF	217-8-1i	220	2	25,8
CIMT+G-CSF	214-14-1i	230	3	20,6
CIMT+G-CSF	217-10-1i	230	1	18
CIMT+G-CSF	217-8-1i	230	2	29,7
CIMT+G-CSF	214-14-1i	240	0	22,9
CIMT+G-CSF	217-10-1i	240	1	11,5
CIMT+G-CSF	217-8-1i	240	1	16,1
CIMT+G-CSF	217-10-1i	250	1	10,6
CIMT+G-CSF	217-8-1i	250	1	11,1
CIMT+G-CSF	217-10-1i	260	1	11,3
CIMT+G-CSF	217-8-1i	260	0	1,3
CIMT+G-CSF	217-10-1i	270	1	19,3
CIMT+G-CSF	217-10-1i	280	1	10,5
CIMT+G-CSF	217-10-1i	290	1	11
CIMT+G-CSF	217-10-1i	300	1	10,5
CIMT+G-CSF	217-10-1i	310	0	7,9
CIMT/G-CSF	306-17-1i	150	2	69,8
CIMT/G-CSF	306-17-2i	150	1	11,1
CIMT/G-CSF	306-18-1i	150	1	13,9
CIMT/G-CSF	306-18-2i	150	1	10,1
CIMT/G-CSF	306-18-3i	150	2	24,1
CIMT/G-CSF	311-16-1i	150	2	26
CIMT/G-CSF	311-17-1i	150	4	59
CIMT/G-CSF	311-17-2i	150	2	30,7
CIMT/G-CSF	311-18-1i	150	1	11,4
CIMT/G-CSF	311-19-1i	150	11	120
CIMT/G-CSF	313-15-1i	150	3	43,1
CIMT/G-CSF	313-17-1i	150	1	17,8
CIMT/G-CSF	313-19-1i	150	1	22
CIMT/G-CSF	313-19-2i	150	2	37,6
CIMT/G-CSF	314-16-1i	150	2	27,5
CIMT/G-CSF	314-16-2i	150	1	12,2
CIMT/G-CSF	314-17-1i	150	2	22,7
CIMT/G-CSF	314-17-2i	150	2	37,1
CIMT/G-CSF	314-18-1i	150	1	14,7
CIMT/G-CSF	315-11-1i	150	1	21
CIMT/G-CSF	315-11-2i	150	1	35,7
CIMT/G-CSF	315-14-1i	150	2	24,3
CIMT/G-CSF	306-17-1i	160	3	31
CIMT/G-CSF	306-17-2i	160	1	10,8
CIMT/G-CSF	306-18-1i	160	1	11,4
CIMT/G-CSF	306-18-2i	160	1	10,9
CIMT/G-CSF	306-18-3i	160	2	22,4
CIMT/G-CSF	311-16-1i	160	2	21,2

CIMT/G-CSF	311-17-1i	160	3	80,5
CIMT/G-CSF	311-17-2i	160	2	20,9
CIMT/G-CSF	311-18-1i	160	1	10,2
CIMT/G-CSF	311-19-1i	160	6	120,1
CIMT/G-CSF	313-15-1i	160	4	55,5
CIMT/G-CSF	313-17-1i	160	1	10,5
CIMT/G-CSF	313-19-1i	160	1	10,8
CIMT/G-CSF	313-19-2i	160	2	39,3
CIMT/G-CSF	314-16-1i	160	1	14,7
CIMT/G-CSF	314-16-2i	160	1	10,6
CIMT/G-CSF	314-17-1i	160	1	17,4
CIMT/G-CSF	314-17-2i	160	1	19,8
CIMT/G-CSF	314-18-1i	160	2	15,9
CIMT/G-CSF	315-11-1i	160	1	19,4
CIMT/G-CSF	315-11-2i	160	1	10,8
CIMT/G-CSF	315-14-1i	160	2	22,1
CIMT/G-CSF	306-17-1i	170	2	31,7
CIMT/G-CSF	306-17-2i	170	1	10,6
CIMT/G-CSF	306-18-1i	170	1	10,5
CIMT/G-CSF	306-18-2i	170	1	10,7
CIMT/G-CSF	306-18-3i	170	2	21,7
CIMT/G-CSF	311-16-1i	170	2	23,6
CIMT/G-CSF	311-17-1i	170	3	39,9
CIMT/G-CSF	311-17-2i	170	2	27,7
CIMT/G-CSF	311-18-1i	170	1	14,4
CIMT/G-CSF	311-19-1i	170	5	100,1
CIMT/G-CSF	313-15-1i	170	3	53,9
CIMT/G-CSF	313-17-1i	170	1	17
CIMT/G-CSF	313-19-1i	170	1	41,1
CIMT/G-CSF	313-19-2i	170	3	35,7
CIMT/G-CSF	314-16-1i	170	0	7
CIMT/G-CSF	314-16-2i	170	0	9,9
CIMT/G-CSF	314-17-1i	170	1	12,8
CIMT/G-CSF	314-17-2i	170	0	6,8
CIMT/G-CSF	314-18-1i	170	3	39,1
CIMT/G-CSF	315-11-1i	170	0	1
CIMT/G-CSF	315-11-2i	170	1	11,7
CIMT/G-CSF	315-14-1i	170	2	21,4
CIMT/G-CSF	306-17-1i	180	2	22,4
CIMT/G-CSF	306-17-2i	180	1	13
CIMT/G-CSF	306-18-1i	180	1	10,8
CIMT/G-CSF	306-18-2i	180	1	10,6
CIMT/G-CSF	306-18-3i	180	1	19,2
CIMT/G-CSF	311-16-1i	180	1	21,1
CIMT/G-CSF	311-17-1i	180	2	26,1
CIMT/G-CSF	311-17-2i	180	2	27,5
CIMT/G-CSF	311-18-1i	180	1	10
CIMT/G-CSF	311-19-1i	180	4	88,9
CIMT/G-CSF	313-15-1i	180	2	28
CIMT/G-CSF	313-17-1i	180	1	12,2
CIMT/G-CSF	313-19-1i	180	1	10,5
CIMT/G-CSF	313-19-2i	180	1	12,3
CIMT/G-CSF	314-17-1i	180	1	12
CIMT/G-CSF	314-18-1i	180	1	33,5
CIMT/G-CSF	315-11-2i	180	1	10,8
CIMT/G-CSF	315-14-1i	180	0	11,3

CIMT/G-CSF	306-17-1i	190	0	9,7
CIMT/G-CSF	306-17-2i	190	1	10,2
CIMT/G-CSF	306-18-1i	190	1	14
CIMT/G-CSF	306-18-2i	190	0	3,3
CIMT/G-CSF	306-18-3i	190	1	13,3
CIMT/G-CSF	311-16-1i	190	0	1,6
CIMT/G-CSF	311-17-1i	190	2	23,9
CIMT/G-CSF	311-17-2i	190	1	20,5
CIMT/G-CSF	311-18-1i	190	0	1,8
CIMT/G-CSF	311-19-1i	190	4	82,4
CIMT/G-CSF	313-15-1i	190	1	16,7
CIMT/G-CSF	313-17-1i	190	1	10,5
CIMT/G-CSF	313-19-1i	190	1	10,2
CIMT/G-CSF	313-19-2i	190	0	0
CIMT/G-CSF	314-17-1i	190	1	21,9
CIMT/G-CSF	314-18-1i	190	0	4,4
CIMT/G-CSF	315-11-2i	190	1	12
CIMT/G-CSF	306-17-2i	200	1	21,3
CIMT/G-CSF	306-18-1i	200	0	34,6
CIMT/G-CSF	306-18-3i	200	0	0,4
CIMT/G-CSF	311-17-1i	200	2	22,9
CIMT/G-CSF	311-17-2i	200	0	10,6
CIMT/G-CSF	311-19-1i	200	5	69,1
CIMT/G-CSF	313-15-1i	200	1	10,3
CIMT/G-CSF	313-17-1i	200	2	17,1
CIMT/G-CSF	313-19-1i	200	1	14,4
CIMT/G-CSF	314-17-1i	200	1	16,2
CIMT/G-CSF	315-11-2i	200	1	10,7
CIMT/G-CSF	306-17-2i	210	0	6,4
CIMT/G-CSF	311-17-1i	210	2	25
CIMT/G-CSF	311-19-1i	210	5	70,6
CIMT/G-CSF	313-15-1i	210	1	14,5
CIMT/G-CSF	313-17-1i	210	2	20,8
CIMT/G-CSF	313-19-1i	210	1	13,4
CIMT/G-CSF	314-17-1i	210	1	12,5
CIMT/G-CSF	315-11-2i	210	0	3,6
CIMT/G-CSF	311-17-1i	220	1	15,6
CIMT/G-CSF	311-19-1i	220	3	48,9
CIMT/G-CSF	313-15-1i	220	1	12,7
CIMT/G-CSF	313-17-1i	220	2	21,9
CIMT/G-CSF	313-19-1i	220	1	10,1
CIMT/G-CSF	314-17-1i	220	0	6,3
CIMT/G-CSF	311-17-1i	230	3	52,1
CIMT/G-CSF	311-19-1i	230	3	43,1
CIMT/G-CSF	313-15-1i	230	1	11,4
CIMT/G-CSF	313-17-1i	230	2	22,4
CIMT/G-CSF	313-19-1i	230	1	10,1
CIMT/G-CSF	311-17-1i	240	1	43
CIMT/G-CSF	311-19-1i	240	3	41,9
CIMT/G-CSF	313-15-1i	240	1	11,3
CIMT/G-CSF	313-17-1i	240	1	20
CIMT/G-CSF	313-19-1i	240	1	11,6
CIMT/G-CSF	311-17-1i	250	1	10,2
CIMT/G-CSF	311-19-1i	250	2	49,7
CIMT/G-CSF	313-15-1i	250	1	37
CIMT/G-CSF	313-17-1i	250	1	10,1

CIMT/G-CSF	313-19-1i	250	1	11,9
CIMT/G-CSF	311-17-1i	260	1	16,6
CIMT/G-CSF	311-19-1i	260	3	28,3
CIMT/G-CSF	313-15-1i	260	1	11
CIMT/G-CSF	313-17-1i	260	1	10,1
CIMT/G-CSF	313-19-1i	260	3	30,7
CIMT/G-CSF	311-17-1i	270	1	10,9
CIMT/G-CSF	311-19-1i	270	2	75,5
CIMT/G-CSF	313-15-1i	270	1	10,2
CIMT/G-CSF	313-17-1i	270	0	5,7
CIMT/G-CSF	313-19-1i	270	3	38,1
CIMT/G-CSF	311-17-1i	280	2	28,5
CIMT/G-CSF	311-19-1i	280	2	22,6
CIMT/G-CSF	313-15-1i	280	1	10,2
CIMT/G-CSF	313-19-1i	280	3	38,4
CIMT/G-CSF	311-17-1i	290	1	24
CIMT/G-CSF	311-19-1i	290	0	17,9
CIMT/G-CSF	313-15-1i	290	1	10,1
CIMT/G-CSF	313-19-1i	290	1	29,6
CIMT/G-CSF	311-17-1i	300	1	20,5
CIMT/G-CSF	313-15-1i	300	1	10,8
CIMT/G-CSF	313-19-1i	300	1	11,2
CIMT/G-CSF	311-17-1i	310	1	14,3
CIMT/G-CSF	313-15-1i	310	1	10,1
CIMT/G-CSF	313-19-1i	310	1	13,7
CIMT/G-CSF	311-17-1i	320	0	0,8
CIMT/G-CSF	313-15-1i	320	0	7,8
CIMT/G-CSF	313-19-1i	320	1	12,8
CIMT/G-CSF	313-19-1i	330	1	11
CIMT/G-CSF	313-19-1i	340	1	10,3
CIMT/G-CSF	313-19-1i	350	1	15,6
CIMT/G-CSF	313-19-1i	360	0	5,8
G-CSF	220-17-1i	150	2	24,3
G-CSF	220-18-1i	150	1	11,2
G-CSF	220-19-1i	150	0	29,9
G-CSF	220-7-1i	150	1	11,5
G-CSF	221-13-1i	150	1	11
G-CSF	221-16-1i	150	2	34,7
G-CSF	221-19-1i	150	5	73,7
G-CSF	221-22-1i	150	1	29,3
G-CSF	221-6-1i	150	1	10,5
G-CSF	222-16-1i	150	1	10,6
G-CSF	222-17-1i	150	1	35,5
G-CSF	222-21-1i	150	2	21,9
G-CSF	222-23-1i	150	1	10,2
G-CSF	222-8-1i	150	1	12,6
G-CSF	301-18-1i	150	1	10,1
G-CSF	301-19-1i	150	2	21,6
G-CSF	301-20-1i	150	1	11,4
G-CSF	301-20-2i	150	1	12,2
G-CSF	301-20-3i	150	4	67,5
G-CSF	302-16-1i	150	1	10,6
G-CSF	302-17-2i	150	1	10,6
G-CSF	302-18-1i	150	1	12,1
G-CSF	303-15-1i	150	2	21,5
G-CSF	303-16-1i	150	3	32,6

G-CSF	303-16-2i	150	1	27
G-CSF	220-17-1i	160	1	17,1
G-CSF	220-18-1i	160	1	10,5
G-CSF	220-7-1i	160	1	10,2
G-CSF	221-13-1i	160	1	14,3
G-CSF	221-16-1i	160	2	22,6
G-CSF	221-19-1i	160	3	55,3
G-CSF	221-22-1i	160	2	21
G-CSF	221-6-1i	160	1	11,2
G-CSF	222-16-1i	160	1	11
G-CSF	222-17-1i	160	1	10,5
G-CSF	222-21-1i	160	2	23,6
G-CSF	222-23-1i	160	1	69
G-CSF	222-8-1i	160	1	11,7
G-CSF	301-18-1i	160	2	31,4
G-CSF	301-19-1i	160	0	16,4
G-CSF	301-20-1i	160	1	10,1
G-CSF	301-20-2i	160	1	10,7
G-CSF	301-20-3i	160	1	32,2
G-CSF	302-16-1i	160	0	7,5
G-CSF	302-17-2i	160	1	10,5
G-CSF	302-18-1i	160	0	2,9
G-CSF	303-15-1i	160	1	12,5
G-CSF	303-16-1i	160	1	23
G-CSF	303-16-2i	160	1	12,4
G-CSF	220-17-1i	170	1	10,2
G-CSF	220-18-1i	170	1	52,2
G-CSF	220-7-1i	170	1	11,1
G-CSF	221-13-1i	170	1	13,1
G-CSF	221-16-1i	170	2	36,3
G-CSF	221-19-1i	170	1	15,8
G-CSF	221-22-1i	170	2	34,5
G-CSF	221-6-1i	170	1	10,7
G-CSF	222-16-1i	170	1	12,3
G-CSF	222-17-1i	170	1	10,7
G-CSF	222-21-1i	170	1	20,6
G-CSF	222-23-1i	170	1	10,3
G-CSF	222-8-1i	170	1	12,1
G-CSF	301-18-1i	170	2	30,1
G-CSF	301-20-1i	170	1	10
G-CSF	301-20-2i	170	1	10,1
G-CSF	301-20-3i	170	1	10,5
G-CSF	302-17-2i	170	0	3,1
G-CSF	303-15-1i	170	0	2,4
G-CSF	303-16-1i	170	1	11,5
G-CSF	303-16-2i	170	1	10,8
G-CSF	220-17-1i	180	2	16,5
G-CSF	220-18-1i	180	1	11,9
G-CSF	220-7-1i	180	0	5,8
G-CSF	221-13-1i	180	0	4,3
G-CSF	221-16-1i	180	1	18,9
G-CSF	221-19-1i	180	1	11
G-CSF	221-22-1i	180	2	21,8
G-CSF	221-6-1i	180	1	10,4
G-CSF	222-16-1i	180	0	19,6
G-CSF	222-17-1i	180	1	10,4

G-CSF	222-21-1i	180	1	11,4
G-CSF	222-23-1i	180	1	13,8
G-CSF	222-8-1i	180	1	14,8
G-CSF	301-18-1i	180	2	23,3
G-CSF	301-20-1i	180	1	10,6
G-CSF	301-20-2i	180	1	10,4
G-CSF	301-20-3i	180	1	12,6
G-CSF	303-16-1i	180	1	12,2
G-CSF	303-16-2i	180	1	14,3
G-CSF	220-17-1i	190	3	45,5
G-CSF	220-18-1i	190	0	4
G-CSF	221-16-1i	190	1	13,3
G-CSF	221-19-1i	190	1	10,2
G-CSF	221-22-1i	190	2	20,6
G-CSF	221-6-1i	190	1	10,3
G-CSF	222-17-1i	190	1	11
G-CSF	222-21-1i	190	1	10,3
G-CSF	222-23-1i	190	1	10,3
G-CSF	222-8-1i	190	0	6
G-CSF	301-18-1i	190	2	45,8
G-CSF	301-20-1i	190	1	11,1
G-CSF	301-20-2i	190	1	12
G-CSF	301-20-3i	190	1	10,7
G-CSF	303-16-1i	190	1	12,1
G-CSF	303-16-2i	190	1	10,6
G-CSF	220-17-1i	200	1	36
G-CSF	221-16-1i	200	1	11,5
G-CSF	221-19-1i	200	1	10,6
G-CSF	221-22-1i	200	2	21,1
G-CSF	221-6-1i	200	1	13,2
G-CSF	222-17-1i	200	1	10,3
G-CSF	222-21-1i	200	0	2,6
G-CSF	222-23-1i	200	2	17
G-CSF	301-18-1i	200	2	23,1
G-CSF	301-20-1i	200	1	10,1
G-CSF	301-20-2i	200	1	10,5
G-CSF	301-20-3i	200	1	10,9
G-CSF	303-16-1i	200	0	0,7
G-CSF	303-16-2i	200	0	0
G-CSF	220-17-1i	210	1	10,9
G-CSF	221-16-1i	210	1	12
G-CSF	221-19-1i	210	0	4,8
G-CSF	221-22-1i	210	1	17,5
G-CSF	221-6-1i	210	0	7,4
G-CSF	222-17-1i	210	1	12,6
G-CSF	222-23-1i	210	2	65,3
G-CSF	301-18-1i	210	1	24,3
G-CSF	301-20-1i	210	1	10,1
G-CSF	301-20-2i	210	1	10,8
G-CSF	301-20-3i	210	1	10,5
G-CSF	220-17-1i	220	1	12,5
G-CSF	221-16-1i	220	1	17,3
G-CSF	221-22-1i	220	1	11,9
G-CSF	222-17-1i	220	0	1,3
G-CSF	222-23-1i	220	2	40,9
G-CSF	301-18-1i	220	1	10,2

G-CSF	301-20-1i	220	1	10,4
G-CSF	301-20-2i	220	0	0,4
G-CSF	301-20-3i	220	0	4,9
G-CSF	220-17-1i	230	1	11,1
G-CSF	221-16-1i	230	1	14,1
G-CSF	221-22-1i	230	1	13,3
G-CSF	222-23-1i	230	2	31,7
G-CSF	301-18-1i	230	1	12
G-CSF	301-20-1i	230	0	2,8
G-CSF	220-17-1i	240	1	11,2
G-CSF	221-16-1i	240	1	23,2
G-CSF	221-22-1i	240	1	10,8
G-CSF	222-23-1i	240	1	24,3
G-CSF	301-18-1i	240	1	11,4
G-CSF	220-17-1i	250	1	10,6
G-CSF	221-16-1i	250	0	18,2
G-CSF	221-22-1i	250	1	13,4
G-CSF	222-23-1i	250	0	2,8
G-CSF	301-18-1i	250	1	10,7
G-CSF	220-17-1i	260	1	10,7
G-CSF	221-22-1i	260	0	13,7
G-CSF	301-18-1i	260	1	13,2
G-CSF	220-17-1i	270	0	5,6
G-CSF	301-18-1i	270	0	9,9

basilar dendrite Sholl 10µm

Group	Number	Radius(µm)	Intersections	Length(µm)
Control	215-11-1i	10	4	4
Control	215-15-1i	10	7	9,1
Control	215-16-1i	10	3	7
Control	215-18-1i	10	5	10,4
Control	215-9-1i	10	3	8,6
Control	216-12-1i	10	6	11,6
Control	216-18-1i	10	4	6,4
Control	216-19-1i	10	2	0,9
Control	216-22-1i	10	2	3,9
Control	216-6-1i	10	0	0
Control	216-8-1i	10	2	4,1
Control	219-10-1i	10	0	0
Control	219-14-1i	10	0	0
Control	219-15-1i	10	0	0
Control	219-17-1i	10	0	0
Control	219-21-1i	10	0	0
Control	297-19-1i	10	0	0
Control	297-19-2i	10	5	8,4
Control	297-20-1i	10	1	2,2
Control	297-22-1i	10	0	0
Control	297-22-2i	10	0	0
Control	298-15-1i	10	2	5,6
Control	298-15-2i	10	1	0,1
Control	298-16-1i	10	0	0
Control	298-17-1i	10	0	0
Control	298-18-1i	10	0	0
Control	308-17-1i	10	0	0

Control	308-18-1i	10	1	0
Control	308-18-2i	10	2	5,4
Control	308-21-1i	10	3	5,3
Control	308-21-2i	10	0	0
CIMT	211-10-1i	10	0	0
CIMT	211-13-1i	10	4	5,9
CIMT	211-13-2i	10	1	4,2
CIMT	211-14-1i	10	1	3,1
CIMT	211-14-2i	10	1	3,1
CIMT	211-15-1i	10	1	0,4
CIMT	211-15-2i	10	3	9,9
CIMT	212-13-1i	10	1	1
CIMT	212-13-2i	10	0	0
CIMT	212-14-1i	10	6	9,1
CIMT	212-14-2i	10	6	9,4
CIMT	212-15-1i	10	5	8,8
CIMT	299-14-1i	10	3	13,1
CIMT	299-14-2i	10	3	2,9
CIMT	299-15-1i	10	4	13,1
CIMT	299-16-1i	10	2	4,5
CIMT	299-17-1i	10	4	3,9
CIMT	300-16-1i	10	0	0
CIMT	300-20-1i	10	5	7,9
CIMT	300-22-1i	10	2	7,1
CIMT	300-24-1i	10	0	0
CIMT	300-24-2i	10	1	3,7
CIMT	309-15-1i	10	1	0,1
CIMT	309-15-2i	10	4	5
CIMT	309-16-1i	10	7	13,2
CIMT	309-16-2i	10	2	5,4
CIMT	309-17-1i	10	2	3,2
CIMT	218-11-1i	10	2	2,9
CIMT	218-14-1i	10	2	1
CIMT	218-16-1i	10	0	0
CIMT	218-17-1i	10	0	0
CIMT	218-19-1i	10	1	1,1
CIMT+G-CSF	213-12-1i	10	6	11,1
CIMT+G-CSF	213-14-1i	10	7	23,7
CIMT+G-CSF	213-17-1i	10	9	18,5
CIMT+G-CSF	213-18-1i	10	8	23,8
CIMT+G-CSF	213-8-1i	10	4	8
CIMT+G-CSF	214-14-1i	10	3	10,9
CIMT+G-CSF	214-14-2i	10	2	2,3
CIMT+G-CSF	214-17-1i	10	1	1
CIMT+G-CSF	214-18-1i	10	4	8,8
CIMT+G-CSF	214-19-1i	10	1	2,1
CIMT+G-CSF	217-10-1i	10	6	17,8
CIMT+G-CSF	217-16-1i	10	0	0
CIMT+G-CSF	217-17-1i	10	0	0
CIMT+G-CSF	217-6-1i	10	2	1,8
CIMT+G-CSF	217-8-1i	10	0	0
CIMT+G-CSF	304-17-1i	10	0	0
CIMT+G-CSF	304-19-1i	10	3	4,6
CIMT+G-CSF	304-19-2i	10	3	12,7
CIMT+G-CSF	304-20-1i	10	0	0
CIMT+G-CSF	304-21-1i	10	3	2,3

CIMT+G-CSF	304-21-2i	10	5	16,7
CIMT+G-CSF	304-22-1i	10	5	17,7
CIMT+G-CSF	305-17-1i	10	4	15,1
CIMT+G-CSF	305-17-2i	10	2	2,1
CIMT+G-CSF	305-18-1i	10	3	4
CIMT+G-CSF	305-19-1i	10	5	15,6
CIMT+G-CSF	305-19-2i	10	0	0
CIMT/G-CSF	306-17-1i	10	0	0
CIMT/G-CSF	306-17-2i	10	7	9
CIMT/G-CSF	306-18-1i	10	4	7,6
CIMT/G-CSF	306-18-2i	10	3	5
CIMT/G-CSF	306-18-3i	10	4	6,1
CIMT/G-CSF	311-16-1i	10	3	12
CIMT/G-CSF	311-17-1i	10	1	0,7
CIMT/G-CSF	311-17-2i	10	2	1,6
CIMT/G-CSF	311-18-1i	10	0	0
CIMT/G-CSF	311-19-1i	10	2	3,3
CIMT/G-CSF	313-14.1i	10	8	33,9
CIMT/G-CSF	313-15-1i	10	0	0
CIMT/G-CSF	313-17-1i	10	1	0,7
CIMT/G-CSF	313-19-1i	10	2	3
CIMT/G-CSF	313-19-2i	10	1	2,7
CIMT/G-CSF	314-16-1i	10	3	2,1
CIMT/G-CSF	314-16-2i	10	2	3,6
CIMT/G-CSF	314-17-1i	10	1	3,6
CIMT/G-CSF	314-17-2i	10	3	3,7
CIMT/G-CSF	314-18-1i	10	3	7,5
CIMT/G-CSF	315-11-1i	10	3	1,7
CIMT/G-CSF	315-11-2i	10	1	3,1
CIMT/G-CSF	315-13-1i	10	6	21,5
CIMT/G-CSF	315-13-2i	10	2	5,4
CIMT/G-CSF	315-14-1i	10	0	0
CIMT/G-CSF	315-14-2i	10	4	11,2
G-CSF	220-16-1i	10	2	2,7
G-CSF	220-17-1i	10	4	8,7
G-CSF	220-18-1i	10	0	0
G-CSF	220-19-1i	10	0	0
G-CSF	220-7-1i	10	0	0
G-CSF	221-13-1i	10	4	9,4
G-CSF	221-16-1i	10	0	0
G-CSF	221-19-1i	10	0	0
G-CSF	221-22-1i	10	0	0
G-CSF	221-6-1i	10	0	0
G-CSF	222-16-1i	10	0	0
G-CSF	222-17-1i	10	2	2,1
G-CSF	222-21-1i	10	0	0
G-CSF	222-23-1i	10	1	0,2
G-CSF	222-8-1i	10	1	0,4
G-CSF	301-18-1i	10	2	7,7
G-CSF	301-19-1i	10	4	7
G-CSF	301-20-1i	10	1	2,6
G-CSF	301-20-2i	10	0	0
G-CSF	301-20-3i	10	1	4,6
G-CSF	302-16-1i	10	4	5,8
G-CSF	302-16-2i	10	5	17
G-CSF	302-17-1i	10	5	18,8

G-CSF	302-17-2i	10	6	17,2
G-CSF	302-18-1i	10	4	5,5
G-CSF	303-15-1i	10	1	2,2
G-CSF	303-16-1i	10	1	0,8
G-CSF	303-16-2i	10	6	12,1
G-CSF	303-17-1i	10	2	5,5
G-CSF	303-17-2i	10	5	9,6

basilar dendrite Sholl 20µm

Group	Number	Radius(µm)	Intersections	Length(µm)
Control	215-11-1i	20	6	84,1
Control	215-15-1i	20	14	165,5
Control	215-16-1i	20	10	98,1
Control	215-18-1i	20	8	134,9
Control	215-9-1i	20	8	104,1
Control	216-12-1i	20	7	93,3
Control	216-18-1i	20	7	97,4
Control	216-19-1i	20	9	82,5
Control	216-22-1i	20	5	70,1
Control	216-6-1i	20	3	13,8
Control	216-8-1i	20	8	92,7
Control	219-10-1i	20	3	49,7
Control	219-14-1i	20	6	43,2
Control	219-15-1i	20	10	97,8
Control	219-17-1i	20	1	0,7
Control	219-21-1i	20	6	58,7
Control	297-19-1i	20	9	73,1
Control	297-19-2i	20	7	82,8
Control	297-20-1i	20	4	42,8
Control	297-22-1i	20	6	66,2
Control	297-22-2i	20	4	33
Control	298-15-1i	20	11	104
Control	298-15-2i	20	3	30,9
Control	298-16-1i	20	0	0
Control	298-17-1i	20	7	65,9
Control	298-18-1i	20	7	76,9
Control	308-17-1i	20	9	79
Control	308-18-1i	20	4	46,9
Control	308-18-2i	20	13	177,7
Control	308-21-1i	20	5	80,8
Control	308-21-2i	20	9	89,4
CIMT	211-10-1i	20	2	10,3
CIMT	211-13-1i	20	8	76,7
CIMT	211-13-2i	20	6	60,1
CIMT	211-14-1i	20	6	62,4
CIMT	211-14-2i	20	10	86,6
CIMT	211-15-1i	20	7	50,1
CIMT	211-15-2i	20	7	89,4
CIMT	212-13-1i	20	4	59,5
CIMT	212-13-2i	20	5	49,5
CIMT	212-14-1i	20	6	67,2
CIMT	212-14-2i	20	9	114,5
CIMT	212-15-1i	20	4	41,1
CIMT	299-14-1i	20	10	115,9

CIMT	299-14-2i	20	10	106,4
CIMT	299-15-1i	20	8	91,9
CIMT	299-16-1i	20	4	48,3
CIMT	299-17-1i	20	6	75,8
CIMT	300-16-1i	20	7	46
CIMT	300-20-1i	20	11	95,3
CIMT	300-22-1i	20	4	59,8
CIMT	300-24-1i	20	3	37,3
CIMT	300-24-2i	20	5	49,4
CIMT	309-15-1i	20	6	68,6
CIMT	309-15-2i	20	11	137,8
CIMT	309-16-1i	20	10	124,3
CIMT	309-16-2i	20	12	97,8
CIMT	309-17-1i	20	11	75
CIMT	218-11-1i	20	8	88,1
CIMT	218-14-1i	20	7	85,4
CIMT	218-16-1i	20	2	4,3
CIMT	218-17-1i	20	5	22,5
CIMT	218-19-1i	20	6	30
CIMT+G-CSF	213-12-1i	20	8	99
CIMT+G-CSF	213-14-1i	20	5	114,7
CIMT+G-CSF	213-17-1i	20	16	170,7
CIMT+G-CSF	213-18-1i	20	11	161,5
CIMT+G-CSF	213-8-1i	20	13	154,6
CIMT+G-CSF	214-14-1i	20	6	74,5
CIMT+G-CSF	214-14-2i	20	5	63,2
CIMT+G-CSF	214-17-1i	20	3	33,7
CIMT+G-CSF	214-18-1i	20	11	91
CIMT+G-CSF	214-19-1i	20	6	75,1
CIMT+G-CSF	217-10-1i	20	9	75,2
CIMT+G-CSF	217-16-1i	20	2	10,6
CIMT+G-CSF	217-17-1i	20	7	48,4
CIMT+G-CSF	217-6-1i	20	6	62,2
CIMT+G-CSF	217-8-1i	20	5	44,8
CIMT+G-CSF	304-17-1i	20	5	54,9
CIMT+G-CSF	304-19-1i	20	5	68,8
CIMT+G-CSF	304-19-2i	20	5	56,9
CIMT+G-CSF	304-20-1i	20	7	40,2
CIMT+G-CSF	304-21-1i	20	9	130,6
CIMT+G-CSF	304-21-2i	20	7	69,4
CIMT+G-CSF	304-22-1i	20	10	108,2
CIMT+G-CSF	305-17-1i	20	11	95,1
CIMT+G-CSF	305-17-2i	20	7	70,5
CIMT+G-CSF	305-18-1i	20	6	59,1
CIMT+G-CSF	305-19-1i	20	7	108,8
CIMT+G-CSF	305-19-2i	20	9	83,3
CIMT/G-CSF	306-17-1i	20	6	74,1
CIMT/G-CSF	306-17-2i	20	13	177,6
CIMT/G-CSF	306-18-1i	20	7	85,3
CIMT/G-CSF	306-18-2i	20	8	88,7
CIMT/G-CSF	306-18-3i	20	7	68,7
CIMT/G-CSF	311-16-1i	20	11	125,2
CIMT/G-CSF	311-17-1i	20	8	65,3
CIMT/G-CSF	311-17-2i	20	7	54,3
CIMT/G-CSF	311-18-1i	20	5	23,3
CIMT/G-CSF	311-19-1i	20	10	112,8

CIMT/G-CSF	313-14.1i	20	11	143,4
CIMT/G-CSF	313-15-1i	20	4	18,1
CIMT/G-CSF	313-17-1i	20	8	55,6
CIMT/G-CSF	313-19-1i	20	7	84,1
CIMT/G-CSF	313-19-2i	20	8	74,4
CIMT/G-CSF	314-16-1i	20	13	155
CIMT/G-CSF	314-16-2i	20	4	37,6
CIMT/G-CSF	314-17-1i	20	5	52
CIMT/G-CSF	314-17-2i	20	5	54,3
CIMT/G-CSF	314-18-1i	20	3	38,5
CIMT/G-CSF	315-11-1i	20	9	97,5
CIMT/G-CSF	315-11-2i	20	7	56,4
CIMT/G-CSF	315-13-1i	20	9	90,2
CIMT/G-CSF	315-13-2i	20	4	41,8
CIMT/G-CSF	315-14-1i	20	7	27
CIMT/G-CSF	315-14-2i	20	5	60,1
G-CSF	220-16-1i	20	7	67,4
G-CSF	220-17-1i	20	8	77,6
G-CSF	220-18-1i	20	3	26,8
G-CSF	220-19-1i	20	3	15,5
G-CSF	220-7-1i	20	1	0,2
G-CSF	221-13-1i	20	9	109,2
G-CSF	221-16-1i	20	5	31,3
G-CSF	221-19-1i	20	8	40,9
G-CSF	221-22-1i	20	1	5,2
G-CSF	221-6-1i	20	5	49
G-CSF	222-16-1i	20	6	44,3
G-CSF	222-17-1i	20	8	60,5
G-CSF	222-21-1i	20	8	44,6
G-CSF	222-23-1i	20	4	27,2
G-CSF	222-8-1i	20	9	65,1
G-CSF	301-18-1i	20	4	55,5
G-CSF	301-19-1i	20	5	69,6
G-CSF	301-20-1i	20	8	72,1
G-CSF	301-20-2i	20	9	78,6
G-CSF	301-20-3i	20	8	92,5
G-CSF	302-16-1i	20	7	81,1
G-CSF	302-16-2i	20	4	56,5
G-CSF	302-17-1i	20	9	117,3
G-CSF	302-17-2i	20	9	97,2
G-CSF	302-18-1i	20	10	112,7
G-CSF	303-15-1i	20	7	89,3
G-CSF	303-16-1i	20	4	21,3
G-CSF	303-16-2i	20	9	122,8
G-CSF	303-17-1i	20	7	83,9
G-CSF	303-17-2i	20	7	88,1

basilar dendrite Sholl 30µm

Group	Number	Radius(µm)	Intersections	Length(µm)
Control	215-11-1i	30	11	159,3
Control	215-15-1i	30	14	209,3
Control	215-16-1i	30	15	224,5
Control	215-18-1i	30	9	104,6
Control	215-9-1i	30	13	200,7

Control	216-12-1i	30	5	77,1
Control	216-18-1i	30	10	132,3
Control	216-19-1i	30	6	121,6
Control	216-22-1i	30	8	77,3
Control	216-6-1i	30	10	118,3
Control	216-8-1i	30	11	153,8
Control	219-10-1i	30	8	81
Control	219-14-1i	30	13	129,7
Control	219-15-1i	30	11	146,5
Control	219-17-1i	30	7	60,5
Control	219-21-1i	30	14	174,1
Control	297-19-1i	30	11	152,1
Control	297-19-2i	30	9	128,2
Control	297-20-1i	30	14	153,8
Control	297-22-1i	30	6	78,6
Control	297-22-2i	30	14	141,6
Control	298-15-1i	30	12	139,9
Control	298-15-2i	30	7	92,2
Control	298-16-1i	30	3	37,2
Control	298-17-1i	30	9	113,4
Control	298-18-1i	30	9	116,7
Control	308-17-1i	30	12	170,1
Control	308-18-1i	30	6	82,9
Control	308-18-2i	30	12	166,2
Control	308-21-1i	30	7	99,1
Control	308-21-2i	30	12	168,7
CIMT	211-10-1i	30	7	92,8
CIMT	211-13-1i	30	12	144,3
CIMT	211-13-2i	30	8	108,4
CIMT	211-14-1i	30	8	132,3
CIMT	211-14-2i	30	8	118
CIMT	211-15-1i	30	8	99,4
CIMT	211-15-2i	30	9	117,4
CIMT	212-13-1i	30	7	72,8
CIMT	212-13-2i	30	7	96
CIMT	212-14-1i	30	7	82,2
CIMT	212-14-2i	30	11	151,4
CIMT	212-15-1i	30	5	59,4
CIMT	299-14-1i	30	15	175,3
CIMT	299-14-2i	30	13	136,8
CIMT	299-15-1i	30	8	94,7
CIMT	299-16-1i	30	6	66
CIMT	299-17-1i	30	3	66
CIMT	300-16-1i	30	10	163,5
CIMT	300-20-1i	30	15	165,4
CIMT	300-22-1i	30	8	117
CIMT	300-24-1i	30	3	32,3
CIMT	300-24-2i	30	9	116,5
CIMT	309-15-1i	30	7	101,4
CIMT	309-15-2i	30	14	172,6
CIMT	309-16-1i	30	10	143,2
CIMT	309-16-2i	30	14	170,1
CIMT	309-17-1i	30	10	164,1
CIMT	218-11-1i	30	11	116,7
CIMT	218-14-1i	30	7	76,7
CIMT	218-16-1i	30	11	82,8

CIMT	218-17-1i	30	13	98,2
CIMT	218-19-1i	30	6	88,1
CIMT+G-CSF	213-12-1i	30	6	124,3
CIMT+G-CSF	213-14-1i	30	5	48,7
CIMT+G-CSF	213-17-1i	30	18	178
CIMT+G-CSF	213-18-1i	30	11	130,9
CIMT+G-CSF	213-8-1i	30	13	149,8
CIMT+G-CSF	214-14-1i	30	12	139,1
CIMT+G-CSF	214-14-2i	30	9	108,9
CIMT+G-CSF	214-17-1i	30	7	81,5
CIMT+G-CSF	214-18-1i	30	12	168
CIMT+G-CSF	214-19-1i	30	8	93,4
CIMT+G-CSF	217-10-1i	30	7	123,8
CIMT+G-CSF	217-16-1i	30	7	73,3
CIMT+G-CSF	217-17-1i	30	10	128,4
CIMT+G-CSF	217-6-1i	30	5	94,5
CIMT+G-CSF	217-8-1i	30	7	94
CIMT+G-CSF	304-17-1i	30	8	86,6
CIMT+G-CSF	304-19-1i	30	6	73,1
CIMT+G-CSF	304-19-2i	30	6	70,9
CIMT+G-CSF	304-20-1i	30	7	136,9
CIMT+G-CSF	304-21-1i	30	11	120,2
CIMT+G-CSF	304-21-2i	30	10	111,1
CIMT+G-CSF	304-22-1i	30	12	160,6
CIMT+G-CSF	305-17-1i	30	9	118,8
CIMT+G-CSF	305-17-2i	30	8	107,5
CIMT+G-CSF	305-18-1i	30	10	107,7
CIMT+G-CSF	305-19-1i	30	11	118,7
CIMT+G-CSF	305-19-2i	30	11	185,4
CIMT/G-CSF	306-17-1i	30	7	104,9
CIMT/G-CSF	306-17-2i	30	17	223,8
CIMT/G-CSF	306-18-1i	30	7	116,9
CIMT/G-CSF	306-18-2i	30	11	139,5
CIMT/G-CSF	306-18-3i	30	8	101,3
CIMT/G-CSF	311-16-1i	30	13	211,3
CIMT/G-CSF	311-17-1i	30	18	190,8
CIMT/G-CSF	311-17-2i	30	11	164,7
CIMT/G-CSF	311-18-1i	30	7	91,1
CIMT/G-CSF	311-19-1i	30	13	152,5
CIMT/G-CSF	313-14.1i	30	10	126,6
CIMT/G-CSF	313-15-1i	30	6	101,7
CIMT/G-CSF	313-17-1i	30	10	154
CIMT/G-CSF	313-19-1i	30	10	158,5
CIMT/G-CSF	313-19-2i	30	10	121,7
CIMT/G-CSF	314-16-1i	30	11	176,7
CIMT/G-CSF	314-16-2i	30	4	45,1
CIMT/G-CSF	314-17-1i	30	8	90,6
CIMT/G-CSF	314-17-2i	30	8	96,7
CIMT/G-CSF	314-18-1i	30	2	37,3
CIMT/G-CSF	315-11-1i	30	10	117,3
CIMT/G-CSF	315-11-2i	30	9	185,4
CIMT/G-CSF	315-13-1i	30	12	157,7
CIMT/G-CSF	315-13-2i	30	7	75,3
CIMT/G-CSF	315-14-1i	30	5	89,6
CIMT/G-CSF	315-14-2i	30	6	83,7
G-CSF	220-16-1i	30	10	127,3

G-CSF	220-17-1i	30	13	150
G-CSF	220-18-1i	30	6	83,1
G-CSF	220-19-1i	30	12	110
G-CSF	220-7-1i	30	5	62,8
G-CSF	221-13-1i	30	9	148,7
G-CSF	221-16-1i	30	17	210,1
G-CSF	221-19-1i	30	17	200,1
G-CSF	221-22-1i	30	8	92,3
G-CSF	221-6-1i	30	9	132,1
G-CSF	222-16-1i	30	7	98,4
G-CSF	222-17-1i	30	14	127,5
G-CSF	222-21-1i	30	9	92,4
G-CSF	222-23-1i	30	10	98,1
G-CSF	222-8-1i	30	16	179,6
G-CSF	301-18-1i	30	4	46,4
G-CSF	301-19-1i	30	5	71,5
G-CSF	301-20-1i	30	9	105,3
G-CSF	301-20-2i	30	9	131,8
G-CSF	301-20-3i	30	11	148,9
G-CSF	302-16-1i	30	8	107,2
G-CSF	302-16-2i	30	4	60,4
G-CSF	302-17-1i	30	5	77,8
G-CSF	302-17-2i	30	9	114,2
G-CSF	302-18-1i	30	11	146,5
G-CSF	303-15-1i	30	7	90,4
G-CSF	303-16-1i	30	8	106,3
G-CSF	303-16-2i	30	9	126
G-CSF	303-17-1i	30	8	100,3
G-CSF	303-17-2i	30	10	125,4

basilar dendrite Sholl 40µm

Group	Number	Radius(µm)	Intersections	Length(µm)
Control	215-11-1i	40	10	168,1
Control	215-15-1i	40	12	171,3
Control	215-16-1i	40	14	207,6
Control	215-18-1i	40	9	101,9
Control	215-9-1i	40	6	123,8
Control	216-12-1i	40	4	66
Control	216-18-1i	40	6	114,2
Control	216-19-1i	40	5	69,2
Control	216-22-1i	40	7	98,8
Control	216-6-1i	40	17	194,6
Control	216-8-1i	40	11	204,2
Control	219-10-1i	40	11	123,2
Control	219-14-1i	40	9	158,3
Control	219-15-1i	40	10	113,6
Control	219-17-1i	40	11	99,8
Control	219-21-1i	40	15	179,2
Control	297-19-1i	40	11	161,3
Control	297-19-2i	40	5	108,4
Control	297-20-1i	40	14	273,5
Control	297-22-1i	40	9	112,8
Control	297-22-2i	40	13	210,6
Control	298-15-1i	40	10	149,7

Control	298-15-2i	40	10	146,5
Control	298-16-1i	40	6	55,1
Control	298-17-1i	40	8	108,4
Control	298-18-1i	40	7	91,4
Control	308-17-1i	40	13	201,9
Control	308-18-1i	40	7	78,9
Control	308-18-2i	40	10	166,8
Control	308-21-1i	40	7	131,2
Control	308-21-2i	40	6	128
CIMT	211-10-1i	40	14	185,6
CIMT	211-13-1i	40	12	173,6
CIMT	211-13-2i	40	8	98,2
CIMT	211-14-1i	40	9	129,4
CIMT	211-14-2i	40	7	131,7
CIMT	211-15-1i	40	8	93,9
CIMT	211-15-2i	40	9	111
CIMT	212-13-1i	40	7	101,3
CIMT	212-13-2i	40	5	75,3
CIMT	212-14-1i	40	6	64,9
CIMT	212-14-2i	40	9	140
CIMT	212-15-1i	40	6	77,4
CIMT	299-14-1i	40	10	175,5
CIMT	299-14-2i	40	13	184
CIMT	299-15-1i	40	8	101,1
CIMT	299-16-1i	40	6	81,3
CIMT	299-17-1i	40	3	31,7
CIMT	300-16-1i	40	12	135
CIMT	300-20-1i	40	15	195,6
CIMT	300-22-1i	40	11	128,2
CIMT	300-24-1i	40	5	33,7
CIMT	300-24-2i	40	13	177,1
CIMT	309-15-1i	40	7	89,2
CIMT	309-15-2i	40	11	177,9
CIMT	309-16-1i	40	8	131,9
CIMT	309-16-2i	40	16	183,2
CIMT	309-17-1i	40	10	136,7
CIMT	218-11-1i	40	11	142,5
CIMT	218-14-1i	40	8	94
CIMT	218-16-1i	40	9	173,4
CIMT	218-17-1i	40	10	197,1
CIMT	218-19-1i	40	7	85,3
CIMT+G-CSF	213-12-1i	40	4	70
CIMT+G-CSF	213-14-1i	40	2	56,7
CIMT+G-CSF	213-17-1i	40	14	203,3
CIMT+G-CSF	213-18-1i	40	11	142
CIMT+G-CSF	213-8-1i	40	11	154,2
CIMT+G-CSF	214-14-1i	40	10	139,1
CIMT+G-CSF	214-14-2i	40	9	121,9
CIMT+G-CSF	214-17-1i	40	6	105,5
CIMT+G-CSF	214-18-1i	40	10	147,9
CIMT+G-CSF	214-19-1i	40	7	102,3
CIMT+G-CSF	217-10-1i	40	9	101,8
CIMT+G-CSF	217-16-1i	40	13	175,1
CIMT+G-CSF	217-17-1i	40	13	147
CIMT+G-CSF	217-6-1i	40	8	96,6
CIMT+G-CSF	217-8-1i	40	8	113,8

CIMT+G-CSF	304-17-1i	40	4	94,2
CIMT+G-CSF	304-19-1i	40	5	81,7
CIMT+G-CSF	304-19-2i	40	6	69
CIMT+G-CSF	304-20-1i	40	4	61,8
CIMT+G-CSF	304-21-1i	40	14	154,9
CIMT+G-CSF	304-21-2i	40	7	102,3
CIMT+G-CSF	304-22-1i	40	9	141
CIMT+G-CSF	305-17-1i	40	8	116,1
CIMT+G-CSF	305-17-2i	40	10	132,4
CIMT+G-CSF	305-18-1i	40	6	120,3
CIMT+G-CSF	305-19-1i	40	9	131,9
CIMT+G-CSF	305-19-2i	40	7	142,9
CIMT/G-CSF	306-17-1i	40	7	101
CIMT/G-CSF	306-17-2i	40	15	214,8
CIMT/G-CSF	306-18-1i	40	8	109,3
CIMT/G-CSF	306-18-2i	40	13	211,6
CIMT/G-CSF	306-18-3i	40	9	120,6
CIMT/G-CSF	311-16-1i	40	11	161,6
CIMT/G-CSF	311-17-1i	40	20	236,9
CIMT/G-CSF	311-17-2i	40	14	224,7
CIMT/G-CSF	311-18-1i	40	7	95,6
CIMT/G-CSF	311-19-1i	40	18	219,1
CIMT/G-CSF	313-14.1i	40	10	130
CIMT/G-CSF	313-15-1i	40	5	77,6
CIMT/G-CSF	313-17-1i	40	9	134,2
CIMT/G-CSF	313-19-1i	40	7	99,8
CIMT/G-CSF	313-19-2i	40	8	179,4
CIMT/G-CSF	314-16-1i	40	13	167,5
CIMT/G-CSF	314-16-2i	40	5	59,4
CIMT/G-CSF	314-17-1i	40	12	164,4
CIMT/G-CSF	314-17-2i	40	11	135,8
CIMT/G-CSF	314-18-1i	40	2	32,2
CIMT/G-CSF	315-11-1i	40	7	145,1
CIMT/G-CSF	315-11-2i	40	8	143,3
CIMT/G-CSF	315-13-1i	40	13	182,3
CIMT/G-CSF	315-13-2i	40	7	88,5
CIMT/G-CSF	315-14-1i	40	1	72,3
CIMT/G-CSF	315-14-2i	40	7	76,9
G-CSF	220-16-1i	40	9	134,1
G-CSF	220-17-1i	40	15	187,5
G-CSF	220-18-1i	40	23	246,8
G-CSF	220-19-1i	40	14	194,1
G-CSF	220-7-1i	40	11	126,5
G-CSF	221-13-1i	40	10	115,5
G-CSF	221-16-1i	40	19	305
G-CSF	221-19-1i	40	18	221,1
G-CSF	221-22-1i	40	9	163,5
G-CSF	221-6-1i	40	12	170
G-CSF	222-16-1i	40	10	95,6
G-CSF	222-17-1i	40	15	188,5
G-CSF	222-21-1i	40	11	138,8
G-CSF	222-23-1i	40	13	200,5
G-CSF	222-8-1i	40	18	250
G-CSF	301-18-1i	40	4	52,2
G-CSF	301-19-1i	40	5	76,1
G-CSF	301-20-1i	40	7	112,5

G-CSF	301-20-2i	40	14	147,5
G-CSF	301-20-3i	40	14	203
G-CSF	302-16-1i	40	4	54,3
G-CSF	302-16-2i	40	2	42,3
G-CSF	302-17-1i	40	3	48,3
G-CSF	302-17-2i	40	9	114
G-CSF	302-18-1i	40	9	123,1
G-CSF	303-15-1i	40	7	95,7
G-CSF	303-16-1i	40	12	165,5
G-CSF	303-16-2i	40	8	110,2
G-CSF	303-17-1i	40	9	107,5
G-CSF	303-17-2i	40	8	105,8

basilar dendrite Sholl 50µm

Group	Number	Radius(µm)	Intersections	Length(µm)
Control	215-11-1i	50	7	103,5
Control	215-15-1i	50	11	162,5
Control	215-16-1i	50	9	136
Control	215-18-1i	50	8	100,4
Control	215-9-1i	50	5	78,4
Control	216-12-1i	50	2	35,8
Control	216-18-1i	50	2	34,3
Control	216-19-1i	50	1	55
Control	216-22-1i	50	8	102,3
Control	216-6-1i	50	20	266,4
Control	216-8-1i	50	9	145,7
Control	219-10-1i	50	9	118,5
Control	219-14-1i	50	9	125,7
Control	219-15-1i	50	9	106,6
Control	219-17-1i	50	10	158,8
Control	219-21-1i	50	13	196,7
Control	297-19-1i	50	10	130,5
Control	297-19-2i	50	5	57,2
Control	297-20-1i	50	14	222,5
Control	297-22-1i	50	8	116,5
Control	297-22-2i	50	10	171,8
Control	298-15-1i	50	11	125,3
Control	298-15-2i	50	8	125,7
Control	298-16-1i	50	5	108,3
Control	298-17-1i	50	7	92,2
Control	298-18-1i	50	5	66,7
Control	308-17-1i	50	13	239
Control	308-18-1i	50	19	181,7
Control	308-18-2i	50	8	121,2
Control	308-21-1i	50	6	94
Control	308-21-2i	50	5	74
CIMT	211-10-1i	50	19	234,3
CIMT	211-13-1i	50	10	146,2
CIMT	211-13-2i	50	7	100,7
CIMT	211-14-1i	50	8	119,1
CIMT	211-14-2i	50	5	78,1
CIMT	211-15-1i	50	7	95,4
CIMT	211-15-2i	50	8	91,3
CIMT	212-13-1i	50	5	88,7

CIMT	212-13-2i	50	6	68,9
CIMT	212-14-1i	50	6	77,6
CIMT	212-14-2i	50	10	120,5
CIMT	212-15-1i	50	4	68,8
CIMT	299-14-1i	50	10	130,1
CIMT	299-14-2i	50	9	161,3
CIMT	299-15-1i	50	8	87,8
CIMT	299-16-1i	50	6	78,1
CIMT	299-17-1i	50	3	33,2
CIMT	300-16-1i	50	12	144,8
CIMT	300-20-1i	50	13	184,5
CIMT	300-22-1i	50	14	171,6
CIMT	300-24-1i	50	7	140,3
CIMT	300-24-2i	50	12	150,4
CIMT	309-15-1i	50	7	99
CIMT	309-15-2i	50	9	125,1
CIMT	309-16-1i	50	4	78,2
CIMT	309-16-2i	50	15	195,5
CIMT	309-17-1i	50	9	121,6
CIMT	218-11-1i	50	8	129,9
CIMT	218-14-1i	50	12	138,2
CIMT	218-16-1i	50	11	154,4
CIMT	218-17-1i	50	7	190,2
CIMT	218-19-1i	50	8	111,4
CIMT+G-CSF	213-12-1i	50	4	52,5
CIMT+G-CSF	213-14-1i	50	1	25
CIMT+G-CSF	213-17-1i	50	13	164,6
CIMT+G-CSF	213-18-1i	50	8	123,4
CIMT+G-CSF	213-8-1i	50	7	116,1
CIMT+G-CSF	214-14-1i	50	8	116,2
CIMT+G-CSF	214-14-2i	50	8	120,3
CIMT+G-CSF	214-17-1i	50	6	72,8
CIMT+G-CSF	214-18-1i	50	9	116,8
CIMT+G-CSF	214-19-1i	50	4	57,3
CIMT+G-CSF	217-10-1i	50	6	76,2
CIMT+G-CSF	217-16-1i	50	17	236,4
CIMT+G-CSF	217-17-1i	50	17	214,6
CIMT+G-CSF	217-6-1i	50	11	130,9
CIMT+G-CSF	217-8-1i	50	9	132,8
CIMT+G-CSF	304-17-1i	50	4	46,8
CIMT+G-CSF	304-19-1i	50	4	48,6
CIMT+G-CSF	304-19-2i	50	7	80
CIMT+G-CSF	304-20-1i	50	5	61
CIMT+G-CSF	304-21-1i	50	12	154,3
CIMT+G-CSF	304-21-2i	50	2	56,5
CIMT+G-CSF	304-22-1i	50	7	104,3
CIMT+G-CSF	305-17-1i	50	8	122,8
CIMT+G-CSF	305-17-2i	50	8	130,1
CIMT+G-CSF	305-18-1i	50	1	44
CIMT+G-CSF	305-19-1i	50	6	93,8
CIMT+G-CSF	305-19-2i	50	6	89,6
CIMT/G-CSF	306-17-1i	50	6	81,3
CIMT/G-CSF	306-17-2i	50	12	189,1
CIMT/G-CSF	306-18-1i	50	7	100,6
CIMT/G-CSF	306-18-2i	50	11	178,3
CIMT/G-CSF	306-18-3i	50	9	116,3

CIMT/G-CSF	311-16-1i	50	10	139,9
CIMT/G-CSF	311-17-1i	50	25	289,8
CIMT/G-CSF	311-17-2i	50	17	222,4
CIMT/G-CSF	311-18-1i	50	6	77,2
CIMT/G-CSF	311-19-1i	50	18	262,4
CIMT/G-CSF	313-14.1i	50	9	137,6
CIMT/G-CSF	313-15-1i	50	6	82,6
CIMT/G-CSF	313-17-1i	50	7	122
CIMT/G-CSF	313-19-1i	50	5	107,1
CIMT/G-CSF	313-19-2i	50	9	125,6
CIMT/G-CSF	314-16-1i	50	14	217,9
CIMT/G-CSF	314-16-2i	50	6	60,4
CIMT/G-CSF	314-17-1i	50	15	233,8
CIMT/G-CSF	314-17-2i	50	10	157
CIMT/G-CSF	314-18-1i	50	1	13,7
CIMT/G-CSF	315-11-1i	50	5	79,1
CIMT/G-CSF	315-11-2i	50	7	109,6
CIMT/G-CSF	315-13-1i	50	15	173,7
CIMT/G-CSF	315-13-2i	50	7	80,7
CIMT/G-CSF	315-14-1i	50	1	10,5
CIMT/G-CSF	315-14-2i	50	7	79,4
G-CSF	220-16-1i	50	8	105,7
G-CSF	220-17-1i	50	9	137,3
G-CSF	220-18-1i	50	16	306,7
G-CSF	220-19-1i	50	12	187,9
G-CSF	220-7-1i	50	14	200,8
G-CSF	221-13-1i	50	9	109,6
G-CSF	221-16-1i	50	17	257,3
G-CSF	221-19-1i	50	21	248,9
G-CSF	221-22-1i	50	11	163,8
G-CSF	221-6-1i	50	10	144,2
G-CSF	222-16-1i	50	9	135,1
G-CSF	222-17-1i	50	16	216,2
G-CSF	222-21-1i	50	14	169
G-CSF	222-23-1i	50	16	224,5
G-CSF	222-8-1i	50	23	277,3
G-CSF	301-18-1i	50	3	50,4
G-CSF	301-19-1i	50	4	62,2
G-CSF	301-20-1i	50	7	88,1
G-CSF	301-20-2i	50	12	166,2
G-CSF	301-20-3i	50	16	191,3
G-CSF	302-16-1i	50	4	46,3
G-CSF	302-16-2i	50	0	11,2
G-CSF	302-17-1i	50	1	26
G-CSF	302-17-2i	50	6	95,2
G-CSF	302-18-1i	50	6	101,5
G-CSF	303-15-1i	50	8	99,4
G-CSF	303-16-1i	50	15	193,1
G-CSF	303-16-2i	50	9	96,1
G-CSF	303-17-1i	50	9	105,2
G-CSF	303-17-2i	50	7	89,3

basilar dendrite Sholl 60µm

Group	Number	Radius(µm)	Intersections	Length(µm)
Control	215-11-1i	60	6	97,6
Control	215-15-1i	60	7	119,7
Control	215-16-1i	60	8	111
Control	215-18-1i	60	8	91,8
Control	215-9-1i	60	3	66,3
Control	216-12-1i	60	0	19,2
Control	216-18-1i	60	1	11,4
Control	216-19-1i	60	1	12
Control	216-22-1i	60	7	103,6
Control	216-6-1i	60	14	315,5
Control	216-8-1i	60	10	123,1
Control	219-10-1i	60	9	107,3
Control	219-14-1i	60	9	115
Control	219-15-1i	60	8	97,1
Control	219-17-1i	60	10	147,3
Control	219-21-1i	60	12	170,1
Control	297-19-1i	60	13	161,8
Control	297-19-2i	60	4	61,6
Control	297-20-1i	60	12	171,4
Control	297-22-1i	60	6	98,4
Control	297-22-2i	60	10	134,6
Control	298-15-1i	60	8	130,4
Control	298-15-2i	60	7	88,5
Control	298-16-1i	60	2	44,3
Control	298-17-1i	60	9	95,8
Control	298-18-1i	60	6	66,3
Control	308-17-1i	60	10	207,3
Control	308-18-1i	60	11	234,8
Control	308-18-2i	60	8	92,5
Control	308-21-1i	60	5	86,8
Control	308-21-2i	60	4	53,2
CIMT	211-10-1i	60	15	243,9
CIMT	211-13-1i	60	8	100,5
CIMT	211-13-2i	60	5	79,5
CIMT	211-14-1i	60	6	84,7
CIMT	211-14-2i	60	4	58,4
CIMT	211-15-1i	60	6	82,1
CIMT	211-15-2i	60	7	84,2
CIMT	212-13-1i	60	4	54,3
CIMT	212-13-2i	60	6	71
CIMT	212-14-1i	60	3	68,7
CIMT	212-14-2i	60	9	116,6
CIMT	212-15-1i	60	3	36
CIMT	299-14-1i	60	7	106,5
CIMT	299-14-2i	60	7	107
CIMT	299-15-1i	60	7	92,4
CIMT	299-16-1i	60	5	73,5
CIMT	299-17-1i	60	2	31
CIMT	300-16-1i	60	9	123,9
CIMT	300-20-1i	60	11	150,7
CIMT	300-22-1i	60	10	150,7
CIMT	300-24-1i	60	9	136,9
CIMT	300-24-2i	60	10	123,2

CIMT	309-15-1i	60	7	90,7
CIMT	309-15-2i	60	7	101,3
CIMT	309-16-1i	60	4	56
CIMT	309-16-2i	60	15	186,1
CIMT	309-17-1i	60	10	108,5
CIMT	218-11-1i	60	7	81,8
CIMT	218-14-1i	60	10	128,7
CIMT	218-16-1i	60	11	120,3
CIMT	218-17-1i	60	7	118,5
CIMT	218-19-1i	60	8	102,4
CIMT+G-CSF	213-12-1i	60	0	29,6
CIMT+G-CSF	213-14-1i	60	0	11,9
CIMT+G-CSF	213-17-1i	60	5	132,6
CIMT+G-CSF	213-18-1i	60	5	96,3
CIMT+G-CSF	213-8-1i	60	7	87,2
CIMT+G-CSF	214-14-1i	60	4	59
CIMT+G-CSF	214-14-2i	60	3	49,7
CIMT+G-CSF	214-17-1i	60	6	75,3
CIMT+G-CSF	214-18-1i	60	6	100
CIMT+G-CSF	214-19-1i	60	5	67
CIMT+G-CSF	217-10-1i	60	4	59,3
CIMT+G-CSF	217-16-1i	60	15	211,3
CIMT+G-CSF	217-17-1i	60	20	255,1
CIMT+G-CSF	217-6-1i	60	10	138,3
CIMT+G-CSF	217-8-1i	60	11	152
CIMT+G-CSF	304-17-1i	60	2	39,5
CIMT+G-CSF	304-19-1i	60	2	23,1
CIMT+G-CSF	304-19-2i	60	5	66
CIMT+G-CSF	304-20-1i	60	3	46,4
CIMT+G-CSF	304-21-1i	60	11	180,9
CIMT+G-CSF	304-21-2i	60	1	20,3
CIMT+G-CSF	304-22-1i	60	5	64,1
CIMT+G-CSF	305-17-1i	60	4	88,5
CIMT+G-CSF	305-17-2i	60	7	96,1
CIMT+G-CSF	305-18-1i	60	1	10,8
CIMT+G-CSF	305-19-1i	60	5	71,3
CIMT+G-CSF	305-19-2i	60	5	77,4
CIMT/G-CSF	306-17-1i	60	3	63,3
CIMT/G-CSF	306-17-2i	60	9	159,5
CIMT/G-CSF	306-18-1i	60	6	92,8
CIMT/G-CSF	306-18-2i	60	8	111
CIMT/G-CSF	306-18-3i	60	9	105,5
CIMT/G-CSF	311-16-1i	60	9	117,3
CIMT/G-CSF	311-17-1i	60	24	310,1
CIMT/G-CSF	311-17-2i	60	17	217,6
CIMT/G-CSF	311-18-1i	60	5	63,6
CIMT/G-CSF	311-19-1i	60	16	202,7
CIMT/G-CSF	313-14.1i	60	6	95,6
CIMT/G-CSF	313-15-1i	60	6	85,4
CIMT/G-CSF	313-17-1i	60	5	99,9
CIMT/G-CSF	313-19-1i	60	2	41
CIMT/G-CSF	313-19-2i	60	10	139,5
CIMT/G-CSF	314-16-1i	60	14	174,3
CIMT/G-CSF	314-16-2i	60	5	79,3
CIMT/G-CSF	314-17-1i	60	12	147,5
CIMT/G-CSF	314-17-2i	60	8	128,5

CIMT/G-CSF	314-18-1i	60	1	11,8
CIMT/G-CSF	315-11-1i	60	5	64,1
CIMT/G-CSF	315-11-2i	60	5	88,3
CIMT/G-CSF	315-13-1i	60	16	182,8
CIMT/G-CSF	315-13-2i	60	5	71,4
CIMT/G-CSF	315-14-1i	60	1	11,9
CIMT/G-CSF	315-14-2i	60	6	77,5
G-CSF	220-16-1i	60	5	63,2
G-CSF	220-17-1i	60	8	104,7
G-CSF	220-18-1i	60	11	189,1
G-CSF	220-19-1i	60	12	186,1
G-CSF	220-7-1i	60	18	227,9
G-CSF	221-13-1i	60	8	89,2
G-CSF	221-16-1i	60	21	247
G-CSF	221-19-1i	60	19	264
G-CSF	221-22-1i	60	11	175,3
G-CSF	221-6-1i	60	11	144,4
G-CSF	222-16-1i	60	8	104
G-CSF	222-17-1i	60	16	246
G-CSF	222-21-1i	60	17	183,6
G-CSF	222-23-1i	60	14	200,4
G-CSF	222-8-1i	60	21	253,2
G-CSF	301-18-1i	60	2	31,5
G-CSF	301-19-1i	60	3	46,6
G-CSF	301-20-1i	60	6	75,1
G-CSF	301-20-2i	60	12	142,7
G-CSF	301-20-3i	60	16	199
G-CSF	302-16-1i	60	3	39,7
G-CSF	302-17-1i	60	1	12,6
G-CSF	302-17-2i	60	6	86,8
G-CSF	302-18-1i	60	3	52,9
G-CSF	303-15-1i	60	8	104,7
G-CSF	303-16-1i	60	16	221,8
G-CSF	303-16-2i	60	9	140,4
G-CSF	303-17-1i	60	10	126,5
G-CSF	303-17-2i	60	6	85,6

basilar dendrite Sholl 70µm

Group	Number	Radius(µm)	Intersections	Length(µm)
Control	215-11-1i	70	4	72,4
Control	215-15-1i	70	4	55,1
Control	215-16-1i	70	5	86
Control	215-18-1i	70	8	89,7
Control	215-9-1i	70	3	32,3
Control	216-18-1i	70	0	6,1
Control	216-19-1i	70	0	7
Control	216-22-1i	70	4	49,9
Control	216-6-1i	70	13	180,8
Control	216-8-1i	70	7	109,5
Control	219-10-1i	70	9	114,6
Control	219-14-1i	70	12	128,6
Control	219-15-1i	70	7	90,1
Control	219-17-1i	70	7	107,4
Control	219-21-1i	70	12	139,1

Control	297-19-1i	70	9	168,1
Control	297-19-2i	70	2	37,2
Control	297-20-1i	70	13	177,1
Control	297-22-1i	70	2	55,1
Control	297-22-2i	70	8	117,5
Control	298-15-1i	70	8	123,3
Control	298-15-2i	70	3	58,3
Control	298-16-1i	70	1	22,5
Control	298-17-1i	70	7	98,7
Control	298-18-1i	70	6	64,7
Control	308-17-1i	70	9	114
Control	308-18-1i	70	11	154
Control	308-18-2i	70	7	106,7
Control	308-21-1i	70	2	64,9
Control	308-21-2i	70	4	72,8
CIMT	211-10-1i	70	13	232
CIMT	211-13-1i	70	8	102,9
CIMT	211-13-2i	70	4	79,2
CIMT	211-14-1i	70	6	94,6
CIMT	211-14-2i	70	3	51,8
CIMT	211-15-1i	70	6	67,2
CIMT	211-15-2i	70	6	76,5
CIMT	212-13-1i	70	3	60
CIMT	212-13-2i	70	5	58,2
CIMT	212-14-1i	70	2	33,8
CIMT	212-14-2i	70	8	101,3
CIMT	212-15-1i	70	3	32,8
CIMT	299-14-1i	70	4	72,9
CIMT	299-14-2i	70	5	90,7
CIMT	299-15-1i	70	8	97
CIMT	299-16-1i	70	3	62,3
CIMT	299-17-1i	70	1	22,8
CIMT	300-16-1i	70	7	104,6
CIMT	300-20-1i	70	10	124,4
CIMT	300-22-1i	70	11	126
CIMT	300-24-1i	70	7	148
CIMT	300-24-2i	70	8	130
CIMT	309-15-1i	70	6	83,1
CIMT	309-15-2i	70	5	89,5
CIMT	309-16-1i	70	3	43,4
CIMT	309-16-2i	70	15	174,3
CIMT	309-17-1i	70	8	101,2
CIMT	218-11-1i	70	6	88,9
CIMT	218-14-1i	70	7	112,5
CIMT	218-16-1i	70	10	150,9
CIMT	218-17-1i	70	5	98,1
CIMT	218-19-1i	70	9	113,2
CIMT+G-CSF	213-17-1i	70	6	66,1
CIMT+G-CSF	213-18-1i	70	4	65,7
CIMT+G-CSF	213-8-1i	70	6	76,6
CIMT+G-CSF	214-14-1i	70	2	40,9
CIMT+G-CSF	214-14-2i	70	3	44,5
CIMT+G-CSF	214-17-1i	70	6	69,1
CIMT+G-CSF	214-18-1i	70	5	67,2
CIMT+G-CSF	214-19-1i	70	5	70,8
CIMT+G-CSF	217-10-1i	70	4	51,8

CIMT+G-CSF	217-16-1i	70	17	176,3
CIMT+G-CSF	217-17-1i	70	21	241,7
CIMT+G-CSF	217-6-1i	70	8	116,5
CIMT+G-CSF	217-8-1i	70	12	166,4
CIMT+G-CSF	304-17-1i	70	4	47,3
CIMT+G-CSF	304-19-1i	70	2	25,2
CIMT+G-CSF	304-19-2i	70	3	61,2
CIMT+G-CSF	304-20-1i	70	3	31,4
CIMT+G-CSF	304-21-1i	70	11	139,8
CIMT+G-CSF	304-21-2i	70	1	10,8
CIMT+G-CSF	304-22-1i	70	2	33,7
CIMT+G-CSF	305-17-1i	70	2	37
CIMT+G-CSF	305-17-2i	70	7	81,9
CIMT+G-CSF	305-18-1i	70	1	11,2
CIMT+G-CSF	305-19-1i	70	3	37,1
CIMT+G-CSF	305-19-2i	70	3	47,6
CIMT/G-CSF	306-17-1i	70	3	45,8
CIMT/G-CSF	306-17-2i	70	6	80,7
CIMT/G-CSF	306-18-1i	70	4	62,7
CIMT/G-CSF	306-18-2i	70	5	73,3
CIMT/G-CSF	306-18-3i	70	6	81,9
CIMT/G-CSF	311-16-1i	70	8	119,4
CIMT/G-CSF	311-17-1i	70	25	321
CIMT/G-CSF	311-17-2i	70	17	218,3
CIMT/G-CSF	311-18-1i	70	8	92,4
CIMT/G-CSF	311-19-1i	70	16	204
CIMT/G-CSF	313-14.1i	70	5	69,8
CIMT/G-CSF	313-15-1i	70	7	103,8
CIMT/G-CSF	313-17-1i	70	5	55,8
CIMT/G-CSF	313-19-1i	70	1	11,9
CIMT/G-CSF	313-19-2i	70	7	137,4
CIMT/G-CSF	314-16-1i	70	16	187
CIMT/G-CSF	314-16-2i	70	5	57,9
CIMT/G-CSF	314-17-1i	70	10	138,7
CIMT/G-CSF	314-17-2i	70	7	88,4
CIMT/G-CSF	314-18-1i	70	1	16,6
CIMT/G-CSF	315-11-1i	70	3	54,9
CIMT/G-CSF	315-11-2i	70	3	47,1
CIMT/G-CSF	315-13-1i	70	13	171,3
CIMT/G-CSF	315-13-2i	70	6	84,6
CIMT/G-CSF	315-14-1i	70	0	2,6
CIMT/G-CSF	315-14-2i	70	5	69,8
G-CSF	220-16-1i	70	5	53,8
G-CSF	220-17-1i	70	7	107,2
G-CSF	220-18-1i	70	9	128,9
G-CSF	220-19-1i	70	11	147,1
G-CSF	220-7-1i	70	17	217,4
G-CSF	221-13-1i	70	9	97,9
G-CSF	221-16-1i	70	23	259,9
G-CSF	221-19-1i	70	20	229
G-CSF	221-22-1i	70	10	135,4
G-CSF	221-6-1i	70	9	121,8
G-CSF	222-16-1i	70	7	105,5
G-CSF	222-17-1i	70	15	185,1
G-CSF	222-21-1i	70	12	164,3
G-CSF	222-23-1i	70	10	144,7

G-CSF	222-8-1i	70	17	226,8
G-CSF	301-18-1i	70	2	23
G-CSF	301-19-1i	70	2	22
G-CSF	301-20-1i	70	6	69,1
G-CSF	301-20-2i	70	12	133,4
G-CSF	301-20-3i	70	13	179,6
G-CSF	302-16-1i	70	1	21,1
G-CSF	302-17-1i	70	0	1,1
G-CSF	302-17-2i	70	2	71,7
G-CSF	302-18-1i	70	2	31,5
G-CSF	303-15-1i	70	8	95,5
G-CSF	303-16-1i	70	13	209,8
G-CSF	303-16-2i	70	9	116,2
G-CSF	303-17-1i	70	9	119,2
G-CSF	303-17-2i	70	5	73,1

basilar dendrite Sholl 80µm

Group	Number	Radius(µm)	Intersections	Length(µm)
Control	215-11-1i	80	3	52,5
Control	215-15-1i	80	2	37,8
Control	215-16-1i	80	3	58,1
Control	215-18-1i	80	6	95,3
Control	215-9-1i	80	2	34,7
Control	216-22-1i	80	4	44,1
Control	216-6-1i	80	12	165,1
Control	216-8-1i	80	6	97,6
Control	219-10-1i	80	8	95,9
Control	219-14-1i	80	9	117
Control	219-15-1i	80	4	71,8
Control	219-17-1i	80	8	84,5
Control	219-21-1i	80	12	129,9
Control	297-19-1i	80	6	136,5
Control	297-19-2i	80	1	16,6
Control	297-20-1i	80	9	177,2
Control	297-22-1i	80	1	16,4
Control	297-22-2i	80	6	80,5
Control	298-15-1i	80	7	99,3
Control	298-15-2i	80	2	28,1
Control	298-16-1i	80	1	15,5
Control	298-17-1i	80	7	89,3
Control	298-18-1i	80	5	89,5
Control	308-17-1i	80	6	94,9
Control	308-18-1i	80	8	120,7
Control	308-18-2i	80	3	44,7
Control	308-21-1i	80	1	20,5
Control	308-21-2i	80	6	66
CIMT	211-10-1i	80	17	249,3
CIMT	211-13-1i	80	6	73,3
CIMT	211-13-2i	80	4	43,9
CIMT	211-14-1i	80	6	94,5
CIMT	211-14-2i	80	1	22,5
CIMT	211-15-1i	80	3	60,4
CIMT	211-15-2i	80	6	71,4
CIMT	212-13-1i	80	3	40,1

CIMT	212-13-2i	80	4	52,3
CIMT	212-14-1i	80	1	49,4
CIMT	212-14-2i	80	6	83,3
CIMT	212-15-1i	80	3	33,9
CIMT	299-14-1i	80	1	34,8
CIMT	299-14-2i	80	3	53,7
CIMT	299-15-1i	80	8	91,7
CIMT	299-16-1i	80	3	35,8
CIMT	299-17-1i	80	1	11,5
CIMT	300-16-1i	80	3	49,6
CIMT	300-20-1i	80	9	109,8
CIMT	300-22-1i	80	10	128,7
CIMT	300-24-1i	80	8	154,3
CIMT	300-24-2i	80	7	85,3
CIMT	309-15-1i	80	6	72,7
CIMT	309-15-2i	80	4	53,4
CIMT	309-16-1i	80	2	33,3
CIMT	309-16-2i	80	15	182,5
CIMT	309-17-1i	80	8	95,5
CIMT	218-11-1i	80	3	67,4
CIMT	218-14-1i	80	5	74,4
CIMT	218-16-1i	80	11	133,3
CIMT	218-17-1i	80	5	84,5
CIMT	218-19-1i	80	10	131,3
CIMT+G-CSF	213-17-1i	80	5	62,5
CIMT+G-CSF	213-18-1i	80	5	52,9
CIMT+G-CSF	213-8-1i	80	5	66,2
CIMT+G-CSF	214-14-1i	80	1	23,6
CIMT+G-CSF	214-14-2i	80	2	26,7
CIMT+G-CSF	214-17-1i	80	4	66,1
CIMT+G-CSF	214-18-1i	80	4	51,3
CIMT+G-CSF	214-19-1i	80	4	52,2
CIMT+G-CSF	217-10-1i	80	1	26,3
CIMT+G-CSF	217-16-1i	80	16	206,6
CIMT+G-CSF	217-17-1i	80	18	248,7
CIMT+G-CSF	217-6-1i	80	7	86
CIMT+G-CSF	217-8-1i	80	12	182,3
CIMT+G-CSF	304-17-1i	80	2	40,1
CIMT+G-CSF	304-19-1i	80	2	20,7
CIMT+G-CSF	304-19-2i	80	2	29,2
CIMT+G-CSF	304-20-1i	80	3	56,4
CIMT+G-CSF	304-21-1i	80	8	107,1
CIMT+G-CSF	304-21-2i	80	0	1,6
CIMT+G-CSF	304-22-1i	80	1	13,6
CIMT+G-CSF	305-17-1i	80	2	21,3
CIMT+G-CSF	305-17-2i	80	7	108,4
CIMT+G-CSF	305-18-1i	80	1	12,1
CIMT+G-CSF	305-19-1i	80	3	35,4
CIMT+G-CSF	305-19-2i	80	3	31,4
CIMT/G-CSF	306-17-1i	80	1	35,9
CIMT/G-CSF	306-17-2i	80	5	75,3
CIMT/G-CSF	306-18-1i	80	2	29,4
CIMT/G-CSF	306-18-2i	80	3	51,9
CIMT/G-CSF	306-18-3i	80	4	62,3
CIMT/G-CSF	311-16-1i	80	5	95,6
CIMT/G-CSF	311-17-1i	80	25	377,9

CIMT/G-CSF	311-17-2i	80	17	205,6
CIMT/G-CSF	311-18-1i	80	5	98,6
CIMT/G-CSF	311-19-1i	80	15	192,9
CIMT/G-CSF	313-14.1i	80	2	53,2
CIMT/G-CSF	313-15-1i	80	7	119,8
CIMT/G-CSF	313-17-1i	80	3	48,4
CIMT/G-CSF	313-19-1i	80	1	10,1
CIMT/G-CSF	313-19-2i	80	5	81,9
CIMT/G-CSF	314-16-1i	80	10	203,6
CIMT/G-CSF	314-16-2i	80	5	71,9
CIMT/G-CSF	314-17-1i	80	10	123,5
CIMT/G-CSF	314-17-2i	80	6	95,5
CIMT/G-CSF	314-18-1i	80	1	15,4
CIMT/G-CSF	315-11-1i	80	2	32,5
CIMT/G-CSF	315-11-2i	80	2	37,1
CIMT/G-CSF	315-13-1i	80	10	146,9
CIMT/G-CSF	315-13-2i	80	5	96
CIMT/G-CSF	315-14-2i	80	3	56,4
G-CSF	220-16-1i	80	4	43,6
G-CSF	220-17-1i	80	7	85
G-CSF	220-18-1i	80	7	140,4
G-CSF	220-19-1i	80	10	138,6
G-CSF	220-7-1i	80	10	179
G-CSF	221-13-1i	80	9	106,9
G-CSF	221-16-1i	80	24	299,2
G-CSF	221-19-1i	80	22	238,1
G-CSF	221-22-1i	80	12	151,7
G-CSF	221-6-1i	80	9	117,2
G-CSF	222-16-1i	80	6	73,3
G-CSF	222-17-1i	80	11	162,6
G-CSF	222-21-1i	80	11	125,5
G-CSF	222-23-1i	80	8	113,1
G-CSF	222-8-1i	80	9	177,9
G-CSF	301-18-1i	80	1	16,3
G-CSF	301-19-1i	80	1	17,2
G-CSF	301-20-1i	80	5	72,5
G-CSF	301-20-2i	80	12	137,8
G-CSF	301-20-3i	80	13	151,1
G-CSF	302-16-1i	80	1	11,2
G-CSF	302-17-2i	80	1	20,2
G-CSF	302-18-1i	80	0	3,6
G-CSF	303-15-1i	80	8	105,1
G-CSF	303-16-1i	80	12	150,6
G-CSF	303-16-2i	80	7	94,9
G-CSF	303-17-1i	80	8	113,8
G-CSF	303-17-2i	80	3	43

basilar dendrite Sholl 90µm

Group	Number	Radius(µm)	Intersections	Length(µm)
Control	215-11-1i	90	1	47,6
Control	215-15-1i	90	1	27,2
Control	215-16-1i	90	2	22,4
Control	215-18-1i	90	5	57,3
Control	215-9-1i	90	1	13,6

Control	216-22-1i	90	1	22,6
Control	216-6-1i	90	10	135,1
Control	216-8-1i	90	5	70,6
Control	219-10-1i	90	5	75,8
Control	219-14-1i	90	9	97,3
Control	219-15-1i	90	4	42
Control	219-17-1i	90	7	90,6
Control	219-21-1i	90	8	115
Control	297-19-1i	90	3	51
Control	297-19-2i	90	0	12,3
Control	297-20-1i	90	7	127
Control	297-22-1i	90	1	14,2
Control	297-22-2i	90	6	74
Control	298-15-1i	90	4	61,3
Control	298-15-2i	90	1	21,2
Control	298-16-1i	90	0	5,3
Control	298-17-1i	90	6	69,1
Control	298-18-1i	90	4	52,7
Control	308-17-1i	90	4	55,2
Control	308-18-1i	90	7	99,2
Control	308-18-2i	90	2	29,3
Control	308-21-1i	90	1	10,6
Control	308-21-2i	90	5	76,1
CIMT	211-10-1i	90	12	200,9
CIMT	211-13-1i	90	4	64,9
CIMT	211-13-2i	90	4	50,6
CIMT	211-14-1i	90	4	63,6
CIMT	211-14-2i	90	0	16,8
CIMT	211-15-1i	90	3	33,7
CIMT	211-15-2i	90	5	66,8
CIMT	212-13-1i	90	3	36,1
CIMT	212-13-2i	90	4	46,4
CIMT	212-14-1i	90	1	11
CIMT	212-14-2i	90	3	68,5
CIMT	212-15-1i	90	1	25,1
CIMT	299-14-1i	90	1	10,3
CIMT	299-14-2i	90	1	34,5
CIMT	299-15-1i	90	6	87,3
CIMT	299-16-1i	90	2	35,1
CIMT	299-17-1i	90	1	12
CIMT	300-16-1i	90	2	35,7
CIMT	300-20-1i	90	6	81,4
CIMT	300-22-1i	90	8	102,9
CIMT	300-24-1i	90	7	135,2
CIMT	300-24-2i	90	8	149,8
CIMT	309-15-1i	90	4	67,5
CIMT	309-15-2i	90	3	54,6
CIMT	309-16-1i	90	2	25,5
CIMT	309-16-2i	90	14	203,5
CIMT	309-17-1i	90	7	84,6
CIMT	218-11-1i	90	1	12,3
CIMT	218-14-1i	90	3	49,3
CIMT	218-16-1i	90	15	148
CIMT	218-17-1i	90	3	63
CIMT	218-19-1i	90	10	125,1
CIMT+G-CSF	213-17-1i	90	4	53,4

CIMT+G-CSF	213-18-1i	90	2	44,1
CIMT+G-CSF	213-8-1i	90	3	43
CIMT+G-CSF	214-14-1i	90	0	6
CIMT+G-CSF	214-14-2i	90	2	43,6
CIMT+G-CSF	214-17-1i	90	4	45,5
CIMT+G-CSF	214-18-1i	90	3	47,1
CIMT+G-CSF	214-19-1i	90	3	47,8
CIMT+G-CSF	217-10-1i	90	1	11
CIMT+G-CSF	217-16-1i	90	13	240,8
CIMT+G-CSF	217-17-1i	90	14	183,9
CIMT+G-CSF	217-6-1i	90	8	115,3
CIMT+G-CSF	217-8-1i	90	8	115,2
CIMT+G-CSF	304-17-1i	90	0	25,2
CIMT+G-CSF	304-19-1i	90	2	21,1
CIMT+G-CSF	304-19-2i	90	2	23,4
CIMT+G-CSF	304-20-1i	90	2	33,6
CIMT+G-CSF	304-21-1i	90	7	88
CIMT+G-CSF	304-22-1i	90	0	1,2
CIMT+G-CSF	305-17-1i	90	2	24,8
CIMT+G-CSF	305-17-2i	90	3	59,8
CIMT+G-CSF	305-18-1i	90	0	10
CIMT+G-CSF	305-19-1i	90	2	32,3
CIMT+G-CSF	305-19-2i	90	2	58
CIMT/G-CSF	306-17-1i	90	1	18,7
CIMT/G-CSF	306-17-2i	90	3	45,8
CIMT/G-CSF	306-18-1i	90	2	25,1
CIMT/G-CSF	306-18-2i	90	2	36,5
CIMT/G-CSF	306-18-3i	90	2	44,2
CIMT/G-CSF	311-16-1i	90	4	78,6
CIMT/G-CSF	311-17-1i	90	24	363,9
CIMT/G-CSF	311-17-2i	90	16	208,8
CIMT/G-CSF	311-18-1i	90	3	61,2
CIMT/G-CSF	311-19-1i	90	16	194,9
CIMT/G-CSF	313-14.1i	90	1	13,6
CIMT/G-CSF	313-15-1i	90	6	82
CIMT/G-CSF	313-17-1i	90	4	44
CIMT/G-CSF	313-19-1i	90	1	14,2
CIMT/G-CSF	313-19-2i	90	3	64,6
CIMT/G-CSF	314-16-1i	90	7	109,1
CIMT/G-CSF	314-16-2i	90	4	54,8
CIMT/G-CSF	314-17-1i	90	8	125,2
CIMT/G-CSF	314-17-2i	90	7	97,8
CIMT/G-CSF	314-18-1i	90	2	24,3
CIMT/G-CSF	315-11-1i	90	0	5,7
CIMT/G-CSF	315-11-2i	90	2	28,7
CIMT/G-CSF	315-13-1i	90	7	99,7
CIMT/G-CSF	315-13-2i	90	1	35,3
CIMT/G-CSF	315-14-2i	90	1	19,3
G-CSF	220-16-1i	90	4	55,3
G-CSF	220-17-1i	90	5	62,6
G-CSF	220-18-1i	90	4	60,3
G-CSF	220-19-1i	90	6	99,4
G-CSF	220-7-1i	90	6	80,9
G-CSF	221-13-1i	90	8	119,4
G-CSF	221-16-1i	90	22	272,5
G-CSF	221-19-1i	90	20	269,6

G-CSF	221-22-1i	90	12	149,1
G-CSF	221-6-1i	90	9	101,1
G-CSF	222-16-1i	90	6	71,9
G-CSF	222-17-1i	90	12	146,8
G-CSF	222-21-1i	90	10	122,3
G-CSF	222-23-1i	90	7	94,5
G-CSF	222-8-1i	90	10	104,4
G-CSF	301-18-1i	90	0	6,2
G-CSF	301-19-1i	90	1	12,5
G-CSF	301-20-1i	90	4	50,1
G-CSF	301-20-2i	90	11	130,5
G-CSF	301-20-3i	90	13	160
G-CSF	302-16-1i	90	1	10,4
G-CSF	302-17-2i	90	1	10,6
G-CSF	303-15-1i	90	5	70,5
G-CSF	303-16-1i	90	10	137,1
G-CSF	303-16-2i	90	4	77,3
G-CSF	303-17-1i	90	4	71,7
G-CSF	303-17-2i	90	3	39,6

basilar dendrite Sholl 100µm

Group	Number	Radius(µm)	Intersections	Length(µm)
Control	215-11-1i	100	0	0,2
Control	215-15-1i	100	0	1,8
Control	215-16-1i	100	2	21,8
Control	215-18-1i	100	2	58,2
Control	215-9-1i	100	0	7,5
Control	216-22-1i	100	1	10,5
Control	216-6-1i	100	7	106,6
Control	216-8-1i	100	3	70,2
Control	219-10-1i	100	3	40
Control	219-14-1i	100	7	89,7
Control	219-15-1i	100	5	50,9
Control	219-17-1i	100	6	69,1
Control	219-21-1i	100	8	88
Control	297-19-1i	100	2	24
Control	297-20-1i	100	4	72,9
Control	297-22-1i	100	1	15,1
Control	297-22-2i	100	6	74,4
Control	298-15-1i	100	4	45,1
Control	298-15-2i	100	0	1
Control	298-17-1i	100	6	70
Control	298-18-1i	100	4	43,4
Control	308-17-1i	100	4	67,4
Control	308-18-1i	100	6	98,1
Control	308-18-2i	100	1	11,9
Control	308-21-1i	100	0	10,2
Control	308-21-2i	100	4	74,9
CIMT	211-10-1i	100	13	166,3
CIMT	211-13-1i	100	5	55,1
CIMT	211-13-2i	100	4	54,2
CIMT	211-14-1i	100	4	41,9
CIMT	211-15-1i	100	2	21,6
CIMT	211-15-2i	100	4	50,7

CIMT	212-13-1i	100	1	23
CIMT	212-13-2i	100	4	46,8
CIMT	212-14-1i	100	1	10,5
CIMT	212-14-2i	100	2	41,3
CIMT	212-15-1i	100	1	11,9
CIMT	299-14-1i	100	1	10,5
CIMT	299-14-2i	100	0	7,3
CIMT	299-15-1i	100	5	62,2
CIMT	299-16-1i	100	1	18,1
CIMT	299-17-1i	100	1	12,8
CIMT	300-16-1i	100	1	14,9
CIMT	300-20-1i	100	2	49,8
CIMT	300-22-1i	100	8	85
CIMT	300-24-1i	100	6	99
CIMT	300-24-2i	100	6	88,9
CIMT	309-15-1i	100	4	52,8
CIMT	309-15-2i	100	1	15,3
CIMT	309-16-1i	100	0	4,3
CIMT	309-16-2i	100	11	188,5
CIMT	309-17-1i	100	5	78,6
CIMT	218-11-1i	100	0	8
CIMT	218-14-1i	100	3	36,8
CIMT	218-16-1i	100	11	167,7
CIMT	218-17-1i	100	3	38
CIMT	218-19-1i	100	8	107,8
CIMT+G-CSF	213-17-1i	100	4	36,1
CIMT+G-CSF	213-18-1i	100	0	4,9
CIMT+G-CSF	213-8-1i	100	1	26,8
CIMT+G-CSF	214-14-2i	100	0	9,6
CIMT+G-CSF	214-17-1i	100	4	44,3
CIMT+G-CSF	214-18-1i	100	2	28,5
CIMT+G-CSF	214-19-1i	100	2	56,3
CIMT+G-CSF	217-10-1i	100	0	1,2
CIMT+G-CSF	217-16-1i	100	14	179,2
CIMT+G-CSF	217-17-1i	100	11	133,2
CIMT+G-CSF	217-6-1i	100	6	77,5
CIMT+G-CSF	217-8-1i	100	6	105,8
CIMT+G-CSF	304-19-1i	100	2	21,7
CIMT+G-CSF	304-19-2i	100	2	24,1
CIMT+G-CSF	304-20-1i	100	2	21,4
CIMT+G-CSF	304-21-1i	100	5	60,9
CIMT+G-CSF	305-17-1i	100	2	23,2
CIMT+G-CSF	305-17-2i	100	2	33,4
CIMT+G-CSF	305-19-1i	100	1	21,5
CIMT+G-CSF	305-19-2i	100	1	23,2
CIMT/G-CSF	306-17-1i	100	0	16,9
CIMT/G-CSF	306-17-2i	100	2	50,1
CIMT/G-CSF	306-18-1i	100	2	28,2
CIMT/G-CSF	306-18-2i	100	0	14,8
CIMT/G-CSF	306-18-3i	100	1	18,7
CIMT/G-CSF	311-16-1i	100	4	57,5
CIMT/G-CSF	311-17-1i	100	22	342,7
CIMT/G-CSF	311-17-2i	100	16	201
CIMT/G-CSF	311-18-1i	100	2	24,8
CIMT/G-CSF	311-19-1i	100	11	195,6
CIMT/G-CSF	313-14.1i	100	0	3,1

CIMT/G-CSF	313-15-1i	100	5	72,3
CIMT/G-CSF	313-17-1i	100	4	47,9
CIMT/G-CSF	313-19-1i	100	0	6,2
CIMT/G-CSF	313-19-2i	100	2	35,3
CIMT/G-CSF	314-16-1i	100	7	81,4
CIMT/G-CSF	314-16-2i	100	1	32,8
CIMT/G-CSF	314-17-1i	100	8	94,6
CIMT/G-CSF	314-17-2i	100	6	89
CIMT/G-CSF	314-18-1i	100	0	1,1
CIMT/G-CSF	315-11-2i	100	2	21,4
CIMT/G-CSF	315-13-1i	100	6	95,9
CIMT/G-CSF	315-13-2i	100	1	10,6
CIMT/G-CSF	315-14-2i	100	0	7,7
G-CSF	220-16-1i	100	2	40,1
G-CSF	220-17-1i	100	4	44,7
G-CSF	220-18-1i	100	2	29,7
G-CSF	220-19-1i	100	6	94,5
G-CSF	220-7-1i	100	5	66,7
G-CSF	221-13-1i	100	8	108,9
G-CSF	221-16-1i	100	20	253,6
G-CSF	221-19-1i	100	17	246,1
G-CSF	221-22-1i	100	8	125,7
G-CSF	221-6-1i	100	8	106,3
G-CSF	222-16-1i	100	2	47,4
G-CSF	222-17-1i	100	11	129,5
G-CSF	222-21-1i	100	9	104,3
G-CSF	222-23-1i	100	5	82,1
G-CSF	222-8-1i	100	10	107,3
G-CSF	301-19-1i	100	1	13,6
G-CSF	301-20-1i	100	3	35,2
G-CSF	301-20-2i	100	11	122,9
G-CSF	301-20-3i	100	12	139,4
G-CSF	302-16-1i	100	1	10,1
G-CSF	302-17-2i	100	1	10,2
G-CSF	303-15-1i	100	4	52,5
G-CSF	303-16-1i	100	11	123,6
G-CSF	303-16-2i	100	2	26,2
G-CSF	303-17-1i	100	3	39,7
G-CSF	303-17-2i	100	1	24,9

basilar dendrite Sholl 110µm

Group	Number	Radius(µm)	Intersections	Length(µm)
Control	215-16-1i	110	2	29,1
Control	215-18-1i	110	1	13,9
Control	216-22-1i	110	1	10,4
Control	216-6-1i	110	7	81,7
Control	216-8-1i	110	2	35,4
Control	219-10-1i	110	3	31,5
Control	219-14-1i	110	7	75,5
Control	219-15-1i	110	4	56,7
Control	219-17-1i	110	3	45,9
Control	219-21-1i	110	8	89,2
Control	297-19-1i	110	1	25,6
Control	297-20-1i	110	2	33,8

Control	297-22-1i	110	1	16,6
Control	297-22-2i	110	3	48,1
Control	298-15-1i	110	4	60,8
Control	298-17-1i	110	6	70,8
Control	298-18-1i	110	4	51
Control	308-17-1i	110	2	33,9
Control	308-18-1i	110	5	91,8
Control	308-18-2i	110	0	9,5
Control	308-21-2i	110	4	54,2
CIMT	211-10-1i	110	9	191,1
CIMT	211-13-1i	110	3	48,8
CIMT	211-13-2i	110	2	24,8
CIMT	211-14-1i	110	2	42,4
CIMT	211-15-1i	110	2	21,1
CIMT	211-15-2i	110	4	54,3
CIMT	212-13-1i	110	0	5,6
CIMT	212-13-2i	110	2	36,2
CIMT	212-14-1i	110	1	10,3
CIMT	212-14-2i	110	0	20,5
CIMT	212-15-1i	110	1	10,6
CIMT	299-14-1i	110	0	9,1
CIMT	299-15-1i	110	6	94,2
CIMT	299-16-1i	110	1	10
CIMT	299-17-1i	110	0	13,7
CIMT	300-16-1i	110	0	5,3
CIMT	300-20-1i	110	0	4,9
CIMT	300-22-1i	110	7	84,8
CIMT	300-24-1i	110	2	43,7
CIMT	300-24-2i	110	3	50,9
CIMT	309-15-1i	110	4	46
CIMT	309-15-2i	110	1	32,6
CIMT	309-16-2i	110	11	139,4
CIMT	309-17-1i	110	5	53,3
CIMT	218-14-1i	110	1	28
CIMT	218-16-1i	110	9	105,7
CIMT	218-17-1i	110	3	39,6
CIMT	218-19-1i	110	9	119,4
CIMT+G-CSF	213-17-1i	110	2	25,8
CIMT+G-CSF	213-8-1i	110	1	11,4
CIMT+G-CSF	214-17-1i	110	4	47,5
CIMT+G-CSF	214-18-1i	110	2	23,6
CIMT+G-CSF	214-19-1i	110	1	24,1
CIMT+G-CSF	217-16-1i	110	13	173,6
CIMT+G-CSF	217-17-1i	110	8	99,2
CIMT+G-CSF	217-6-1i	110	3	48,5
CIMT+G-CSF	217-8-1i	110	5	76,7
CIMT+G-CSF	304-19-1i	110	1	14,5
CIMT+G-CSF	304-19-2i	110	1	13,6
CIMT+G-CSF	304-20-1i	110	3	21,9
CIMT+G-CSF	304-21-1i	110	6	70,9
CIMT+G-CSF	305-17-1i	110	2	26,7
CIMT+G-CSF	305-17-2i	110	1	19,6
CIMT+G-CSF	305-19-1i	110	2	12
CIMT+G-CSF	305-19-2i	110	1	10,9
CIMT/G-CSF	306-17-2i	110	0	20,5
CIMT/G-CSF	306-18-1i	110	0	12,9

CIMT/G-CSF	306-18-3i	110	0	0,1
CIMT/G-CSF	311-16-1i	110	4	46,4
CIMT/G-CSF	311-17-1i	110	19	293,2
CIMT/G-CSF	311-17-2i	110	15	197,2
CIMT/G-CSF	311-18-1i	110	2	23,4
CIMT/G-CSF	311-19-1i	110	9	128,8
CIMT/G-CSF	313-15-1i	110	4	61,8
CIMT/G-CSF	313-17-1i	110	4	53,1
CIMT/G-CSF	313-19-2i	110	1	18,9
CIMT/G-CSF	314-16-1i	110	5	75,2
CIMT/G-CSF	314-16-2i	110	1	11,8
CIMT/G-CSF	314-17-1i	110	8	96,8
CIMT/G-CSF	314-17-2i	110	5	70,1
CIMT/G-CSF	315-11-2i	110	1	21,4
CIMT/G-CSF	315-13-1i	110	6	64,2
CIMT/G-CSF	315-13-2i	110	1	10,2
G-CSF	220-16-1i	110	2	27,1
G-CSF	220-17-1i	110	3	55
G-CSF	220-18-1i	110	1	14,7
G-CSF	220-19-1i	110	6	101,8
G-CSF	220-7-1i	110	4	49,3
G-CSF	221-13-1i	110	5	70,6
G-CSF	221-16-1i	110	17	214,2
G-CSF	221-19-1i	110	17	204
G-CSF	221-22-1i	110	6	69,8
G-CSF	221-6-1i	110	8	106
G-CSF	222-16-1i	110	2	22,5
G-CSF	222-17-1i	110	7	91,9
G-CSF	222-21-1i	110	9	101,9
G-CSF	222-23-1i	110	5	57,9
G-CSF	222-8-1i	110	9	115,2
G-CSF	301-19-1i	110	0	9,7
G-CSF	301-20-1i	110	2	26,4
G-CSF	301-20-2i	110	8	132,2
G-CSF	301-20-3i	110	10	134,9
G-CSF	302-16-1i	110	1	10,1
G-CSF	302-17-2i	110	1	13,7
G-CSF	303-15-1i	110	4	44,9
G-CSF	303-16-1i	110	7	175,1
G-CSF	303-16-2i	110	1	17
G-CSF	303-17-1i	110	3	37,4
G-CSF	303-17-2i	110	0	8,6

basilar dendrite Sholl 120µm

Group	Number	Radius(µm)	Intersections	Length(µm)
Control	215-16-1i	120	1	18,3
Control	215-18-1i	120	0	8
Control	216-22-1i	120	1	10,5
Control	216-6-1i	120	4	79,9
Control	216-8-1i	120	1	19,3
Control	219-10-1i	120	2	29,9
Control	219-14-1i	120	6	70,1
Control	219-15-1i	120	3	41,4
Control	219-17-1i	120	3	37

Control	219-21-1i	120	7	86,1
Control	297-19-1i	120	0	0,7
Control	297-20-1i	120	1	18,8
Control	297-22-1i	120	1	10,5
Control	297-22-2i	120	3	36,8
Control	298-15-1i	120	2	45,3
Control	298-17-1i	120	2	49,3
Control	298-18-1i	120	3	36,8
Control	308-17-1i	120	1	28,9
Control	308-18-1i	120	3	64,8
Control	308-21-2i	120	2	58,9
CIMT	211-10-1i	120	8	113,3
CIMT	211-13-1i	120	3	39,4
CIMT	211-13-2i	120	1	24,8
CIMT	211-14-1i	120	2	34,8
CIMT	211-15-1i	120	3	38,1
CIMT	211-15-2i	120	4	52,9
CIMT	212-13-2i	120	1	16,5
CIMT	212-14-1i	120	1	12,2
CIMT	212-15-1i	120	1	12,8
CIMT	299-15-1i	120	1	27,7
CIMT	299-16-1i	120	1	10,9
CIMT	300-22-1i	120	7	74,5
CIMT	300-24-1i	120	1	17,7
CIMT	300-24-2i	120	2	34,1
CIMT	309-15-1i	120	3	42,4
CIMT	309-15-2i	120	1	17,9
CIMT	309-16-2i	120	9	146,8
CIMT	309-17-1i	120	5	54
CIMT	218-14-1i	120	0	6,7
CIMT	218-16-1i	120	9	99,6
CIMT	218-17-1i	120	2	35,9
CIMT	218-19-1i	120	2	81,1
CIMT+G-CSF	213-17-1i	120	1	22
CIMT+G-CSF	213-8-1i	120	0	13,9
CIMT+G-CSF	214-17-1i	120	2	50,9
CIMT+G-CSF	214-18-1i	120	2	27,2
CIMT+G-CSF	214-19-1i	120	1	10,8
CIMT+G-CSF	217-16-1i	120	12	147,2
CIMT+G-CSF	217-17-1i	120	7	83,3
CIMT+G-CSF	217-6-1i	120	2	29,7
CIMT+G-CSF	217-8-1i	120	5	77,6
CIMT+G-CSF	304-19-1i	120	1	10,4
CIMT+G-CSF	304-19-2i	120	1	10,8
CIMT+G-CSF	304-20-1i	120	2	41,1
CIMT+G-CSF	304-21-1i	120	6	70,7
CIMT+G-CSF	305-17-1i	120	2	23,9
CIMT+G-CSF	305-17-2i	120	0	8,9
CIMT+G-CSF	305-19-1i	120	0	32,3
CIMT+G-CSF	305-19-2i	120	1	16,7
CIMT/G-CSF	311-16-1i	120	3	57
CIMT/G-CSF	311-17-1i	120	14	231,2
CIMT/G-CSF	311-17-2i	120	14	163,5
CIMT/G-CSF	311-18-1i	120	2	40,8
CIMT/G-CSF	311-19-1i	120	8	106,7
CIMT/G-CSF	313-15-1i	120	2	33

CIMT/G-CSF	313-17-1i	120	4	50
CIMT/G-CSF	313-19-2i	120	1	12,4
CIMT/G-CSF	314-16-1i	120	4	62,6
CIMT/G-CSF	314-16-2i	120	0	1,2
CIMT/G-CSF	314-17-1i	120	7	117,3
CIMT/G-CSF	314-17-2i	120	3	60
CIMT/G-CSF	315-11-2i	120	1	11,7
CIMT/G-CSF	315-13-1i	120	5	63,4
CIMT/G-CSF	315-13-2i	120	1	10,1
G-CSF	220-16-1i	120	1	19,3
G-CSF	220-17-1i	120	1	33,9
G-CSF	220-18-1i	120	1	10,5
G-CSF	220-19-1i	120	4	65,2
G-CSF	220-7-1i	120	4	44,7
G-CSF	221-13-1i	120	5	57,2
G-CSF	221-16-1i	120	13	176,7
G-CSF	221-19-1i	120	17	185,2
G-CSF	221-22-1i	120	6	68,5
G-CSF	221-6-1i	120	5	65,8
G-CSF	222-16-1i	120	1	10,5
G-CSF	222-17-1i	120	6	64,5
G-CSF	222-21-1i	120	9	101,9
G-CSF	222-23-1i	120	4	53,9
G-CSF	222-8-1i	120	5	80,7
G-CSF	301-20-1i	120	0	15,5
G-CSF	301-20-2i	120	6	83,8
G-CSF	301-20-3i	120	8	108,2
G-CSF	302-16-1i	120	0	2,1
G-CSF	302-17-2i	120	1	11,9
G-CSF	303-15-1i	120	1	20,9
G-CSF	303-16-1i	120	6	97,8
G-CSF	303-16-2i	120	1	10,8
G-CSF	303-17-1i	120	2	33,3

basilar dendrite Sholl 130µm

Group	Number	Radius(µm)	Intersections	Length(µm)
Control	215-16-1i	130	1	12,6
Control	216-22-1i	130	0	11,1
Control	216-6-1i	130	3	34,7
Control	216-8-1i	130	1	11,7
Control	219-10-1i	130	2	22,3
Control	219-14-1i	130	5	56,5
Control	219-15-1i	130	2	30,2
Control	219-17-1i	130	3	34,2
Control	219-21-1i	130	6	75,1
Control	297-20-1i	130	0	6,8
Control	297-22-1i	130	1	10,6
Control	297-22-2i	130	1	15,9
Control	298-15-1i	130	1	36,9
Control	298-17-1i	130	1	20,3
Control	298-18-1i	130	3	37,6
Control	308-17-1i	130	0	0
Control	308-18-1i	130	0	24
Control	308-21-2i	130	2	22,3

CIMT	211-10-1i	130	2	76
CIMT	211-13-1i	130	2	34,1
CIMT	211-13-2i	130	0	0,5
CIMT	211-14-1i	130	1	13,2
CIMT	211-15-1i	130	2	31,7
CIMT	211-15-2i	130	3	32,2
CIMT	212-13-2i	130	0	3,8
CIMT	212-14-1i	130	1	11,7
CIMT	212-15-1i	130	1	13,3
CIMT	299-15-1i	130	1	14,2
CIMT	299-16-1i	130	1	12,4
CIMT	300-22-1i	130	6	74,3
CIMT	300-24-1i	130	2	28,6
CIMT	300-24-2i	130	2	21,8
CIMT	309-15-1i	130	2	39,3
CIMT	309-15-2i	130	1	13
CIMT	309-16-2i	130	5	99,6
CIMT	309-17-1i	130	5	53,9
CIMT	218-16-1i	130	9	109
CIMT	218-17-1i	130	2	25,7
CIMT	218-19-1i	130	2	21,5
CIMT+G-CSF	213-17-1i	130	1	17,6
CIMT+G-CSF	214-17-1i	130	0	17,9
CIMT+G-CSF	214-18-1i	130	2	20,7
CIMT+G-CSF	214-19-1i	130	1	13
CIMT+G-CSF	217-16-1i	130	10	125,9
CIMT+G-CSF	217-17-1i	130	8	69,3
CIMT+G-CSF	217-6-1i	130	1	21,7
CIMT+G-CSF	217-8-1i	130	5	69,2
CIMT+G-CSF	304-19-1i	130	1	10,7
CIMT+G-CSF	304-19-2i	130	0	12
CIMT+G-CSF	304-20-1i	130	0	6
CIMT+G-CSF	304-21-1i	130	4	56,2
CIMT+G-CSF	305-17-1i	130	2	27,2
CIMT+G-CSF	305-19-2i	130	1	18,3
CIMT/G-CSF	311-16-1i	130	1	37,3
CIMT/G-CSF	311-17-1i	130	8	180,6
CIMT/G-CSF	311-17-2i	130	14	174
CIMT/G-CSF	311-18-1i	130	2	23,9
CIMT/G-CSF	311-19-1i	130	2	72,9
CIMT/G-CSF	313-15-1i	130	1	41,4
CIMT/G-CSF	313-17-1i	130	3	42,2
CIMT/G-CSF	313-19-2i	130	0	1,8
CIMT/G-CSF	314-16-1i	130	2	36,4
CIMT/G-CSF	314-17-1i	130	6	77,4
CIMT/G-CSF	314-17-2i	130	2	34,1
CIMT/G-CSF	315-11-2i	130	0	4
CIMT/G-CSF	315-13-1i	130	2	62,6
CIMT/G-CSF	315-13-2i	130	2	11,7
G-CSF	220-16-1i	130	1	19,8
G-CSF	220-17-1i	130	1	10,3
G-CSF	220-18-1i	130	0	9,1
G-CSF	220-19-1i	130	4	52,5
G-CSF	220-7-1i	130	2	37,5
G-CSF	221-13-1i	130	3	48,4
G-CSF	221-16-1i	130	11	134,5

G-CSF	221-19-1i	130	16	203,9
G-CSF	221-22-1i	130	7	75,2
G-CSF	221-6-1i	130	4	49,8
G-CSF	222-16-1i	130	0	0,8
G-CSF	222-17-1i	130	5	52,3
G-CSF	222-21-1i	130	8	91,4
G-CSF	222-23-1i	130	3	40,7
G-CSF	222-8-1i	130	3	61,2
G-CSF	301-20-2i	130	3	70,8
G-CSF	301-20-3i	130	9	104,7
G-CSF	302-17-2i	130	1	11,7
G-CSF	303-15-1i	130	1	12,8
G-CSF	303-16-1i	130	7	84,9
G-CSF	303-16-2i	130	1	11,7
G-CSF	303-17-1i	130	1	21,1

basilar dendrite Sholl 140µm

Group	Number	Radius(µm)	Intersections	Length(µm)
Control	215-16-1i	140	0	15,3
Control	216-6-1i	140	2	25,8
Control	216-8-1i	140	1	13,6
Control	219-10-1i	140	2	21,4
Control	219-14-1i	140	4	52,4
Control	219-15-1i	140	2	20,8
Control	219-17-1i	140	1	25,3
Control	219-21-1i	140	3	48,3
Control	297-22-1i	140	1	10,2
Control	297-22-2i	140	0	0
Control	298-15-1i	140	1	11,1
Control	298-17-1i	140	0	4,8
Control	298-18-1i	140	2	23,5
Control	308-21-2i	140	0	21,4
CIMT	211-10-1i	140	1	11,5
CIMT	211-13-1i	140	2	25,9
CIMT	211-14-1i	140	1	11,3
CIMT	211-15-1i	140	2	22,4
CIMT	211-15-2i	140	2	27,9
CIMT	212-14-1i	140	1	11
CIMT	212-15-1i	140	1	11,5
CIMT	299-15-1i	140	0	6,9
CIMT	299-16-1i	140	1	10,4
CIMT	300-22-1i	140	5	55,2
CIMT	300-24-1i	140	0	23,4
CIMT	300-24-2i	140	2	20,7
CIMT	309-15-1i	140	1	15,5
CIMT	309-15-2i	140	1	11,9
CIMT	309-16-2i	140	1	40,8
CIMT	309-17-1i	140	4	53,9
CIMT	218-16-1i	140	7	83
CIMT	218-17-1i	140	1	16,2
CIMT	218-19-1i	140	2	22,5
CIMT+G-CSF	213-17-1i	140	0	2
CIMT+G-CSF	214-18-1i	140	1	16,8
CIMT+G-CSF	214-19-1i	140	0	7,7

CIMT+G-CSF	217-16-1i	140	10	108,8
CIMT+G-CSF	217-17-1i	140	4	65,7
CIMT+G-CSF	217-6-1i	140	1	10,9
CIMT+G-CSF	217-8-1i	140	2	58,5
CIMT+G-CSF	304-19-1i	140	1	10,6
CIMT+G-CSF	304-21-1i	140	1	28,5
CIMT+G-CSF	305-17-1i	140	1	13,3
CIMT+G-CSF	305-19-2i	140	0	3,1
CIMT/G-CSF	311-16-1i	140	1	10,3
CIMT/G-CSF	311-17-1i	140	4	65,6
CIMT/G-CSF	311-17-2i	140	11	154,2
CIMT/G-CSF	311-18-1i	140	3	50,8
CIMT/G-CSF	311-19-1i	140	1	17,8
CIMT/G-CSF	313-15-1i	140	1	12,6
CIMT/G-CSF	313-17-1i	140	1	17
CIMT/G-CSF	314-16-1i	140	2	21,8
CIMT/G-CSF	314-17-1i	140	6	70,2
CIMT/G-CSF	314-17-2i	140	1	21,2
CIMT/G-CSF	315-13-1i	140	2	21,7
CIMT/G-CSF	315-13-2i	140	2	25,3
G-CSF	220-16-1i	140	1	12
G-CSF	220-17-1i	140	1	10,6
G-CSF	220-19-1i	140	3	47,4
G-CSF	220-7-1i	140	1	25,2
G-CSF	221-13-1i	140	5	55,8
G-CSF	221-16-1i	140	8	122,6
G-CSF	221-19-1i	140	14	159,2
G-CSF	221-22-1i	140	5	68,1
G-CSF	221-6-1i	140	4	42,7
G-CSF	222-17-1i	140	5	57,7
G-CSF	222-21-1i	140	8	85,1
G-CSF	222-23-1i	140	2	43,9
G-CSF	222-8-1i	140	2	33,7
G-CSF	301-20-2i	140	3	36,1
G-CSF	301-20-3i	140	6	81,4
G-CSF	302-17-2i	140	1	10,2
G-CSF	303-15-1i	140	1	10,8
G-CSF	303-16-1i	140	4	128,3
G-CSF	303-16-2i	140	1	13,8
G-CSF	303-17-1i	140	1	13,1

basilar dendrite Sholl 150µm +

Group	Number	Radius(µm)	Intersections	Length(µm)
Control	216-6-1i	150	2	24,1
Control	216-8-1i	150	0	3,4
Control	219-10-1i	150	1	21,3
Control	219-14-1i	150	4	41,7
Control	219-15-1i	150	2	20,6
Control	219-17-1i	150	1	11,6
Control	219-21-1i	150	3	31,4
Control	297-22-1i	150	1	10,2
Control	298-15-1i	150	0	4
Control	298-18-1i	150	2	25,2
Control	216-6-1i	160	0	21,3

Control	219-10-1i	160	1	11,3
Control	219-14-1i	160	2	40,2
Control	219-15-1i	160	2	21
Control	219-17-1i	160	1	10,4
Control	219-21-1i	160	1	15,3
Control	297-22-1i	160	1	10,2
Control	298-18-1i	160	2	21,4
Control	219-10-1i	170	1	10,7
Control	219-14-1i	170	2	21,8
Control	219-15-1i	170	1	10,3
Control	219-17-1i	170	0	3
Control	219-21-1i	170	1	10,4
Control	297-22-1i	170	1	14,1
Control	298-18-1i	170	1	22
Control	219-10-1i	180	1	10,6
Control	219-14-1i	180	2	21,6
Control	219-15-1i	180	1	10,6
Control	219-21-1i	180	1	11
Control	297-22-1i	180	0	1,8
Control	298-18-1i	180	1	12,8
Control	219-10-1i	190	1	10,4
Control	219-14-1i	190	2	21,4
Control	219-15-1i	190	1	10,4
Control	219-21-1i	190	0	10,7
Control	298-18-1i	190	1	11,2
Control	219-10-1i	200	1	10,2
Control	219-14-1i	200	2	21,2
Control	219-15-1i	200	0	0,4
Control	298-18-1i	200	1	11
Control	219-10-1i	210	0	3,4
Control	219-14-1i	210	1	15,2
Control	298-18-1i	210	0	3,4
Control	219-14-1i	220	0	4
CIMT	211-10-1i	150	0	11,8
CIMT	211-13-1i	150	1	16,9
CIMT	211-14-1i	150	0	7
CIMT	211-15-1i	150	2	20,9
CIMT	211-15-2i	150	1	16,5
CIMT	212-14-1i	150	1	12,6
CIMT	212-15-1i	150	1	12,1
CIMT	299-16-1i	150	0	3,9
CIMT	300-22-1i	150	3	48
CIMT	300-24-2i	150	2	21,6
CIMT	309-15-1i	150	0	6,2
CIMT	309-15-2i	150	0	8,2
CIMT	309-16-2i	150	1	10,9
CIMT	309-17-1i	150	4	43,4
CIMT	211-13-1i	160	0	6,6
CIMT	211-15-1i	160	2	30,5
CIMT	211-15-2i	160	1	10,2
CIMT	212-14-1i	160	0	6,6
CIMT	212-15-1i	160	1	11,3
CIMT	300-22-1i	160	3	32,2
CIMT	300-24-2i	160	2	22,1
CIMT	309-16-2i	160	1	10,4
CIMT	309-17-1i	160	4	41,2

CIMT	211-15-1i	170	1	15,5
CIMT	211-15-2i	170	1	17,2
CIMT	212-15-1i	170	1	13,4
CIMT	300-22-1i	170	2	24
CIMT	300-24-2i	170	0	15,9
CIMT	309-16-2i	170	0	5,6
CIMT	309-17-1i	170	2	29,6
CIMT	211-15-1i	180	0	9,9
CIMT	211-15-2i	180	1	10,9
CIMT	212-15-1i	180	0	10,9
CIMT	300-22-1i	180	2	25,8
CIMT	309-17-1i	180	1	16,3
CIMT	211-15-2i	190	0	9,2
CIMT	300-22-1i	190	2	22,2
CIMT	309-17-1i	190	1	10,2
CIMT	300-22-1i	200	1	12
CIMT	309-17-1i	200	0	8,6
CIMT	300-22-1i	210	1	11,8
CIMT	300-22-1i	220	0	8,2
CIMT	218-16-1i	150	5	71,9
CIMT	218-17-1i	150	1	10,3
CIMT	218-19-1i	150	2	23,2
CIMT	218-16-1i	160	3	43,1
CIMT	218-17-1i	160	1	11,1
CIMT	218-19-1i	160	1	26,9
CIMT	218-16-1i	170	2	28,9
CIMT	218-17-1i	170	1	12,7
CIMT	218-19-1i	170	1	12,6
CIMT	218-16-1i	180	2	22,5
CIMT	218-17-1i	180	0	6,7
CIMT	218-19-1i	180	0	6,3
CIMT	218-16-1i	190	2	21,2
CIMT	218-16-1i	200	1	20,6
CIMT	218-16-1i	210	0	0,1
CIMT+G-CSF	214-18-1i	150	0	12,1
CIMT+G-CSF	217-16-1i	150	10	111,3
CIMT+G-CSF	217-17-1i	150	4	44,7
CIMT+G-CSF	217-6-1i	150	2	25,8
CIMT+G-CSF	217-8-1i	150	1	14,5
CIMT+G-CSF	304-19-1i	150	0	3,4
CIMT+G-CSF	304-21-1i	150	0	13,6
CIMT+G-CSF	305-17-1i	150	0	11,7
CIMT+G-CSF	217-16-1i	160	9	125,7
CIMT+G-CSF	217-17-1i	160	5	59,4
CIMT+G-CSF	217-6-1i	160	2	23,9
CIMT+G-CSF	217-8-1i	160	0	9,6
CIMT+G-CSF	217-16-1i	170	7	106
CIMT+G-CSF	217-17-1i	170	3	43,6
CIMT+G-CSF	217-6-1i	170	2	23,5
CIMT+G-CSF	217-16-1i	180	2	56,8
CIMT+G-CSF	217-17-1i	180	3	61
CIMT+G-CSF	217-6-1i	180	2	22,3
CIMT+G-CSF	217-16-1i	190	2	20,7
CIMT+G-CSF	217-17-1i	190	2	27,5
CIMT+G-CSF	217-6-1i	190	2	24,6
CIMT+G-CSF	217-16-1i	200	1	19,3

CIMT+G-CSF	217-17-1i	200	2	21,9
CIMT+G-CSF	217-6-1i	200	1	17,1
CIMT+G-CSF	217-16-1i	210	1	11,3
CIMT+G-CSF	217-17-1i	210	0	3,4
CIMT+G-CSF	217-6-1i	210	1	10,4
CIMT+G-CSF	217-16-1i	220	1	10,7
CIMT+G-CSF	217-6-1i	220	1	10,2
CIMT+G-CSF	217-16-1i	230	1	10,3
CIMT+G-CSF	217-6-1i	230	1	11
CIMT+G-CSF	217-16-1i	240	1	10,7
CIMT+G-CSF	217-6-1i	240	0	11,6
CIMT+G-CSF	217-16-1i	250	1	10,5
CIMT+G-CSF	217-16-1i	260	1	10,3
CIMT+G-CSF	217-16-1i	270	1	10,3
CIMT+G-CSF	217-16-1i	280	0	2,9
CIMT/G-CSF	311-16-1i	150	0	19,5
CIMT/G-CSF	311-17-1i	150	4	79,8
CIMT/G-CSF	311-17-2i	150	11	123,7
CIMT/G-CSF	311-18-1i	150	1	48,2
CIMT/G-CSF	311-19-1i	150	0	6,7
CIMT/G-CSF	313-15-1i	150	1	13,4
CIMT/G-CSF	313-17-1i	150	1	13,3
CIMT/G-CSF	314-16-1i	150	1	16
CIMT/G-CSF	314-17-1i	150	4	63,8
CIMT/G-CSF	314-17-2i	150	1	14,7
CIMT/G-CSF	315-13-1i	150	2	30,3
CIMT/G-CSF	315-13-2i	150	1	16,9
CIMT/G-CSF	311-17-1i	160	3	43,7
CIMT/G-CSF	311-17-2i	160	11	137,7
CIMT/G-CSF	311-18-1i	160	0	9
CIMT/G-CSF	313-15-1i	160	1	10,5
CIMT/G-CSF	313-17-1i	160	1	19,8
CIMT/G-CSF	314-16-1i	160	1	11,1
CIMT/G-CSF	314-17-1i	160	4	49,4
CIMT/G-CSF	314-17-2i	160	0	2,3
CIMT/G-CSF	315-13-1i	160	0	20,4
CIMT/G-CSF	315-13-2i	160	0	0,8
CIMT/G-CSF	311-17-1i	170	2	38,5
CIMT/G-CSF	311-17-2i	170	7	120,1
CIMT/G-CSF	313-15-1i	170	1	11,4
CIMT/G-CSF	313-17-1i	170	0	14,7
CIMT/G-CSF	314-16-1i	170	0	13,5
CIMT/G-CSF	314-17-1i	170	4	50,4
CIMT/G-CSF	311-17-1i	180	2	24,8
CIMT/G-CSF	311-17-2i	180	4	57,4
CIMT/G-CSF	313-15-1i	180	1	10,8
CIMT/G-CSF	314-17-1i	180	2	36,4
CIMT/G-CSF	311-17-1i	190	0	2,3
CIMT/G-CSF	311-17-2i	190	4	56,4
CIMT/G-CSF	313-15-1i	190	0	11,9
CIMT/G-CSF	314-17-1i	190	2	25,8
CIMT/G-CSF	311-17-2i	200	5	51,4
CIMT/G-CSF	314-17-1i	200	2	26,1
CIMT/G-CSF	311-17-2i	210	4	46,3
CIMT/G-CSF	314-17-1i	210	2	24,2
CIMT/G-CSF	311-17-2i	220	3	41,2

CIMT/G-CSF	314-17-1i	220	2	21
CIMT/G-CSF	311-17-2i	230	1	22,6
CIMT/G-CSF	314-17-1i	230	1	18,4
CIMT/G-CSF	311-17-2i	240	0	3,9
CIMT/G-CSF	314-17-1i	240	0	2,6
G-CSF	220-16-1i	150	1	11,1
G-CSF	220-17-1i	150	1	11,5
G-CSF	220-19-1i	150	3	34
G-CSF	220-7-1i	150	1	10,3
G-CSF	221-13-1i	150	2	54,3
G-CSF	221-16-1i	150	7	86,1
G-CSF	221-19-1i	150	13	153,7
G-CSF	221-22-1i	150	4	50,9
G-CSF	221-6-1i	150	5	51,2
G-CSF	222-17-1i	150	4	52
G-CSF	222-21-1i	150	8	85,7
G-CSF	222-23-1i	150	2	22,7
G-CSF	222-8-1i	150	1	15
G-CSF	301-20-2i	150	3	53,7
G-CSF	301-20-3i	150	4	49,8
G-CSF	302-17-2i	150	1	10,4
G-CSF	303-15-1i	150	1	14,9
G-CSF	303-16-1i	150	3	42,4
G-CSF	303-16-2i	150	1	12,9
G-CSF	303-17-1i	150	0	8,8
G-CSF	220-16-1i	160	0	3,4
G-CSF	220-17-1i	160	1	10,6
G-CSF	220-19-1i	160	2	27,8
G-CSF	220-7-1i	160	1	10,3
G-CSF	221-13-1i	160	1	12,8
G-CSF	221-16-1i	160	6	72,4
G-CSF	221-19-1i	160	10	125,4
G-CSF	221-22-1i	160	4	46,4
G-CSF	221-6-1i	160	5	58,3
G-CSF	222-17-1i	160	3	32,8
G-CSF	222-21-1i	160	6	80,6
G-CSF	222-23-1i	160	1	18,9
G-CSF	222-8-1i	160	1	10,4
G-CSF	301-20-2i	160	3	39,1
G-CSF	301-20-3i	160	2	26,4
G-CSF	302-17-2i	160	1	10,4
G-CSF	303-15-1i	160	1	11
G-CSF	303-16-1i	160	3	53,9
G-CSF	303-16-2i	160	1	31,4
G-CSF	220-17-1i	170	1	11,5
G-CSF	220-19-1i	170	2	23,9
G-CSF	220-7-1i	170	2	23,5
G-CSF	221-13-1i	170	1	10,2
G-CSF	221-16-1i	170	5	59,3
G-CSF	221-19-1i	170	9	121,3
G-CSF	221-22-1i	170	3	49,1
G-CSF	221-6-1i	170	4	56,3
G-CSF	222-17-1i	170	3	32,4
G-CSF	222-21-1i	170	5	62,2
G-CSF	222-23-1i	170	0	2,4
G-CSF	222-8-1i	170	1	10,6

G-CSF	301-20-2i	170	3	42,4
G-CSF	301-20-3i	170	0	1,5
G-CSF	302-17-2i	170	1	10,5
G-CSF	303-15-1i	170	1	10,7
G-CSF	303-16-1i	170	2	35
G-CSF	303-16-2i	170	1	13,3
G-CSF	220-17-1i	180	1	10,8
G-CSF	220-19-1i	180	1	24,6
G-CSF	220-7-1i	180	2	22,8
G-CSF	221-13-1i	180	1	11
G-CSF	221-16-1i	180	5	53,5
G-CSF	221-19-1i	180	6	76,1
G-CSF	221-22-1i	180	3	34,1
G-CSF	221-6-1i	180	3	37,8
G-CSF	222-17-1i	180	0	16
G-CSF	222-21-1i	180	4	45,6
G-CSF	222-8-1i	180	1	10,5
G-CSF	301-20-2i	180	1	28,1
G-CSF	302-17-2i	180	0	8,3
G-CSF	303-15-1i	180	1	10,5
G-CSF	303-16-1i	180	2	22,6
G-CSF	303-16-2i	180	0	5,5
G-CSF	220-17-1i	190	1	10,9
G-CSF	220-19-1i	190	1	13,4
G-CSF	220-7-1i	190	2	21,9
G-CSF	221-13-1i	190	0	1,2
G-CSF	221-16-1i	190	5	69,2
G-CSF	221-19-1i	190	4	62,6
G-CSF	221-22-1i	190	1	17,9
G-CSF	221-6-1i	190	3	38,9
G-CSF	222-21-1i	190	4	41
G-CSF	222-8-1i	190	1	11,2
G-CSF	301-20-2i	190	1	11,7
G-CSF	303-15-1i	190	1	12,5
G-CSF	303-16-1i	190	2	24,7
G-CSF	220-17-1i	200	0	9,6
G-CSF	220-19-1i	200	1	10,4
G-CSF	220-7-1i	200	1	16,7
G-CSF	221-16-1i	200	2	54,8
G-CSF	221-19-1i	200	1	36,3
G-CSF	221-22-1i	200	1	11,5
G-CSF	221-6-1i	200	2	29,5
G-CSF	222-21-1i	200	4	49,2
G-CSF	222-8-1i	200	1	11,3
G-CSF	301-20-2i	200	1	11,1
G-CSF	303-15-1i	200	1	11
G-CSF	303-16-1i	200	2	23,8
G-CSF	220-19-1i	210	1	10,3
G-CSF	220-7-1i	210	0	6,1
G-CSF	221-16-1i	210	2	25
G-CSF	221-19-1i	210	1	11,5
G-CSF	221-22-1i	210	0	2,3
G-CSF	221-6-1i	210	0	13,6
G-CSF	222-21-1i	210	1	39,2
G-CSF	222-8-1i	210	1	10,4
G-CSF	301-20-2i	210	0	1

G-CSF	303-15-1i	210	1	10,8
G-CSF	303-16-1i	210	2	22,3
G-CSF	220-19-1i	220	1	10,3
G-CSF	221-16-1i	220	2	24,3
G-CSF	221-19-1i	220	1	10,3
G-CSF	222-21-1i	220	1	10,3
G-CSF	222-8-1i	220	0	5,4
G-CSF	303-15-1i	220	1	11,1
G-CSF	303-16-1i	220	2	23,5
G-CSF	220-19-1i	230	1	11,1
G-CSF	221-16-1i	230	1	16,9
G-CSF	221-19-1i	230	1	10,4
G-CSF	222-21-1i	230	1	10,1
G-CSF	303-15-1i	230	0	5,8
G-CSF	303-16-1i	230	2	24,5
G-CSF	220-19-1i	240	0	2,8
G-CSF	221-16-1i	240	1	11,1
G-CSF	221-19-1i	240	1	11,6
G-CSF	222-21-1i	240	1	10,4
G-CSF	303-16-1i	240	1	27,5
G-CSF	221-16-1i	250	1	10,6
G-CSF	221-19-1i	250	0	2,6
G-CSF	222-21-1i	250	1	10,8
G-CSF	303-16-1i	250	0	3
G-CSF	221-16-1i	260	0	9,5
G-CSF	222-21-1i	260	0	5,2
