# Supplementary material for: Functional Improvement after Photothrombotic Stroke in Rats Is Associated with Different Patterns of Dendritic Plasticity after G-CSF Treatment and G-CSF Treatment Combined with Concomitant or Sequential Constraint-Induced Movement Therapy
Source: PLoS One. 2016 Jan 11;11(1):e0146679. doi: 10.1371/journal.pone.0146679 (PMC4713830; doi:10.1371/journal.pone.0146679)
Supplement: S2 Table — (PDF) [file pone.0146679.s002.pdf]

Data Figure 2

basilar dendrites summary corresponding contralateral cortex

Group	Number	Name	Qty	Length (µm)	Mean length (µm)
control	V215-10_1	basilar	6	480,3	80,1
control	V215-10_2	basilar	8	2557,1	319,6
control	V215-11_1	basilar	7	854,2	122
control	V215-12_1	basilar	7	966,2	138
control	V215-12_2	basilar	6	1519,3	253,2
control	V215-13_1	basilar	7	1129,2	161,3
control	V215-15_1	basilar	5	357,2	71,4
control	V215-15_2	basilar	6	819,2	136,5
control	V215-16_1	basilar	6	800,9	133,5
control	V215-18_1	basilar	5	655	131
control	V216-11_1	basilar	4	715,1	178,8
control	V216-12_1	basilar	5	563	112,6
control	V216-13_1	basilar	5	431,1	86,2
control	V216-14_1	basilar	6	731	121,8
control	V216-14_2	basilar	6	481,4	80,2
control	V216-16_1	basilar	6	348,2	58
control	V216-16_2	basilar	4	982,4	245,6
control	V216-17_1	basilar	6	614,8	102,5
control	V216-18_1	basilar	5	432,3	86,5
control	V216-18_2	basilar	9	946,1	105,1
control	V219-6_1	basilar	8	3217,9	402,2
control	V219-11_1	basilar	7	1916,8	273,8
control	V219-12_1	basilar	8	1623	202,9
control	V219-13_1	basilar	7	940,8	134,4
control	V219-13_2	basilar	7	628,9	89,8
control	V219-14_1	basilar	7	595	85
control	V219-14_2	basilar	10	1858,7	185,9
control	V219-16_1	basilar	5	976,2	195,2
control	V297-19_1	basilar	8	1713,5	214,2
control	V297-19_2	basilar	6	656,3	109,4
control	V297-20_1	basilar	10	679	67,9
control	V297-20_2	basilar	5	467	93,4
control	V297-20_3	basilar	5	1355	271
control	V297-22_1	basilar	5	2076,1	415,2
control	V297-22_2	basilar	5	480,1	96
control	V298-15_1	basilar	7	2260,2	322,9
control	V298-16_1	basilar	3	476,2	158,7
control	V298-16_2	basilar	7	823,5	117,6
control	V298-16_3	basilar	3	320,4	106,8
control	V298-17_1	basilar	5	430,3	86,1
control	V298-17_2	basilar	7	1671,3	238,8
control	V298-17_3	basilar	8	2063,6	257,9
control	V308-17_1	basilar	7	883,8	126,3
control	V308-17_2	basilar	8	1797,4	224,7
control	V308-17_3	basilar	6	1499,2	249,9
control	V308-17_4	basilar	5	1146,9	229,4
control	V308-18_1	basilar	8	1229,5	153,7
control	V308-18_2	basilar	6	1045,1	174,2
control	V308-18_3	basilar	6	1896,1	316
CIMT	V211-10_1	basilar	11	3488,5	317,1
CIMT	V211-10_2	basilar	9	1611	179

CIMT	V211-11_1	basilar	5	540,6	108,1
CIMT	V211-11_2	basilar	9	595,4	66,2
CIMT	V211-12_1	basilar	5	1222,9	244,6
CIMT	V211-13_1	basilar	4	764,8	191,2
CIMT	V211-13_2	basilar	7	1475,8	210,8
CIMT	V211-15_1	basilar	9	710,4	78,9
CIMT	V211-15_2	basilar	7	1030,9	147,3
CIMT	V211-16_1	basilar	10	1200,1	120
CIMT	V212-14_1	basilar	5	1763,1	352,6
CIMT	V212-14_2	basilar	5	2619,6	523,9
CIMT	V212-15_1	basilar	5	1951,3	390,3
CIMT	V212-18_1	basilar	4	1154,1	288,5
CIMT	V212-21_1	basilar	6	1150,9	191,8
CIMT	V212-21_2	basilar	8	2820,2	352,5
CIMT	V212-22_1	basilar	8	946,5	118,3
CIMT	V212-23_1	basilar	5	1009,7	201,9
CIMT	V212-23_2	basilar	6	4410,4	735,1
CIMT	V212-24_1	basilar	6	757,9	126,3
CIMT	V299-14_1	basilar	5	777,7	155,5
CIMT	V299-15_1	basilar	3	249,9	83,3
CIMT	V299-15_2	basilar	5	269,3	53,9
CIMT	V299-16_1	basilar	7	681,8	97,4
CIMT	V299-16_2	basilar	8	1307	163,4
CIMT	V299-17_1	basilar	7	1588	226,9
CIMT	V299-17_2	basilar	6	849,7	141,6
CIMT	V300-16_1	basilar	5	1504,2	300,8
CIMT	V300-18_1	basilar	7	793,7	113,4
CIMT	V300-18_2	basilar	7	1421,4	203,1
CIMT	V300-20_1	basilar	6	1074,6	179,1
CIMT	V300-22_1	basilar	8	2044,2	255,5
CIMT	V300-23_1	basilar	6	855,7	142,6
CIMT	V300-23_2	basilar	4	653,3	163,3
CIMT	V309-15_1	basilar	6	692	115,3
CIMT	V309-15_2	basilar	5	914,5	182,9
CIMT	V309-16_1	basilar	8	1200,6	150,1
CIMT	V309-16_2	basilar	8	481	60,1
CIMT	V309-16_3	basilar	7	1384,7	197,8
CIMT	V309-17_1	basilar	7	1061,5	151,6
CIMT	V309-17_2	basilar	5	2055,2	411
CIMT	V218-9_1	basilar	5	682,5	136,5
CIMT	V218-10_1	basilar	11	1617,8	147,1
CIMT	V218-11_1	basilar	6	2190,1	365
CIMT	V218-12_1	basilar	7	1780	254,3
CIMT	V218-12_2	basilar	6	926,9	154,5
CIMT	V218-13_1	basilar	6	834,9	139,2
CIMT	V218-15_1	basilar	4	838,8	209,7
CIMT	V218-17_1	basilar	5	1010,1	202
G-CSF	V220-9_1	basilar	9	1870,6	207,8
G-CSF	V220-11_1	basilar	5	338,1	67,6
G-CSF	V220-12_1	basilar	7	867,9	124
G-CSF	V220-16_1	basilar	4	1439,5	359,9
G-CSF	V220-16_2	basilar	6	692,2	115,4
G-CSF	V220-17_1	basilar	6	1813,8	302,3
G-CSF	V220-18_1	basilar	4	840,8	210,2
G-CSF	V221-2_1	basilar	8	1302,2	162,8
G-CSF	V221-4_1	basilar	5	244,7	48,9

G-CSF	V221-5_1	basilar	10	306,7	30,7
G-CSF	V221-6_1	basilar	4	1061,8	265,4
G-CSF	V221-7_1	basilar	5	762,2	152,4
G-CSF	V221-7_2	basilar	6	1780,8	296,8
G-CSF	V221-11_1	basilar	3	371,3	123,8
G-CSF	V221-13_1	basilar	12	3151,1	262,6
G-CSF	V221-15_1	basilar	5	424,2	84,8
G-CSF	V221-20_1	basilar	11	1252,8	113,9
G-CSF	V222-19_1	basilar	9	1946,2	216,2
G-CSF	V222-19_2	basilar	10	583,3	58,3
G-CSF	V222-20_1	basilar	12	1731,4	144,3
G-CSF	V222-20_2	basilar	4	1064,8	266,2
G-CSF	V222-21_1	basilar	8	2504,3	313
G-CSF	V222-22_1	basilar	6	1326,5	221,1
G-CSF	V222-22_2	basilar	8	1675,1	209,4
G-CSF	V222-23_1	basilar	8	1956,8	244,6
G-CSF	V222-24_1	basilar	7	1708	244
G-CSF	V301-18_1	basilar	7	893,4	127,6
G-CSF	V301-18_2	basilar	6	865,4	144,2
G-CSF	V301-18_3	basilar	4	495,9	124
G-CSF	V301-19_1	basilar	5	702,6	140,5
G-CSF	V301-19_2	basilar	9	789,7	87,7
G-CSF	V301-20_1	basilar	6	882,7	147,1
G-CSF	V301-20_2	basilar	9	1206,7	134,1
G-CSF	V302-15_1	basilar	4	644,4	161,1
G-CSF	V302-15_2	basilar	6	936,7	156,1
G-CSF	V302-15_3	basilar	5	709	141,8
G-CSF	V302-16_1	basilar	3	369,7	123,2
G-CSF	V302-16_2	basilar	3	191,2	63,7
G-CSF	V302-16_3	basilar	5	603,6	120,7
G-CSF	V302-16_4	basilar	5	877	175,4
G-CSF	V302-17_1	basilar	10	2361,3	236,1
G-CSF	V302-17_2	basilar	6	752	125,3
G-CSF	V302-17_3	basilar	4	585	146,3
G-CSF	V303-15_1	basilar	4	396,1	99
G-CSF	V303-15_2	basilar	7	870,9	124,4
G-CSF	V303-15_3	basilar	5	1205,3	241,1
G-CSF	V303-16_1	basilar	7	2162,9	309
G-CSF	V303-16_2	basilar	8	1196,7	149,6
G-CSF	V303-16_3	basilar	8	1155	144,4
G-CSF	V303-17_1	basilar	9	2169,8	241,1
CIMT+G-CSF	V213-13_1	basilar	5	556	111,2
CIMT+G-CSF	V213-16_1	basilar	3	283,6	94,5
CIMT+G-CSF	V213-17_1	basilar	6	998,2	166,4
CIMT+G-CSF	V213-17_2	basilar	4	1340,2	335,1
CIMT+G-CSF	V213-18_1	basilar	5	1791,5	358,3
CIMT+G-CSF	V213-18_2	basilar	3	772,7	257,6
CIMT+G-CSF	V213-19_1	basilar	6	1249,5	208,2
CIMT+G-CSF	V213-19_2	basilar	10	690,8	69,1
CIMT+G-CSF	V213-20_1	basilar	4	573,6	143,4
CIMT+G-CSF	V213-22_1	basilar	5	1444,6	288,9
CIMT+G-CSF	V213-23_1	basilar	9	866	96,2
CIMT+G-CSF	V214-14_1	basilar	8	1862	232,8
CIMT+G-CSF	V214-17_1	basilar	5	1427,1	285,4
CIMT+G-CSF	V214-18_1	basilar	9	2897,1	321,9
CIMT+G-CSF	V214-19_1	basilar	5	650,6	130,1

CIMT+G-CSF	V214-19_2	basilar	4	1142,4	285,6
CIMT+G-CSF	V214-20_1	basilar	6	1669,2	278,2
CIMT+G-CSF	V214-21_1	basilar	5	1076,2	215,2
CIMT+G-CSF	V217-1_1	basilar	9	2910,3	323,4
CIMT+G-CSF	V217-2_1	basilar	7	1582,6	226,1
CIMT+G-CSF	V217-4_1	basilar	5	3923,5	784,7
CIMT+G-CSF	V217-6_1	basilar	6	1092,1	182
CIMT+G-CSF	V217-6_2	basilar	5	1616,4	323,3
CIMT+G-CSF	V217-6_3	basilar	3	1502,6	500,9
CIMT+G-CSF	V217-8_1	basilar	7	893,5	127,6
CIMT+G-CSF	V217-8_4	basilar	6	1462	243,7
CIMT+G-CSF	V217-10_1	basilar	9	670,6	74,5
CIMT+G-CSF	V217-11_1	basilar	7	2564,2	366,3
CIMT+G-CSF	V304-16_1	basilar	3	341,8	113,9
CIMT+G-CSF	V304-17_1	basilar	6	988	164,7
CIMT+G-CSF	V304-17_2	basilar	2	360,3	180,2
CIMT+G-CSF	V304-17_3	basilar	6	1000,2	166,7
CIMT+G-CSF	V304-17_4	basilar	5	1138,7	227,7
CIMT+G-CSF	V304-19_1	basilar	4	447,6	111,9
CIMT+G-CSF	V304-19_2	basilar	4	703,4	175,9
CIMT+G-CSF	V305-17_1	basilar	6	351,1	58,5
CIMT+G-CSF	V305-17_2	basilar	6	550,5	91,8
CIMT+G-CSF	V305-17_3	basilar	8	711,8	89
CIMT+G-CSF	V305-18_1	basilar	9	1274,8	141,6
CIMT+G-CSF	V305-18_2	basilar	5	648,8	129,8
CIMT+G-CSF	V305-18_3	basilar	6	324	54
CIMT+G-CSF	V305-18_4	basilar	6	635,7	105,9
CIMT+G-CSF	V305-19_1	basilar	4	227	56,7
CIMT/G-CSF	V305-16_1	basilar	7	964,9	137,8
CIMT/G-CSF	V305-16_2	basilar	5	583,4	116,7
CIMT/G-CSF	V305-17_1	basilar	9	1494,2	166
CIMT/G-CSF	V305-17_2	basilar	7	1458,4	208,3
CIMT/G-CSF	V305-17_3	basilar	8	1089,5	136,2
CIMT/G-CSF	V305-17_4	basilar	7	803,7	114,8
CIMT/G-CSF	V305-17_5	basilar	5	512,9	102,6
CIMT/G-CSF	V311-16_1	basilar	8	2715,1	339,4
CIMT/G-CSF	V311-17_1	basilar	10	1861,2	186,1
CIMT/G-CSF	V311-17_2	basilar	4	1031,2	257,8
CIMT/G-CSF	V311-19_1	basilar	7	1226,4	175,2
CIMT/G-CSF	V311-20_1	basilar	8	2671,2	333,9
CIMT/G-CSF	V311-20_2	basilar	3	791,1	263,7
CIMT/G-CSF	V311-20_3	basilar	6	768,1	128
CIMT/G-CSF	V313-14_1	basilar	4	1245,5	311,4
CIMT/G-CSF	V313-15_1	basilar	6	285,3	47,5
CIMT/G-CSF	V313-15_2	basilar	5	821,9	164,4
CIMT/G-CSF	V313-17_1	basilar	5	1445,3	289,1
CIMT/G-CSF	V313-17_2	basilar	3	420,6	140,2
CIMT/G-CSF	V313-17_3	basilar	5	212,1	42,4
CIMT/G-CSF	V313-19_1	basilar	9	411,4	45,7
CIMT/G-CSF	V314-16_1	basilar	5	483	96,6
CIMT/G-CSF	V314-16_2	basilar	7	1091,6	155,9
CIMT/G-CSF	V314-17_1	basilar	8	1140,1	142,5
CIMT/G-CSF	V314-17_2	basilar	8	219,8	27,5
CIMT/G-CSF	V314-17_3	basilar	8	905,1	113,1
CIMT/G-CSF	V314-18_1	basilar	9	822	91,3
CIMT/G-CSF	V314-18_2	basilar	9	1210,3	134,5

CIMT/G-CSF	V315-11_1	basilar	4	852,2	213
CIMT/G-CSF	V315-11_2	basilar	8	939	117,4
CIMT/G-CSF	V315-13_1	basilar	6	1192,8	198,8
CIMT/G-CSF	V315-13_2	basilar	6	2082	347
CIMT/G-CSF	V315-14_1	basilar	6	926,2	154,4
CIMT/G-CSF	V315-14_2	basilar	8	1104,9	138,1
CIMT/G-CSF	V315-14_3	basilar	7	2328,6	332,7

Data Figure 3

Dendrite length corresponding contralateral cortex

Group	animal no	length basilar	no of neurons	length per neuron basilar	length apical	length per neuron apical
Control	215	10138,6	10	1013,9	3898,6	389,9
Control	216	6245,4	10	624,5	3997,5	399,8
Control	219	10381,3	8	1297,7	4646,2	580,8
Control	297	7427	7	1061,0	3359,4	479,9
Control	298	8045,5	7	1149,4	4855,4	693,6
Control	308	9498	7	1356,9	4570,4	652,9
CIMT	211	12640,4	10	1264,0	6745,7	674,6
CIMT	212	18583,7	10	1858,4	9849,3	984,9
CIMT	299	7330,5	7	1047,2	4355,9	622,3
CIMT	300	8347,1	7	1192,4	4171,5	595,9
CIMT	309	7789,5	7	1112,8	5979,6	854,2
CIMT	218	9881,1	8	1235,1	4593,4	574,2
CIMT+G-CSF	213	10566,7	11	960,6	5339,2	485,4
CIMT+G-CSF	214	10724,6	7	1532,1	4090,8	584,4
CIMT+G-CSF	217	18217,8	10	1821,8	4234,4	423,4
CIMT+G-CSF	304	4980	7	711,4	2774,6	396,4
CIMT+G-CSF	305	5124	8	640,5	3824,6	478,1
CIMT/G-CSF	306	6907	7	986,7	4657,8	665,4
CIMT/G-CSF	311	10084,9	7	1440,7	5792,8	827,5
CIMT/G-CSF	313	4842,1	7	691,7	3058,9	437,0
CIMT/G-CSF	314	5871,9	6	978,7	4727,9	788,0
CIMT/G-CSF	315	9425,7	7	1346,5	4032,4	576,1
G-CSF	220	7862,9	7	1123,3	4967,2	709,6
G-CSF	221	10657,8	10	1065,8	6827,1	682,7
G-CSF	222	14316,3	9	1590,7	8178,1	908,7
G-CSF	301	5836,4	7	833,8	4840,8	691,5
G-CSF	302	8029,9	10	803,0	5822	582,2
G-CSF	303	9156,7	7	1308,1	5242,9	749,0

Data Figure 5

Intersections corresponding contralateral cortex

Group	animal no	Qty basilar	no of neurons	Qty per neuron basilar	Qty apical	Qty per neuron apical
Control	215	746	10	74,6	273	27,3
Control	216	473	10	47,3	285	28,5
Control	219	789	8	98,6	310	38,8
Control	297	534	7	76,3	237	33,9
Control	298	623	7	89,0	445	63,6
Control	308	643	7	91,9	347	49,6
CIMT	211	912	10	91,2	471	47,1
CIMT	212	1332	10	133,2	699	69,9
CIMT	299	692	7	98,9	311	44,4
CIMT	300	411	7	58,7	305	43,6
CIMT	309	636	8	79,5	293	36,6
CIMT	218	583	8	72,9	371	46,4
CIMT+G-CSF	213	792	11	72,0	380	34,5
CIMT+G-CSF	214	818	7	116,9	279	39,9
CIMT+G-CSF	217	1163	10	116,3	306	30,6
CIMT+G-CSF	304	370	7	52,9	147	21,0
CIMT+G-CSF	305	341	8	42,6	283	35,4
CIMT/G-CSF	306	501	7	71,6	337	48,1
CIMT/G-CSF	311	806	7	115,1	372	53,1
CIMT/G-CSF	313	356	7	50,9	214	30,6
CIMT/G-CSF	314	425	6	70,8	299	49,8
CIMT/G-CSF	315	675	7	96,4	217	31,0
G-CSF	220	552	7	78,9	332	47,4
G-CSF	221	838	10	83,8	473	47,3
G-CSF	222	1054	9	117,1	568	63,1
G-CSF	301	427	7	61,0	332	47,4
G-CSF	302	594	10	59,4	340	34,0
G-CSF	303	683	7	97,6	378	54,0

Group	animal no	number of neu	Qty total	Qty per neuron total
Control	215	10	1019	101,9
Control	216	10	758	75,8
Control	219	8	1099	137,4
Control	297	7	771	110,1
Control	298	7	1068	152,6
Control	308	7	990	141,4
CIMT	211	10	1383	138,3
CIMT	212	10	2031	203,1
CIMT	299	7	1003	143,3
CIMT	300	7	716	102,3
CIMT	309	8	929	116,1
CIMT	218	8	954	119,3
CIMT+G-CSF	213	11	1172	106,5
CIMT+G-CSF	214	7	1097	156,7
CIMT+G-CSF	217	10	1469	146,9
CIMT+G-CSF	304	7	517	73,9
CIMT+G-CSF	305	8	624	78,0
CIMT/G-CSF	306	7	838	119,7
CIMT/G-CSF	311	7	1178	168,3
CIMT/G-CSF	313	7	570	81,4
CIMT/G-CSF	314	6	724	120,7

CIMT/G-CSF	315	7	892	127,4
G-CSF	220	7	884	126,3
G-CSF	221	10	1311	131,1
G-CSF	222	9	1622	180,2
G-CSF	301	7	759	108,4
G-CSF	302	10	934	93,4
G-CSF	303	7	1061	151,6

Data Figure 6

sholl analysis
apical dendrite Sholl 10µm

Group	number	Radius(µm)	Intersections	Length(µm)
Control	V215-10_1	10	2	7,9
Control	V215-10_2	10	0	0
Control	V215-11_1	10	0	0
Control	V215-12_1	10	0	0
Control	V215-12_2	10	1	3
Control	V215-13_1	10	0	0
Control	V215-15_1	10	0	0
Control	V215-15_2	10	0	0
Control	V215-16_1	10	0	0
Control	V215-18_1	10	0	0
Control	V216-11_1	10	0	0
Control	V216-12_1	10	1	1
Control	V216-13_1	10	1	1,9
Control	V216-14_1	10	0	0
Control	V216-14_2	10	0	0
Control	V216-16_1	10	1	0,5
Control	V216-16_2	10	0	0
Control	V216-17_1	10	1	0,1
Control	V216-18_1	10	1	1,9
Control	V216-18_2	10	0	0
Control	V219-6_1	10	0	0
Control	V219-11_1	10	0	0
Control	V219-12_1	10	0	0
Control	V219-13_1	10	2	2,1
Control	V219-13_2	10	0	0
Control	V219-14_1	10	0	0
Control	V219-14_2	10	1	1,4
Control	V219-16_1	10	0	0
Control	V297-19_1	10	0	0
Control	V297-19_2	10	0	0
Control	V297-20_1	10	0	0
Control	V297-20_2	10	0	0
Control	V297-20_3	10	0	0
Control	V297-22_1	10	0	0
Control	V297-22_2	10	0	0
Control	V298-15_1	10	0	0
Control	V298-16_1	10	1	0,1
Control	V298-16_2	10	0	0
Control	V298-16_3	10	0	0
Control	V298-17_1	10	1	1
Control	V298-17_2	10	1	0,7

Control	V298-17_3	10	0	0
Control	V308-17_1	10	0	0
Control	V308-17_2	10	0	0
Control	V308-17_3	10	0	0
Control	V308-17_4	10	0	0
Control	V308-18_1	10	1	9,5
Control	V308-18_2	10	0	0
Control	V308-18_3	10	0	0
CIMT	V211-10_1	10	0	0
CIMT	V211-10_2	10	0	0
CIMT	V211-11_1	10	0	0
CIMT	V211-11_2	10	0	0
CIMT	V211-12_1	10	0	0
CIMT	V211-13_1	10	0	0
CIMT	V211-13_2	10	0	0
CIMT	V211-15_1	10	0	0
CIMT	V211-15_2	10	1	0,9
CIMT	V211-16_1	10	1	0,2
CIMT	V212-14_1	10	0	0
CIMT	V212-14_2	10	0	0
CIMT	V212-15_1	10	0	0
CIMT	V212-18_1	10	0	0
CIMT	V212-21_1	10	0	0
CIMT	V212-21_2	10	0	0
CIMT	V212-22_1	10	0	0
CIMT	V212-23_1	10	0	0
CIMT	V212-23_2	10	1	0,4
CIMT	V212-24_1	10	0	0
CIMT	V218-9_1	10	0	0
CIMT	V218-10_1	10	0	0
CIMT	V218-11_1	10	0	0
CIMT	V218-12_1	10	1	1,4
CIMT	V218-12_2	10	0	0
CIMT	V218-13_1	10	0	0
CIMT	V218-15_1	10	0	0
CIMT	V218-17_1	10	0	0
CIMT	V299-14_1	10	1	2,9
CIMT	V299-15_1	10	1	0,1
CIMT	V299-15_2	10	0	0
CIMT	V299-16_1	10	0	0
CIMT	V299-16_2	10	0	0
CIMT	V299-17_1	10	0	0
CIMT	V299-17_2	10	0	0
CIMT	V300-16_1	10	0	0
CIMT	V300-18_1	10	0	0
CIMT	V300-18_2	10	1	0,9
CIMT	V300-20_1	10	0	0
CIMT	V300-22_1	10	0	0
CIMT	V300-23_1	10	0	0
CIMT	V300-23_2	10	1	0,1
CIMT	V309-15_1	10	0	0
CIMT	V309-15_2	10	0	0
CIMT	V309-16_1	10	0	0
CIMT	V309-16_2	10	0	0
CIMT	V309-16_3	10	1	0,6
CIMT	V309-17_1	10	0	0

CIMT	V309-17_2	10	1	1,1
CIMT+G-CSF	V213-13_1	10	0	0
CIMT+G-CSF	V213-16_1	10	1	1,6
CIMT+G-CSF	V213-17_1	10	0	0
CIMT+G-CSF	V213-17_2	10	0	0
CIMT+G-CSF	V213-18_1	10	1	0,6
CIMT+G-CSF	V213-18_2	10	1	1,4
CIMT+G-CSF	V213-19_1	10	0	0
CIMT+G-CSF	V213-19_2	10	0	0
CIMT+G-CSF	V213-20_1	10	1	1
CIMT+G-CSF	V213-22_1	10	0	0
CIMT+G-CSF	V213-23_1	10	1	1,6
CIMT+G-CSF	V214-14_1	10	0	0
CIMT+G-CSF	V214-17_1	10	0	0
CIMT+G-CSF	V214-18_1	10	0	0
CIMT+G-CSF	V214-19_1	10	0	0
CIMT+G-CSF	V214-19_2	10	1	1,1
CIMT+G-CSF	V214-20_1	10	0	0
CIMT+G-CSF	V214-21_1	10	0	0
CIMT+G-CSF	V217-1_1	10	0	0
CIMT+G-CSF	V217-2_1	10	0	0
CIMT+G-CSF	V217-4_1	10	0	0
CIMT+G-CSF	V217-6_1	10	0	0
CIMT+G-CSF	V217-6_3	10	0	0
CIMT+G-CSF	V217-8_1	10	0	0
CIMT+G-CSF	V217-8_4	10	0	0
CIMT+G-CSF	V217-10_1	10	0	0
CIMT+G-CSF	V217-11_1	10	0	0
CIMT+G-CSF	V304-16_1	10	1	15,8
CIMT+G-CSF	V304-17_1	10	1	4,2
CIMT+G-CSF	V304-17_2	10	1	4,5
CIMT+G-CSF	V304-17_3	10	1	0,3
CIMT+G-CSF	V304-17_4	10	0	0
CIMT+G-CSF	V304-19_1	10	1	2,5
CIMT+G-CSF	V304-19_2	10	1	1,9
CIMT+G-CSF	V305-17_1	10	0	0
CIMT+G-CSF	V305-17_2	10	1	4,5
CIMT+G-CSF	V305-17_3	10	1	0,3
CIMT+G-CSF	V305-18_1	10	1	0,4
CIMT+G-CSF	V305-18_2	10	1	2,1
CIMT+G-CSF	V305-18_3	10	0	0
CIMT+G-CSF	V305-18_4	10	0	0
CIMT+G-CSF	V305-19_1	10	1	0,3
CIMT/G-CSF	V306-16_1	10	1	1,3
CIMT/G-CSF	V306-16_2	10	0	0
CIMT/G-CSF	V306-17_1	10	0	0
CIMT/G-CSF	V306-17_2	10	1	0,6
CIMT/G-CSF	V306-17_3	10	0	0
CIMT/G-CSF	V306-17_4	10	1	0,8
CIMT/G-CSF	V306-17_5	10	1	1,6
CIMT/G-CSF	V311-16_1	10	0	0
CIMT/G-CSF	V311-17_1	10	0	0
CIMT/G-CSF	V311-17_2	10	0	0
CIMT/G-CSF	V311-19_1	10	1	0,8
CIMT/G-CSF	V311-20_1	10	0	0
CIMT/G-CSF	V311-20_2	10	0	0

CIMT/G-CSF	V311-20_3	10	0	0
CIMT/G-CSF	V313-14_1	10	0	0
CIMT/G-CSF	V313-15_1	10	0	0
CIMT/G-CSF	V313-15_2	10	1	0,9
CIMT/G-CSF	V313-17_1	10	0	0
CIMT/G-CSF	V313-17_2	10	1	1
CIMT/G-CSF	V313-17_3	10	0	0
CIMT/G-CSF	V313-19_1	10	0	0
CIMT/G-CSF	V314-16_1	10	2	4
CIMT/G-CSF	V314-16_2	10	0	0
CIMT/G-CSF	V314-17_1	10	0	0
CIMT/G-CSF	V314-17_2	10	1	0,1
CIMT/G-CSF	V314-17_3	10	1	2,3
CIMT/G-CSF	V314-18_1	10	1	0,3
CIMT/G-CSF	V314-18_2	10	0	0
CIMT/G-CSF	V315-11_1	10	1	0,8
CIMT/G-CSF	V315-11_2	10	0	0
CIMT/G-CSF	V315-13_1	10	0	0
CIMT/G-CSF	V315-13_2	10	0	0
CIMT/G-CSF	V315-14_1	10	0	0
CIMT/G-CSF	V315-14_2	10	0	0
CIMT/G-CSF	V315-14_3	10	0	0
G-CSF	V220-9_1	10	0	0
G-CSF	V220-11_1	10	1	4,2
G-CSF	V220-12_1	10	0	0
G-CSF	V220-16_1	10	1	2,7
G-CSF	V220-16_2	10	1	3,6
G-CSF	V220-17_1	10	0	0
G-CSF	V220-18_1	10	1	1,6
G-CSF	V221-2_1	10	0	0
G-CSF	V221-4_1	10	0	0
G-CSF	V221-5_1	10	0	0
G-CSF	V221-6_1	10	1	0,2
G-CSF	V221-7_1	10	1	1,1
G-CSF	V221-7_2	10	0	0
G-CSF	V221-11_1	10	0	0
G-CSF	V221-13_1	10	0	0
G-CSF	V221-15_1	10	0	0
G-CSF	V221-20_1	10	0	0
G-CSF	V222-19_1	10	0	0
G-CSF	V222-19_2	10	0	0
G-CSF	V222-20_1	10	0	0
G-CSF	V222-20_2	10	0	0
G-CSF	V222-21_1	10	0	0
G-CSF	V222-22_1	10	0	0
G-CSF	V222-22_2	10	0	0
G-CSF	V222-23_1	10	0	0
G-CSF	V222-24_1	10	0	0
G-CSF	V301-18_1	10	1	0,6
G-CSF	V301-18_2	10	1	3,6
G-CSF	V301-18_3	10	0	0
G-CSF	V301-19_1	10	1	1
G-CSF	V301-19_2	10	1	1,7
G-CSF	V301-20_1	10	0	0
G-CSF	V301-20_2	10	1	2,2
G-CSF	V302-15_1	10	0	0

G-CSF	V302-15_2	10	1	0,5
G-CSF	V302-15_3	10	1	0,2
G-CSF	V302-16_1	10	1	1,2
G-CSF	V302-16_2	10	0	0
G-CSF	V302-16_3	10	0	0
G-CSF	V302-16_4	10	0	0
G-CSF	V302-17_1	10	0	0
G-CSF	V302-17_2	10	0	0
G-CSF	V302-17_3	10	0	0
G-CSF	V303-15_1	10	1	0,9
G-CSF	V303-15_2	10	1	0,8
G-CSF	V303-15_3	10	0	0
G-CSF	V303-16_1	10	0	0
G-CSF	V303-16_2	10	0	0
G-CSF	V303-16_3	10	1	2,3
G-CSF	V303-17_1	10	0	0

apical dendrite Sholl 20µm

Group	number	Radius(µm)	Intersections	Length(µm)
Control	V215-10_1	20	2	25,9
Control	V215-10_2	20	1	4,9
Control	V215-11_1	20	1	10,1
Control	V215-12_1	20	2	19,2
Control	V215-12_2	20	1	13,3
Control	V215-13_1	20	2	20
Control	V215-15_1	20	1	9,2
Control	V215-15_2	20	1	10,8
Control	V215-16_1	20	5	54,7
Control	V215-18_1	20	1	10,4
Control	V216-11_1	20	1	9,2
Control	V216-12_1	20	1	14,7
Control	V216-13_1	20	1	11,3
Control	V216-14_1	20	1	13,8
Control	V216-14_2	20	1	3,6
Control	V216-16_1	20	1	10,2
Control	V216-16_2	20	1	9,2
Control	V216-17_1	20	1	10,4
Control	V216-18_1	20	1	14,9
Control	V216-18_2	20	1	9,8
Control	V219-6_1	20	1	5,5
Control	V219-11_1	20	0	0
Control	V219-12_1	20	1	5,9
Control	V219-13_1	20	3	30,6
Control	V219-13_2	20	1	9,6
Control	V219-14_1	20	1	7,2
Control	V219-14_2	20	1	11,7
Control	V219-16_1	20	2	31,4
Control	V297-19_1	20	1	6,8
Control	V297-19_2	20	1	7,6
Control	V297-20_1	20	2	7
Control	V297-20_2	20	1	7,8
Control	V297-20_3	20	1	6,1
Control	V297-22_1	20	1	7,5
Control	V297-22_2	20	1	9,9

Control	V298-15_1	20	4	15,6
Control	V298-16_1	20	1	13,6
Control	V298-16_2	20	2	15
Control	V298-16_3	20	2	27,8
Control	V298-17_1	20	1	15,2
Control	V298-17_2	20	2	11,5
Control	V298-17_3	20	1	15,1
Control	V308-17_1	20	3	29,4
Control	V308-17_2	20	1	11,1
Control	V308-17_3	20	1	10,9
Control	V308-17_4	20	1	11
Control	V308-18_1	20	1	11,1
Control	V308-18_2	20	2	45,2
Control	V308-18_3	20	1	9,9
CIMT	V211-10_1	20	2	10,8
CIMT	V211-10_2	20	1	7,1
CIMT	V211-11_1	20	1	10,8
CIMT	V211-11_2	20	3	28,2
CIMT	V211-12_1	20	1	5
CIMT	V211-13_1	20	1	6,6
CIMT	V211-13_2	20	1	8,3
CIMT	V211-15_1	20	2	32,5
CIMT	V211-15_2	20	2	16,4
CIMT	V211-16_1	20	1	10,5
CIMT	V212-14_1	20	1	7,7
CIMT	V212-14_2	20	1	7,5
CIMT	V212-15_1	20	3	13,6
CIMT	V212-18_1	20	1	11,1
CIMT	V212-21_1	20	1	9,7
CIMT	V212-21_2	20	1	1,6
CIMT	V212-22_1	20	1	7,4
CIMT	V212-23_1	20	1	10,9
CIMT	V212-23_2	20	1	10,3
CIMT	V212-24_1	20	1	13,1
CIMT	V218-9_1	20	1	6,7
CIMT	V218-10_1	20	1	8,6
CIMT	V218-11_1	20	1	3,1
CIMT	V218-12_1	20	2	15,5
CIMT	V218-12_2	20	1	9,8
CIMT	V218-13_1	20	1	10,1
CIMT	V218-15_1	20	2	25,8
CIMT	V218-17_1	20	1	10,5
CIMT	V299-14_1	20	1	11,2
CIMT	V299-15_1	20	1	11
CIMT	V299-15_2	20	1	14,1
CIMT	V299-16_1	20	1	8,6
CIMT	V299-16_2	20	1	5,1
CIMT	V299-17_1	20	1	6
CIMT	V299-17_2	20	1	11,1
CIMT	V300-16_1	20	1	9,5
CIMT	V300-18_1	20	1	12,2
CIMT	V300-18_2	20	1	10,4
CIMT	V300-20_1	20	1	10,6
CIMT	V300-22_1	20	1	10,2
CIMT	V300-23_1	20	2	40,4
CIMT	V300-23_2	20	1	10,8

CIMT	V309-15_1	20	3	29,3
CIMT	V309-15_2	20	1	4,3
CIMT	V309-16_1	20	1	10,6
CIMT	V309-16_2	20	1	11,5
CIMT	V309-16_3	20	1	10,1
CIMT	V309-17_1	20	2	30,3
CIMT	V309-17_2	20	1	11,1
CIMT+G-CSF	V213-13_1	20	1	12,2
CIMT+G-CSF	V213-16_1	20	2	16,3
CIMT+G-CSF	V213-17_1	20	1	10,1
CIMT+G-CSF	V213-17_2	20	2	41,7
CIMT+G-CSF	V213-18_1	20	1	12,4
CIMT+G-CSF	V213-18_2	20	2	45,6
CIMT+G-CSF	V213-19_1	20	1	11,7
CIMT+G-CSF	V213-19_2	20	1	10,5
CIMT+G-CSF	V213-20_1	20	1	10,1
CIMT+G-CSF	V213-22_1	20	1	11,1
CIMT+G-CSF	V213-23_1	20	1	10,1
CIMT+G-CSF	V214-14_1	20	0	0
CIMT+G-CSF	V214-17_1	20	0	0
CIMT+G-CSF	V214-18_1	20	1	8,1
CIMT+G-CSF	V214-19_1	20	1	10
CIMT+G-CSF	V214-19_2	20	1	10,2
CIMT+G-CSF	V214-20_1	20	1	8,6
CIMT+G-CSF	V214-21_1	20	1	8,4
CIMT+G-CSF	V217-1_1	20	1	2,5
CIMT+G-CSF	V217-2_1	20	1	7,8
CIMT+G-CSF	V217-4_1	20	1	4,1
CIMT+G-CSF	V217-6_1	20	1	10,5
CIMT+G-CSF	V217-6_3	20	1	11,5
CIMT+G-CSF	V217-8_1	20	1	15
CIMT+G-CSF	V217-8_4	20	1	3,3
CIMT+G-CSF	V217-10_1	20	1	8,3
CIMT+G-CSF	V217-11_1	20	2	27,2
CIMT+G-CSF	V304-16_1	20	1	13,8
CIMT+G-CSF	V304-17_1	20	2	35,4
CIMT+G-CSF	V304-17_2	20	1	10,6
CIMT+G-CSF	V304-17_3	20	1	17,1
CIMT+G-CSF	V304-17_4	20	1	13,6
CIMT+G-CSF	V304-19_1	20	1	11,4
CIMT+G-CSF	V304-19_2	20	1	16,3
CIMT+G-CSF	V305-17_1	20	1	9,5
CIMT+G-CSF	V305-17_2	20	2	30,3
CIMT+G-CSF	V305-17_3	20	1	12,1
CIMT+G-CSF	V305-18_1	20	1	11,7
CIMT+G-CSF	V305-18_2	20	1	12,4
CIMT+G-CSF	V305-18_3	20	1	11,1
CIMT+G-CSF	V305-18_4	20	2	12
CIMT+G-CSF	V305-19_1	20	1	12,6
CIMT/G-CSF	V306-16_1	20	1	10,3
CIMT/G-CSF	V306-16_2	20	2	19,4
CIMT/G-CSF	V306-17_1	20	1	12
CIMT/G-CSF	V306-17_2	20	1	10,3
CIMT/G-CSF	V306-17_3	20	1	8,7
CIMT/G-CSF	V306-17_4	20	1	12,9
CIMT/G-CSF	V306-17_5	20	1	10,4

CIMT/G-CSF	V311-16_1	20	1	7
CIMT/G-CSF	V311-17_1	20	1	7
CIMT/G-CSF	V311-17_2	20	1	10,8
CIMT/G-CSF	V311-19_1	20	1	11,8
CIMT/G-CSF	V311-20_1	20	1	6,9
CIMT/G-CSF	V311-20_2	20	1	7,7
CIMT/G-CSF	V311-20_3	20	2	10,1
CIMT/G-CSF	V313-14_1	20	3	29,6
CIMT/G-CSF	V313-15_1	20	1	9,8
CIMT/G-CSF	V313-15_2	20	1	11,3
CIMT/G-CSF	V313-17_1	20	2	13
CIMT/G-CSF	V313-17_2	20	3	25,9
CIMT/G-CSF	V313-17_3	20	1	12,7
CIMT/G-CSF	V313-19_1	20	1	10,6
CIMT/G-CSF	V314-16_1	20	4	51,9
CIMT/G-CSF	V314-16_2	20	1	6,8
CIMT/G-CSF	V314-17_1	20	1	8,9
CIMT/G-CSF	V314-17_2	20	1	10,1
CIMT/G-CSF	V314-17_3	20	1	35,5
CIMT/G-CSF	V314-18_1	20	1	15,2
CIMT/G-CSF	V314-18_2	20	1	9,3
CIMT/G-CSF	V315-11_1	20	3	30,2
CIMT/G-CSF	V315-11_2	20	1	7,4
CIMT/G-CSF	V315-13_1	20	1	7,3
CIMT/G-CSF	V315-13_2	20	2	8,3
CIMT/G-CSF	V315-14_1	20	1	9,7
CIMT/G-CSF	V315-14_2	20	2	12,1
CIMT/G-CSF	V315-14_3	20	1	1
G-CSF	V220-9_1	20	1	10,9
G-CSF	V220-11_1	20	1	10,2
G-CSF	V220-12_1	20	1	9,1
G-CSF	V220-16_1	20	1	10,2
G-CSF	V220-16_2	20	1	13,8
G-CSF	V220-17_1	20	1	8
G-CSF	V220-18_1	20	1	11,5
G-CSF	V221-2_1	20	1	7,6
G-CSF	V221-4_1	20	1	9,9
G-CSF	V221-5_1	20	1	8,1
G-CSF	V221-6_1	20	1	10,4
G-CSF	V221-7_1	20	1	10,2
G-CSF	V221-7_2	20	1	8,6
G-CSF	V221-11_1	20	1	6,9
G-CSF	V221-13_1	20	1	10,1
G-CSF	V221-15_1	20	1	9,2
G-CSF	V221-20_1	20	1	12,1
G-CSF	V222-19_1	20	1	10,4
G-CSF	V222-19_2	20	1	6,1
G-CSF	V222-20_1	20	1	8,3
G-CSF	V222-20_2	20	1	9,8
G-CSF	V222-21_1	20	1	8,6
G-CSF	V222-22_1	20	2	23,9
G-CSF	V222-22_2	20	1	1,8
G-CSF	V222-23_1	20	2	4,1
G-CSF	V222-24_1	20	1	5,2
G-CSF	V301-18_1	20	1	14,7
G-CSF	V301-18_2	20	1	11,4

G-CSF	V301-18_3	20	1	13,7
G-CSF	V301-19_1	20	1	10,1
G-CSF	V301-19_2	20	1	10,5
G-CSF	V301-20_1	20	2	18,1
G-CSF	V301-20_2	20	1	10,5
G-CSF	V302-15_1	20	1	11
G-CSF	V302-15_2	20	1	12,4
G-CSF	V302-15_3	20	1	10,4
G-CSF	V302-16_1	20	2	23,1
G-CSF	V302-16_2	20	1	14,5
G-CSF	V302-16_3	20	1	15,2
G-CSF	V302-16_4	20	1	1
G-CSF	V302-17_1	20	1	10,8
G-CSF	V302-17_2	20	1	9,6
G-CSF	V302-17_3	20	1	9,7
G-CSF	V303-15_1	20	1	12
G-CSF	V303-15_2	20	2	29
G-CSF	V303-15_3	20	1	12
G-CSF	V303-16_1	20	2	13
G-CSF	V303-16_2	20	1	8
G-CSF	V303-16_3	20	1	18,3
G-CSF	V303-17_1	20	1	7,1

apical dendrite Sholl 30µm

Group	number	Radius(µm)	Intersections	Length(µm)
Control	V215-10_1	30	2	25,5
Control	V215-10_2	30	1	10,4
Control	V215-11_1	30	1	11
Control	V215-12_1	30	2	34
Control	V215-12_2	30	2	17,5
Control	V215-13_1	30	2	24,8
Control	V215-15_1	30	5	24,2
Control	V215-15_2	30	2	21
Control	V215-16_1	30	5	77,7
Control	V215-18_1	30	2	38
Control	V216-11_1	30	2	22,5
Control	V216-12_1	30	1	11,3
Control	V216-13_1	30	1	13,2
Control	V216-14_1	30	1	10,9
Control	V216-14_2	30	1	12,5
Control	V216-16_1	30	2	38,1
Control	V216-16_2	30	2	31,8
Control	V216-17_1	30	2	12
Control	V216-18_1	30	1	11,4
Control	V216-18_2	30	1	11,5
Control	V219-6_1	30	1	11
Control	V219-11_1	30	1	9,8
Control	V219-12_1	30	1	10,5
Control	V219-13_1	30	4	61,8
Control	V219-13_2	30	1	11,7
Control	V219-14_1	30	1	14,3
Control	V219-14_2	30	1	10,3
Control	V219-16_1	30	4	82
Control	V297-19_1	30	1	14,2

Control	V297-19_2	30	1	10,3
Control	V297-20_1	30	2	33,4
Control	V297-20_2	30	1	10,5
Control	V297-20_3	30	2	21,5
Control	V297-22_1	30	2	25
Control	V297-22_2	30	1	10,9
Control	V298-15_1	30	2	33,9
Control	V298-16_1	30	2	16,3
Control	V298-16_2	30	3	23
Control	V298-16_3	30	4	35,3
Control	V298-17_1	30	2	15,1
Control	V298-17_2	30	2	23,9
Control	V298-17_3	30	1	10,6
Control	V308-17_1	30	4	64,3
Control	V308-17_2	30	1	10,6
Control	V308-17_3	30	1	11
Control	V308-17_4	30	2	38,5
Control	V308-18_1	30	2	18
Control	V308-18_2	30	2	27,5
Control	V308-18_3	30	5	46
CIMT	V211-10_1	30	2	25,9
CIMT	V211-10_2	30	1	14,1
CIMT	V211-11_1	30	1	11,8
CIMT	V211-11_2	30	3	35
CIMT	V211-12_1	30	1	12,4
CIMT	V211-13_1	30	2	12,2
CIMT	V211-13_2	30	2	19
CIMT	V211-15_1	30	2	23,9
CIMT	V211-15_2	30	2	40,7
CIMT	V211-16_1	30	1	11
CIMT	V212-14_1	30	1	11,5
CIMT	V212-14_2	30	3	39,1
CIMT	V212-15_1	30	4	47,8
CIMT	V212-18_1	30	3	49,1
CIMT	V212-21_1	30	1	11,3
CIMT	V212-21_2	30	1	11,6
CIMT	V212-22_1	30	1	11,9
CIMT	V212-23_1	30	1	11,8
CIMT	V212-23_2	30	1	10,9
CIMT	V212-24_1	30	1	10,7
CIMT	V218-9_1	30	1	10,1
CIMT	V218-10_1	30	3	41
CIMT	V218-11_1	30	1	10,9
CIMT	V218-12_1	30	2	26,5
CIMT	V218-12_2	30	2	25,3
CIMT	V218-13_1	30	1	12,1
CIMT	V218-15_1	30	2	31,8
CIMT	V218-17_1	30	2	35
CIMT	V299-14_1	30	4	56,2
CIMT	V299-15_1	30	1	10,4
CIMT	V299-15_2	30	1	10,4
CIMT	V299-16_1	30	1	12,2
CIMT	V299-16_2	30	1	11,8
CIMT	V299-17_1	30	2	13,7
CIMT	V299-17_2	30	3	34,6
CIMT	V300-16_1	30	2	21,4

CIMT	V300-18_1	30	1	10,4
CIMT	V300-18_2	30	1	10,3
CIMT	V300-20_1	30	1	10,1
CIMT	V300-22_1	30	1	10,8
CIMT	V300-23_1	30	3	26,4
CIMT	V300-23_2	30	1	10,6
CIMT	V309-15_1	30	4	66,6
CIMT	V309-15_2	30	1	10,5
CIMT	V309-16_1	30	1	12
CIMT	V309-16_2	30	2	84,8
CIMT	V309-16_3	30	2	36,6
CIMT	V309-17_1	30	2	23,7
CIMT	V309-17_2	30	2	33,5
CIMT+G-CSF	V213-13_1	30	1	10,9
CIMT+G-CSF	V213-16_1	30	2	25,4
CIMT+G-CSF	V213-17_1	30	1	10,2
CIMT+G-CSF	V213-17_2	30	3	31,9
CIMT+G-CSF	V213-18_1	30	2	30,8
CIMT+G-CSF	V213-18_2	30	2	24,8
CIMT+G-CSF	V213-19_1	30	2	21
CIMT+G-CSF	V213-19_2	30	1	10,7
CIMT+G-CSF	V213-20_1	30	1	13,1
CIMT+G-CSF	V213-22_1	30	1	10,1
CIMT+G-CSF	V213-23_1	30	1	11,6
CIMT+G-CSF	V214-14_1	30	0	0
CIMT+G-CSF	V214-17_1	30	2	7,8
CIMT+G-CSF	V214-18_1	30	1	10,6
CIMT+G-CSF	V214-19_1	30	1	10,1
CIMT+G-CSF	V214-19_2	30	2	25,2
CIMT+G-CSF	V214-20_1	30	2	34,8
CIMT+G-CSF	V214-21_1	30	1	10,9
CIMT+G-CSF	V217-1_1	30	1	11,3
CIMT+G-CSF	V217-2_1	30	0	3,5
CIMT+G-CSF	V217-4_1	30	1	14,4
CIMT+G-CSF	V217-6_1	30	1	13,4
CIMT+G-CSF	V217-6_3	30	5	61,7
CIMT+G-CSF	V217-8_1	30	1	13,4
CIMT+G-CSF	V217-8_4	30	2	37,8
CIMT+G-CSF	V217-10_1	30	1	10,2
CIMT+G-CSF	V217-11_1	30	2	24,4
CIMT+G-CSF	V304-16_1	30	1	11,7
CIMT+G-CSF	V304-17_1	30	1	10,7
CIMT+G-CSF	V304-17_2	30	1	10,9
CIMT+G-CSF	V304-17_3	30	2	39,2
CIMT+G-CSF	V304-17_4	30	1	13,1
CIMT+G-CSF	V304-19_1	30	1	11,3
CIMT+G-CSF	V304-19_2	30	1	13,5
CIMT+G-CSF	V305-17_1	30	1	11,7
CIMT+G-CSF	V305-17_2	30	2	30,3
CIMT+G-CSF	V305-17_3	30	1	10,7
CIMT+G-CSF	V305-18_1	30	1	10,5
CIMT+G-CSF	V305-18_2	30	2	13,6
CIMT+G-CSF	V305-18_3	30	1	10,9
CIMT+G-CSF	V305-18_4	30	2	30,8
CIMT+G-CSF	V305-19_1	30	1	12
CIMT/G-CSF	V306-16_1	30	1	10,2

CIMT/G-CSF	V306-16_2	30	2	24,2
CIMT/G-CSF	V306-17_1	30	1	10,6
CIMT/G-CSF	V306-17_2	30	3	47,7
CIMT/G-CSF	V306-17_3	30	1	11,7
CIMT/G-CSF	V306-17_4	30	1	10,3
CIMT/G-CSF	V306-17_5	30	1	13,2
CIMT/G-CSF	V311-16_1	30	1	13,7
CIMT/G-CSF	V311-17_1	30	1	11,9
CIMT/G-CSF	V311-17_2	30	1	11,3
CIMT/G-CSF	V311-19_1	30	1	14,1
CIMT/G-CSF	V311-20_1	30	1	11,6
CIMT/G-CSF	V311-20_2	30	3	43,3
CIMT/G-CSF	V311-20_3	30	3	31,8
CIMT/G-CSF	V313-14_1	30	3	38,1
CIMT/G-CSF	V313-15_1	30	1	10,4
CIMT/G-CSF	V313-15_2	30	1	12,2
CIMT/G-CSF	V313-17_1	30	2	34,6
CIMT/G-CSF	V313-17_2	30	4	59,8
CIMT/G-CSF	V313-17_3	30	2	17,3
CIMT/G-CSF	V313-19_1	30	1	13,4
CIMT/G-CSF	V314-16_1	30	3	68,7
CIMT/G-CSF	V314-16_2	30	1	10,3
CIMT/G-CSF	V314-17_1	30	2	27,9
CIMT/G-CSF	V314-17_2	30	2	10,7
CIMT/G-CSF	V314-17_3	30	1	10,7
CIMT/G-CSF	V314-18_1	30	1	10,2
CIMT/G-CSF	V314-18_2	30	2	18,4
CIMT/G-CSF	V315-11_1	30	3	32,9
CIMT/G-CSF	V315-11_2	30	2	15,5
CIMT/G-CSF	V315-13_1	30	1	10,4
CIMT/G-CSF	V315-13_2	30	3	37,5
CIMT/G-CSF	V315-14_1	30	1	10,6
CIMT/G-CSF	V315-14_2	30	3	28,6
CIMT/G-CSF	V315-14_3	30	1	20,2
G-CSF	V220-9_1	30	1	10,2
G-CSF	V220-11_1	30	1	16,8
G-CSF	V220-12_1	30	2	28
G-CSF	V220-16_1	30	1	13,1
G-CSF	V220-16_2	30	3	62,8
G-CSF	V220-17_1	30	2	16,7
G-CSF	V220-18_1	30	1	11,3
G-CSF	V221-2_1	30	1	10,2
G-CSF	V221-4_1	30	1	10,7
G-CSF	V221-5_1	30	3	35
G-CSF	V221-6_1	30	2	22,1
G-CSF	V221-7_1	30	2	21,8
G-CSF	V221-7_2	30	1	11,5
G-CSF	V221-11_1	30	1	10,2
G-CSF	V221-13_1	30	3	48,4
G-CSF	V221-15_1	30	1	10,1
G-CSF	V221-20_1	30	1	10,1
G-CSF	V222-19_1	30	1	11,6
G-CSF	V222-19_2	30	1	11,3
G-CSF	V222-20_1	30	1	10,8
G-CSF	V222-20_2	30	1	10,1
G-CSF	V222-21_1	30	1	10,1

G-CSF	V222-22_1	30	3	57,7
G-CSF	V222-22_2	30	1	10,2
G-CSF	V222-23_1	30	2	26,1
G-CSF	V222-24_1	30	1	12,3
G-CSF	V301-18_1	30	1	12,1
G-CSF	V301-18_2	30	1	12,5
G-CSF	V301-18_3	30	1	11,2
G-CSF	V301-19_1	30	3	36,3
G-CSF	V301-19_2	30	2	23,8
G-CSF	V301-20_1	30	4	72,1
G-CSF	V301-20_2	30	1	10,1
G-CSF	V302-15_1	30	1	10,6
G-CSF	V302-15_2	30	1	12,4
G-CSF	V302-15_3	30	1	10,3
G-CSF	V302-16_1	30	3	34,7
G-CSF	V302-16_2	30	1	10,3
G-CSF	V302-16_3	30	1	11,9
G-CSF	V302-16_4	30	1	10,7
G-CSF	V302-17_1	30	1	11,5
G-CSF	V302-17_2	30	2	17,8
G-CSF	V302-17_3	30	1	10,3
G-CSF	V303-15_1	30	1	13,3
G-CSF	V303-15_2	30	5	36,5
G-CSF	V303-15_3	30	1	13,3
G-CSF	V303-16_1	30	3	28,3
G-CSF	V303-16_2	30	2	14
G-CSF	V303-16_3	30	1	10,9
G-CSF	V303-17_1	30	1	11,4

apical dendrite Sholl 40μm

Group	number	Radius(μm)	Intersections	Length(μm)
Control	V215-10_1	40	2	36,3
Control	V215-10_2	40	1	11,4
Control	V215-11_1	40	1	11,5
Control	V215-12_1	40	1	30,6
Control	V215-12_2	40	2	30,5
Control	V215-13_1	40	2	25,3
Control	V215-15_1	40	3	84,9
Control	V215-15_2	40	2	24,1
Control	V215-16_1	40	5	90,7
Control	V215-18_1	40	2	31,2
Control	V216-11_1	40	2	25,7
Control	V216-12_1	40	2	23,3
Control	V216-13_1	40	1	13,1
Control	V216-14_1	40	2	34,6
Control	V216-14_2	40	1	12
Control	V216-16_1	40	2	21,6
Control	V216-16_2	40	2	47,2
Control	V216-17_1	40	2	29,6
Control	V216-18_1	40	1	10,1
Control	V216-18_2	40	1	10,9
Control	V219-6_1	40	1	12,8
Control	V219-11_1	40	1	11,2
Control	V219-12_1	40	1	10,2

Control	V219-13_1	40	4	50,4
Control	V219-13_2	40	1	14,8
Control	V219-14_1	40	2	24,5
Control	V219-14_2	40	2	28,1
Control	V219-16_1	40	4	80,7
Control	V297-19_1	40	1	10,4
Control	V297-19_2	40	2	27
Control	V297-20_1	40	1	15,8
Control	V297-20_2	40	2	32,5
Control	V297-20_3	40	1	39,8
Control	V297-22_1	40	5	77,8
Control	V297-22_2	40	2	25,3
Control	V298-15_1	40	1	20,5
Control	V298-16_1	40	3	41,4
Control	V298-16_2	40	2	36,6
Control	V298-16_3	40	5	57,2
Control	V298-17_1	40	2	25,9
Control	V298-17_2	40	2	24,1
Control	V298-17_3	40	1	10,2
Control	V308-17_1	40	4	60,6
Control	V308-17_2	40	1	10,3
Control	V308-17_3	40	2	33,2
Control	V308-17_4	40	2	29,8
Control	V308-18_1	40	2	27,7
Control	V308-18_2	40	3	43,5
Control	V308-18_3	40	5	68,4
CIMT	V211-10_1	40	2	24
CIMT	V211-10_2	40	1	13,1
CIMT	V211-11_1	40	1	11,4
CIMT	V211-11_2	40	2	38,6
CIMT	V211-12_1	40	1	10,6
CIMT	V211-13_1	40	3	35,9
CIMT	V211-13_2	40	7	67,2
CIMT	V211-15_1	40	2	32,3
CIMT	V211-15_2	40	2	23,5
CIMT	V211-16_1	40	1	11,2
CIMT	V212-14_1	40	1	10,2
CIMT	V212-14_2	40	4	45,4
CIMT	V212-15_1	40	4	62,6
CIMT	V212-18_1	40	3	40,6
CIMT	V212-21_1	40	1	10,2
CIMT	V212-21_2	40	2	32,2
CIMT	V212-22_1	40	2	16,2
CIMT	V212-23_1	40	1	10
CIMT	V212-23_2	40	1	10,1
CIMT	V212-24_1	40	2	25,2
CIMT	V218-9_1	40	2	39,2
CIMT	V218-10_1	40	3	69,3
CIMT	V218-11_1	40	2	26,2
CIMT	V218-12_1	40	4	49,9
CIMT	V218-12_2	40	3	46,6
CIMT	V218-13_1	40	1	13,7
CIMT	V218-15_1	40	4	37,4
CIMT	V218-17_1	40	2	22,2
CIMT	V299-14_1	40	3	70,3
CIMT	V299-15_1	40	1	10,2

CIMT	V299-15_2	40	1	10,5
CIMT	V299-16_1	40	3	22,2
CIMT	V299-16_2	40	1	10,7
CIMT	V299-17_1	40	4	38,6
CIMT	V299-17_2	40	3	53,3
CIMT	V300-16_1	40	2	32,8
CIMT	V300-18_1	40	1	10,6
CIMT	V300-18_2	40	1	11,7
CIMT	V300-20_1	40	1	10,1
CIMT	V300-22_1	40	1	10,4
CIMT	V300-23_1	40	3	32,7
CIMT	V300-23_2	40	3	40,1
CIMT	V309-15_1	40	4	81,8
CIMT	V309-15_2	40	3	29,3
CIMT	V309-16_1	40	2	15,2
CIMT	V309-16_2	40	3	33,6
CIMT	V309-16_3	40	3	44,1
CIMT	V309-17_1	40	2	30,9
CIMT	V309-17_2	40	3	41,8
CIMT+G-CSF	V213-13_1	40	2	19,9
CIMT+G-CSF	V213-16_1	40	2	24,2
CIMT+G-CSF	V213-17_1	40	1	10,1
CIMT+G-CSF	V213-17_2	40	5	51,9
CIMT+G-CSF	V213-18_1	40	2	25,9
CIMT+G-CSF	V213-18_2	40	2	29
CIMT+G-CSF	V213-19_1	40	2	32,4
CIMT+G-CSF	V213-19_2	40	1	11,4
CIMT+G-CSF	V213-20_1	40	1	21
CIMT+G-CSF	V213-22_1	40	1	11
CIMT+G-CSF	V213-23_1	40	1	10,7
CIMT+G-CSF	V214-14_1	40	1	13,5
CIMT+G-CSF	V214-17_1	40	4	67,1
CIMT+G-CSF	V214-18_1	40	1	10,6
CIMT+G-CSF	V214-19_1	40	1	10,1
CIMT+G-CSF	V214-19_2	40	2	23,7
CIMT+G-CSF	V214-20_1	40	2	25,1
CIMT+G-CSF	V214-21_1	40	1	10,4
CIMT+G-CSF	V217-1_1	40	1	10,3
CIMT+G-CSF	V217-4_1	40	1	13,2
CIMT+G-CSF	V217-6_1	40	1	15,3
CIMT+G-CSF	V217-6_3	40	3	55,1
CIMT+G-CSF	V217-8_1	40	2	23,7
CIMT+G-CSF	V217-8_4	40	2	25,4
CIMT+G-CSF	V217-10_1	40	1	12,3
CIMT+G-CSF	V217-11_1	40	2	22,1
CIMT+G-CSF	V304-16_1	40	1	14
CIMT+G-CSF	V304-17_1	40	1	10,2
CIMT+G-CSF	V304-17_2	40	1	15,7
CIMT+G-CSF	V304-17_3	40	4	37
CIMT+G-CSF	V304-17_4	40	1	10,6
CIMT+G-CSF	V304-19_1	40	1	10,1
CIMT+G-CSF	V304-19_2	40	1	11
CIMT+G-CSF	V305-17_1	40	1	11,5
CIMT+G-CSF	V305-17_2	40	2	28,2
CIMT+G-CSF	V305-17_3	40	2	29,5
CIMT+G-CSF	V305-18_1	40	1	11,1

CIMT+G-CSF	V305-18_2	40	2	28
CIMT+G-CSF	V305-18_3	40	1	10,5
CIMT+G-CSF	V305-18_4	40	4	53,3
CIMT+G-CSF	V305-19_1	40	2	35,5
CIMT/G-CSF	V306-16_1	40	1	12,2
CIMT/G-CSF	V306-16_2	40	5	82,5
CIMT/G-CSF	V306-17_1	40	2	44,1
CIMT/G-CSF	V306-17_2	40	3	54,6
CIMT/G-CSF	V306-17_3	40	1	10,7
CIMT/G-CSF	V306-17_4	40	1	15,1
CIMT/G-CSF	V306-17_5	40	1	11,9
CIMT/G-CSF	V311-16_1	40	2	32,4
CIMT/G-CSF	V311-17_1	40	1	10,7
CIMT/G-CSF	V311-17_2	40	1	11,1
CIMT/G-CSF	V311-19_1	40	1	10,2
CIMT/G-CSF	V311-20_1	40	1	10,4
CIMT/G-CSF	V311-20_2	40	4	57,6
CIMT/G-CSF	V311-20_3	40	3	34,7
CIMT/G-CSF	V313-14_1	40	6	78,5
CIMT/G-CSF	V313-15_1	40	1	10,4
CIMT/G-CSF	V313-15_2	40	1	13
CIMT/G-CSF	V313-17_1	40	1	20,8
CIMT/G-CSF	V313-17_2	40	2	38,9
CIMT/G-CSF	V313-17_3	40	2	23,5
CIMT/G-CSF	V313-19_1	40	1	10,2
CIMT/G-CSF	V314-16_1	40	2	30,2
CIMT/G-CSF	V314-16_2	40	1	10,4
CIMT/G-CSF	V314-17_1	40	4	47,7
CIMT/G-CSF	V314-17_2	40	3	23,2
CIMT/G-CSF	V314-17_3	40	1	11,2
CIMT/G-CSF	V314-18_1	40	1	10,1
CIMT/G-CSF	V314-18_2	40	3	34,7
CIMT/G-CSF	V315-11_1	40	2	49,9
CIMT/G-CSF	V315-11_2	40	2	36,5
CIMT/G-CSF	V315-13_1	40	1	11,2
CIMT/G-CSF	V315-13_2	40	3	34,7
CIMT/G-CSF	V315-14_1	40	1	11,1
CIMT/G-CSF	V315-14_2	40	2	35,5
CIMT/G-CSF	V315-14_3	40	1	10,8
G-CSF	V220-9_1	40	1	12,4
G-CSF	V220-11_1	40	1	10,6
G-CSF	V220-12_1	40	2	27,7
G-CSF	V220-16_1	40	1	13,5
G-CSF	V220-16_2	40	2	43,2
G-CSF	V220-17_1	40	4	63,4
G-CSF	V220-18_1	40	1	11,1
G-CSF	V221-2_1	40	0	0,1
G-CSF	V221-4_1	40	1	10,8
G-CSF	V221-5_1	40	3	35,9
G-CSF	V221-6_1	40	2	24,5
G-CSF	V221-7_1	40	3	27
G-CSF	V221-7_2	40	2	34
G-CSF	V221-11_1	40	1	10,6
G-CSF	V221-13_1	40	2	26,9
G-CSF	V221-15_1	40	2	38,6
G-CSF	V221-20_1	40	2	58,3

G-CSF	V222-19_1	40	1	15,2
G-CSF	V222-19_2	40	1	10,3
G-CSF	V222-20_1	40	3	70,1
G-CSF	V222-20_2	40	1	13
G-CSF	V222-21_1	40	3	28,1
G-CSF	V222-22_1	40	3	34,1
G-CSF	V222-22_2	40	1	10,2
G-CSF	V222-23_1	40	3	54,2
G-CSF	V222-24_1	40	3	68,3
G-CSF	V301-18_1	40	1	10,5
G-CSF	V301-18_2	40	1	11,3
G-CSF	V301-18_3	40	1	10,7
G-CSF	V301-19_1	40	5	45,5
G-CSF	V301-19_2	40	2	25,2
G-CSF	V301-20_1	40	4	51,8
G-CSF	V301-20_2	40	2	39,7
G-CSF	V302-15_1	40	1	12,1
G-CSF	V302-15_2	40	1	14,6
G-CSF	V302-15_3	40	4	45,2
G-CSF	V302-16_1	40	3	41,8
G-CSF	V302-16_2	40	1	13,1
G-CSF	V302-16_3	40	1	10,9
G-CSF	V302-16_4	40	1	11,1
G-CSF	V302-17_1	40	2	22,9
G-CSF	V302-17_2	40	3	24,9
G-CSF	V302-17_3	40	1	12,2
G-CSF	V303-15_1	40	1	12,1
G-CSF	V303-15_2	40	5	127
G-CSF	V303-15_3	40	1	10,8
G-CSF	V303-16_1	40	3	40,2
G-CSF	V303-16_2	40	2	43,2
G-CSF	V303-16_3	40	3	70
G-CSF	V303-17_1	40	1	10,4

apical dendrite Sholl 50µm

Group	number	Radius(µm)	Intersections	Length(µm)
Control	V215-10_1	50	2	23,4
Control	V215-10_2	50	0	3,8
Control	V215-11_1	50	1	10,9
Control	V215-12_1	50	1	11,1
Control	V215-12_2	50	2	41,9
Control	V215-13_1	50	2	22,1
Control	V215-15_1	50	4	75,1
Control	V215-15_2	50	2	22,4
Control	V215-16_1	50	5	59,8
Control	V215-18_1	50	2	33,4
Control	V216-11_1	50	3	26,2
Control	V216-12_1	50	2	27,5
Control	V216-13_1	50	1	11,9
Control	V216-14_1	50	2	26,1
Control	V216-14_2	50	1	11,6
Control	V216-16_1	50	1	13,4
Control	V216-16_2	50	3	37,5
Control	V216-17_1	50	4	40

Control	V216-18_1	50	1	10,2
Control	V216-18_2	50	1	10,7
Control	V219-6_1	50	1	11,3
Control	V219-11_1	50	1	10,2
Control	V219-12_1	50	1	10,2
Control	V219-13_1	50	7	92,6
Control	V219-13_2	50	1	12,3
Control	V219-14_1	50	2	30,7
Control	V219-14_2	50	7	66,7
Control	V219-16_1	50	4	69,2
Control	V297-19_1	50	2	33,7
Control	V297-19_2	50	2	50,7
Control	V297-20_1	50	2	19,4
Control	V297-20_2	50	2	27,3
Control	V297-20_3	50	1	10,1
Control	V297-22_1	50	9	117,9
Control	V297-22_2	50	2	49,8
Control	V298-15_1	50	4	65,4
Control	V298-16_1	50	3	49,7
Control	V298-16_2	50	3	54
Control	V298-16_3	50	3	71,5
Control	V298-17_1	50	2	24,3
Control	V298-17_2	50	2	21,1
Control	V298-17_3	50	1	11,3
Control	V308-17_1	50	3	58,2
Control	V308-17_2	50	2	33,8
Control	V308-17_3	50	3	41,1
Control	V308-17_4	50	3	53,2
Control	V308-18_1	50	2	34
Control	V308-18_2	50	5	45,8
Control	V308-18_3	50	6	86,5
CIMT	V211-10_1	50	4	66,8
CIMT	V211-10_2	50	1	10,6
CIMT	V211-11_1	50	1	10,6
CIMT	V211-11_2	50	1	18,7
CIMT	V211-12_1	50	1	10,2
CIMT	V211-13_1	50	4	48,7
CIMT	V211-13_2	50	4	84,5
CIMT	V211-15_1	50	2	22,7
CIMT	V211-15_2	50	2	21,8
CIMT	V211-16_1	50	1	12,9
CIMT	V212-14_1	50	2	17
CIMT	V212-14_2	50	6	86,6
CIMT	V212-15_1	50	6	59,3
CIMT	V212-18_1	50	3	32,8
CIMT	V212-21_1	50	1	10,4
CIMT	V212-21_2	50	3	39,7
CIMT	V212-22_1	50	2	25,2
CIMT	V212-23_1	50	2	35,1
CIMT	V212-23_2	50	2	42,3
CIMT	V212-24_1	50	3	27,1
CIMT	V218-9_1	50	1	14,3
CIMT	V218-10_1	50	4	63,8
CIMT	V218-11_1	50	1	24
CIMT	V218-12_1	50	6	88,3
CIMT	V218-12_2	50	3	50,5

CIMT	V218-13_1	50	1	11
CIMT	V218-15_1	50	10	128,5
CIMT	V218-17_1	50	3	35,2
CIMT	V299-14_1	50	4	65,7
CIMT	V299-15_1	50	1	10,6
CIMT	V299-15_2	50	1	13
CIMT	V299-16_1	50	3	88,4
CIMT	V299-16_2	50	1	10,6
CIMT	V299-17_1	50	5	71,4
CIMT	V299-17_2	50	3	51,5
CIMT	V300-16_1	50	2	23,1
CIMT	V300-18_1	50	1	10,1
CIMT	V300-18_2	50	1	11
CIMT	V300-20_1	50	1	11,3
CIMT	V300-22_1	50	1	10,5
CIMT	V300-23_1	50	4	60,8
CIMT	V300-23_2	50	5	69,9
CIMT	V309-15_1	50	5	59,8
CIMT	V309-15_2	50	3	36,4
CIMT	V309-16_1	50	2	65,7
CIMT	V309-16_2	50	2	33,4
CIMT	V309-16_3	50	5	112,7
CIMT	V309-17_1	50	3	68,7
CIMT	V309-17_2	50	2	23,3
CIMT+G-CSF	V213-13_1	50	2	23,2
CIMT+G-CSF	V213-16_1	50	3	30,6
CIMT+G-CSF	V213-17_1	50	2	15,4
CIMT+G-CSF	V213-17_2	50	5	70,2
CIMT+G-CSF	V213-18_1	50	4	46,2
CIMT+G-CSF	V213-18_2	50	3	44,7
CIMT+G-CSF	V213-19_1	50	2	32,5
CIMT+G-CSF	V213-19_2	50	1	13,1
CIMT+G-CSF	V213-20_1	50	4	31,8
CIMT+G-CSF	V213-22_1	50	2	24,9
CIMT+G-CSF	V213-23_1	50	1	13
CIMT+G-CSF	V214-14_1	50	1	14
CIMT+G-CSF	V214-17_1	50	2	43
CIMT+G-CSF	V214-18_1	50	1	10,3
CIMT+G-CSF	V214-19_1	50	3	53
CIMT+G-CSF	V214-19_2	50	2	21,6
CIMT+G-CSF	V214-20_1	50	2	25,3
CIMT+G-CSF	V214-21_1	50	1	10,9
CIMT+G-CSF	V217-1_1	50	1	10,5
CIMT+G-CSF	V217-4_1	50	1	12,4
CIMT+G-CSF	V217-6_1	50	3	15,1
CIMT+G-CSF	V217-6_3	50	3	47,4
CIMT+G-CSF	V217-8_1	50	3	49,5
CIMT+G-CSF	V217-8_4	50	3	50,7
CIMT+G-CSF	V217-10_1	50	1	10
CIMT+G-CSF	V217-11_1	50	2	20,8
CIMT+G-CSF	V304-16_1	50	0	12,9
CIMT+G-CSF	V304-17_1	50	1	13,4
CIMT+G-CSF	V304-17_2	50	1	11,1
CIMT+G-CSF	V304-17_3	50	4	52,2
CIMT+G-CSF	V304-17_4	50	1	10,8
CIMT+G-CSF	V304-19_1	50	1	11,3

CIMT+G-CSF	V304-19_2	50	2	20,8
CIMT+G-CSF	V305-17_1	50	3	55,4
CIMT+G-CSF	V305-17_2	50	2	22,9
CIMT+G-CSF	V305-17_3	50	2	43,6
CIMT+G-CSF	V305-18_1	50	1	12
CIMT+G-CSF	V305-18_2	50	2	25,2
CIMT+G-CSF	V305-18_3	50	1	10,4
CIMT+G-CSF	V305-18_4	50	4	45,2
CIMT+G-CSF	V305-19_1	50	2	27,3
CIMT/G-CSF	V306-16_1	50	2	25,8
CIMT/G-CSF	V306-16_2	50	3	60,3
CIMT/G-CSF	V306-17_1	50	4	36,3
CIMT/G-CSF	V306-17_2	50	5	87,1
CIMT/G-CSF	V306-17_3	50	1	11,1
CIMT/G-CSF	V306-17_4	50	1	11,8
CIMT/G-CSF	V306-17_5	50	1	10,5
CIMT/G-CSF	V311-16_1	50	5	68,1
CIMT/G-CSF	V311-17_1	50	2	72,4
CIMT/G-CSF	V311-17_2	50	2	18,1
CIMT/G-CSF	V311-19_1	50	1	12,5
CIMT/G-CSF	V311-20_1	50	1	13,5
CIMT/G-CSF	V311-20_2	50	4	50,6
CIMT/G-CSF	V311-20_3	50	3	35,5
CIMT/G-CSF	V313-14_1	50	6	151,9
CIMT/G-CSF	V313-15_1	50	1	14,4
CIMT/G-CSF	V313-15_2	50	1	14,4
CIMT/G-CSF	V313-17_1	50	1	10,7
CIMT/G-CSF	V313-17_2	50	3	31,7
CIMT/G-CSF	V313-17_3	50	2	23,1
CIMT/G-CSF	V313-19_1	50	1	10,2
CIMT/G-CSF	V314-16_1	50	3	26,3
CIMT/G-CSF	V314-16_2	50	1	10,2
CIMT/G-CSF	V314-17_1	50	6	124,7
CIMT/G-CSF	V314-17_2	50	3	35,5
CIMT/G-CSF	V314-17_3	50	1	10,7
CIMT/G-CSF	V314-18_1	50	1	10,6
CIMT/G-CSF	V314-18_2	50	3	83,4
CIMT/G-CSF	V315-11_1	50	2	24,3
CIMT/G-CSF	V315-11_2	50	5	66,3
CIMT/G-CSF	V315-13_1	50	2	15,9
CIMT/G-CSF	V315-13_2	50	4	40,5
CIMT/G-CSF	V315-14_1	50	3	43,6
CIMT/G-CSF	V315-14_2	50	3	31,6
CIMT/G-CSF	V315-14_3	50	1	11,4
G-CSF	V220-9_1	50	4	43,2
G-CSF	V220-11_1	50	1	10,6
G-CSF	V220-12_1	50	2	22,2
G-CSF	V220-16_1	50	1	20,9
G-CSF	V220-16_2	50	7	98,2
G-CSF	V220-17_1	50	4	91,1
G-CSF	V220-18_1	50	2	12,9
G-CSF	V221-4_1	50	1	12,3
G-CSF	V221-5_1	50	5	56,9
G-CSF	V221-6_1	50	3	33,2
G-CSF	V221-7_1	50	3	43,8
G-CSF	V221-7_2	50	2	33

G-CSF	V221-11_1	50	2	28,4
G-CSF	V221-13_1	50	2	24,4
G-CSF	V221-15_1	50	2	41,9
G-CSF	V221-20_1	50	1	27,2
G-CSF	V222-19_1	50	5	53,3
G-CSF	V222-19_2	50	1	10,2
G-CSF	V222-20_1	50	3	40,6
G-CSF	V222-20_2	50	1	10,5
G-CSF	V222-21_1	50	4	58,7
G-CSF	V222-22_1	50	3	32,1
G-CSF	V222-22_2	50	1	10,7
G-CSF	V222-23_1	50	3	35,5
G-CSF	V222-24_1	50	2	37,4
G-CSF	V301-18_1	50	1	11,2
G-CSF	V301-18_2	50	1	13,9
G-CSF	V301-18_3	50	1	10
G-CSF	V301-19_1	50	6	55,4
G-CSF	V301-19_2	50	3	23,1
G-CSF	V301-20_1	50	6	64,6
G-CSF	V301-20_2	50	2	66,5
G-CSF	V302-15_1	50	1	10,1
G-CSF	V302-15_2	50	2	31,8
G-CSF	V302-15_3	50	3	76,9
G-CSF	V302-16_1	50	3	36
G-CSF	V302-16_2	50	1	11,7
G-CSF	V302-16_3	50	1	10,4
G-CSF	V302-16_4	50	3	46,3
G-CSF	V302-17_1	50	1	33,7
G-CSF	V302-17_2	50	3	52,5
G-CSF	V302-17_3	50	2	19
G-CSF	V303-15_1	50	1	11,2
G-CSF	V303-15_2	50	7	91,8
G-CSF	V303-15_3	50	1	11,4
G-CSF	V303-16_1	50	3	36,5
G-CSF	V303-16_2	50	3	36,4
G-CSF	V303-16_3	50	2	34,8
G-CSF	V303-17_1	50	1	12,2

apical dendrite Sholl 60µm

Group	number	Radius(µm)	Intersections	Length(µm)
Control	V215-10_1	60	1	22
Control	V215-11_1	60	1	10,9
Control	V215-12_1	60	1	10,2
Control	V215-12_2	60	1	13,6
Control	V215-13_1	60	3	41,8
Control	V215-15_1	60	2	33,3
Control	V215-15_2	60	2	21,4
Control	V215-16_1	60	5	62,9
Control	V215-18_1	60	2	42,7
Control	V216-11_1	60	4	64,5
Control	V216-12_1	60	2	26,7
Control	V216-13_1	60	1	13,4
Control	V216-14_1	60	2	24,1
Control	V216-14_2	60	1	13,1
Control	V216-16_1	60	1	10,8
Control	V216-16_2	60	2	30,9
Control	V216-17_1	60	6	99,4
Control	V216-18_1	60	1	10,6
Control	V216-18_2	60	1	11,6
Control	V219-6_1	60	1	10,9
Control	V219-11_1	60	2	30,3
Control	V219-12_1	60	1	11,3
Control	V219-13_1	60	3	60,6
Control	V219-13_2	60	1	15,5
Control	V219-14_1	60	2	28,7
Control	V219-14_2	60	4	66,9
Control	V219-16_1	60	2	34,9
Control	V297-19_1	60	1	24,6
Control	V297-19_2	60	2	32,6
Control	V297-20_1	60	1	27,8
Control	V297-20_2	60	3	31,7
Control	V297-20_3	60	1	10,3
Control	V297-22_1	60	7	101,5
Control	V297-22_2	60	2	22,4
Control	V298-15_1	60	5	75,3
Control	V298-16_1	60	3	47,5
Control	V298-16_2	60	3	43,3
Control	V298-16_3	60	2	59,9
Control	V298-17_1	60	3	33,3
Control	V298-17_2	60	2	20,6
Control	V298-17_3	60	1	10,2
Control	V308-17_1	60	2	30,3
Control	V308-17_2	60	2	26,8
Control	V308-17_3	60	3	52,9
Control	V308-17_4	60	3	33,9
Control	V308-18_1	60	3	46,4
Control	V308-18_2	60	6	126
Control	V308-18_3	60	6	78,4
CIMT	V211-10_1	60	4	55,6
CIMT	V211-10_2	60	3	59,6
CIMT	V211-11_1	60	2	15,2
CIMT	V211-11_2	60	1	15
CIMT	V211-12_1	60	1	10,1

CIMT	V211-13_1	60	3	57,6
CIMT	V211-13_2	60	3	51,6
CIMT	V211-15_1	60	2	20,3
CIMT	V211-15_2	60	1	20
CIMT	V211-16_1	60	4	13,2
CIMT	V212-14_1	60	2	55,9
CIMT	V212-14_2	60	8	115,5
CIMT	V212-15_1	60	11	168,2
CIMT	V212-18_1	60	3	31,8
CIMT	V212-21_1	60	2	16,3
CIMT	V212-21_2	60	3	43,8
CIMT	V212-22_1	60	1	13,9
CIMT	V212-23_1	60	3	37,6
CIMT	V212-23_2	60	2	25,3
CIMT	V212-24_1	60	5	89,5
CIMT	V218-9_1	60	1	10,1
CIMT	V218-10_1	60	4	54,7
CIMT	V218-11_1	60	1	18
CIMT	V218-12_1	60	5	76,6
CIMT	V218-12_2	60	4	46,9
CIMT	V218-13_1	60	1	11,1
CIMT	V218-15_1	60	9	144,7
CIMT	V218-17_1	60	3	44,1
CIMT	V299-14_1	60	4	86,6
CIMT	V299-15_1	60	1	10,8
CIMT	V299-15_2	60	1	10,5
CIMT	V299-16_1	60	2	43,9
CIMT	V299-16_2	60	1	11,1
CIMT	V299-17_1	60	7	84,2
CIMT	V299-17_2	60	3	39,6
CIMT	V300-16_1	60	3	41,8
CIMT	V300-18_1	60	2	23,2
CIMT	V300-18_2	60	1	10,2
CIMT	V300-20_1	60	1	10,1
CIMT	V300-22_1	60	1	10,1
CIMT	V300-23_1	60	5	82,5
CIMT	V300-23_2	60	4	72,9
CIMT	V309-15_1	60	3	50,3
CIMT	V309-15_2	60	3	32,2
CIMT	V309-16_1	60	2	23,4
CIMT	V309-16_2	60	2	27
CIMT	V309-16_3	60	4	90,5
CIMT	V309-17_1	60	4	77,3
CIMT	V309-17_2	60	3	27,5
CIMT+G-CSF	V213-13_1	60	2	22,2
CIMT+G-CSF	V213-16_1	60	3	36,4
CIMT+G-CSF	V213-17_1	60	2	20,9
CIMT+G-CSF	V213-17_2	60	6	61,7
CIMT+G-CSF	V213-18_1	60	6	84,3
CIMT+G-CSF	V213-18_2	60	3	43,8
CIMT+G-CSF	V213-19_1	60	2	24
CIMT+G-CSF	V213-19_2	60	1	10,4
CIMT+G-CSF	V213-20_1	60	3	45,8
CIMT+G-CSF	V213-22_1	60	2	26,5
CIMT+G-CSF	V213-23_1	60	1	12,1
CIMT+G-CSF	V214-14_1	60	1	12

CIMT+G-CSF	V214-17_1	60	2	65,2
CIMT+G-CSF	V214-18_1	60	2	15,1
CIMT+G-CSF	V214-19_1	60	3	62,5
CIMT+G-CSF	V214-19_2	60	2	21,6
CIMT+G-CSF	V214-20_1	60	6	63,3
CIMT+G-CSF	V214-21_1	60	1	10,6
CIMT+G-CSF	V217-1_1	60	1	14,1
CIMT+G-CSF	V217-4_1	60	1	12,8
CIMT+G-CSF	V217-6_1	60	4	99
CIMT+G-CSF	V217-6_3	60	1	24,9
CIMT+G-CSF	V217-8_1	60	2	48
CIMT+G-CSF	V217-8_4	60	3	41,9
CIMT+G-CSF	V217-10_1	60	1	12,3
CIMT+G-CSF	V217-11_1	60	2	22,6
CIMT+G-CSF	V304-17_1	60	2	35,9
CIMT+G-CSF	V304-17_2	60	1	11,7
CIMT+G-CSF	V304-17_3	60	4	49,1
CIMT+G-CSF	V304-17_4	60	1	11,6
CIMT+G-CSF	V304-19_1	60	1	11,1
CIMT+G-CSF	V304-19_2	60	2	42,7
CIMT+G-CSF	V305-17_1	60	2	24,9
CIMT+G-CSF	V305-17_2	60	2	23,5
CIMT+G-CSF	V305-17_3	60	3	28,6
CIMT+G-CSF	V305-18_1	60	2	28,2
CIMT+G-CSF	V305-18_2	60	3	52,4
CIMT+G-CSF	V305-18_3	60	1	11,9
CIMT+G-CSF	V305-18_4	60	4	50,2
CIMT+G-CSF	V305-19_1	60	1	11,2
CIMT/G-CSF	V306-16_1	60	3	27,3
CIMT/G-CSF	V306-16_2	60	3	33,7
CIMT/G-CSF	V306-17_1	60	6	96,8
CIMT/G-CSF	V306-17_2	60	5	77,1
CIMT/G-CSF	V306-17_3	60	1	10,9
CIMT/G-CSF	V306-17_4	60	1	10,7
CIMT/G-CSF	V306-17_5	60	2	17,3
CIMT/G-CSF	V311-16_1	60	5	83,1
CIMT/G-CSF	V311-17_1	60	3	40,9
CIMT/G-CSF	V311-17_2	60	2	36,7
CIMT/G-CSF	V311-19_1	60	2	22,8
CIMT/G-CSF	V311-20_1	60	5	90,1
CIMT/G-CSF	V311-20_2	60	8	51,7
CIMT/G-CSF	V311-20_3	60	2	23,7
CIMT/G-CSF	V313-14_1	60	6	94,7
CIMT/G-CSF	V313-15_1	60	1	12
CIMT/G-CSF	V313-15_2	60	1	12,1
CIMT/G-CSF	V313-17_1	60	1	12,3
CIMT/G-CSF	V313-17_2	60	2	37,2
CIMT/G-CSF	V313-17_3	60	2	22,9
CIMT/G-CSF	V313-19_1	60	1	10,3
CIMT/G-CSF	V314-16_1	60	3	36,1
CIMT/G-CSF	V314-16_2	60	3	39,5
CIMT/G-CSF	V314-17_1	60	5	87,6
CIMT/G-CSF	V314-17_2	60	4	49,3
CIMT/G-CSF	V314-17_3	60	1	11
CIMT/G-CSF	V314-18_1	60	5	56,5
CIMT/G-CSF	V314-18_2	60	3	37,9

CIMT/G-CSF	V315-11_1	60	2	25,2
CIMT/G-CSF	V315-11_2	60	3	60,6
CIMT/G-CSF	V315-13_1	60	2	43,8
CIMT/G-CSF	V315-13_2	60	4	58,6
CIMT/G-CSF	V315-14_1	60	1	16,5
CIMT/G-CSF	V315-14_2	60	3	39,6
CIMT/G-CSF	V315-14_3	60	1	13,4
G-CSF	V220-9_1	60	3	94,1
G-CSF	V220-11_1	60	1	10,4
G-CSF	V220-12_1	60	2	22,5
G-CSF	V220-16_1	60	1	12,3
G-CSF	V220-16_2	60	4	82,6
G-CSF	V220-17_1	60	4	49
G-CSF	V220-18_1	60	2	27,2
G-CSF	V221-4_1	60	0	2,3
G-CSF	V221-5_1	60	2	37,8
G-CSF	V221-6_1	60	7	122
G-CSF	V221-7_1	60	5	53,5
G-CSF	V221-7_2	60	3	36
G-CSF	V221-11_1	60	2	23,1
G-CSF	V221-13_1	60	3	28,8
G-CSF	V221-15_1	60	2	24
G-CSF	V221-20_1	60	1	10,3
G-CSF	V222-19_1	60	3	75,7
G-CSF	V222-19_2	60	1	11,7
G-CSF	V222-20_1	60	3	86,1
G-CSF	V222-20_2	60	1	10,6
G-CSF	V222-21_1	60	5	67,6
G-CSF	V222-22_1	60	3	31,3
G-CSF	V222-22_2	60	1	29,3
G-CSF	V222-23_1	60	3	71,7
G-CSF	V222-24_1	60	2	30,2
G-CSF	V301-18_1	60	1	10,6
G-CSF	V301-18_2	60	1	11,2
G-CSF	V301-18_3	60	1	11,6
G-CSF	V301-19_1	60	5	116,3
G-CSF	V301-19_2	60	3	52,1
G-CSF	V301-20_1	60	6	105,6
G-CSF	V301-20_2	60	4	84,8
G-CSF	V302-15_1	60	2	22,4
G-CSF	V302-15_2	60	4	68,4
G-CSF	V302-15_3	60	3	46,7
G-CSF	V302-16_1	60	3	33,1
G-CSF	V302-16_2	60	1	10,5
G-CSF	V302-16_3	60	1	11,2
G-CSF	V302-16_4	60	4	74
G-CSF	V302-17_1	60	1	13,5
G-CSF	V302-17_2	60	3	39
G-CSF	V302-17_3	60	3	35
G-CSF	V303-15_1	60	1	10,1
G-CSF	V303-15_2	60	8	116,2
G-CSF	V303-15_3	60	1	11,1
G-CSF	V303-16_1	60	4	49
G-CSF	V303-16_2	60	4	75,7
G-CSF	V303-16_3	60	3	32,9
G-CSF	V303-17_1	60	1	12,4

apical dendrite Sholl 70µm

Group	number	Radius(µm)	Intersections	Length(µm)
Control	V215-10_1	70	1	11,8
Control	V215-11_1	70	1	10,2
Control	V215-12_1	70	2	12,5
Control	V215-12_2	70	2	36,9
Control	V215-13_1	70	3	40,2
Control	V215-15_1	70	1	15,6
Control	V215-15_2	70	2	23,7
Control	V215-16_1	70	5	74,4
Control	V215-18_1	70	2	28,5
Control	V216-11_1	70	4	69,8
Control	V216-12_1	70	1	36,2
Control	V216-13_1	70	1	11,3
Control	V216-14_1	70	2	26,7
Control	V216-14_2	70	1	10,2
Control	V216-16_1	70	1	10,3
Control	V216-16_2	70	2	28,7
Control	V216-17_1	70	5	136,8
Control	V216-18_1	70	1	13,4
Control	V216-18_2	70	1	10,1
Control	V219-6_1	70	1	14,4
Control	V219-11_1	70	2	25,6
Control	V219-12_1	70	3	41
Control	V219-13_1	70	3	54,9
Control	V219-13_2	70	1	19,8
Control	V219-14_1	70	1	20,9
Control	V219-14_2	70	1	28,2
Control	V219-16_1	70	2	26,8
Control	V297-19_1	70	2	48,8
Control	V297-19_2	70	2	27
Control	V297-20_1	70	2	22,7
Control	V297-20_2	70	2	37,1
Control	V297-20_3	70	1	11,9
Control	V297-22_1	70	6	104,3
Control	V297-22_2	70	1	14,4
Control	V298-15_1	70	5	64,8
Control	V298-16_1	70	1	23,3
Control	V298-16_2	70	3	36
Control	V298-16_3	70	1	16,4
Control	V298-17_1	70	2	32,6
Control	V298-17_2	70	3	26
Control	V298-17_3	70	2	21,7
Control	V308-17_1	70	2	32,2
Control	V308-17_2	70	3	57,1
Control	V308-17_3	70	4	37,1
Control	V308-17_4	70	3	56,4
Control	V308-18_1	70	3	54,4
Control	V308-18_2	70	6	100,9
Control	V308-18_3	70	6	79,9
CIMT	V211-10_1	70	4	55,9
CIMT	V211-10_2	70	4	55,4
CIMT	V211-11_1	70	2	39,5
CIMT	V211-11_2	70	2	26,5

CIMT	V211-12_1	70	1	11,1
CIMT	V211-13_1	70	3	51,1
CIMT	V211-13_2	70	4	49
CIMT	V211-15_1	70	3	36,3
CIMT	V211-15_2	70	2	18,6
CIMT	V211-16_1	70	2	40
CIMT	V212-14_1	70	2	24,4
CIMT	V212-14_2	70	7	90,8
CIMT	V212-15_1	70	9	144,8
CIMT	V212-18_1	70	3	36,9
CIMT	V212-21_1	70	2	28,8
CIMT	V212-21_2	70	3	79,9
CIMT	V212-22_1	70	1	10,4
CIMT	V212-23_1	70	3	48
CIMT	V212-23_2	70	3	43,5
CIMT	V212-24_1	70	5	88,3
CIMT	V218-9_1	70	2	41,3
CIMT	V218-10_1	70	4	71,8
CIMT	V218-11_1	70	1	11,2
CIMT	V218-12_1	70	5	75
CIMT	V218-12_2	70	6	105,5
CIMT	V218-13_1	70	1	11,1
CIMT	V218-15_1	70	7	125,6
CIMT	V218-17_1	70	3	36,3
CIMT	V299-14_1	70	4	51,7
CIMT	V299-15_1	70	1	10,8
CIMT	V299-15_2	70	1	10,1
CIMT	V299-16_1	70	2	27,6
CIMT	V299-16_2	70	1	10,2
CIMT	V299-17_1	70	8	97,3
CIMT	V299-17_2	70	3	44,9
CIMT	V300-16_1	70	3	39,4
CIMT	V300-18_1	70	3	32
CIMT	V300-18_2	70	1	10,5
CIMT	V300-20_1	70	2	13
CIMT	V300-22_1	70	1	10,4
CIMT	V300-23_1	70	5	72,2
CIMT	V300-23_2	70	5	76,6
CIMT	V309-15_1	70	3	91,8
CIMT	V309-15_2	70	3	31,2
CIMT	V309-16_1	70	3	26,2
CIMT	V309-16_2	70	2	25,4
CIMT	V309-16_3	70	4	58
CIMT	V309-17_1	70	3	37
CIMT	V309-17_2	70	2	34,7
CIMT+G-CSF	V213-13_1	70	2	20,3
CIMT+G-CSF	V213-16_1	70	3	31,9
CIMT+G-CSF	V213-17_1	70	2	22,8
CIMT+G-CSF	V213-17_2	70	6	85,4
CIMT+G-CSF	V213-18_1	70	6	101,4
CIMT+G-CSF	V213-18_2	70	4	43,9
CIMT+G-CSF	V213-19_1	70	2	21,4
CIMT+G-CSF	V213-19_2	70	1	10,8
CIMT+G-CSF	V213-20_1	70	3	48,8
CIMT+G-CSF	V213-22_1	70	3	47,4
CIMT+G-CSF	V213-23_1	70	2	27,5

CIMT+G-CSF	V214-14_1	70	2	47,5
CIMT+G-CSF	V214-17_1	70	3	57,2
CIMT+G-CSF	V214-18_1	70	2	26,3
CIMT+G-CSF	V214-19_1	70	4	36,9
CIMT+G-CSF	V214-19_2	70	3	29,7
CIMT+G-CSF	V214-20_1	70	4	90,1
CIMT+G-CSF	V214-21_1	70	3	44,5
CIMT+G-CSF	V217-1_1	70	1	10,7
CIMT+G-CSF	V217-4_1	70	1	11,7
CIMT+G-CSF	V217-6_1	70	4	94
CIMT+G-CSF	V217-6_3	70	1	10,9
CIMT+G-CSF	V217-8_1	70	2	29,9
CIMT+G-CSF	V217-8_4	70	3	45,1
CIMT+G-CSF	V217-10_1	70	1	10,1
CIMT+G-CSF	V217-11_1	70	2	24,8
CIMT+G-CSF	V304-17_1	70	2	28,1
CIMT+G-CSF	V304-17_2	70	1	11,1
CIMT+G-CSF	V304-17_3	70	3	45,9
CIMT+G-CSF	V304-17_4	70	1	10,4
CIMT+G-CSF	V304-19_1	70	1	11,2
CIMT+G-CSF	V304-19_2	70	2	30,6
CIMT+G-CSF	V305-17_1	70	2	23,4
CIMT+G-CSF	V305-17_2	70	1	18,9
CIMT+G-CSF	V305-17_3	70	2	41,9
CIMT+G-CSF	V305-18_1	70	3	45,3
CIMT+G-CSF	V305-18_2	70	4	85,6
CIMT+G-CSF	V305-18_3	70	2	15,5
CIMT+G-CSF	V305-18_4	70	3	50,2
CIMT+G-CSF	V305-19_1	70	1	10,6
CIMT/G-CSF	V306-16_1	70	5	87,6
CIMT/G-CSF	V306-16_2	70	4	38,2
CIMT/G-CSF	V306-17_1	70	6	93,4
CIMT/G-CSF	V306-17_2	70	5	78,5
CIMT/G-CSF	V306-17_3	70	2	14
CIMT/G-CSF	V306-17_4	70	1	10,5
CIMT/G-CSF	V306-17_5	70	2	21
CIMT/G-CSF	V311-16_1	70	6	88,5
CIMT/G-CSF	V311-17_1	70	3	52,7
CIMT/G-CSF	V311-17_2	70	4	54,3
CIMT/G-CSF	V311-19_1	70	1	88,4
CIMT/G-CSF	V311-20_1	70	5	93,6
CIMT/G-CSF	V311-20_2	70	4	87
CIMT/G-CSF	V311-20_3	70	2	20,5
CIMT/G-CSF	V313-14_1	70	6	75
CIMT/G-CSF	V313-15_1	70	2	26
CIMT/G-CSF	V313-15_2	70	1	24,6
CIMT/G-CSF	V313-17_1	70	1	10,7
CIMT/G-CSF	V313-17_2	70	2	23,5
CIMT/G-CSF	V313-17_3	70	2	23,2
CIMT/G-CSF	V313-19_1	70	1	10,5
CIMT/G-CSF	V314-16_1	70	5	70,1
CIMT/G-CSF	V314-16_2	70	3	51,9
CIMT/G-CSF	V314-17_1	70	5	85,5
CIMT/G-CSF	V314-17_2	70	5	80,7
CIMT/G-CSF	V314-17_3	70	3	34,6
CIMT/G-CSF	V314-18_1	70	4	80,4

CIMT/G-CSF	V314-18_2	70	2	60,1
CIMT/G-CSF	V315-11_1	70	3	37,8
CIMT/G-CSF	V315-11_2	70	2	51,6
CIMT/G-CSF	V315-13_1	70	1	31,7
CIMT/G-CSF	V315-13_2	70	5	59,5
CIMT/G-CSF	V315-14_1	70	1	11,8
CIMT/G-CSF	V315-14_2	70	2	29,3
CIMT/G-CSF	V315-14_3	70	1	12,1
G-CSF	V220-9_1	70	4	69,2
G-CSF	V220-11_1	70	1	11,3
G-CSF	V220-12_1	70	2	20,8
G-CSF	V220-16_1	70	1	15,2
G-CSF	V220-16_2	70	3	58,3
G-CSF	V220-17_1	70	6	77,5
G-CSF	V220-18_1	70	2	23,7
G-CSF	V221-5_1	70	2	21,4
G-CSF	V221-6_1	70	7	153,3
G-CSF	V221-7_1	70	6	117,9
G-CSF	V221-7_2	70	4	39,6
G-CSF	V221-11_1	70	3	29,9
G-CSF	V221-13_1	70	3	35,2
G-CSF	V221-15_1	70	3	23,4
G-CSF	V221-20_1	70	1	10,7
G-CSF	V222-19_1	70	3	43,5
G-CSF	V222-19_2	70	1	10,4
G-CSF	V222-20_1	70	3	33,2
G-CSF	V222-20_2	70	1	13,7
G-CSF	V222-21_1	70	6	86,4
G-CSF	V222-22_1	70	5	66,1
G-CSF	V222-22_2	70	2	23,3
G-CSF	V222-23_1	70	3	42
G-CSF	V222-24_1	70	2	22,1
G-CSF	V301-18_1	70	1	10,5
G-CSF	V301-18_2	70	1	10,2
G-CSF	V301-18_3	70	1	10,8
G-CSF	V301-19_1	70	6	118
G-CSF	V301-19_2	70	4	56,8
G-CSF	V301-20_1	70	5	92
G-CSF	V301-20_2	70	5	106,3
G-CSF	V302-15_1	70	2	26,9
G-CSF	V302-15_2	70	4	57,6
G-CSF	V302-15_3	70	5	63,2
G-CSF	V302-16_1	70	3	37,9
G-CSF	V302-16_2	70	2	17,8
G-CSF	V302-16_3	70	1	11,7
G-CSF	V302-16_4	70	3	68,3
G-CSF	V302-17_1	70	1	11,6
G-CSF	V302-17_2	70	3	36,4
G-CSF	V302-17_3	70	4	64,3
G-CSF	V303-15_1	70	1	10,1
G-CSF	V303-15_2	70	7	126,1
G-CSF	V303-15_3	70	1	15,8
G-CSF	V303-16_1	70	4	60,6
G-CSF	V303-16_2	70	4	70,9
G-CSF	V303-16_3	70	3	33,3
G-CSF	V303-17_1	70	2	39,3

apical dendrite Sholl 80µm

Group	number	Radius(µm)	Intersections	Length(µm)
Control	V215-10_1	80	1	14,2
Control	V215-11_1	80	2	25,2
Control	V215-12_1	80	3	76,4
Control	V215-12_2	80	2	40
Control	V215-13_1	80	3	37,6
Control	V215-15_1	80	1	11,4
Control	V215-15_2	80	2	22,2
Control	V215-16_1	80	8	81,7
Control	V215-18_1	80	2	24,3
Control	V216-11_1	80	4	59,5
Control	V216-12_1	80	1	11,2
Control	V216-13_1	80	1	10,7
Control	V216-14_1	80	2	23,8
Control	V216-14_2	80	1	10,8
Control	V216-16_1	80	1	12,5
Control	V216-16_2	80	4	57,4
Control	V216-17_1	80	5	86,4
Control	V216-18_1	80	1	10,4
Control	V216-18_2	80	1	12,3
Control	V219-6_1	80	3	18,8
Control	V219-11_1	80	1	16,5
Control	V219-12_1	80	3	39,3
Control	V219-13_1	80	3	39,4
Control	V219-13_2	80	1	15,7
Control	V219-14_1	80	2	31,3
Control	V219-14_2	80	2	29
Control	V219-16_1	80	2	29,7
Control	V297-19_1	80	2	29
Control	V297-19_2	80	2	26,4
Control	V297-20_1	80	2	25,3
Control	V297-20_2	80	1	23,7
Control	V297-20_3	80	1	10,3
Control	V297-22_1	80	5	76,9
Control	V297-22_2	80	1	12,8
Control	V298-15_1	80	7	97,5
Control	V298-16_1	80	2	41,4
Control	V298-16_2	80	3	34,2
Control	V298-16_3	80	1	11,3
Control	V298-17_1	80	2	22,9
Control	V298-17_2	80	3	46,1
Control	V298-17_3	80	2	23,5
Control	V308-17_1	80	1	14,5
Control	V308-17_2	80	4	93,2
Control	V308-17_3	80	5	101,6
Control	V308-17_4	80	3	35,9
Control	V308-18_1	80	2	62,5
Control	V308-18_2	80	6	76,5
Control	V308-18_3	80	6	70,1
CIMT	V211-10_1	80	4	43,7
CIMT	V211-10_2	80	4	85,6
CIMT	V211-11_1	80	2	33,1
CIMT	V211-11_2	80	2	25,8

CIMT	V211-12_1	80	1	11,1
CIMT	V211-13_1	80	3	36,2
CIMT	V211-13_2	80	4	52,7
CIMT	V211-15_1	80	3	35,7
CIMT	V211-15_2	80	1	48,5
CIMT	V211-16_1	80	2	24,8
CIMT	V212-14_1	80	2	32,9
CIMT	V212-14_2	80	7	93,7
CIMT	V212-15_1	80	7	126
CIMT	V212-18_1	80	3	46,6
CIMT	V212-21_1	80	2	22,4
CIMT	V212-21_2	80	2	60,4
CIMT	V212-22_1	80	1	12,3
CIMT	V212-23_1	80	3	40,4
CIMT	V212-23_2	80	3	38,5
CIMT	V212-24_1	80	5	57,7
CIMT	V218-9_1	80	2	23,2
CIMT	V218-10_1	80	2	34,9
CIMT	V218-11_1	80	4	24,9
CIMT	V218-12_1	80	5	93,3
CIMT	V218-12_2	80	4	61,8
CIMT	V218-13_1	80	1	11
CIMT	V218-15_1	80	4	57,2
CIMT	V218-17_1	80	3	35,4
CIMT	V299-14_1	80	5	60,5
CIMT	V299-15_1	80	1	10,6
CIMT	V299-15_2	80	1	10,5
CIMT	V299-16_1	80	2	27
CIMT	V299-16_2	80	1	10,1
CIMT	V299-17_1	80	9	96,3
CIMT	V299-17_2	80	3	32,1
CIMT	V300-16_1	80	3	40,9
CIMT	V300-18_1	80	2	37,9
CIMT	V300-18_2	80	2	14,7
CIMT	V300-20_1	80	2	40,6
CIMT	V300-22_1	80	1	10,2
CIMT	V300-23_1	80	5	58,8
CIMT	V300-23_2	80	4	54,7
CIMT	V309-15_1	80	2	22,8
CIMT	V309-15_2	80	4	63,2
CIMT	V309-16_1	80	3	39
CIMT	V309-16_2	80	1	20,6
CIMT	V309-16_3	80	5	73,5
CIMT	V309-17_1	80	8	64,9
CIMT	V309-17_2	80	4	31
CIMT+G-CSF	V213-13_1	80	3	35,5
CIMT+G-CSF	V213-16_1	80	1	25,5
CIMT+G-CSF	V213-17_1	80	2	25,7
CIMT+G-CSF	V213-17_2	80	4	64
CIMT+G-CSF	V213-18_1	80	4	55,3
CIMT+G-CSF	V213-18_2	80	3	87,3
CIMT+G-CSF	V213-19_1	80	2	21,2
CIMT+G-CSF	V213-19_2	80	2	14,8
CIMT+G-CSF	V213-20_1	80	2	21,6
CIMT+G-CSF	V213-22_1	80	3	49,8
CIMT+G-CSF	V213-23_1	80	3	33,5

CIMT+G-CSF	V214-14_1	80	2	43,2
CIMT+G-CSF	V214-17_1	80	4	69,7
CIMT+G-CSF	V214-18_1	80	2	27,3
CIMT+G-CSF	V214-19_1	80	3	74,4
CIMT+G-CSF	V214-19_2	80	3	42,2
CIMT+G-CSF	V214-20_1	80	2	37,8
CIMT+G-CSF	V214-21_1	80	2	49,6
CIMT+G-CSF	V217-1_1	80	1	11,3
CIMT+G-CSF	V217-4_1	80	1	12,8
CIMT+G-CSF	V217-6_1	80	3	53,1
CIMT+G-CSF	V217-6_3	80	1	10,6
CIMT+G-CSF	V217-8_1	80	2	29,7
CIMT+G-CSF	V217-8_4	80	3	39,6
CIMT+G-CSF	V217-10_1	80	1	10,7
CIMT+G-CSF	V217-11_1	80	1	15,7
CIMT+G-CSF	V304-17_1	80	2	24,9
CIMT+G-CSF	V304-17_2	80	1	10,2
CIMT+G-CSF	V304-17_3	80	3	42,8
CIMT+G-CSF	V304-17_4	80	1	10,7
CIMT+G-CSF	V304-19_1	80	1	12,8
CIMT+G-CSF	V304-19_2	80	1	20,2
CIMT+G-CSF	V305-17_1	80	2	27,6
CIMT+G-CSF	V305-17_2	80	1	10,5
CIMT+G-CSF	V305-17_3	80	2	29
CIMT+G-CSF	V305-18_1	80	2	53,8
CIMT+G-CSF	V305-18_2	80	3	53,9
CIMT+G-CSF	V305-18_3	80	2	25,7
CIMT+G-CSF	V305-18_4	80	3	44,2
CIMT+G-CSF	V305-19_1	80	1	11,7
CIMT/G-CSF	V306-16_1	80	5	81,7
CIMT/G-CSF	V306-16_2	80	4	53,6
CIMT/G-CSF	V306-17_1	80	8	120,7
CIMT/G-CSF	V306-17_2	80	3	91
CIMT/G-CSF	V306-17_3	80	5	103,9
CIMT/G-CSF	V306-17_4	80	1	10,8
CIMT/G-CSF	V306-17_5	80	2	21,2
CIMT/G-CSF	V311-16_1	80	6	85,9
CIMT/G-CSF	V311-17_1	80	3	46
CIMT/G-CSF	V311-17_2	80	3	78,4
CIMT/G-CSF	V311-19_1	80	1	10,8
CIMT/G-CSF	V311-20_1	80	4	63,6
CIMT/G-CSF	V311-20_2	80	3	59
CIMT/G-CSF	V311-20_3	80	2	21,1
CIMT/G-CSF	V313-14_1	80	6	70
CIMT/G-CSF	V313-15_1	80	4	56
CIMT/G-CSF	V313-15_2	80	1	12
CIMT/G-CSF	V313-17_1	80	1	11,1
CIMT/G-CSF	V313-17_2	80	2	23,5
CIMT/G-CSF	V313-17_3	80	2	22,4
CIMT/G-CSF	V313-19_1	80	4	51,9
CIMT/G-CSF	V314-16_1	80	4	65,4
CIMT/G-CSF	V314-16_2	80	3	61,6
CIMT/G-CSF	V314-17_1	80	2	56,8
CIMT/G-CSF	V314-17_2	80	5	59
CIMT/G-CSF	V314-17_3	80	2	120,5
CIMT/G-CSF	V314-18_1	80	3	58,1

CIMT/G-CSF	V314-18_2	80	2	60
CIMT/G-CSF	V315-11_1	80	2	36,5
CIMT/G-CSF	V315-11_2	80	2	32,5
CIMT/G-CSF	V315-13_1	80	1	10,1
CIMT/G-CSF	V315-13_2	80	5	59,4
CIMT/G-CSF	V315-14_1	80	1	12,2
CIMT/G-CSF	V315-14_2	80	2	22,1
CIMT/G-CSF	V315-14_3	80	2	18,8
G-CSF	V220-9_1	80	4	102,6
G-CSF	V220-11_1	80	1	10,3
G-CSF	V220-12_1	80	3	49
G-CSF	V220-16_1	80	1	18,5
G-CSF	V220-16_2	80	3	70,3
G-CSF	V220-17_1	80	5	95,5
G-CSF	V220-18_1	80	3	39,9
G-CSF	V221-5_1	80	2	23,2
G-CSF	V221-6_1	80	6	101
G-CSF	V221-7_1	80	5	74,1
G-CSF	V221-7_2	80	6	85,5
G-CSF	V221-11_1	80	3	40,3
G-CSF	V221-13_1	80	5	60,2
G-CSF	V221-15_1	80	3	35,2
G-CSF	V221-20_1	80	2	15
G-CSF	V222-19_1	80	2	23,7
G-CSF	V222-19_2	80	1	11,2
G-CSF	V222-20_1	80	5	47
G-CSF	V222-20_2	80	1	13,4
G-CSF	V222-21_1	80	3	62,8
G-CSF	V222-22_1	80	3	55,4
G-CSF	V222-22_2	80	2	27,2
G-CSF	V222-23_1	80	2	37,1
G-CSF	V222-24_1	80	3	26,8
G-CSF	V301-18_1	80	1	10,4
G-CSF	V301-18_2	80	1	10,5
G-CSF	V301-18_3	80	1	10,3
G-CSF	V301-19_1	80	5	73,2
G-CSF	V301-19_2	80	4	68,4
G-CSF	V301-20_1	80	4	54
G-CSF	V301-20_2	80	4	68
G-CSF	V302-15_1	80	2	37,4
G-CSF	V302-15_2	80	2	54,3
G-CSF	V302-15_3	80	4	61,7
G-CSF	V302-16_1	80	3	34,8
G-CSF	V302-16_2	80	2	25,7
G-CSF	V302-16_3	80	1	13,6
G-CSF	V302-16_4	80	3	44,2
G-CSF	V302-17_1	80	1	13,7
G-CSF	V302-17_2	80	3	33,8
G-CSF	V302-17_3	80	4	52,1
G-CSF	V303-15_1	80	1	11
G-CSF	V303-15_2	80	6	92,7
G-CSF	V303-15_3	80	1	10,4
G-CSF	V303-16_1	80	4	55,7
G-CSF	V303-16_2	80	4	82,6
G-CSF	V303-16_3	80	3	40,6
G-CSF	V303-17_1	80	5	59,1

apical dendrite Sholl 90µm

Group	number	Radius(µm)	Intersections	Length(µm)
Control	V215-10_1	90	1	14,1
Control	V215-11_1	90	2	27,2
Control	V215-12_1	90	2	90,3
Control	V215-12_2	90	2	22,6
Control	V215-13_1	90	2	56,7
Control	V215-15_1	90	1	10,4
Control	V215-15_2	90	3	32
Control	V215-16_1	90	3	42,4
Control	V215-18_1	90	2	22,1
Control	V216-11_1	90	3	38,8
Control	V216-12_1	90	1	10,8
Control	V216-13_1	90	1	11,1
Control	V216-14_1	90	2	21
Control	V216-14_2	90	1	11,5
Control	V216-16_1	90	1	11,1
Control	V216-16_2	90	3	45,6
Control	V216-17_1	90	4	61,1
Control	V216-18_1	90	1	10,6
Control	V216-18_2	90	2	16,1
Control	V219-6_1	90	4	84,6
Control	V219-11_1	90	1	10,6
Control	V219-12_1	90	3	37,9
Control	V219-13_1	90	5	57,2
Control	V219-13_2	90	1	17,6
Control	V219-14_1	90	2	36
Control	V219-14_2	90	2	30,3
Control	V219-16_1	90	2	23,1
Control	V297-19_1	90	2	29
Control	V297-19_2	90	1	19,4
Control	V297-20_1	90	2	22,6
Control	V297-20_2	90	1	11,2
Control	V297-20_3	90	2	35,2
Control	V297-22_1	90	5	59,1
Control	V297-22_2	90	1	13,1
Control	V298-15_1	90	7	92,2
Control	V298-16_1	90	5	53,4
Control	V298-16_2	90	2	28,6
Control	V298-16_3	90	1	10,6
Control	V298-17_1	90	2	33,9
Control	V298-17_2	90	3	34,5
Control	V298-17_3	90	2	25,4
Control	V308-17_1	90	1	10,9
Control	V308-17_2	90	4	76,6
Control	V308-17_3	90	4	84,8
Control	V308-17_4	90	2	33,6
Control	V308-18_1	90	2	32,2
Control	V308-18_2	90	4	74,8
Control	V308-18_3	90	8	132,2
CIMT	V211-10_1	90	6	52,5
CIMT	V211-10_2	90	3	61
CIMT	V211-11_1	90	2	23,7
CIMT	V211-11_2	90	2	24,2

CIMT	V211-12_1	90	1	13,6
CIMT	V211-13_1	90	3	35,3
CIMT	V211-13_2	90	5	60,6
CIMT	V211-15_1	90	2	49,4
CIMT	V211-15_2	90	1	12
CIMT	V211-16_1	90	3	34,6
CIMT	V212-14_1	90	3	30,2
CIMT	V212-14_2	90	8	92,4
CIMT	V212-15_1	90	8	133
CIMT	V212-18_1	90	3	34,3
CIMT	V212-21_1	90	2	20,6
CIMT	V212-21_2	90	5	142,6
CIMT	V212-22_1	90	2	15,8
CIMT	V212-23_1	90	3	38,9
CIMT	V212-23_2	90	3	35,1
CIMT	V212-24_1	90	5	65,5
CIMT	V218-9_1	90	2	22,6
CIMT	V218-10_1	90	2	32,4
CIMT	V218-11_1	90	1	36,4
CIMT	V218-12_1	90	4	72,4
CIMT	V218-12_2	90	3	55,1
CIMT	V218-13_1	90	1	10,8
CIMT	V218-15_1	90	4	55
CIMT	V218-17_1	90	5	41,1
CIMT	V299-14_1	90	4	49,8
CIMT	V299-15_1	90	1	10,7
CIMT	V299-15_2	90	1	10,9
CIMT	V299-16_1	90	1	16,5
CIMT	V299-16_2	90	1	10,2
CIMT	V299-17_1	90	9	107,2
CIMT	V299-17_2	90	3	32,7
CIMT	V300-16_1	90	3	34,1
CIMT	V300-18_1	90	1	12,2
CIMT	V300-18_2	90	2	25,6
CIMT	V300-20_1	90	2	29,7
CIMT	V300-22_1	90	1	10,3
CIMT	V300-23_1	90	3	52,4
CIMT	V300-23_2	90	3	35,9
CIMT	V309-15_1	90	1	13,6
CIMT	V309-15_2	90	5	82,6
CIMT	V309-16_1	90	3	54,8
CIMT	V309-16_2	90	2	30,3
CIMT	V309-16_3	90	6	92,9
CIMT	V309-17_1	90	4	76,1
CIMT	V309-17_2	90	3	72,3
CIMT+G-CSF	V213-13_1	90	3	40,3
CIMT+G-CSF	V213-16_1	90	1	12,1
CIMT+G-CSF	V213-17_1	90	3	35,4
CIMT+G-CSF	V213-17_2	90	10	69,6
CIMT+G-CSF	V213-18_1	90	4	94,7
CIMT+G-CSF	V213-18_2	90	2	30,7
CIMT+G-CSF	V213-19_1	90	2	32,9
CIMT+G-CSF	V213-19_2	90	2	66,4
CIMT+G-CSF	V213-20_1	90	2	22,2
CIMT+G-CSF	V213-22_1	90	2	32,1
CIMT+G-CSF	V213-23_1	90	1	28,7

CIMT+G-CSF	V214-14_1	90	2	40,7
CIMT+G-CSF	V214-17_1	90	3	48,8
CIMT+G-CSF	V214-18_1	90	3	28,4
CIMT+G-CSF	V214-19_1	90	3	37,5
CIMT+G-CSF	V214-19_2	90	4	37,6
CIMT+G-CSF	V214-20_1	90	4	28,2
CIMT+G-CSF	V214-21_1	90	2	87,1
CIMT+G-CSF	V217-1_1	90	1	12,5
CIMT+G-CSF	V217-4_1	90	1	16,4
CIMT+G-CSF	V217-6_1	90	3	56,2
CIMT+G-CSF	V217-6_3	90	3	76
CIMT+G-CSF	V217-8_1	90	2	24,7
CIMT+G-CSF	V217-8_4	90	3	49,3
CIMT+G-CSF	V217-10_1	90	1	13,3
CIMT+G-CSF	V217-11_1	90	1	15,4
CIMT+G-CSF	V304-17_1	90	2	29,9
CIMT+G-CSF	V304-17_2	90	1	10,6
CIMT+G-CSF	V304-17_3	90	3	41,8
CIMT+G-CSF	V304-17_4	90	1	29,3
CIMT+G-CSF	V304-19_1	90	2	20,4
CIMT+G-CSF	V304-19_2	90	1	11,9
CIMT+G-CSF	V305-17_1	90	1	17,6
CIMT+G-CSF	V305-17_2	90	4	33,1
CIMT+G-CSF	V305-17_3	90	1	13,5
CIMT+G-CSF	V305-18_1	90	1	15,3
CIMT+G-CSF	V305-18_2	90	3	41,7
CIMT+G-CSF	V305-18_3	90	2	27,9
CIMT+G-CSF	V305-18_4	90	2	32,1
CIMT+G-CSF	V305-19_1	90	1	10,2
CIMT/G-CSF	V306-16_1	90	4	66,9
CIMT/G-CSF	V306-16_2	90	2	29
CIMT/G-CSF	V306-17_1	90	8	150,3
CIMT/G-CSF	V306-17_2	90	2	26,2
CIMT/G-CSF	V306-17_3	90	5	63,3
CIMT/G-CSF	V306-17_4	90	1	10,4
CIMT/G-CSF	V306-17_5	90	2	22,3
CIMT/G-CSF	V311-16_1	90	7	93,3
CIMT/G-CSF	V311-17_1	90	5	107,3
CIMT/G-CSF	V311-17_2	90	3	44
CIMT/G-CSF	V311-19_1	90	2	59,3
CIMT/G-CSF	V311-20_1	90	4	46,3
CIMT/G-CSF	V311-20_2	90	2	49,3
CIMT/G-CSF	V311-20_3	90	2	23
CIMT/G-CSF	V313-14_1	90	5	64,2
CIMT/G-CSF	V313-15_1	90	2	40,8
CIMT/G-CSF	V313-15_2	90	1	10,6
CIMT/G-CSF	V313-17_1	90	1	11,7
CIMT/G-CSF	V313-17_2	90	2	21,9
CIMT/G-CSF	V313-17_3	90	3	35,8
CIMT/G-CSF	V313-19_1	90	2	28,9
CIMT/G-CSF	V314-16_1	90	4	53,7
CIMT/G-CSF	V314-16_2	90	4	57,3
CIMT/G-CSF	V314-17_1	90	2	24,3
CIMT/G-CSF	V314-17_2	90	4	50,7
CIMT/G-CSF	V314-17_3	90	2	23,6
CIMT/G-CSF	V314-18_1	90	3	39,8

CIMT/G-CSF	V314-18_2	90	2	24,2
CIMT/G-CSF	V315-11_1	90	2	25,2
CIMT/G-CSF	V315-11_2	90	1	14,6
CIMT/G-CSF	V315-13_1	90	1	10,1
CIMT/G-CSF	V315-13_2	90	7	107,4
CIMT/G-CSF	V315-14_1	90	1	10,3
CIMT/G-CSF	V315-14_2	90	3	29
CIMT/G-CSF	V315-14_3	90	2	25
G-CSF	V220-9_1	90	3	45,4
G-CSF	V220-11_1	90	1	13,2
G-CSF	V220-12_1	90	3	39,3
G-CSF	V220-16_1	90	0	0,3
G-CSF	V220-16_2	90	2	25,6
G-CSF	V220-17_1	90	6	71,3
G-CSF	V220-18_1	90	3	68,9
G-CSF	V221-5_1	90	2	21,7
G-CSF	V221-6_1	90	7	117,8
G-CSF	V221-7_1	90	4	45,1
G-CSF	V221-7_2	90	7	102,8
G-CSF	V221-11_1	90	3	38,1
G-CSF	V221-13_1	90	8	83,5
G-CSF	V221-15_1	90	2	29,9
G-CSF	V221-20_1	90	2	27,9
G-CSF	V222-19_1	90	2	23,2
G-CSF	V222-19_2	90	1	13
G-CSF	V222-20_1	90	3	67,5
G-CSF	V222-20_2	90	1	12,1
G-CSF	V222-21_1	90	3	33,8
G-CSF	V222-22_1	90	5	113,8
G-CSF	V222-22_2	90	1	10,5
G-CSF	V222-23_1	90	3	43,5
G-CSF	V222-24_1	90	3	48,9
G-CSF	V301-18_1	90	1	12,3
G-CSF	V301-18_2	90	2	17,5
G-CSF	V301-18_3	90	1	11,1
G-CSF	V301-19_1	90	4	56,8
G-CSF	V301-19_2	90	3	36,9
G-CSF	V301-20_1	90	4	48,2
G-CSF	V301-20_2	90	4	45
G-CSF	V302-15_1	90	3	40
G-CSF	V302-15_2	90	1	30,9
G-CSF	V302-15_3	90	4	46,7
G-CSF	V302-16_1	90	4	58
G-CSF	V302-16_2	90	1	23,4
G-CSF	V302-16_3	90	1	11,1
G-CSF	V302-16_4	90	3	38,7
G-CSF	V302-17_1	90	1	11,3
G-CSF	V302-17_2	90	4	80,4
G-CSF	V302-17_3	90	3	45,8
G-CSF	V303-15_1	90	1	10,7
G-CSF	V303-15_2	90	6	106,4
G-CSF	V303-15_3	90	1	10,5
G-CSF	V303-16_1	90	3	42,8
G-CSF	V303-16_2	90	3	47,7
G-CSF	V303-16_3	90	3	39,5
G-CSF	V303-17_1	90	5	68,3

apical dendrite Sholl 100µm

Group	number	Radius(µm)	Intersections	Length(µm)
Control	V215-10_1	100	0	9,6
Control	V215-11_1	100	2	25,7
Control	V215-12_1	100	2	32,1
Control	V215-12_2	100	2	22,6
Control	V215-13_1	100	2	29,5
Control	V215-15_1	100	2	18,7
Control	V215-15_2	100	4	55,6
Control	V215-16_1	100	2	33,3
Control	V215-18_1	100	2	25,3
Control	V216-11_1	100	3	45,5
Control	V216-12_1	100	2	11,1
Control	V216-13_1	100	1	11
Control	V216-14_1	100	3	34,5
Control	V216-14_2	100	2	30,8
Control	V216-16_1	100	1	11,1
Control	V216-16_2	100	2	26,2
Control	V216-17_1	100	3	56,8
Control	V216-18_1	100	1	11,4
Control	V216-18_2	100	2	27,2
Control	V219-6_1	100	2	59,3
Control	V219-11_1	100	1	13,7
Control	V219-12_1	100	3	37,6
Control	V219-13_1	100	3	61
Control	V219-13_2	100	1	12,3
Control	V219-14_1	100	2	33,5
Control	V219-14_2	100	3	27,6
Control	V219-16_1	100	1	14,5
Control	V297-19_1	100	3	38,5
Control	V297-19_2	100	1	16,5
Control	V297-20_1	100	2	30,8
Control	V297-20_2	100	1	10,9
Control	V297-20_3	100	2	51,2
Control	V297-22_1	100	5	79,6
Control	V297-22_2	100	1	11,5
Control	V298-15_1	100	6	95,4
Control	V298-16_1	100	3	68,1
Control	V298-16_2	100	3	31,2
Control	V298-16_3	100	1	10,2
Control	V298-17_1	100	1	15,5
Control	V298-17_2	100	3	33,5
Control	V298-17_3	100	2	29,2
Control	V308-17_1	100	1	10,4
Control	V308-17_2	100	2	47
Control	V308-17_3	100	2	43,4
Control	V308-17_4	100	1	18,1
Control	V308-18_1	100	1	21,3
Control	V308-18_2	100	4	49,9
Control	V308-18_3	100	10	159,7
CIMT	V211-10_1	100	6	101,7
CIMT	V211-10_2	100	3	56,4
CIMT	V211-11_1	100	6	31,3
CIMT	V211-11_2	100	2	27,4

CIMT	V211-12_1	100	2	23,2
CIMT	V211-13_1	100	3	35,5
CIMT	V211-13_2	100	6	65,1
CIMT	V211-15_1	100	1	26,5
CIMT	V211-15_2	100	1	11,2
CIMT	V211-16_1	100	4	60,7
CIMT	V212-14_1	100	3	48,3
CIMT	V212-14_2	100	9	114
CIMT	V212-15_1	100	7	100,4
CIMT	V212-18_1	100	4	60,4
CIMT	V212-21_1	100	2	23,9
CIMT	V212-21_2	100	5	106,8
CIMT	V212-22_1	100	3	74,9
CIMT	V212-23_1	100	3	42,3
CIMT	V212-23_2	100	3	35,2
CIMT	V212-24_1	100	4	61,2
CIMT	V218-9_1	100	2	24,1
CIMT	V218-10_1	100	1	32,6
CIMT	V218-11_1	100	1	10,3
CIMT	V218-12_1	100	3	49,5
CIMT	V218-12_2	100	2	48
CIMT	V218-13_1	100	0	6,1
CIMT	V218-15_1	100	2	31,2
CIMT	V218-17_1	100	5	84,5
CIMT	V299-14_1	100	3	42,3
CIMT	V299-15_1	100	1	20,3
CIMT	V299-15_2	100	1	15,9
CIMT	V299-16_1	100	1	10,1
CIMT	V299-16_2	100	1	10,1
CIMT	V299-17_1	100	9	126,3
CIMT	V299-17_2	100	2	20,6
CIMT	V300-16_1	100	2	24,6
CIMT	V300-18_1	100	4	36,4
CIMT	V300-18_2	100	2	25,3
CIMT	V300-20_1	100	2	35,1
CIMT	V300-22_1	100	1	10,5
CIMT	V300-23_1	100	2	22,5
CIMT	V300-23_2	100	4	49,1
CIMT	V309-15_1	100	1	11,7
CIMT	V309-15_2	100	5	56,6
CIMT	V309-16_1	100	4	76,7
CIMT	V309-16_2	100	2	36
CIMT	V309-16_3	100	6	96,8
CIMT	V309-17_1	100	3	49,6
CIMT	V309-17_2	100	3	46
CIMT+G-CSF	V213-13_1	100	3	37,3
CIMT+G-CSF	V213-16_1	100	1	12,4
CIMT+G-CSF	V213-17_1	100	1	34,2
CIMT+G-CSF	V213-17_2	100	3	132,3
CIMT+G-CSF	V213-18_1	100	4	83,2
CIMT+G-CSF	V213-18_2	100	2	22
CIMT+G-CSF	V213-19_1	100	1	19,5
CIMT+G-CSF	V213-19_2	100	2	28,2
CIMT+G-CSF	V213-20_1	100	1	19,3
CIMT+G-CSF	V213-22_1	100	2	29,2
CIMT+G-CSF	V213-23_1	100	1	10,2

CIMT+G-CSF	V214-14_1	100	2	32
CIMT+G-CSF	V214-17_1	100	3	38,3
CIMT+G-CSF	V214-18_1	100	2	36,1
CIMT+G-CSF	V214-19_1	100	2	37,6
CIMT+G-CSF	V214-19_2	100	4	58,8
CIMT+G-CSF	V214-20_1	100	3	79,9
CIMT+G-CSF	V214-21_1	100	2	27,2
CIMT+G-CSF	V217-1_1	100	1	10,3
CIMT+G-CSF	V217-4_1	100	0	0,9
CIMT+G-CSF	V217-6_1	100	3	41,9
CIMT+G-CSF	V217-6_3	100	3	46,6
CIMT+G-CSF	V217-8_1	100	2	26,7
CIMT+G-CSF	V217-8_4	100	2	50,6
CIMT+G-CSF	V217-10_1	100	1	14,1
CIMT+G-CSF	V217-11_1	100	1	15,5
CIMT+G-CSF	V304-17_1	100	1	16,9
CIMT+G-CSF	V304-17_2	100	1	12,1
CIMT+G-CSF	V304-17_3	100	3	32
CIMT+G-CSF	V304-17_4	100	1	12
CIMT+G-CSF	V304-19_1	100	2	23,4
CIMT+G-CSF	V304-19_2	100	1	11,1
CIMT+G-CSF	V305-17_1	100	1	10,6
CIMT+G-CSF	V305-17_2	100	1	52,8
CIMT+G-CSF	V305-17_3	100	1	10,7
CIMT+G-CSF	V305-18_1	100	1	11,2
CIMT+G-CSF	V305-18_2	100	2	56,2
CIMT+G-CSF	V305-18_3	100	2	40,2
CIMT+G-CSF	V305-18_4	100	1	19,9
CIMT+G-CSF	V305-19_1	100	1	13,2
CIMT/G-CSF	V306-16_1	100	4	47,6
CIMT/G-CSF	V306-16_2	100	2	23,2
CIMT/G-CSF	V306-17_1	100	7	95,6
CIMT/G-CSF	V306-17_2	100	1	11,3
CIMT/G-CSF	V306-17_3	100	3	46,4
CIMT/G-CSF	V306-17_4	100	3	31,9
CIMT/G-CSF	V306-17_5	100	2	20,8
CIMT/G-CSF	V311-16_1	100	7	94,4
CIMT/G-CSF	V311-17_1	100	4	75,1
CIMT/G-CSF	V311-17_2	100	1	30,9
CIMT/G-CSF	V311-19_1	100	1	20
CIMT/G-CSF	V311-20_1	100	4	57,4
CIMT/G-CSF	V311-20_2	100	1	21,5
CIMT/G-CSF	V311-20_3	100	2	20,3
CIMT/G-CSF	V313-14_1	100	5	84
CIMT/G-CSF	V313-15_1	100	1	46,9
CIMT/G-CSF	V313-15_2	100	1	10,4
CIMT/G-CSF	V313-17_1	100	1	10
CIMT/G-CSF	V313-17_2	100	2	21,9
CIMT/G-CSF	V313-17_3	100	3	34,7
CIMT/G-CSF	V313-19_1	100	2	44,6
CIMT/G-CSF	V314-16_1	100	6	67,5
CIMT/G-CSF	V314-16_2	100	4	86,8
CIMT/G-CSF	V314-17_1	100	1	22,2
CIMT/G-CSF	V314-17_2	100	6	85,1
CIMT/G-CSF	V314-17_3	100	3	46,6
CIMT/G-CSF	V314-18_1	100	2	33,2

CIMT/G-CSF	V314-18_2	100	2	25,7
CIMT/G-CSF	V315-11_1	100	2	24,2
CIMT/G-CSF	V315-11_2	100	1	10,1
CIMT/G-CSF	V315-13_1	100	2	22,2
CIMT/G-CSF	V315-13_2	100	7	125,4
CIMT/G-CSF	V315-14_1	100	1	10,4
CIMT/G-CSF	V315-14_2	100	3	31,2
CIMT/G-CSF	V315-14_3	100	3	29,1
G-CSF	V220-9_1	100	2	45,5
G-CSF	V220-11_1	100	1	10,2
G-CSF	V220-12_1	100	3	37
G-CSF	V220-16_2	100	2	24
G-CSF	V220-17_1	100	4	71,3
G-CSF	V220-18_1	100	3	53,2
G-CSF	V221-5_1	100	1	14,6
G-CSF	V221-6_1	100	6	108,4
G-CSF	V221-7_1	100	4	41,9
G-CSF	V221-7_2	100	8	91,2
G-CSF	V221-11_1	100	3	36,1
G-CSF	V221-13_1	100	6	100,4
G-CSF	V221-15_1	100	2	22,6
G-CSF	V221-20_1	100	1	43,6
G-CSF	V222-19_1	100	2	22,1
G-CSF	V222-19_2	100	2	35,3
G-CSF	V222-20_1	100	4	49,7
G-CSF	V222-20_2	100	1	11,1
G-CSF	V222-21_1	100	2	24,1
G-CSF	V222-22_1	100	6	109,1
G-CSF	V222-22_2	100	1	12,8
G-CSF	V222-23_1	100	4	77,8
G-CSF	V222-24_1	100	2	32,8
G-CSF	V301-18_1	100	2	31,3
G-CSF	V301-18_2	100	2	29,8
G-CSF	V301-18_3	100	1	17,3
G-CSF	V301-19_1	100	4	49,5
G-CSF	V301-19_2	100	2	29,5
G-CSF	V301-20_1	100	4	51,2
G-CSF	V301-20_2	100	4	47,1
G-CSF	V302-15_1	100	3	51,1
G-CSF	V302-15_2	100	1	13,6
G-CSF	V302-15_3	100	5	56,7
G-CSF	V302-16_1	100	4	49,8
G-CSF	V302-16_2	100	1	11,5
G-CSF	V302-16_3	100	1	11,4
G-CSF	V302-16_4	100	2	28,7
G-CSF	V302-17_1	100	1	11,2
G-CSF	V302-17_2	100	4	47,7
G-CSF	V302-17_3	100	3	36
G-CSF	V303-15_1	100	1	10,6
G-CSF	V303-15_2	100	4	57,1
G-CSF	V303-15_3	100	2	21,9
G-CSF	V303-16_1	100	3	34,4
G-CSF	V303-16_2	100	1	17,7
G-CSF	V303-16_3	100	2	31,2
G-CSF	V303-17_1	100	5	56

apical dendrite Sholl 110μm

Group	number	Radius(μm)	Intersections	Length(μm)
Control	V215-11_1	110	2	30,5
Control	V215-12_1	110	2	32
Control	V215-12_2	110	1	21,8
Control	V215-13_1	110	3	32,6
Control	V215-15_1	110	2	35,1
Control	V215-15_2	110	5	76,3
Control	V215-16_1	110	2	22,2
Control	V215-18_1	110	1	16,2
Control	V216-11_1	110	3	55,3
Control	V216-12_1	110	2	28
Control	V216-13_1	110	1	10,9
Control	V216-14_1	110	2	26,7
Control	V216-14_2	110	2	29,6
Control	V216-16_1	110	1	14
Control	V216-16_2	110	3	86,7
Control	V216-17_1	110	2	28,8
Control	V216-18_1	110	1	13,2
Control	V216-18_2	110	2	24,5
Control	V219-6_1	110	2	24,3
Control	V219-11_1	110	1	19
Control	V219-12_1	110	3	36,8
Control	V219-13_1	110	2	40,5
Control	V219-13_2	110	1	15
Control	V219-14_1	110	2	60,8
Control	V219-14_2	110	4	42,2
Control	V219-16_1	110	2	16,3
Control	V297-19_1	110	3	72
Control	V297-19_2	110	1	10,3
Control	V297-20_1	110	2	26
Control	V297-20_2	110	1	11,1
Control	V297-20_3	110	3	27,2
Control	V297-22_1	110	5	63,2
Control	V297-22_2	110	1	11,2
Control	V298-15_1	110	6	90,9
Control	V298-16_1	110	5	62,1
Control	V298-16_2	110	3	35,6
Control	V298-16_3	110	1	10,2
Control	V298-17_1	110	1	11,5
Control	V298-17_2	110	3	35,9
Control	V298-17_3	110	2	28,1
Control	V308-17_1	110	1	11,5
Control	V308-17_2	110	2	23,1
Control	V308-17_3	110	2	23,3
Control	V308-17_4	110	1	10,6
Control	V308-18_1	110	1	22,8
Control	V308-18_2	110	2	35,3
Control	V308-18_3	110	11	145,1
CIMT	V211-10_1	110	7	89,2
CIMT	V211-10_2	110	2	34
CIMT	V211-11_1	110	3	82,7
CIMT	V211-11_2	110	3	38,4
CIMT	V211-12_1	110	2	31,4

CIMT	V211-13_1	110	3	38,7
CIMT	V211-13_2	110	5	71,4
CIMT	V211-15_1	110	1	14
CIMT	V211-15_2	110	1	12
CIMT	V211-16_1	110	5	77
CIMT	V212-14_1	110	4	53,3
CIMT	V212-14_2	110	6	88,5
CIMT	V212-15_1	110	7	80,9
CIMT	V212-18_1	110	4	63,8
CIMT	V212-21_1	110	2	24,4
CIMT	V212-21_2	110	4	55,6
CIMT	V212-22_1	110	3	36,8
CIMT	V212-23_1	110	5	53,3
CIMT	V212-23_2	110	3	33,7
CIMT	V212-24_1	110	4	46,3
CIMT	V218-9_1	110	1	22,6
CIMT	V218-10_1	110	1	12,6
CIMT	V218-11_1	110	1	10,3
CIMT	V218-12_1	110	2	58,5
CIMT	V218-12_2	110	2	23
CIMT	V218-15_1	110	2	25
CIMT	V218-17_1	110	6	94,3
CIMT	V299-14_1	110	3	34,4
CIMT	V299-15_1	110	1	22,1
CIMT	V299-15_2	110	2	22,7
CIMT	V299-16_1	110	1	10
CIMT	V299-16_2	110	1	11,6
CIMT	V299-17_1	110	10	115
CIMT	V299-17_2	110	1	17,5
CIMT	V300-16_1	110	2	21,3
CIMT	V300-18_1	110	4	74,2
CIMT	V300-18_2	110	3	40,1
CIMT	V300-20_1	110	2	28,6
CIMT	V300-22_1	110	3	18,1
CIMT	V300-23_1	110	2	21
CIMT	V300-23_2	110	4	49
CIMT	V309-15_1	110	1	10,8
CIMT	V309-15_2	110	6	57,9
CIMT	V309-16_1	110	5	65,1
CIMT	V309-16_2	110	1	29,4
CIMT	V309-16_3	110	6	82,4
CIMT	V309-17_1	110	3	34,5
CIMT	V309-17_2	110	3	50,2
CIMT+G-CSF	V213-13_1	110	3	33,5
CIMT+G-CSF	V213-16_1	110	1	14
CIMT+G-CSF	V213-17_1	110	1	10,3
CIMT+G-CSF	V213-17_2	110	3	38
CIMT+G-CSF	V213-18_1	110	5	99,4
CIMT+G-CSF	V213-18_2	110	2	22,7
CIMT+G-CSF	V213-19_1	110	2	27,6
CIMT+G-CSF	V213-19_2	110	3	49,1
CIMT+G-CSF	V213-20_1	110	1	10,2
CIMT+G-CSF	V213-22_1	110	2	38,4
CIMT+G-CSF	V213-23_1	110	1	10,1
CIMT+G-CSF	V214-14_1	110	1	24,7
CIMT+G-CSF	V214-17_1	110	3	36

CIMT+G-CSF	V214-18_1	110	3	33,6
CIMT+G-CSF	V214-19_1	110	2	40,7
CIMT+G-CSF	V214-19_2	110	6	70,2
CIMT+G-CSF	V214-20_1	110	4	47,8
CIMT+G-CSF	V214-21_1	110	2	27
CIMT+G-CSF	V217-1_1	110	1	11,7
CIMT+G-CSF	V217-6_1	110	3	60,6
CIMT+G-CSF	V217-6_3	110	3	41,1
CIMT+G-CSF	V217-8_1	110	2	24,5
CIMT+G-CSF	V217-8_4	110	2	23,6
CIMT+G-CSF	V217-10_1	110	1	21,9
CIMT+G-CSF	V217-11_1	110	1	10,2
CIMT+G-CSF	V304-17_1	110	1	14,9
CIMT+G-CSF	V304-17_2	110	1	11,2
CIMT+G-CSF	V304-17_3	110	2	23,1
CIMT+G-CSF	V304-17_4	110	1	10,7
CIMT+G-CSF	V304-19_1	110	2	23,9
CIMT+G-CSF	V304-19_2	110	0	3,9
CIMT+G-CSF	V305-17_1	110	4	27,6
CIMT+G-CSF	V305-17_2	110	1	11,4
CIMT+G-CSF	V305-17_3	110	2	12,6
CIMT+G-CSF	V305-18_1	110	1	11,4
CIMT+G-CSF	V305-18_2	110	2	20,7
CIMT+G-CSF	V305-18_3	110	1	37,8
CIMT+G-CSF	V305-18_4	110	1	10,5
CIMT+G-CSF	V305-19_1	110	1	15,3
CIMT/G-CSF	V306-16_1	110	4	46,6
CIMT/G-CSF	V306-16_2	110	2	26,3
CIMT/G-CSF	V306-17_1	110	7	94,9
CIMT/G-CSF	V306-17_2	110	1	10,6
CIMT/G-CSF	V306-17_3	110	4	40,7
CIMT/G-CSF	V306-17_4	110	3	45,9
CIMT/G-CSF	V306-17_5	110	1	20
CIMT/G-CSF	V311-16_1	110	6	97,5
CIMT/G-CSF	V311-17_1	110	4	51,9
CIMT/G-CSF	V311-17_2	110	2	46,9
CIMT/G-CSF	V311-19_1	110	1	10,7
CIMT/G-CSF	V311-20_1	110	4	48,3
CIMT/G-CSF	V311-20_2	110	2	31,8
CIMT/G-CSF	V311-20_3	110	2	21,7
CIMT/G-CSF	V313-14_1	110	4	55,3
CIMT/G-CSF	V313-15_1	110	0	7,8
CIMT/G-CSF	V313-15_2	110	1	10,5
CIMT/G-CSF	V313-17_1	110	1	10
CIMT/G-CSF	V313-17_2	110	2	21,4
CIMT/G-CSF	V313-17_3	110	4	35,1
CIMT/G-CSF	V313-19_1	110	1	19,4
CIMT/G-CSF	V314-16_1	110	4	75,8
CIMT/G-CSF	V314-16_2	110	2	35,7
CIMT/G-CSF	V314-17_1	110	4	58,5
CIMT/G-CSF	V314-17_2	110	3	56,7
CIMT/G-CSF	V314-17_3	110	3	44,4
CIMT/G-CSF	V314-18_1	110	1	15,1
CIMT/G-CSF	V314-18_2	110	1	20,4
CIMT/G-CSF	V315-11_1	110	1	15,1
CIMT/G-CSF	V315-11_2	110	1	10,8

CIMT/G-CSF	V315-13_1	110	3	87,8
CIMT/G-CSF	V315-13_2	110	5	84,9
CIMT/G-CSF	V315-14_1	110	0	5,1
CIMT/G-CSF	V315-14_2	110	2	27,8
CIMT/G-CSF	V315-14_3	110	3	37,2
G-CSF	V220-9_1	110	5	32
G-CSF	V220-11_1	110	1	11,6
G-CSF	V220-12_1	110	3	46,7
G-CSF	V220-16_2	110	2	28,6
G-CSF	V220-17_1	110	3	52,2
G-CSF	V220-18_1	110	2	54,2
G-CSF	V221-5_1	110	0	9
G-CSF	V221-6_1	110	4	74,8
G-CSF	V221-7_1	110	2	38,1
G-CSF	V221-7_2	110	8	116,5
G-CSF	V221-11_1	110	3	36,6
G-CSF	V221-13_1	110	10	122,3
G-CSF	V221-15_1	110	3	30,6
G-CSF	V221-20_1	110	0	5
G-CSF	V222-19_1	110	2	24,1
G-CSF	V222-19_2	110	2	33,4
G-CSF	V222-20_1	110	3	49,9
G-CSF	V222-20_2	110	1	10,7
G-CSF	V222-21_1	110	3	37,2
G-CSF	V222-22_1	110	5	148,5
G-CSF	V222-22_2	110	1	10,2
G-CSF	V222-23_1	110	4	67,2
G-CSF	V222-24_1	110	2	24,3
G-CSF	V301-18_1	110	1	12,4
G-CSF	V301-18_2	110	2	31,9
G-CSF	V301-18_3	110	1	10,6
G-CSF	V301-19_1	110	3	57
G-CSF	V301-19_2	110	1	24,4
G-CSF	V301-20_1	110	3	91,7
G-CSF	V301-20_2	110	5	70,3
G-CSF	V302-15_1	110	3	51,9
G-CSF	V302-15_2	110	1	15,3
G-CSF	V302-15_3	110	5	115,2
G-CSF	V302-16_1	110	2	33,1
G-CSF	V302-16_2	110	1	13,4
G-CSF	V302-16_3	110	1	14,4
G-CSF	V302-16_4	110	2	25
G-CSF	V302-17_1	110	1	10,5
G-CSF	V302-17_2	110	5	90
G-CSF	V302-17_3	110	2	35,2
G-CSF	V303-15_1	110	1	11,8
G-CSF	V303-15_2	110	5	69,1
G-CSF	V303-15_3	110	2	21,5
G-CSF	V303-16_1	110	3	32,9
G-CSF	V303-16_2	110	1	10,5
G-CSF	V303-16_3	110	2	32,3
G-CSF	V303-17_1	110	7	104

apical dendrite Sholl 120µm

Group	number	Radius(µm)	Intersections	Length(µm)
Control	V215-11_1	120	0	21,6
Control	V215-12_1	120	1	29,8
Control	V215-12_2	120	1	10,8
Control	V215-13_1	120	1	24,3
Control	V215-15_1	120	2	23,5
Control	V215-15_2	120	5	75,9
Control	V215-16_1	120	2	23,7
Control	V215-18_1	120	1	11,3
Control	V216-11_1	120	3	41,1
Control	V216-12_1	120	1	22,7
Control	V216-13_1	120	1	12,8
Control	V216-14_1	120	3	37,5
Control	V216-14_2	120	1	35,4
Control	V216-16_1	120	1	11,6
Control	V216-16_2	120	2	43
Control	V216-17_1	120	1	21,8
Control	V216-18_1	120	1	10,6
Control	V216-18_2	120	3	27,1
Control	V219-6_1	120	2	23,7
Control	V219-11_1	120	0	0
Control	V219-12_1	120	2	27
Control	V219-13_1	120	1	22,8
Control	V219-13_2	120	1	10,4
Control	V219-14_1	120	4	73,2
Control	V219-14_2	120	6	107,1
Control	V219-16_1	120	1	21,5
Control	V297-19_1	120	4	70,8
Control	V297-19_2	120	1	10,5
Control	V297-20_1	120	1	10,6
Control	V297-20_2	120	1	13,3
Control	V297-20_3	120	3	40,7
Control	V297-22_1	120	5	50,7
Control	V297-22_2	120	1	10,4
Control	V298-15_1	120	4	60,6
Control	V298-16_1	120	2	42,1
Control	V298-16_2	120	3	36,7
Control	V298-16_3	120	1	10,9
Control	V298-17_1	120	1	10,4
Control	V298-17_2	120	3	38,6
Control	V298-17_3	120	1	10,3
Control	V308-17_1	120	1	10,5
Control	V308-17_2	120	2	27,6
Control	V308-17_3	120	2	24
Control	V308-17_4	120	4	33,7
Control	V308-18_1	120	1	18,9
Control	V308-18_2	120	2	24,7
Control	V308-18_3	120	11	151,2
CIMT	V211-10_1	120	7	179
CIMT	V211-10_2	120	2	23,4
CIMT	V211-11_1	120	3	41,5
CIMT	V211-11_2	120	3	48,9
CIMT	V211-12_1	120	3	34,4
CIMT	V211-13_1	120	3	39,7

CIMT	V211-13_2	120	5	72
CIMT	V211-15_1	120	2	51,2
CIMT	V211-15_2	120	1	10,6
CIMT	V211-16_1	120	3	50,4
CIMT	V212-14_1	120	3	40,8
CIMT	V212-14_2	120	6	89,1
CIMT	V212-15_1	120	7	82,8
CIMT	V212-18_1	120	4	57,7
CIMT	V212-21_1	120	2	36
CIMT	V212-21_2	120	4	49,6
CIMT	V212-22_1	120	3	33,6
CIMT	V212-23_1	120	5	71
CIMT	V212-23_2	120	4	40,5
CIMT	V212-24_1	120	4	45
CIMT	V218-9_1	120	1	13,9
CIMT	V218-10_1	120	1	15,1
CIMT	V218-11_1	120	2	21,3
CIMT	V218-12_1	120	2	24,6
CIMT	V218-12_2	120	2	21,6
CIMT	V218-15_1	120	2	24,5
CIMT	V218-17_1	120	5	68,1
CIMT	V299-14_1	120	2	31,1
CIMT	V299-15_1	120	1	17,1
CIMT	V299-15_2	120	2	23,7
CIMT	V299-16_1	120	1	13,9
CIMT	V299-16_2	120	1	10,6
CIMT	V299-17_1	120	9	109,4
CIMT	V299-17_2	120	1	10,8
CIMT	V300-16_1	120	2	24
CIMT	V300-18_1	120	3	45,3
CIMT	V300-18_2	120	2	53
CIMT	V300-20_1	120	3	32,9
CIMT	V300-22_1	120	3	43,7
CIMT	V300-23_1	120	2	20,6
CIMT	V300-23_2	120	3	34,3
CIMT	V309-15_1	120	2	14,4
CIMT	V309-15_2	120	5	76,7
CIMT	V309-16_1	120	3	74,9
CIMT	V309-16_2	120	2	44,6
CIMT	V309-16_3	120	6	95,2
CIMT	V309-17_1	120	1	47,3
CIMT	V309-17_2	120	2	32,1
CIMT+G-CSF	V213-13_1	120	4	35,9
CIMT+G-CSF	V213-16_1	120	1	10,8
CIMT+G-CSF	V213-17_1	120	1	11,1
CIMT+G-CSF	V213-17_2	120	3	40,2
CIMT+G-CSF	V213-18_1	120	3	39,2
CIMT+G-CSF	V213-18_2	120	2	21,4
CIMT+G-CSF	V213-19_1	120	2	39,5
CIMT+G-CSF	V213-19_2	120	1	31,2
CIMT+G-CSF	V213-20_1	120	2	12,1
CIMT+G-CSF	V213-22_1	120	2	37,7
CIMT+G-CSF	V213-23_1	120	0	7
CIMT+G-CSF	V214-14_1	120	1	11,7
CIMT+G-CSF	V214-17_1	120	2	31,8
CIMT+G-CSF	V214-18_1	120	3	33

CIMT+G-CSF	V214-19_1	120	2	20,5
CIMT+G-CSF	V214-19_2	120	4	78,5
CIMT+G-CSF	V214-20_1	120	2	49,3
CIMT+G-CSF	V214-21_1	120	2	23,3
CIMT+G-CSF	V217-1_1	120	1	12,3
CIMT+G-CSF	V217-6_1	120	5	84,7
CIMT+G-CSF	V217-6_3	120	3	41,6
CIMT+G-CSF	V217-8_1	120	1	19,2
CIMT+G-CSF	V217-8_4	120	2	39,1
CIMT+G-CSF	V217-10_1	120	1	13,4
CIMT+G-CSF	V217-11_1	120	1	10,5
CIMT+G-CSF	V304-17_1	120	1	10,5
CIMT+G-CSF	V304-17_2	120	1	11,1
CIMT+G-CSF	V304-17_3	120	2	22,1
CIMT+G-CSF	V304-17_4	120	1	11,6
CIMT+G-CSF	V304-19_1	120	2	23,1
CIMT+G-CSF	V305-17_1	120	2	39,6
CIMT+G-CSF	V305-17_2	120	1	14
CIMT+G-CSF	V305-17_3	120	3	58,2
CIMT+G-CSF	V305-18_1	120	0	2,6
CIMT+G-CSF	V305-18_2	120	1	13,4
CIMT+G-CSF	V305-18_3	120	1	14,2
CIMT+G-CSF	V305-18_4	120	1	12,3
CIMT+G-CSF	V305-19_1	120	1	11,8
CIMT/G-CSF	V306-16_1	120	4	51,9
CIMT/G-CSF	V306-16_2	120	1	22,3
CIMT/G-CSF	V306-17_1	120	6	75,4
CIMT/G-CSF	V306-17_2	120	2	22
CIMT/G-CSF	V306-17_3	120	3	75,6
CIMT/G-CSF	V306-17_4	120	3	36,8
CIMT/G-CSF	V306-17_5	120	1	10,7
CIMT/G-CSF	V311-16_1	120	6	86,8
CIMT/G-CSF	V311-17_1	120	3	53,1
CIMT/G-CSF	V311-17_2	120	2	34
CIMT/G-CSF	V311-19_1	120	1	10,4
CIMT/G-CSF	V311-20_1	120	4	46,6
CIMT/G-CSF	V311-20_2	120	0	5,8
CIMT/G-CSF	V311-20_3	120	2	26,5
CIMT/G-CSF	V313-14_1	120	3	38,2
CIMT/G-CSF	V313-15_2	120	1	10,5
CIMT/G-CSF	V313-17_1	120	1	10
CIMT/G-CSF	V313-17_2	120	1	20,9
CIMT/G-CSF	V313-17_3	120	5	63,2
CIMT/G-CSF	V313-19_1	120	2	21,2
CIMT/G-CSF	V314-16_1	120	2	37,1
CIMT/G-CSF	V314-16_2	120	1	23,1
CIMT/G-CSF	V314-17_1	120	3	62,6
CIMT/G-CSF	V314-17_2	120	3	33
CIMT/G-CSF	V314-17_3	120	2	37,9
CIMT/G-CSF	V314-18_1	120	1	10,5
CIMT/G-CSF	V314-18_2	120	1	10,1
CIMT/G-CSF	V315-11_1	120	1	10,5
CIMT/G-CSF	V315-11_2	120	1	13,2
CIMT/G-CSF	V315-13_1	120	2	77,4
CIMT/G-CSF	V315-13_2	120	2	53,3
CIMT/G-CSF	V315-14_2	120	2	20,9

CIMT/G-CSF	V315-14_3	120	2	32,7
G-CSF	V220-9_1	120	4	122,1
G-CSF	V220-11_1	120	1	10,2
G-CSF	V220-12_1	120	3	54
G-CSF	V220-16_2	120	2	22,8
G-CSF	V220-17_1	120	3	44,1
G-CSF	V220-18_1	120	2	31
G-CSF	V221-6_1	120	5	60,9
G-CSF	V221-7_1	120	2	23,5
G-CSF	V221-7_2	120	5	97,8
G-CSF	V221-11_1	120	3	42,4
G-CSF	V221-13_1	120	13	183,6
G-CSF	V221-15_1	120	3	40,1
G-CSF	V222-19_1	120	2	22,5
G-CSF	V222-19_2	120	2	25,6
G-CSF	V222-20_1	120	3	46,4
G-CSF	V222-20_2	120	1	11,3
G-CSF	V222-21_1	120	3	39,9
G-CSF	V222-22_1	120	5	84,9
G-CSF	V222-22_2	120	2	30,2
G-CSF	V222-23_1	120	8	76,5
G-CSF	V222-24_1	120	2	26,7
G-CSF	V301-18_1	120	1	14,3
G-CSF	V301-18_2	120	2	27
G-CSF	V301-18_3	120	1	10,3
G-CSF	V301-19_1	120	3	37,3
G-CSF	V301-19_2	120	1	12,8
G-CSF	V301-20_1	120	3	32,5
G-CSF	V301-20_2	120	3	81,5
G-CSF	V302-15_1	120	2	44,5
G-CSF	V302-15_2	120	1	10,3
G-CSF	V302-15_3	120	4	55
G-CSF	V302-16_1	120	2	22,2
G-CSF	V302-16_2	120	1	10,3
G-CSF	V302-16_3	120	1	10,4
G-CSF	V302-16_4	120	1	13,3
G-CSF	V302-17_1	120	1	24,7
G-CSF	V302-17_2	120	3	151,1
G-CSF	V302-17_3	120	2	23,2
G-CSF	V303-15_1	120	1	11,2
G-CSF	V303-15_2	120	3	61
G-CSF	V303-15_3	120	2	21
G-CSF	V303-16_1	120	3	31,9
G-CSF	V303-16_2	120	1	10,8
G-CSF	V303-16_3	120	2	28,2
G-CSF	V303-17_1	120	5	145

apical dendrite Sholl 130µm

Group	number	Radius(µm)	Intersections	Length(µm)
Control	V215-12_1	130	2	32,6
Control	V215-12_2	130	0	3,8
Control	V215-13_1	130	0	1,5
Control	V215-15_1	130	2	21,5
Control	V215-15_2	130	5	64,1
Control	V215-16_1	130	1	25,1
Control	V215-18_1	130	1	10,6
Control	V216-11_1	130	3	35,7
Control	V216-12_1	130	0	0,7
Control	V216-13_1	130	1	10,4
Control	V216-14_1	130	1	38,5
Control	V216-14_2	130	1	10,2
Control	V216-16_1	130	1	12,8
Control	V216-16_2	130	1	20,6
Control	V216-17_1	130	2	17,4
Control	V216-18_1	130	2	20,6
Control	V216-18_2	130	3	36,6
Control	V219-6_1	130	1	36,9
Control	V219-12_1	130	2	22
Control	V219-13_1	130	1	23,9
Control	V219-13_2	130	1	10,3
Control	V219-14_1	130	2	44,8
Control	V219-14_2	130	4	64,2
Control	V219-16_1	130	1	10,1
Control	V297-19_1	130	4	53,1
Control	V297-19_2	130	1	11,2
Control	V297-20_1	130	1	10,2
Control	V297-20_2	130	0	2
Control	V297-20_3	130	2	32,5
Control	V297-22_1	130	4	60,8
Control	V297-22_2	130	1	10,2
Control	V298-15_1	130	4	94,8
Control	V298-16_1	130	4	48,9
Control	V298-16_2	130	2	27,5
Control	V298-16_3	130	1	10,1
Control	V298-17_1	130	1	10,3
Control	V298-17_2	130	3	39,1
Control	V298-17_3	130	1	10,3
Control	V308-17_1	130	1	11
Control	V308-17_2	130	1	22,9
Control	V308-17_3	130	2	22,3
Control	V308-17_4	130	2	53,9
Control	V308-18_1	130	1	15,3
Control	V308-18_2	130	2	21,8
Control	V308-18_3	130	8	109,2
CIMT	V211-10_1	130	4	96,7
CIMT	V211-10_2	130	2	21,2
CIMT	V211-11_1	130	4	47,5
CIMT	V211-11_2	130	3	34,3
CIMT	V211-12_1	130	3	40,8
CIMT	V211-13_1	130	2	31,5
CIMT	V211-13_2	130	3	51,3
CIMT	V211-15_1	130	1	26,2

CIMT	V211-15_2	130	1	10,3
CIMT	V211-16_1	130	2	38,8
CIMT	V212-14_1	130	2	37,7
CIMT	V212-14_2	130	6	89,9
CIMT	V212-15_1	130	6	77,2
CIMT	V212-18_1	130	3	51,6
CIMT	V212-21_1	130	2	50,9
CIMT	V212-21_2	130	4	64,6
CIMT	V212-22_1	130	4	38,4
CIMT	V212-23_1	130	2	126,9
CIMT	V212-23_2	130	3	35,5
CIMT	V212-24_1	130	3	48,1
CIMT	V218-9_1	130	1	12,1
CIMT	V218-10_1	130	1	32,7
CIMT	V218-11_1	130	2	28
CIMT	V218-12_1	130	2	22,3
CIMT	V218-12_2	130	3	102,6
CIMT	V218-15_1	130	2	22,7
CIMT	V218-17_1	130	2	51,8
CIMT	V299-14_1	130	2	23,9
CIMT	V299-15_1	130	3	15,5
CIMT	V299-15_2	130	1	21,3
CIMT	V299-16_1	130	2	19,4
CIMT	V299-16_2	130	1	11,1
CIMT	V299-17_1	130	7	96,4
CIMT	V299-17_2	130	1	12,8
CIMT	V300-16_1	130	2	23,3
CIMT	V300-18_1	130	6	70,2
CIMT	V300-18_2	130	2	22,6
CIMT	V300-20_1	130	3	44
CIMT	V300-22_1	130	3	37,9
CIMT	V300-23_1	130	1	16,2
CIMT	V300-23_2	130	3	48,1
CIMT	V309-15_1	130	2	40,5
CIMT	V309-15_2	130	4	59
CIMT	V309-16_1	130	2	42,8
CIMT	V309-16_2	130	1	16,4
CIMT	V309-16_3	130	4	75,6
CIMT	V309-17_1	130	1	10,3
CIMT	V309-17_2	130	2	20,8
CIMT+G-CSF	V213-13_1	130	3	34,2
CIMT+G-CSF	V213-16_1	130	1	11,4
CIMT+G-CSF	V213-17_1	130	1	10,3
CIMT+G-CSF	V213-17_2	130	3	39,2
CIMT+G-CSF	V213-18_1	130	3	43,5
CIMT+G-CSF	V213-18_2	130	2	22
CIMT+G-CSF	V213-19_1	130	2	29,8
CIMT+G-CSF	V213-19_2	130	2	18,7
CIMT+G-CSF	V213-20_1	130	1	33,2
CIMT+G-CSF	V213-22_1	130	1	50,8
CIMT+G-CSF	V214-14_1	130	1	11
CIMT+G-CSF	V214-17_1	130	2	26,1
CIMT+G-CSF	V214-18_1	130	2	30,8
CIMT+G-CSF	V214-19_1	130	2	34,6
CIMT+G-CSF	V214-19_2	130	4	55,2
CIMT+G-CSF	V214-20_1	130	1	19,3

CIMT+G-CSF	V214-21_1	130	1	20,3
CIMT+G-CSF	V217-1_1	130	0	6,3
CIMT+G-CSF	V217-6_1	130	5	103,2
CIMT+G-CSF	V217-6_3	130	3	38,2
CIMT+G-CSF	V217-8_1	130	1	13,9
CIMT+G-CSF	V217-8_4	130	2	21,7
CIMT+G-CSF	V217-10_1	130	1	10,1
CIMT+G-CSF	V217-11_1	130	1	12,3
CIMT+G-CSF	V304-17_1	130	1	16,7
CIMT+G-CSF	V304-17_2	130	1	10,7
CIMT+G-CSF	V304-17_3	130	2	20,9
CIMT+G-CSF	V304-17_4	130	2	22
CIMT+G-CSF	V304-19_1	130	2	24,9
CIMT+G-CSF	V305-17_1	130	2	30,6
CIMT+G-CSF	V305-17_2	130	1	10,8
CIMT+G-CSF	V305-17_3	130	0	36,9
CIMT+G-CSF	V305-18_2	130	1	10,1
CIMT+G-CSF	V305-18_3	130	1	10,8
CIMT+G-CSF	V305-18_4	130	1	11,5
CIMT+G-CSF	V305-19_1	130	1	10,6
CIMT/G-CSF	V306-16_1	130	3	47,6
CIMT/G-CSF	V306-16_2	130	1	10,6
CIMT/G-CSF	V306-17_1	130	7	76,4
CIMT/G-CSF	V306-17_2	130	2	26,1
CIMT/G-CSF	V306-17_3	130	3	46,6
CIMT/G-CSF	V306-17_4	130	3	36,3
CIMT/G-CSF	V306-17_5	130	1	11,3
CIMT/G-CSF	V311-16_1	130	6	90
CIMT/G-CSF	V311-17_1	130	4	54,5
CIMT/G-CSF	V311-17_2	130	2	37,3
CIMT/G-CSF	V311-19_1	130	1	10,7
CIMT/G-CSF	V311-20_1	130	4	45,7
CIMT/G-CSF	V311-20_3	130	2	21,4
CIMT/G-CSF	V313-14_1	130	3	42,4
CIMT/G-CSF	V313-15_2	130	1	10,5
CIMT/G-CSF	V313-17_1	130	1	10,1
CIMT/G-CSF	V313-17_2	130	0	0,8
CIMT/G-CSF	V313-17_3	130	2	48,2
CIMT/G-CSF	V313-19_1	130	2	96,8
CIMT/G-CSF	V314-16_1	130	1	24,3
CIMT/G-CSF	V314-16_2	130	0	8,1
CIMT/G-CSF	V314-17_1	130	2	56,3
CIMT/G-CSF	V314-17_2	130	2	38,7
CIMT/G-CSF	V314-17_3	130	2	20,8
CIMT/G-CSF	V314-18_1	130	1	10,5
CIMT/G-CSF	V314-18_2	130	1	12,8
CIMT/G-CSF	V315-11_1	130	1	10,2
CIMT/G-CSF	V315-11_2	130	1	11,8
CIMT/G-CSF	V315-13_1	130	3	45,7
CIMT/G-CSF	V315-13_2	130	2	24,4
CIMT/G-CSF	V315-14_2	130	1	20,3
CIMT/G-CSF	V315-14_3	130	2	23,9
G-CSF	V220-9_1	130	3	43,4
G-CSF	V220-11_1	130	1	12,8
G-CSF	V220-12_1	130	3	39,3
G-CSF	V220-16_2	130	1	20,7

G-CSF	V220-17_1	130	1	21,6
G-CSF	V220-18_1	130	2	23,1
G-CSF	V221-6_1	130	4	46,7
G-CSF	V221-7_1	130	1	22,5
G-CSF	V221-7_2	130	5	56,3
G-CSF	V221-11_1	130	3	32,6
G-CSF	V221-13_1	130	8	188
G-CSF	V221-15_1	130	3	36,1
G-CSF	V222-19_1	130	2	14,1
G-CSF	V222-19_2	130	2	23,3
G-CSF	V222-20_1	130	3	40,5
G-CSF	V222-20_2	130	0	8,7
G-CSF	V222-21_1	130	3	33,3
G-CSF	V222-22_1	130	6	76,1
G-CSF	V222-22_2	130	2	29,7
G-CSF	V222-23_1	130	6	156,4
G-CSF	V222-24_1	130	4	55,4
G-CSF	V301-18_1	130	3	22,5
G-CSF	V301-18_2	130	2	32
G-CSF	V301-18_3	130	1	10,6
G-CSF	V301-19_1	130	2	40,1
G-CSF	V301-19_2	130	1	11,4
G-CSF	V301-20_1	130	3	35
G-CSF	V301-20_2	130	3	34,5
G-CSF	V302-15_1	130	1	19,4
G-CSF	V302-15_2	130	1	10,6
G-CSF	V302-15_3	130	3	37,7
G-CSF	V302-16_1	130	2	21,2
G-CSF	V302-16_2	130	1	10,9
G-CSF	V302-16_3	130	1	10,4
G-CSF	V302-16_4	130	4	68,7
G-CSF	V302-17_1	130	1	10,4
G-CSF	V302-17_2	130	2	41,3
G-CSF	V302-17_3	130	1	12,9
G-CSF	V303-15_1	130	1	10,9
G-CSF	V303-15_2	130	2	43,6
G-CSF	V303-15_3	130	2	21,2
G-CSF	V303-16_1	130	3	33,4
G-CSF	V303-16_2	130	1	10,2
G-CSF	V303-16_3	130	1	32,6
G-CSF	V303-17_1	130	5	65,3

apical dendrite Sholl 140μm

Group	number	Radius(μm)	Intersections	Length(μm)
Control	V215-12_1	140	2	31,4
Control	V215-15_1	140	3	46,6
Control	V215-15_2	140	2	72,7
Control	V215-16_1	140	1	10,3
Control	V215-18_1	140	0	0,8
Control	V216-11_1	140	4	41,8
Control	V216-13_1	140	1	10,3
Control	V216-14_1	140	1	10,6
Control	V216-14_2	140	1	10,8
Control	V216-16_1	140	1	11
Control	V216-16_2	140	1	10,4
Control	V216-17_1	140	2	42,2
Control	V216-18_1	140	1	23,6
Control	V216-18_2	140	3	34,2
Control	V219-6_1	140	1	11,2
Control	V219-12_1	140	4	125
Control	V219-13_1	140	1	10,5
Control	V219-13_2	140	1	10,4
Control	V219-14_1	140	2	24,6
Control	V219-14_2	140	2	68,5
Control	V219-16_1	140	1	10,5
Control	V297-19_1	140	3	40,4
Control	V297-19_2	140	1	10,6
Control	V297-20_1	140	1	10,5
Control	V297-20_3	140	1	31,1
Control	V297-22_1	140	3	45,4
Control	V297-22_2	140	1	10,3
Control	V298-15_1	140	2	31,1
Control	V298-16_1	140	2	45,7
Control	V298-16_2	140	1	17,5
Control	V298-16_3	140	1	13,1
Control	V298-17_1	140	2	28
Control	V298-17_2	140	3	41
Control	V298-17_3	140	2	14,6
Control	V308-17_1	140	1	11
Control	V308-17_2	140	1	14,9
Control	V308-17_3	140	1	10,9
Control	V308-17_4	140	1	35,2
Control	V308-18_1	140	1	10,6
Control	V308-18_2	140	2	22,2
Control	V308-18_3	140	6	95,4
CIMT	V211-10_1	140	3	41,4
CIMT	V211-10_2	140	1	21,7
CIMT	V211-11_1	140	4	75,2
CIMT	V211-11_2	140	2	46,6
CIMT	V211-12_1	140	3	42,7
CIMT	V211-13_1	140	1	24,9
CIMT	V211-13_2	140	5	45,5
CIMT	V211-15_1	140	1	11
CIMT	V211-15_2	140	2	22,7
CIMT	V211-16_1	140	1	17,9
CIMT	V212-14_1	140	2	46,7
CIMT	V212-14_2	140	5	79,2

CIMT	V212-15_1	140	5	68,3
CIMT	V212-18_1	140	3	37
CIMT	V212-21_1	140	1	23,7
CIMT	V212-21_2	140	5	59,8
CIMT	V212-22_1	140	4	47,3
CIMT	V212-23_1	140	2	24,6
CIMT	V212-23_2	140	3	31,5
CIMT	V212-24_1	140	3	33,4
CIMT	V218-9_1	140	1	10,7
CIMT	V218-10_1	140	1	20,5
CIMT	V218-11_1	140	1	38,9
CIMT	V218-12_1	140	1	14,4
CIMT	V218-12_2	140	3	43,3
CIMT	V218-15_1	140	3	37,5
CIMT	V218-17_1	140	1	13,6
CIMT	V299-14_1	140	2	23,4
CIMT	V299-15_1	140	2	87,4
CIMT	V299-15_2	140	1	12,9
CIMT	V299-16_1	140	2	21,4
CIMT	V299-16_2	140	2	15
CIMT	V299-17_1	140	5	56,9
CIMT	V299-17_2	140	1	11
CIMT	V300-16_1	140	2	22,5
CIMT	V300-18_1	140	3	62,2
CIMT	V300-18_2	140	1	15,1
CIMT	V300-20_1	140	2	41,2
CIMT	V300-22_1	140	1	29,4
CIMT	V300-23_1	140	1	10,3
CIMT	V300-23_2	140	2	57,8
CIMT	V309-15_1	140	2	64,9
CIMT	V309-15_2	140	4	55
CIMT	V309-16_1	140	4	157,7
CIMT	V309-16_2	140	1	10,4
CIMT	V309-16_3	140	3	39,3
CIMT	V309-17_1	140	1	10,5
CIMT	V309-17_2	140	2	21,1
CIMT+G-CSF	V213-13_1	140	2	26,6
CIMT+G-CSF	V213-16_1	140	1	10,6
CIMT+G-CSF	V213-17_1	140	1	10,2
CIMT+G-CSF	V213-17_2	140	2	30,3
CIMT+G-CSF	V213-18_1	140	2	45,9
CIMT+G-CSF	V213-18_2	140	2	21,9
CIMT+G-CSF	V213-19_1	140	2	29,4
CIMT+G-CSF	V213-19_2	140	4	45,7
CIMT+G-CSF	V213-20_1	140	1	11
CIMT+G-CSF	V213-22_1	140	1	10,2
CIMT+G-CSF	V214-14_1	140	1	10,9
CIMT+G-CSF	V214-17_1	140	2	24,5
CIMT+G-CSF	V214-18_1	140	2	22,7
CIMT+G-CSF	V214-19_1	140	2	23
CIMT+G-CSF	V214-19_2	140	3	45,7
CIMT+G-CSF	V214-20_1	140	1	10,9
CIMT+G-CSF	V214-21_1	140	1	10,4
CIMT+G-CSF	V217-6_1	140	4	79,1
CIMT+G-CSF	V217-6_3	140	3	33,5
CIMT+G-CSF	V217-8_1	140	2	14,2

CIMT+G-CSF	V217-8_4	140	2	20,6
CIMT+G-CSF	V217-10_1	140	1	12,7
CIMT+G-CSF	V217-11_1	140	1	12,8
CIMT+G-CSF	V304-17_1	140	1	12
CIMT+G-CSF	V304-17_2	140	1	11,2
CIMT+G-CSF	V304-17_3	140	0	11,9
CIMT+G-CSF	V304-17_4	140	2	31,6
CIMT+G-CSF	V304-19_1	140	1	19,1
CIMT+G-CSF	V305-17_1	140	2	24,2
CIMT+G-CSF	V305-17_2	140	1	12,2
CIMT+G-CSF	V305-18_2	140	1	11,2
CIMT+G-CSF	V305-18_3	140	1	10,2
CIMT+G-CSF	V305-18_4	140	1	10,3
CIMT+G-CSF	V305-19_1	140	1	11,3
CIMT/G-CSF	V306-16_1	140	3	35,1
CIMT/G-CSF	V306-16_2	140	1	11,3
CIMT/G-CSF	V306-17_1	140	7	85
CIMT/G-CSF	V306-17_2	140	2	26,4
CIMT/G-CSF	V306-17_3	140	2	23,3
CIMT/G-CSF	V306-17_4	140	3	45,3
CIMT/G-CSF	V306-17_5	140	1	10,4
CIMT/G-CSF	V311-16_1	140	10	113,3
CIMT/G-CSF	V311-17_1	140	5	98,5
CIMT/G-CSF	V311-17_2	140	1	29,9
CIMT/G-CSF	V311-19_1	140	4	98,1
CIMT/G-CSF	V311-20_1	140	3	36,7
CIMT/G-CSF	V311-20_3	140	2	20,6
CIMT/G-CSF	V313-14_1	140	3	47
CIMT/G-CSF	V313-15_2	140	0	40,9
CIMT/G-CSF	V313-17_1	140	1	10,3
CIMT/G-CSF	V313-17_3	140	1	16,5
CIMT/G-CSF	V313-19_1	140	1	25,1
CIMT/G-CSF	V314-16_1	140	1	12,3
CIMT/G-CSF	V314-17_1	140	1	43,6
CIMT/G-CSF	V314-17_2	140	2	28,9
CIMT/G-CSF	V314-17_3	140	2	34,2
CIMT/G-CSF	V314-18_1	140	1	11,1
CIMT/G-CSF	V314-18_2	140	0	5,3
CIMT/G-CSF	V315-11_1	140	1	13,9
CIMT/G-CSF	V315-11_2	140	1	10,1
CIMT/G-CSF	V315-13_1	140	3	56,2
CIMT/G-CSF	V315-13_2	140	3	38,1
CIMT/G-CSF	V315-14_2	140	1	10,7
CIMT/G-CSF	V315-14_3	140	1	20,8
G-CSF	V220-9_1	140	3	35,2
G-CSF	V220-11_1	140	1	12,8
G-CSF	V220-12_1	140	3	39,5
G-CSF	V220-16_2	140	1	31,9
G-CSF	V220-17_1	140	0	1,9
G-CSF	V220-18_1	140	2	24,2
G-CSF	V221-6_1	140	5	54,2
G-CSF	V221-7_1	140	0	8,6
G-CSF	V221-7_2	140	4	57,2
G-CSF	V221-11_1	140	3	36,3
G-CSF	V221-13_1	140	7	92,8
G-CSF	V221-15_1	140	2	45,6

G-CSF	V222-19_1	140	2	24,1
G-CSF	V222-19_2	140	2	20,7
G-CSF	V222-20_1	140	5	41,2
G-CSF	V222-21_1	140	3	34,4
G-CSF	V222-22_1	140	5	76,9
G-CSF	V222-22_2	140	3	47,9
G-CSF	V222-23_1	140	5	76,6
G-CSF	V222-24_1	140	4	46,6
G-CSF	V301-18_1	140	1	31,3
G-CSF	V301-18_2	140	1	12
G-CSF	V301-18_3	140	1	11,9
G-CSF	V301-19_1	140	3	34,1
G-CSF	V301-19_2	140	1	11,4
G-CSF	V301-20_1	140	3	36,6
G-CSF	V301-20_2	140	2	26,7
G-CSF	V302-15_1	140	1	10,7
G-CSF	V302-15_2	140	1	10,9
G-CSF	V302-15_3	140	3	38,5
G-CSF	V302-16_1	140	2	23,3
G-CSF	V302-16_2	140	1	11,2
G-CSF	V302-16_3	140	1	10,3
G-CSF	V302-16_4	140	1	29,8
G-CSF	V302-17_1	140	1	11,3
G-CSF	V302-17_2	140	2	23,2
G-CSF	V302-17_3	140	1	10,9
G-CSF	V303-15_1	140	2	38,8
G-CSF	V303-15_2	140	1	19,5
G-CSF	V303-15_3	140	1	18,5
G-CSF	V303-16_1	140	2	50,1
G-CSF	V303-16_2	140	1	12,2
G-CSF	V303-16_3	140	1	11,2
G-CSF	V303-17_1	140	5	58,3

apical dendrite Sholl 150μm

Group	number	Radius(μm)	Intersections	Length(μm)
Control	V215-12_1	150	2	24,6
Control	V215-15_1	150	6	43,1
Control	V215-15_2	150	4	54,1
Control	V215-16_1	150	1	11,5
Control	V216-11_1	150	3	35,1
Control	V216-13_1	150	0	8,5
Control	V216-14_1	150	1	10,1
Control	V216-14_2	150	1	12,6
Control	V216-16_1	150	0	9,9
Control	V216-16_2	150	1	10,2
Control	V216-17_1	150	2	21,7
Control	V216-18_1	150	1	26,3
Control	V216-18_2	150	2	29,6
Control	V219-6_1	150	1	10,8
Control	V219-12_1	150	2	67,2
Control	V219-13_1	150	1	10,9
Control	V219-13_2	150	1	11,5
Control	V219-14_1	150	2	23,7
Control	V219-14_2	150	3	31,3
Control	V219-16_1	150	1	10,2
Control	V297-19_1	150	2	42,1
Control	V297-19_2	150	1	12,9
Control	V297-20_1	150	1	10,8
Control	V297-20_3	150	1	13,6
Control	V297-22_1	150	3	38,3
Control	V297-22_2	150	0	9,6
Control	V298-15_1	150	3	24,8
Control	V298-16_1	150	2	43,3
Control	V298-16_2	150	1	12
Control	V298-16_3	150	3	65,7
Control	V298-17_1	150	1	16,9
Control	V298-17_2	150	3	59,8
Control	V298-17_3	150	2	22,8
Control	V308-17_1	150	1	15,7
Control	V308-17_2	150	1	10,7
Control	V308-17_3	150	3	26,9
Control	V308-17_4	150	1	12
Control	V308-18_1	150	1	10,6
Control	V308-18_2	150	0	9
Control	V308-18_3	150	3	69,3
CIMT	V211-10_1	150	2	49,4
CIMT	V211-10_2	150	0	8,9
CIMT	V211-11_1	150	5	78,9
CIMT	V211-11_2	150	2	23,7
CIMT	V211-12_1	150	2	25
CIMT	V211-13_1	150	2	26,1
CIMT	V211-13_2	150	5	76,9
CIMT	V211-15_1	150	1	11,5
CIMT	V211-15_2	150	4	35,5
CIMT	V211-16_1	150	1	10,5
CIMT	V212-14_1	150	2	34,3
CIMT	V212-14_2	150	6	58,2
CIMT	V212-15_1	150	4	65,3

CIMT	V212-18_1	150	2	29,5
CIMT	V212-21_1	150	1	24,9
CIMT	V212-21_2	150	5	61,6
CIMT	V212-22_1	150	3	42,2
CIMT	V212-23_1	150	2	31,8
CIMT	V212-23_2	150	3	32,5
CIMT	V212-24_1	150	3	31,8
CIMT	V218-9_1	150	1	10,8
CIMT	V218-10_1	150	1	18,3
CIMT	V218-11_1	150	1	11,3
CIMT	V218-12_1	150	1	11,3
CIMT	V218-12_2	150	3	46
CIMT	V218-15_1	150	3	37,2
CIMT	V218-17_1	150	1	12,9
CIMT	V299-14_1	150	2	21,9
CIMT	V299-15_1	150	2	37,9
CIMT	V299-15_2	150	1	10,7
CIMT	V299-16_1	150	2	33,3
CIMT	V299-16_2	150	1	21,7
CIMT	V299-17_1	150	5	56,8
CIMT	V299-17_2	150	1	10
CIMT	V300-16_1	150	2	20,5
CIMT	V300-18_1	150	2	26,3
CIMT	V300-18_2	150	0	3,3
CIMT	V300-20_1	150	2	25,8
CIMT	V300-22_1	150	2	18,3
CIMT	V300-23_1	150	1	10,8
CIMT	V300-23_2	150	2	24,3
CIMT	V309-15_1	150	1	21,9
CIMT	V309-15_2	150	3	89,5
CIMT	V309-16_1	150	1	32,6
CIMT	V309-16_2	150	2	71,3
CIMT	V309-16_3	150	4	44,8
CIMT	V309-17_1	150	2	15,8
CIMT	V309-17_2	150	1	17,9
CIMT+G-CSF	V213-13_1	150	1	20,3
CIMT+G-CSF	V213-16_1	150	1	10,7
CIMT+G-CSF	V213-17_1	150	1	10,2
CIMT+G-CSF	V213-17_2	150	2	22,3
CIMT+G-CSF	V213-18_1	150	2	28,8
CIMT+G-CSF	V213-18_2	150	2	21,3
CIMT+G-CSF	V213-19_1	150	2	39,7
CIMT+G-CSF	V213-19_2	150	2	31,3
CIMT+G-CSF	V213-20_1	150	1	10,1
CIMT+G-CSF	V213-22_1	150	1	10,1
CIMT+G-CSF	V214-14_1	150	1	11,3
CIMT+G-CSF	V214-17_1	150	2	21,5
CIMT+G-CSF	V214-18_1	150	2	27,3
CIMT+G-CSF	V214-19_1	150	1	15,1
CIMT+G-CSF	V214-19_2	150	4	45,2
CIMT+G-CSF	V214-20_1	150	1	10,2
CIMT+G-CSF	V214-21_1	150	1	10,9
CIMT+G-CSF	V217-6_1	150	2	35,8
CIMT+G-CSF	V217-6_3	150	2	35,5
CIMT+G-CSF	V217-8_1	150	2	66,3
CIMT+G-CSF	V217-8_4	150	2	29,3

CIMT+G-CSF	V217-10_1	150	1	12,1
CIMT+G-CSF	V217-11_1	150	1	11,6
CIMT+G-CSF	V304-17_1	150	0	7
CIMT+G-CSF	V304-17_2	150	1	11,5
CIMT+G-CSF	V304-17_4	150	2	40,4
CIMT+G-CSF	V304-19_1	150	1	11
CIMT+G-CSF	V305-17_1	150	2	31
CIMT+G-CSF	V305-17_2	150	1	12,1
CIMT+G-CSF	V305-18_2	150	1	11,3
CIMT+G-CSF	V305-18_3	150	1	13,3
CIMT+G-CSF	V305-18_4	150	1	10,4
CIMT+G-CSF	V305-19_1	150	1	11,9
CIMT/G-CSF	V306-16_1	150	3	35
CIMT/G-CSF	V306-16_2	150	1	11,5
CIMT/G-CSF	V306-17_1	150	5	87,6
CIMT/G-CSF	V306-17_2	150	2	26,1
CIMT/G-CSF	V306-17_3	150	1	18,9
CIMT/G-CSF	V306-17_4	150	2	25,4
CIMT/G-CSF	V306-17_5	150	1	10,3
CIMT/G-CSF	V311-16_1	150	5	66,2
CIMT/G-CSF	V311-17_1	150	5	96,1
CIMT/G-CSF	V311-17_2	150	1	15,9
CIMT/G-CSF	V311-19_1	150	6	118,9
CIMT/G-CSF	V311-20_1	150	2	56,3
CIMT/G-CSF	V311-20_3	150	2	20,4
CIMT/G-CSF	V313-14_1	150	2	39,9
CIMT/G-CSF	V313-17_1	150	1	10,2
CIMT/G-CSF	V313-17_3	150	1	11,7
CIMT/G-CSF	V313-19_1	150	1	10,2
CIMT/G-CSF	V314-16_1	150	0	5,8
CIMT/G-CSF	V314-17_1	150	1	11,5
CIMT/G-CSF	V314-17_2	150	3	43,9
CIMT/G-CSF	V314-17_3	150	2	35,1
CIMT/G-CSF	V314-18_1	150	2	23
CIMT/G-CSF	V315-11_1	150	1	10,2
CIMT/G-CSF	V315-11_2	150	5	58,2
CIMT/G-CSF	V315-13_1	150	3	45,3
CIMT/G-CSF	V315-13_2	150	1	50,4
CIMT/G-CSF	V315-14_2	150	1	15,5
CIMT/G-CSF	V315-14_3	150	1	10,4
G-CSF	V220-9_1	150	3	34
G-CSF	V220-11_1	150	1	11,3
G-CSF	V220-12_1	150	3	50,6
G-CSF	V220-16_2	150	1	11,5
G-CSF	V220-18_1	150	2	28,5
G-CSF	V221-6_1	150	6	82
G-CSF	V221-7_2	150	4	44,2
G-CSF	V221-11_1	150	2	30,6
G-CSF	V221-13_1	150	7	85,2
G-CSF	V221-15_1	150	1	16,9
G-CSF	V222-19_1	150	2	25,7
G-CSF	V222-19_2	150	2	22,9
G-CSF	V222-20_1	150	3	53,4
G-CSF	V222-21_1	150	3	39,5
G-CSF	V222-22_1	150	6	79,9
G-CSF	V222-22_2	150	3	64,9

G-CSF	V222-23_1	150	6	136,7
G-CSF	V222-24_1	150	4	47
G-CSF	V301-18_1	150	1	10,5
G-CSF	V301-18_2	150	1	10,8
G-CSF	V301-18_3	150	1	11,3
G-CSF	V301-19_1	150	1	21,2
G-CSF	V301-19_2	150	1	11,2
G-CSF	V301-20_1	150	3	42,4
G-CSF	V301-20_2	150	2	29,1
G-CSF	V302-15_1	150	1	10,8
G-CSF	V302-15_2	150	2	21,2
G-CSF	V302-15_3	150	2	36,2
G-CSF	V302-16_1	150	2	22,2
G-CSF	V302-16_2	150	2	31,7
G-CSF	V302-16_3	150	1	81,7
G-CSF	V302-16_4	150	1	11,2
G-CSF	V302-17_1	150	1	12,6
G-CSF	V302-17_2	150	2	20,9
G-CSF	V302-17_3	150	1	13,3
G-CSF	V303-15_1	150	1	40,2
G-CSF	V303-15_2	150	1	10,6
G-CSF	V303-15_3	150	1	10,7
G-CSF	V303-16_1	150	1	24,9
G-CSF	V303-16_2	150	1	15,8
G-CSF	V303-16_3	150	3	22
G-CSF	V303-17_1	150	6	83,6

basilar dendrite Sholl 10μm

Group	number	Radius(μm)	Intersections	Length(μm)
Control	V215-10_1	10	4	6,8
Control	V215-10_2	10	0	0
Control	V215-11_1	10	4	14,5
Control	V215-12_1	10	0	0
Control	V215-12_2	10	4	6
Control	V215-13_1	10	3	4,6
Control	V215-15_1	10	3	11,6
Control	V215-15_2	10	0	0
Control	V215-16_1	10	4	9,9
Control	V215-18_1	10	6	12
Control	V216-11_1	10	0	0
Control	V216-12_1	10	2	5,3
Control	V216-13_1	10	3	4,4
Control	V216-14_1	10	1	1,3
Control	V216-14_2	10	0	0
Control	V216-16_1	10	4	8,2
Control	V216-16_2	10	4	3
Control	V216-17_1	10	0	0
Control	V216-18_1	10	5	22,5
Control	V216-18_2	10	2	2,1
Control	V219-6_1	10	0	0
Control	V219-11_1	10	0	0
Control	V219-12_1	10	2	2,4
Control	V219-13_1	10	2	4,9
Control	V219-13_2	10	5	17

Control	V219-14_1	10	1	0,1
Control	V219-14_2	10	2	1,9
Control	V219-16_1	10	4	4,6
Control	V297-19_1	10	1	1,5
Control	V297-19_2	10	0	0
Control	V297-20_1	10	0	0
Control	V297-20_2	10	3	5,2
Control	V297-20_3	10	0	0
Control	V297-22_1	10	2	2,1
Control	V297-22_2	10	3	5,1
Control	V298-15_1	10	1	2,6
Control	V298-16_1	10	1	1
Control	V298-16_2	10	0	0
Control	V298-16_3	10	0	0
Control	V298-17_1	10	6	10,2
Control	V298-17_2	10	2	4,2
Control	V298-17_3	10	3	2,6
Control	V308-17_1	10	4	7,4
Control	V308-17_2	10	0	0
Control	V308-17_3	10	4	5,9
Control	V308-17_4	10	5	14,2
Control	V308-18_1	10	3	4,9
Control	V308-18_2	10	2	4,2
Control	V308-18_3	10	1	3,1
CIMT	V211-10_1	10	1	0,6
CIMT	V211-10_2	10	0	0
CIMT	V211-11_1	10	0	0
CIMT	V211-11_2	10	0	0
CIMT	V211-12_1	10	2	3,4
CIMT	V211-13_1	10	1	5,1
CIMT	V211-13_2	10	2	1,6
CIMT	V211-15_1	10	6	12,4
CIMT	V211-15_2	10	3	2,9
CIMT	V211-16_1	10	2	5,2
CIMT	V212-14_1	10	1	0,6
CIMT	V212-14_2	10	3	0,6
CIMT	V212-15_1	10	3	2
CIMT	V212-18_1	10	0	0
CIMT	V212-21_1	10	0	0
CIMT	V212-21_2	10	0	0
CIMT	V212-22_1	10	2	2,2
CIMT	V212-23_1	10	2	22,2
CIMT	V212-23_2	10	0	0
CIMT	V212-24_1	10	2	0,6
CIMT	V218-9_1	10	0	0
CIMT	V218-10_1	10	0	0
CIMT	V218-11_1	10	0	0
CIMT	V218-12_1	10	2	3,3
CIMT	V218-12_2	10	0	0
CIMT	V218-13_1	10	0	0
CIMT	V218-15_1	10	4	4,9
CIMT	V218-17_1	10	1	1,1
CIMT	V299-14_1	10	2	3,6
CIMT	V299-15_1	10	0	0
CIMT	V299-15_2	10	1	1
CIMT	V299-16_1	10	1	3

CIMT	V299-16_2	10	2	4,3
CIMT	V299-17_1	10	1	2,4
CIMT	V299-17_2	10	2	6,8
CIMT	V300-16_1	10	0	0
CIMT	V300-18_1	10	5	9,2
CIMT	V300-18_2	10	6	9,9
CIMT	V300-20_1	10	4	3,6
CIMT	V300-22_1	10	5	11,2
CIMT	V300-23_1	10	4	10,2
CIMT	V300-23_2	10	1	6
CIMT	V309-15_1	10	5	12,8
CIMT	V309-15_2	10	4	13,3
CIMT	V309-16_1	10	0	0
CIMT	V309-16_2	10	3	4,4
CIMT	V309-16_3	10	3	5,7
CIMT	V309-17_1	10	7	21
CIMT	V309-17_2	10	5	9,1
CIMT+G-CSF	V213-13_1	10	4	14,4
CIMT+G-CSF	V213-16_1	10	3	8,3
CIMT+G-CSF	V213-17_1	10	6	24,9
CIMT+G-CSF	V213-17_2	10	3	1,4
CIMT+G-CSF	V213-18_1	10	2	1,6
CIMT+G-CSF	V213-18_2	10	0	0
CIMT+G-CSF	V213-19_1	10	0	0
CIMT+G-CSF	V213-19_2	10	7	24,7
CIMT+G-CSF	V213-20_1	10	4	8,1
CIMT+G-CSF	V213-22_1	10	2	1,5
CIMT+G-CSF	V213-23_1	10	2	7,2
CIMT+G-CSF	V214-14_1	10	4	12,9
CIMT+G-CSF	V214-17_1	10	3	4,8
CIMT+G-CSF	V214-18_1	10	0	0
CIMT+G-CSF	V214-19_1	10	5	16,5
CIMT+G-CSF	V214-19_2	10	2	1,1
CIMT+G-CSF	V214-20_1	10	3	5
CIMT+G-CSF	V214-21_1	10	1	2,8
CIMT+G-CSF	V217-1_1	10	0	0
CIMT+G-CSF	V217-2_1	10	2	0,8
CIMT+G-CSF	V217-4_1	10	0	0
CIMT+G-CSF	V217-6_1	10	4	7,1
CIMT+G-CSF	V217-6_3	10	0	0
CIMT+G-CSF	V217-8_1	10	3	6
CIMT+G-CSF	V217-8_4	10	0	0
CIMT+G-CSF	V217-10_1	10	1	0,2
CIMT+G-CSF	V217-11_1	10	0	0
CIMT+G-CSF	V304-16_1	10	7	24,2
CIMT+G-CSF	V304-17_1	10	2	6,6
CIMT+G-CSF	V304-17_2	10	2	10,6
CIMT+G-CSF	V304-17_3	10	1	0,9
CIMT+G-CSF	V304-17_4	10	0	0
CIMT+G-CSF	V304-19_1	10	3	8,8
CIMT+G-CSF	V304-19_2	10	5	26,7
CIMT+G-CSF	V305-17_1	10	3	2,7
CIMT+G-CSF	V305-17_2	10	0	0
CIMT+G-CSF	V305-17_3	10	5	12
CIMT+G-CSF	V305-18_1	10	6	15,5
CIMT+G-CSF	V305-18_2	10	4	23,2

CIMT+G-CSF	V305-18_3	10	3	5,3
CIMT+G-CSF	V305-18_4	10	3	9,7
CIMT+G-CSF	V305-19_1	10	1	3,1
CIMT/G-CSF	V306-16_1	10	4	10,1
CIMT/G-CSF	V306-16_2	10	5	8,7
CIMT/G-CSF	V306-17_1	10	5	6,5
CIMT/G-CSF	V306-17_2	10	2	4,9
CIMT/G-CSF	V306-17_3	10	4	10
CIMT/G-CSF	V306-17_4	10	5	21,6
CIMT/G-CSF	V306-17_5	10	4	12,3
CIMT/G-CSF	V311-16_1	10	0	0
CIMT/G-CSF	V311-17_1	10	0	0
CIMT/G-CSF	V311-17_2	10	3	8,7
CIMT/G-CSF	V311-19_1	10	0	0
CIMT/G-CSF	V311-20_1	10	0	0
CIMT/G-CSF	V311-20_2	10	0	0
CIMT/G-CSF	V311-20_3	10	1	1
CIMT/G-CSF	V313-14_1	10	2	4,6
CIMT/G-CSF	V313-15_1	10	6	18
CIMT/G-CSF	V313-15_2	10	1	1,2
CIMT/G-CSF	V313-17_1	10	2	2,4
CIMT/G-CSF	V313-17_2	10	0	0
CIMT/G-CSF	V313-17_3	10	2	8,1
CIMT/G-CSF	V313-19_1	10	5	8,5
CIMT/G-CSF	V314-16_1	10	3	8,8
CIMT/G-CSF	V314-16_2	10	0	0
CIMT/G-CSF	V314-17_1	10	3	3,3
CIMT/G-CSF	V314-17_2	10	4	27,3
CIMT/G-CSF	V314-17_3	10	7	23,9
CIMT/G-CSF	V314-18_1	10	7	8,4
CIMT/G-CSF	V314-18_2	10	1	6,9
CIMT/G-CSF	V315-11_1	10	1	7,5
CIMT/G-CSF	V315-11_2	10	2	5,1
CIMT/G-CSF	V315-13_1	10	0	0
CIMT/G-CSF	V315-13_2	10	0	0
CIMT/G-CSF	V315-14_1	10	4	11,7
CIMT/G-CSF	V315-14_2	10	0	0
CIMT/G-CSF	V315-14_3	10	0	0
G-CSF	V220-9_1	10	6	18,1
G-CSF	V220-11_1	10	2	7,8
G-CSF	V220-12_1	10	1	1,4
G-CSF	V220-16_1	10	0	0
G-CSF	V220-16_2	10	4	17,9
G-CSF	V220-17_1	10	1	1,6
G-CSF	V220-18_1	10	7	21
G-CSF	V221-2_1	10	3	4,2
G-CSF	V221-4_1	10	1	0,7
G-CSF	V221-5_1	10	1	2,4
G-CSF	V221-6_1	10	1	4,3
G-CSF	V221-7_1	10	5	19,9
G-CSF	V221-7_2	10	2	1,8
G-CSF	V221-11_1	10	2	2
G-CSF	V221-13_1	10	6	6,5
G-CSF	V221-15_1	10	2	1,7
G-CSF	V221-20_1	10	2	3,9
G-CSF	V222-19_1	10	4	8,9

G-CSF	V222-19_2	10	1	0,3
G-CSF	V222-20_1	10	0	0
G-CSF	V222-20_2	10	1	1,1
G-CSF	V222-21_1	10	1	0,7
G-CSF	V222-22_1	10	0	0
G-CSF	V222-22_2	10	0	0
G-CSF	V222-23_1	10	0	0
G-CSF	V222-24_1	10	0	0
G-CSF	V301-18_1	10	2	1,7
G-CSF	V301-18_2	10	4	14,8
G-CSF	V301-18_3	10	1	0,6
G-CSF	V301-19_1	10	5	13,4
G-CSF	V301-19_2	10	4	13,8
G-CSF	V301-20_1	10	4	7,6
G-CSF	V301-20_2	10	7	24,1
G-CSF	V302-15_1	10	3	5,6
G-CSF	V302-15_2	10	0	0
G-CSF	V302-15_3	10	2	10
G-CSF	V302-16_1	10	0	0
G-CSF	V302-16_2	10	2	3,2
G-CSF	V302-16_3	10	4	9,3
G-CSF	V302-16_4	10	0	0
G-CSF	V302-17_1	10	0	0
G-CSF	V302-17_2	10	2	5,6
G-CSF	V302-17_3	10	3	13,3
G-CSF	V303-15_1	10	0	0
G-CSF	V303-15_2	10	0	0
G-CSF	V303-15_3	10	0	0
G-CSF	V303-16_1	10	1	2,1
G-CSF	V303-16_2	10	3	7,5
G-CSF	V303-16_3	10	1	3,5
G-CSF	V303-17_1	10	3	5,5

basilar dendrite Sholl 20μm

Group	number	Radius(μm)	Intersections	Length(μm)
Control	V215-10_1	20	9	111
Control	V215-10_2	20	10	68,6
Control	V215-11_1	20	9	107,9
Control	V215-12_1	20	1	0,8
Control	V215-12_2	20	8	100,9
Control	V215-13_1	20	13	129,2
Control	V215-15_1	20	7	75,4
Control	V215-15_2	20	6	55,1
Control	V215-16_1	20	6	57,5
Control	V215-18_1	20	10	120,8
Control	V216-11_1	20	8	44,2
Control	V216-12_1	20	6	63,9
Control	V216-13_1	20	6	77,7
Control	V216-14_1	20	9	95,8
Control	V216-14_2	20	7	73
Control	V216-16_1	20	10	98
Control	V216-16_2	20	8	106,5
Control	V216-17_1	20	4	50,2
Control	V216-18_1	20	6	108,2

Control	V216-18_2	20	9	72,8
Control	V219-6_1	20	7	42
Control	V219-11_1	20	5	33,2
Control	V219-12_1	20	10	101,9
Control	V219-13_1	20	4	51
Control	V219-13_2	20	10	164,4
Control	V219-14_1	20	11	142,8
Control	V219-14_2	20	7	72,5
Control	V219-16_1	20	8	108
Control	V297-19_1	20	13	86,6
Control	V297-19_2	20	5	41,3
Control	V297-20_1	20	10	52,1
Control	V297-20_2	20	7	62,3
Control	V297-20_3	20	4	17,1
Control	V297-22_1	20	8	94,2
Control	V297-22_2	20	7	88,8
Control	V298-15_1	20	9	82,6
Control	V298-16_1	20	4	40,7
Control	V298-16_2	20	4	11,8
Control	V298-16_3	20	4	14,5
Control	V298-17_1	20	7	95,9
Control	V298-17_2	20	6	59,1
Control	V298-17_3	20	13	146,9
Control	V308-17_1	20	8	98,1
Control	V308-17_2	20	2	15,1
Control	V308-17_3	20	9	100,6
Control	V308-17_4	20	9	86,8
Control	V308-18_1	20	14	145,2
Control	V308-18_2	20	9	65,1
Control	V308-18_3	20	8	76
CIMT	V211-10_1	20	8	52,9
CIMT	V211-10_2	20	8	60
CIMT	V211-11_1	20	3	56,9
CIMT	V211-11_2	20	7	61,7
CIMT	V211-12_1	20	12	146,3
CIMT	V211-13_1	20	4	40,2
CIMT	V211-13_2	20	5	48
CIMT	V211-15_1	20	10	116,8
CIMT	V211-15_2	20	15	122,9
CIMT	V211-16_1	20	10	82,3
CIMT	V212-14_1	20	3	33,3
CIMT	V212-14_2	20	5	44,6
CIMT	V212-15_1	20	6	76
CIMT	V212-18_1	20	7	61,1
CIMT	V212-21_1	20	6	57,5
CIMT	V212-21_2	20	8	34,8
CIMT	V212-22_1	20	6	50,5
CIMT	V212-23_1	20	6	62,3
CIMT	V212-23_2	20	2	34,9
CIMT	V212-24_1	20	4	55,1
CIMT	V218-9_1	20	8	57,5
CIMT	V218-10_1	20	9	93,4
CIMT	V218-11_1	20	6	33,6
CIMT	V218-12_1	20	12	93,8
CIMT	V218-12_2	20	9	46,7
CIMT	V218-13_1	20	15	147,1

CIMT	V218-15_1	20	9	84,1
CIMT	V218-17_1	20	10	96,6
CIMT	V299-14_1	20	10	83,5
CIMT	V299-15_1	20	5	16,7
CIMT	V299-15_2	20	8	53,4
CIMT	V299-16_1	20	10	89,4
CIMT	V299-16_2	20	5	37,1
CIMT	V299-17_1	20	12	79,1
CIMT	V299-17_2	20	7	72,7
CIMT	V300-16_1	20	3	12,7
CIMT	V300-18_1	20	8	83,5
CIMT	V300-18_2	20	8	95,1
CIMT	V300-20_1	20	10	137,5
CIMT	V300-22_1	20	12	166,9
CIMT	V300-23_1	20	11	98,3
CIMT	V300-23_2	20	14	147,6
CIMT	V309-15_1	20	8	86,4
CIMT	V309-15_2	20	10	121,2
CIMT	V309-16_1	20	1	16,4
CIMT	V309-16_2	20	6	80,4
CIMT	V309-16_3	20	11	93,8
CIMT	V309-17_1	20	11	118,4
CIMT	V309-17_2	20	9	124,3
CIMT+G-CSF	V213-13_1	20	5	68,7
CIMT+G-CSF	V213-16_1	20	4	45,2
CIMT+G-CSF	V213-17_1	20	8	106,8
CIMT+G-CSF	V213-17_2	20	6	57,8
CIMT+G-CSF	V213-18_1	20	9	72,6
CIMT+G-CSF	V213-18_2	20	9	70,4
CIMT+G-CSF	V213-19_1	20	1	3,3
CIMT+G-CSF	V213-19_2	20	10	131,6
CIMT+G-CSF	V213-20_1	20	7	70,5
CIMT+G-CSF	V213-22_1	20	7	59,4
CIMT+G-CSF	V213-23_1	20	11	139,4
CIMT+G-CSF	V214-14_1	20	15	171,6
CIMT+G-CSF	V214-17_1	20	7	66
CIMT+G-CSF	V214-18_1	20	4	43,2
CIMT+G-CSF	V214-19_1	20	7	86,3
CIMT+G-CSF	V214-19_2	20	6	71,2
CIMT+G-CSF	V214-20_1	20	13	129,4
CIMT+G-CSF	V214-21_1	20	8	61,4
CIMT+G-CSF	V217-1_1	20	14	79
CIMT+G-CSF	V217-2_1	20	12	124,7
CIMT+G-CSF	V217-4_1	20	4	15,4
CIMT+G-CSF	V217-6_1	20	9	116,2
CIMT+G-CSF	V217-6_3	20	7	102,1
CIMT+G-CSF	V217-8_1	20	13	102,5
CIMT+G-CSF	V217-8_4	20	2	9,2
CIMT+G-CSF	V217-10_1	20	12	100,3
CIMT+G-CSF	V217-11_1	20	9	70,5
CIMT+G-CSF	V304-16_1	20	7	114,3
CIMT+G-CSF	V304-17_1	20	8	62,9
CIMT+G-CSF	V304-17_2	20	3	38,9
CIMT+G-CSF	V304-17_3	20	9	99,2
CIMT+G-CSF	V304-17_4	20	5	53,7
CIMT+G-CSF	V304-19_1	20	6	89,4

CIMT+G-CSF	V304-19_2	20	6	94,8
CIMT+G-CSF	V305-17_1	20	6	84,9
CIMT+G-CSF	V305-17_2	20	4	24,3
CIMT+G-CSF	V305-17_3	20	11	153,8
CIMT+G-CSF	V305-18_1	20	11	117,5
CIMT+G-CSF	V305-18_2	20	10	111,3
CIMT+G-CSF	V305-18_3	20	7	45,7
CIMT+G-CSF	V305-18_4	20	7	85,5
CIMT+G-CSF	V305-19_1	20	6	39,1
CIMT/G-CSF	V306-16_1	20	10	145
CIMT/G-CSF	V306-16_2	20	10	112,1
CIMT/G-CSF	V306-17_1	20	12	140,8
CIMT/G-CSF	V306-17_2	20	3	35,8
CIMT/G-CSF	V306-17_3	20	10	127,6
CIMT/G-CSF	V306-17_4	20	11	112,8
CIMT/G-CSF	V306-17_5	20	7	75,4
CIMT/G-CSF	V311-16_1	20	12	92,7
CIMT/G-CSF	V311-17_1	20	13	131,9
CIMT/G-CSF	V311-17_2	20	9	116,6
CIMT/G-CSF	V311-19_1	20	1	17,5
CIMT/G-CSF	V311-20_1	20	7	57,6
CIMT/G-CSF	V311-20_2	20	6	80,8
CIMT/G-CSF	V311-20_3	20	7	42,5
CIMT/G-CSF	V313-14_1	20	3	48,8
CIMT/G-CSF	V313-15_1	20	8	71,1
CIMT/G-CSF	V313-15_2	20	5	66,6
CIMT/G-CSF	V313-17_1	20	9	88,5
CIMT/G-CSF	V313-17_2	20	1	4,8
CIMT/G-CSF	V313-17_3	20	4	71,7
CIMT/G-CSF	V313-19_1	20	9	105,1
CIMT/G-CSF	V314-16_1	20	8	70,8
CIMT/G-CSF	V314-16_2	20	3	26,4
CIMT/G-CSF	V314-17_1	20	12	120,6
CIMT/G-CSF	V314-17_2	20	5	80,2
CIMT/G-CSF	V314-17_3	20	12	136,2
CIMT/G-CSF	V314-18_1	20	12	140,5
CIMT/G-CSF	V314-18_2	20	7	72
CIMT/G-CSF	V315-11_1	20	8	123,9
CIMT/G-CSF	V315-11_2	20	11	116,4
CIMT/G-CSF	V315-13_1	20	3	55
CIMT/G-CSF	V315-13_2	20	8	65,7
CIMT/G-CSF	V315-14_1	20	8	98,6
CIMT/G-CSF	V315-14_2	20	9	79,6
CIMT/G-CSF	V315-14_3	20	11	93,4
G-CSF	V220-9_1	20	11	136,2
G-CSF	V220-11_1	20	6	69,9
G-CSF	V220-12_1	20	5	85,8
G-CSF	V220-16_1	20	1	21,4
G-CSF	V220-16_2	20	11	124,9
G-CSF	V220-17_1	20	4	27,3
G-CSF	V220-18_1	20	8	86,2
G-CSF	V221-2_1	20	11	116,6
G-CSF	V221-4_1	20	5	53
G-CSF	V221-5_1	20	9	79,7
G-CSF	V221-6_1	20	4	24,4
G-CSF	V221-7_1	20	6	70,9

G-CSF	V221-7_2	20	11	106,2
G-CSF	V221-11_1	20	3	28,2
G-CSF	V221-13_1	20	17	181
G-CSF	V221-15_1	20	5	52,8
G-CSF	V221-20_1	20	9	87,9
G-CSF	V222-19_1	20	10	105,9
G-CSF	V222-19_2	20	11	138,4
G-CSF	V222-20_1	20	6	37,2
G-CSF	V222-20_2	20	4	41,5
G-CSF	V222-21_1	20	12	112,2
G-CSF	V222-22_1	20	1	1,5
G-CSF	V222-22_2	20	9	97
G-CSF	V222-23_1	20	8	62,5
G-CSF	V222-24_1	20	5	27,4
G-CSF	V301-18_1	20	11	139,4
G-CSF	V301-18_2	20	7	95,3
G-CSF	V301-18_3	20	5	64,2
G-CSF	V301-19_1	20	8	83,9
G-CSF	V301-19_2	20	11	95
G-CSF	V301-20_1	20	10	135,6
G-CSF	V301-20_2	20	13	179,5
G-CSF	V302-15_1	20	10	105,1
G-CSF	V302-15_2	20	8	104,8
G-CSF	V302-15_3	20	6	70,8
G-CSF	V302-16_1	20	2	17,3
G-CSF	V302-16_2	20	4	68
G-CSF	V302-16_3	20	6	68,1
G-CSF	V302-16_4	20	7	37,6
G-CSF	V302-17_1	20	9	84,9
G-CSF	V302-17_2	20	10	118,7
G-CSF	V302-17_3	20	6	69,2
G-CSF	V303-15_1	20	7	76,8
G-CSF	V303-15_2	20	5	22,6
G-CSF	V303-15_3	20	1	9,3
G-CSF	V303-16_1	20	9	107,5
G-CSF	V303-16_2	20	10	94,1
G-CSF	V303-16_3	20	14	127,6
G-CSF	V303-17_1	20	7	67

basilar dendrite Sholl 30μm

Group	number	Radius(μm)	Intersections	Length(μm)
Control	V215-10_1	30	9	120,5
Control	V215-10_2	30	15	184,8
Control	V215-11_1	30	10	172,1
Control	V215-12_1	30	10	132,2
Control	V215-12_2	30	15	182,9
Control	V215-13_1	30	12	164,4
Control	V215-15_1	30	6	77,8
Control	V215-15_2	30	6	97,2
Control	V215-16_1	30	8	101
Control	V215-18_1	30	9	137,6
Control	V216-11_1	30	11	149
Control	V216-12_1	30	8	88,1
Control	V216-13_1	30	6	95,9

Control	V216-14_1	30	10	146,8
Control	V216-14_2	30	9	107
Control	V216-16_1	30	9	149
Control	V216-16_2	30	8	101,6
Control	V216-17_1	30	11	135,8
Control	V216-18_1	30	7	79,4
Control	V216-18_2	30	12	175,4
Control	V219-6_1	30	10	148,2
Control	V219-11_1	30	9	110,1
Control	V219-12_1	30	13	189,1
Control	V219-13_1	30	8	82,9
Control	V219-13_2	30	10	158,1
Control	V219-14_1	30	9	148,6
Control	V219-14_2	30	12	208,1
Control	V219-16_1	30	10	111
Control	V297-19_1	30	16	198,7
Control	V297-19_2	30	7	124,4
Control	V297-20_1	30	10	164,9
Control	V297-20_2	30	6	87,2
Control	V297-20_3	30	9	162
Control	V297-22_1	30	15	180,3
Control	V297-22_2	30	9	155,3
Control	V298-15_1	30	12	145,3
Control	V298-16_1	30	8	98,4
Control	V298-16_2	30	15	173,2
Control	V298-16_3	30	5	72,6
Control	V298-17_1	30	6	80,1
Control	V298-17_2	30	10	109,2
Control	V298-17_3	30	14	179,4
Control	V308-17_1	30	12	138,4
Control	V308-17_2	30	5	89,2
Control	V308-17_3	30	15	185,8
Control	V308-17_4	30	10	132,1
Control	V308-18_1	30	17	225
Control	V308-18_2	30	9	153,2
Control	V308-18_3	30	11	128,9
CIMT	V211-10_1	30	13	158,4
CIMT	V211-10_2	30	10	168,6
CIMT	V211-11_1	30	6	59,2
CIMT	V211-11_2	30	10	152,6
CIMT	V211-12_1	30	11	198
CIMT	V211-13_1	30	7	58,6
CIMT	V211-13_2	30	11	165,3
CIMT	V211-15_1	30	8	130,1
CIMT	V211-15_2	30	12	254
CIMT	V211-16_1	30	14	188,7
CIMT	V212-14_1	30	6	70,8
CIMT	V212-14_2	30	7	100,1
CIMT	V212-15_1	30	10	115,2
CIMT	V212-18_1	30	11	189,8
CIMT	V212-21_1	30	8	86,4
CIMT	V212-21_2	30	15	187,9
CIMT	V212-22_1	30	9	104,9
CIMT	V212-23_1	30	8	127,1
CIMT	V212-23_2	30	4	46,9
CIMT	V212-24_1	30	4	51,7

CIMT	V218-9_1	30	9	131,4
CIMT	V218-10_1	30	10	132,6
CIMT	V218-11_1	30	8	78,5
CIMT	V218-12_1	30	10	144,9
CIMT	V218-12_2	30	6	85,3
CIMT	V218-13_1	30	11	219,2
CIMT	V218-15_1	30	8	106,2
CIMT	V218-17_1	30	10	158,3
CIMT	V299-14_1	30	10	171,1
CIMT	V299-15_1	30	7	120,2
CIMT	V299-15_2	30	7	109
CIMT	V299-16_1	30	11	168
CIMT	V299-16_2	30	7	87,9
CIMT	V299-17_1	30	16	179,6
CIMT	V299-17_2	30	7	101,4
CIMT	V300-16_1	30	7	56,9
CIMT	V300-18_1	30	11	126,1
CIMT	V300-18_2	30	10	113,8
CIMT	V300-20_1	30	11	161,4
CIMT	V300-22_1	30	12	190,7
CIMT	V300-23_1	30	9	115,9
CIMT	V300-23_2	30	6	135,1
CIMT	V309-15_1	30	7	96,8
CIMT	V309-15_2	30	11	150,7
CIMT	V309-16_1	30	13	54,7
CIMT	V309-16_2	30	8	107,5
CIMT	V309-16_3	30	13	207,3
CIMT	V309-17_1	30	12	158,2
CIMT	V309-17_2	30	11	131
CIMT+G-CSF	V213-13_1	30	5	75,3
CIMT+G-CSF	V213-16_1	30	5	60,1
CIMT+G-CSF	V213-17_1	30	9	120
CIMT+G-CSF	V213-17_2	30	13	180,8
CIMT+G-CSF	V213-18_1	30	16	195,7
CIMT+G-CSF	V213-18_2	30	8	119,6
CIMT+G-CSF	V213-19_1	30	3	26,2
CIMT+G-CSF	V213-19_2	30	11	130,5
CIMT+G-CSF	V213-20_1	30	8	92,6
CIMT+G-CSF	V213-22_1	30	10	172,9
CIMT+G-CSF	V213-23_1	30	15	189,1
CIMT+G-CSF	V214-14_1	30	13	177,1
CIMT+G-CSF	V214-17_1	30	10	117,4
CIMT+G-CSF	V214-18_1	30	9	128,3
CIMT+G-CSF	V214-19_1	30	10	99,5
CIMT+G-CSF	V214-19_2	30	8	84,6
CIMT+G-CSF	V214-20_1	30	13	193,4
CIMT+G-CSF	V214-21_1	30	9	111,5
CIMT+G-CSF	V217-1_1	30	16	252,8
CIMT+G-CSF	V217-2_1	30	16	240,5
CIMT+G-CSF	V217-4_1	30	12	73,5
CIMT+G-CSF	V217-6_1	30	11	168,7
CIMT+G-CSF	V217-6_3	30	12	169,2
CIMT+G-CSF	V217-8_1	30	14	169
CIMT+G-CSF	V217-8_4	30	7	104,9
CIMT+G-CSF	V217-10_1	30	9	143,9
CIMT+G-CSF	V217-11_1	30	11	154,9

CIMT+G-CSF	V304-16_1	30	4	64,1
CIMT+G-CSF	V304-17_1	30	12	178,9
CIMT+G-CSF	V304-17_2	30	2	29,8
CIMT+G-CSF	V304-17_3	30	10	147,3
CIMT+G-CSF	V304-17_4	30	9	78,5
CIMT+G-CSF	V304-19_1	30	4	63,9
CIMT+G-CSF	V304-19_2	30	7	79,9
CIMT+G-CSF	V305-17_1	30	10	124,6
CIMT+G-CSF	V305-17_2	30	8	137,4
CIMT+G-CSF	V305-17_3	30	12	150,9
CIMT+G-CSF	V305-18_1	30	13	168,4
CIMT+G-CSF	V305-18_2	30	10	142,2
CIMT+G-CSF	V305-18_3	30	7	106,4
CIMT+G-CSF	V305-18_4	30	5	76,6
CIMT+G-CSF	V305-19_1	30	5	76,2
CIMT/G-CSF	V306-16_1	30	11	142,9
CIMT/G-CSF	V306-16_2	30	10	153,8
CIMT/G-CSF	V306-17_1	30	11	168,2
CIMT/G-CSF	V306-17_2	30	16	136,9
CIMT/G-CSF	V306-17_3	30	14	179,2
CIMT/G-CSF	V306-17_4	30	13	178,1
CIMT/G-CSF	V306-17_5	30	10	138,6
CIMT/G-CSF	V311-16_1	30	21	270,9
CIMT/G-CSF	V311-17_1	30	19	234,8
CIMT/G-CSF	V311-17_2	30	11	143,1
CIMT/G-CSF	V311-19_1	30	8	94,4
CIMT/G-CSF	V311-20_1	30	19	267
CIMT/G-CSF	V311-20_2	30	7	104,2
CIMT/G-CSF	V311-20_3	30	7	83,4
CIMT/G-CSF	V313-14_1	30	8	68,1
CIMT/G-CSF	V313-15_1	30	8	113,2
CIMT/G-CSF	V313-15_2	30	13	185,3
CIMT/G-CSF	V313-17_1	30	11	137,9
CIMT/G-CSF	V313-17_2	30	3	45,2
CIMT/G-CSF	V313-17_3	30	2	45,4
CIMT/G-CSF	V313-19_1	30	7	103,2
CIMT/G-CSF	V314-16_1	30	8	124,1
CIMT/G-CSF	V314-16_2	30	5	68,7
CIMT/G-CSF	V314-17_1	30	12	177,4
CIMT/G-CSF	V314-17_2	30	4	76,5
CIMT/G-CSF	V314-17_3	30	12	167,9
CIMT/G-CSF	V314-18_1	30	11	146,4
CIMT/G-CSF	V314-18_2	30	10	164,3
CIMT/G-CSF	V315-11_1	30	6	89,6
CIMT/G-CSF	V315-11_2	30	12	169,3
CIMT/G-CSF	V315-13_1	30	6	86,9
CIMT/G-CSF	V315-13_2	30	14	180,3
CIMT/G-CSF	V315-14_1	30	9	105,2
CIMT/G-CSF	V315-14_2	30	10	128,2
CIMT/G-CSF	V315-14_3	30	14	176,5
G-CSF	V220-9_1	30	17	250,9
G-CSF	V220-11_1	30	9	109,6
G-CSF	V220-12_1	30	8	145,3
G-CSF	V220-16_1	30	0	14,5
G-CSF	V220-16_2	30	6	123,8
G-CSF	V220-17_1	30	14	109

G-CSF	V220-18_1	30	8	98,5
G-CSF	V221-2_1	30	13	158,5
G-CSF	V221-4_1	30	6	89,5
G-CSF	V221-5_1	30	9	134,2
G-CSF	V221-6_1	30	9	71,7
G-CSF	V221-7_1	30	7	63,7
G-CSF	V221-7_2	30	15	161,8
G-CSF	V221-11_1	30	4	41,5
G-CSF	V221-13_1	30	21	245,7
G-CSF	V221-15_1	30	6	68,5
G-CSF	V221-20_1	30	11	156,6
G-CSF	V222-19_1	30	10	133,8
G-CSF	V222-19_2	30	8	133,4
G-CSF	V222-20_1	30	7	87,9
G-CSF	V222-20_2	30	6	83,6
G-CSF	V222-21_1	30	11	168
G-CSF	V222-22_1	30	13	119
G-CSF	V222-22_2	30	17	198,8
G-CSF	V222-23_1	30	10	112,3
G-CSF	V222-24_1	30	9	113,8
G-CSF	V301-18_1	30	13	208,9
G-CSF	V301-18_2	30	7	99,7
G-CSF	V301-18_3	30	8	96,7
G-CSF	V301-19_1	30	7	83,6
G-CSF	V301-19_2	30	10	139,1
G-CSF	V301-20_1	30	11	159,9
G-CSF	V301-20_2	30	13	181,2
G-CSF	V302-15_1	30	12	160,1
G-CSF	V302-15_2	30	10	180,4
G-CSF	V302-15_3	30	7	96
G-CSF	V302-16_1	30	8	72,3
G-CSF	V302-16_2	30	4	50,8
G-CSF	V302-16_3	30	8	106
G-CSF	V302-16_4	30	11	108,3
G-CSF	V302-17_1	30	14	150,6
G-CSF	V302-17_2	30	11	158,6
G-CSF	V302-17_3	30	9	92,6
G-CSF	V303-15_1	30	6	109,3
G-CSF	V303-15_2	30	11	96
G-CSF	V303-15_3	30	9	25
G-CSF	V303-16_1	30	12	121,1
G-CSF	V303-16_2	30	9	122,5
G-CSF	V303-16_3	30	14	180,8
G-CSF	V303-17_1	30	12	158,3

basilar dendrite Sholl 40μm

Group	number	Radius(μm)	Intersections	Length(μm)
Control	V215-10_1	40	7	100
Control	V215-10_2	40	18	250,8
Control	V215-11_1	40	11	147,2
Control	V215-12_1	40	9	166,8
Control	V215-12_2	40	16	246,1
Control	V215-13_1	40	12	187,7
Control	V215-15_1	40	5	68,1

Control	V215-15_2	40	7	94,2
Control	V215-16_1	40	8	120,5
Control	V215-18_1	40	7	106,1
Control	V216-11_1	40	9	109,8
Control	V216-12_1	40	9	121,2
Control	V216-13_1	40	5	95,2
Control	V216-14_1	40	10	126,6
Control	V216-14_2	40	8	123,7
Control	V216-16_1	40	3	83,6
Control	V216-16_2	40	8	95,5
Control	V216-17_1	40	8	110,2
Control	V216-18_1	40	5	84,7
Control	V216-18_2	40	9	125,7
Control	V219-6_1	40	11	150,8
Control	V219-11_1	40	15	213,8
Control	V219-12_1	40	12	194,1
Control	V219-13_1	40	12	193,8
Control	V219-13_2	40	8	125,8
Control	V219-14_1	40	6	83,7
Control	V219-14_2	40	19	235,7
Control	V219-16_1	40	8	128,4
Control	V297-19_1	40	21	306,9
Control	V297-19_2	40	13	174,7
Control	V297-20_1	40	9	153,4
Control	V297-20_2	40	4	82,5
Control	V297-20_3	40	10	126,2
Control	V297-22_1	40	16	214,7
Control	V297-22_2	40	8	101,5
Control	V298-15_1	40	17	190,2
Control	V298-16_1	40	8	134,2
Control	V298-16_2	40	11	184,8
Control	V298-16_3	40	6	86,1
Control	V298-17_1	40	4	51,3
Control	V298-17_2	40	12	156,3
Control	V298-17_3	40	15	199,7
Control	V308-17_1	40	10	165,9
Control	V308-17_2	40	16	193,1
Control	V308-17_3	40	12	197,9
Control	V308-17_4	40	10	169,1
Control	V308-18_1	40	16	215,8
Control	V308-18_2	40	9	173
Control	V308-18_3	40	15	182,7
CIMT	V211-10_1	40	23	345,7
CIMT	V211-10_2	40	10	136,4
CIMT	V211-11_1	40	6	76,2
CIMT	V211-11_2	40	6	184,1
CIMT	V211-12_1	40	14	213,9
CIMT	V211-13_1	40	8	115,6
CIMT	V211-13_2	40	15	222,5
CIMT	V211-15_1	40	10	143,5
CIMT	V211-15_2	40	13	170,7
CIMT	V211-16_1	40	11	171,9
CIMT	V212-14_1	40	12	157
CIMT	V212-14_2	40	11	126,9
CIMT	V212-15_1	40	15	181,6
CIMT	V212-18_1	40	12	168,3

CIMT	V212-21_1	40	9	99,5
CIMT	V212-21_2	40	15	200
CIMT	V212-22_1	40	9	124,9
CIMT	V212-23_1	40	9	138,5
CIMT	V212-23_2	40	7	103,9
CIMT	V212-24_1	40	4	52,6
CIMT	V218-9_1	40	8	104,8
CIMT	V218-10_1	40	13	159,3
CIMT	V218-11_1	40	14	203,8
CIMT	V218-12_1	40	15	211,7
CIMT	V218-12_2	40	8	107,1
CIMT	V218-13_1	40	9	202,1
CIMT	V218-15_1	40	11	153,8
CIMT	V218-17_1	40	10	152
CIMT	V299-14_1	40	9	125,4
CIMT	V299-15_1	40	4	99
CIMT	V299-15_2	40	3	75,9
CIMT	V299-16_1	40	7	151
CIMT	V299-16_2	40	8	124,5
CIMT	V299-17_1	40	16	212,5
CIMT	V299-17_2	40	7	151,9
CIMT	V300-16_1	40	9	128,8
CIMT	V300-18_1	40	11	149,8
CIMT	V300-18_2	40	12	129,4
CIMT	V300-20_1	40	10	141,2
CIMT	V300-22_1	40	14	169,6
CIMT	V300-23_1	40	7	99,4
CIMT	V300-23_2	40	6	96,8
CIMT	V309-15_1	40	9	103,7
CIMT	V309-15_2	40	11	133
CIMT	V309-16_1	40	13	251,7
CIMT	V309-16_2	40	7	108,2
CIMT	V309-16_3	40	14	191,3
CIMT	V309-17_1	40	10	134
CIMT	V309-17_2	40	11	142
CIMT+G-CSF	V213-13_1	40	5	58,2
CIMT+G-CSF	V213-16_1	40	4	63,3
CIMT+G-CSF	V213-17_1	40	10	122,1
CIMT+G-CSF	V213-17_2	40	15	201,6
CIMT+G-CSF	V213-18_1	40	19	299,6
CIMT+G-CSF	V213-18_2	40	6	86,3
CIMT+G-CSF	V213-19_1	40	12	124,1
CIMT+G-CSF	V213-19_2	40	7	108,2
CIMT+G-CSF	V213-20_1	40	8	93,3
CIMT+G-CSF	V213-22_1	40	12	134,6
CIMT+G-CSF	V213-23_1	40	9	153,9
CIMT+G-CSF	V214-14_1	40	15	209,2
CIMT+G-CSF	V214-17_1	40	10	141,3
CIMT+G-CSF	V214-18_1	40	12	156,5
CIMT+G-CSF	V214-19_1	40	7	97,3
CIMT+G-CSF	V214-19_2	40	8	92,6
CIMT+G-CSF	V214-20_1	40	12	157,8
CIMT+G-CSF	V214-21_1	40	7	121,8
CIMT+G-CSF	V217-1_1	40	17	219,6
CIMT+G-CSF	V217-2_1	40	14	231,6
CIMT+G-CSF	V217-4_1	40	16	231,6

CIMT+G-CSF	V217-6_1	40	11	199,6
CIMT+G-CSF	V217-6_3	40	14	222,9
CIMT+G-CSF	V217-8_1	40	15	236,6
CIMT+G-CSF	V217-8_4	40	7	116,7
CIMT+G-CSF	V217-10_1	40	9	130,2
CIMT+G-CSF	V217-11_1	40	15	180,1
CIMT+G-CSF	V304-16_1	40	3	59,2
CIMT+G-CSF	V304-17_1	40	13	171
CIMT+G-CSF	V304-17_2	40	3	41,2
CIMT+G-CSF	V304-17_3	40	11	139,1
CIMT+G-CSF	V304-17_4	40	7	98,2
CIMT+G-CSF	V304-19_1	40	3	51,4
CIMT+G-CSF	V304-19_2	40	5	92,2
CIMT+G-CSF	V305-17_1	40	5	85,7
CIMT+G-CSF	V305-17_2	40	7	155,6
CIMT+G-CSF	V305-17_3	40	10	162
CIMT+G-CSF	V305-18_1	40	14	182,1
CIMT+G-CSF	V305-18_2	40	7	118,3
CIMT+G-CSF	V305-18_3	40	6	98,7
CIMT+G-CSF	V305-18_4	40	6	167,7
CIMT+G-CSF	V305-19_1	40	4	85,9
CIMT/G-CSF	V306-16_1	40	11	130,5
CIMT/G-CSF	V306-16_2	40	8	106,7
CIMT/G-CSF	V306-17_1	40	14	201,2
CIMT/G-CSF	V306-17_2	40	13	200,5
CIMT/G-CSF	V306-17_3	40	10	166,2
CIMT/G-CSF	V306-17_4	40	10	182,7
CIMT/G-CSF	V306-17_5	40	5	143,5
CIMT/G-CSF	V311-16_1	40	19	280,6
CIMT/G-CSF	V311-17_1	40	17	309,7
CIMT/G-CSF	V311-17_2	40	8	123,1
CIMT/G-CSF	V311-19_1	40	11	184,6
CIMT/G-CSF	V311-20_1	40	20	312,9
CIMT/G-CSF	V311-20_2	40	7	91,6
CIMT/G-CSF	V311-20_3	40	7	80,5
CIMT/G-CSF	V313-14_1	40	7	105,5
CIMT/G-CSF	V313-15_1	40	3	60,8
CIMT/G-CSF	V313-15_2	40	8	279,9
CIMT/G-CSF	V313-17_1	40	11	141,6
CIMT/G-CSF	V313-17_2	40	7	89
CIMT/G-CSF	V313-17_3	40	2	34,7
CIMT/G-CSF	V313-19_1	40	6	75,1
CIMT/G-CSF	V314-16_1	40	8	113,6
CIMT/G-CSF	V314-16_2	40	5	82,1
CIMT/G-CSF	V314-17_1	40	10	139,2
CIMT/G-CSF	V314-17_2	40	1	27,2
CIMT/G-CSF	V314-17_3	40	11	151,8
CIMT/G-CSF	V314-18_1	40	12	152,8
CIMT/G-CSF	V314-18_2	40	11	158,2
CIMT/G-CSF	V315-11_1	40	7	79,9
CIMT/G-CSF	V315-11_2	40	15	173
CIMT/G-CSF	V315-13_1	40	10	132
CIMT/G-CSF	V315-13_2	40	18	214,3
CIMT/G-CSF	V315-14_1	40	8	124
CIMT/G-CSF	V315-14_2	40	14	197,3
CIMT/G-CSF	V315-14_3	40	15	192,1

G-CSF	V220-9_1	40	15	207,5
G-CSF	V220-11_1	40	5	71,8
G-CSF	V220-12_1	40	8	127
G-CSF	V220-16_1	40	1	1,7
G-CSF	V220-16_2	40	7	78,8
G-CSF	V220-17_1	40	14	197,3
G-CSF	V220-18_1	40	7	94,2
G-CSF	V221-2_1	40	14	180,8
G-CSF	V221-4_1	40	4	51,9
G-CSF	V221-5_1	40	3	64,7
G-CSF	V221-6_1	40	17	249,3
G-CSF	V221-7_1	40	7	99,9
G-CSF	V221-7_2	40	15	216,7
G-CSF	V221-11_1	40	5	63,4
G-CSF	V221-13_1	40	26	306,4
G-CSF	V221-15_1	40	5	84
G-CSF	V221-20_1	40	15	209,2
G-CSF	V222-19_1	40	10	116,3
G-CSF	V222-19_2	40	7	90,8
G-CSF	V222-20_1	40	5	82
G-CSF	V222-20_2	40	8	81,2
G-CSF	V222-21_1	40	14	179,2
G-CSF	V222-22_1	40	11	276,6
G-CSF	V222-22_2	40	20	260,8
G-CSF	V222-23_1	40	11	132,2
G-CSF	V222-24_1	40	15	162,1
G-CSF	V301-18_1	40	12	200,9
G-CSF	V301-18_2	40	8	128,5
G-CSF	V301-18_3	40	7	84,3
G-CSF	V301-19_1	40	8	99,1
G-CSF	V301-19_2	40	11	141,5
G-CSF	V301-20_1	40	9	128,1
G-CSF	V301-20_2	40	14	219,1
G-CSF	V302-15_1	40	8	114,3
G-CSF	V302-15_2	40	11	154,8
G-CSF	V302-15_3	40	8	110,3
G-CSF	V302-16_1	40	4	134,4
G-CSF	V302-16_2	40	2	44,8
G-CSF	V302-16_3	40	10	141,6
G-CSF	V302-16_4	40	9	140,2
G-CSF	V302-17_1	40	18	234,9
G-CSF	V302-17_2	40	9	128
G-CSF	V302-17_3	40	8	120,3
G-CSF	V303-15_1	40	5	55,5
G-CSF	V303-15_2	40	13	159,2
G-CSF	V303-15_3	40	8	139,9
G-CSF	V303-16_1	40	13	201,3
G-CSF	V303-16_2	40	10	137,7
G-CSF	V303-16_3	40	14	202,3
G-CSF	V303-17_1	40	14	170,4

basilar dendrite Sholl 50µm

Group	number	Radius(µm)	Intersections	Length(µm)
Control	V215-10_1	50	4	66,7
Control	V215-10_2	50	21	269,1
Control	V215-11_1	50	8	132,7
Control	V215-12_1	50	6	110,3
Control	V215-12_2	50	14	176,9
Control	V215-13_1	50	10	151,3
Control	V215-15_1	50	3	52,6
Control	V215-15_2	50	8	105,6
Control	V215-16_1	50	7	109,5
Control	V215-18_1	50	6	82,8
Control	V216-11_1	50	8	108,1
Control	V216-12_1	50	8	134,1
Control	V216-13_1	50	2	64,7
Control	V216-14_1	50	10	138,2
Control	V216-14_2	50	5	71,4
Control	V216-16_1	50	0	9,4
Control	V216-16_2	50	7	99,1
Control	V216-17_1	50	6	103,5
Control	V216-18_1	50	2	68,8
Control	V216-18_2	50	8	113
Control	V219-6_1	50	11	158,2
Control	V219-11_1	50	15	225,8
Control	V219-12_1	50	12	175,8
Control	V219-13_1	50	9	197,5
Control	V219-13_2	50	4	72,6
Control	V219-14_1	50	5	69,4
Control	V219-14_2	50	18	261,9
Control	V219-16_1	50	9	108,4
Control	V297-19_1	50	15	231,5
Control	V297-19_2	50	7	142,5
Control	V297-20_1	50	9	114,5
Control	V297-20_2	50	4	79,4
Control	V297-20_3	50	10	129,7
Control	V297-22_1	50	13	204,9
Control	V297-22_2	50	4	88,9
Control	V298-15_1	50	13	178,1
Control	V298-16_1	50	6	85,2
Control	V298-16_2	50	6	105,5
Control	V298-16_3	50	3	66,4
Control	V298-17_1	50	5	52,1
Control	V298-17_2	50	12	147
Control	V298-17_3	50	13	185,1
Control	V308-17_1	50	7	159,3
Control	V308-17_2	50	15	260
Control	V308-17_3	50	11	194,7
Control	V308-17_4	50	10	162,7
Control	V308-18_1	50	13	246,3
Control	V308-18_2	50	10	187,5
Control	V308-18_3	50	12	206,2
CIMT	V211-10_1	50	23	324,3
CIMT	V211-10_2	50	11	135,4
CIMT	V211-11_1	50	5	60,8
CIMT	V211-11_2	50	3	79,7

CIMT	V211-12_1	50	13	196,6
CIMT	V211-13_1	50	6	78,5
CIMT	V211-13_2	50	15	224,6
CIMT	V211-15_1	50	8	115,1
CIMT	V211-15_2	50	11	152,3
CIMT	V211-16_1	50	10	126,4
CIMT	V212-14_1	50	12	164
CIMT	V212-14_2	50	13	151,8
CIMT	V212-15_1	50	17	215,6
CIMT	V212-18_1	50	10	169,6
CIMT	V212-21_1	50	7	98
CIMT	V212-21_2	50	17	206,8
CIMT	V212-22_1	50	8	141,1
CIMT	V212-23_1	50	9	124,3
CIMT	V212-23_2	50	16	157
CIMT	V212-24_1	50	3	50,6
CIMT	V218-9_1	50	7	118,8
CIMT	V218-10_1	50	13	203,9
CIMT	V218-11_1	50	15	228,7
CIMT	V218-12_1	50	13	204,1
CIMT	V218-12_2	50	6	107,8
CIMT	V218-13_1	50	5	131,1
CIMT	V218-15_1	50	9	151,5
CIMT	V218-17_1	50	7	118,6
CIMT	V299-14_1	50	7	120,3
CIMT	V299-15_1	50	0	14
CIMT	V299-15_2	50	1	20,3
CIMT	V299-16_1	50	7	115,8
CIMT	V299-16_2	50	7	117,6
CIMT	V299-17_1	50	16	228,7
CIMT	V299-17_2	50	7	88,6
CIMT	V300-16_1	50	10	148,6
CIMT	V300-18_1	50	8	120,2
CIMT	V300-18_2	50	13	150,8
CIMT	V300-20_1	50	8	97,5
CIMT	V300-22_1	50	15	235,2
CIMT	V300-23_1	50	9	143,7
CIMT	V300-23_2	50	4	63
CIMT	V309-15_1	50	6	102,8
CIMT	V309-15_2	50	10	144,6
CIMT	V309-16_1	50	12	198,1
CIMT	V309-16_2	50	6	104,1
CIMT	V309-16_3	50	12	170,1
CIMT	V309-17_1	50	9	137,3
CIMT	V309-17_2	50	11	156
CIMT+G-CSF	V213-13_1	50	4	53,6
CIMT+G-CSF	V213-16_1	50	3	38,6
CIMT+G-CSF	V213-17_1	50	9	120,9
CIMT+G-CSF	V213-17_2	50	11	180,1
CIMT+G-CSF	V213-18_1	50	16	258,3
CIMT+G-CSF	V213-18_2	50	5	63,3
CIMT+G-CSF	V213-19_1	50	13	227,1
CIMT+G-CSF	V213-19_2	50	6	72,7
CIMT+G-CSF	V213-20_1	50	7	90,9
CIMT+G-CSF	V213-22_1	50	11	156
CIMT+G-CSF	V213-23_1	50	7	100,3

CIMT+G-CSF	V214-14_1	50	14	190,7
CIMT+G-CSF	V214-17_1	50	11	132,1
CIMT+G-CSF	V214-18_1	50	13	191,5
CIMT+G-CSF	V214-19_1	50	6	81,3
CIMT+G-CSF	V214-19_2	50	10	104,8
CIMT+G-CSF	V214-20_1	50	13	133,3
CIMT+G-CSF	V214-21_1	50	8	94,8
CIMT+G-CSF	V217-1_1	50	17	232,7
CIMT+G-CSF	V217-2_1	50	15	215
CIMT+G-CSF	V217-4_1	50	24	271,9
CIMT+G-CSF	V217-6_1	50	12	204
CIMT+G-CSF	V217-6_3	50	16	213
CIMT+G-CSF	V217-8_1	50	10	213,2
CIMT+G-CSF	V217-8_4	50	7	93,6
CIMT+G-CSF	V217-10_1	50	6	127,4
CIMT+G-CSF	V217-11_1	50	15	220,7
CIMT+G-CSF	V304-16_1	50	1	40,7
CIMT+G-CSF	V304-17_1	50	11	154,8
CIMT+G-CSF	V304-17_2	50	3	37,5
CIMT+G-CSF	V304-17_3	50	9	154,9
CIMT+G-CSF	V304-17_4	50	8	104,4
CIMT+G-CSF	V304-19_1	50	3	37,1
CIMT+G-CSF	V304-19_2	50	6	82,9
CIMT+G-CSF	V305-17_1	50	2	40,5
CIMT+G-CSF	V305-17_2	50	7	92,3
CIMT+G-CSF	V305-17_3	50	6	85,1
CIMT+G-CSF	V305-18_1	50	13	189,8
CIMT+G-CSF	V305-18_2	50	6	95,2
CIMT+G-CSF	V305-18_3	50	2	37,7
CIMT+G-CSF	V305-18_4	50	5	80,8
CIMT+G-CSF	V305-19_1	50	1	19,9
CIMT/G-CSF	V306-16_1	50	8	126,6
CIMT/G-CSF	V306-16_2	50	7	88,5
CIMT/G-CSF	V306-17_1	50	10	166,5
CIMT/G-CSF	V306-17_2	50	13	208,9
CIMT/G-CSF	V306-17_3	50	10	118,9
CIMT/G-CSF	V306-17_4	50	7	121,8
CIMT/G-CSF	V306-17_5	50	3	62,7
CIMT/G-CSF	V311-16_1	50	18	237,2
CIMT/G-CSF	V311-17_1	50	18	245,3
CIMT/G-CSF	V311-17_2	50	10	125,8
CIMT/G-CSF	V311-19_1	50	8	134,3
CIMT/G-CSF	V311-20_1	50	18	264,3
CIMT/G-CSF	V311-20_2	50	6	79,9
CIMT/G-CSF	V311-20_3	50	6	92,3
CIMT/G-CSF	V313-14_1	50	7	88,8
CIMT/G-CSF	V313-15_1	50	1	14
CIMT/G-CSF	V313-15_2	50	5	122
CIMT/G-CSF	V313-17_1	50	12	134,4
CIMT/G-CSF	V313-17_2	50	4	109,5
CIMT/G-CSF	V313-17_3	50	1	14,4
CIMT/G-CSF	V313-19_1	50	3	45,1
CIMT/G-CSF	V314-16_1	50	5	87
CIMT/G-CSF	V314-16_2	50	10	116,5
CIMT/G-CSF	V314-17_1	50	10	122,3
CIMT/G-CSF	V314-17_2	50	0	8,7

CIMT/G-CSF	V314-17_3	50	10	132,3
CIMT/G-CSF	V314-18_1	50	6	124,5
CIMT/G-CSF	V314-18_2	50	12	143
CIMT/G-CSF	V315-11_1	50	7	140,2
CIMT/G-CSF	V315-11_2	50	10	215
CIMT/G-CSF	V315-13_1	50	16	206,3
CIMT/G-CSF	V315-13_2	50	17	231,6
CIMT/G-CSF	V315-14_1	50	7	89,1
CIMT/G-CSF	V315-14_2	50	12	168
CIMT/G-CSF	V315-14_3	50	14	190,4
G-CSF	V220-9_1	50	15	189,5
G-CSF	V220-11_1	50	2	43,3
G-CSF	V220-12_1	50	7	98
G-CSF	V220-16_1	50	5	48,5
G-CSF	V220-16_2	50	6	82,8
G-CSF	V220-17_1	50	15	206,8
G-CSF	V220-18_1	50	7	84,5
G-CSF	V221-2_1	50	13	170,1
G-CSF	V221-4_1	50	2	41,9
G-CSF	V221-5_1	50	1	24,2
G-CSF	V221-6_1	50	16	207,6
G-CSF	V221-7_1	50	6	86,1
G-CSF	V221-7_2	50	18	202,5
G-CSF	V221-11_1	50	6	72,8
G-CSF	V221-13_1	50	29	352,9
G-CSF	V221-15_1	50	3	39,4
G-CSF	V221-20_1	50	13	202,8
G-CSF	V222-19_1	50	9	109,1
G-CSF	V222-19_2	50	4	66
G-CSF	V222-20_1	50	5	91
G-CSF	V222-20_2	50	13	160,4
G-CSF	V222-21_1	50	18	247,3
G-CSF	V222-22_1	50	9	192,3
G-CSF	V222-22_2	50	18	251,4
G-CSF	V222-23_1	50	13	149,6
G-CSF	V222-24_1	50	19	260,6
G-CSF	V301-18_1	50	7	143,9
G-CSF	V301-18_2	50	6	103,5
G-CSF	V301-18_3	50	5	59,6
G-CSF	V301-19_1	50	6	78,5
G-CSF	V301-19_2	50	9	125,3
G-CSF	V301-20_1	50	9	108,5
G-CSF	V301-20_2	50	10	190,7
G-CSF	V302-15_1	50	7	91,1
G-CSF	V302-15_2	50	9	138,5
G-CSF	V302-15_3	50	6	90,5
G-CSF	V302-16_1	50	2	41,8
G-CSF	V302-16_2	50	0	24,3
G-CSF	V302-16_3	50	8	114,6
G-CSF	V302-16_4	50	7	123,2
G-CSF	V302-17_1	50	18	245
G-CSF	V302-17_2	50	8	100,2
G-CSF	V302-17_3	50	9	101,2
G-CSF	V303-15_1	50	3	69,5
G-CSF	V303-15_2	50	10	195,9
G-CSF	V303-15_3	50	13	184,6

G-CSF	V303-16_1	50	15	216,8
G-CSF	V303-16_2	50	10	139,9
G-CSF	V303-16_3	50	11	160,1
G-CSF	V303-17_1	50	17	228,5

basilar dendrite Sholl 60µm

Group	number	Radius(µm)	Intersections	Length(µm)
Control	V215-10_1	60	3	68
Control	V215-10_2	60	19	272,8
Control	V215-11_1	60	6	87
Control	V215-12_1	60	7	101,2
Control	V215-12_2	60	16	189,7
Control	V215-13_1	60	6	109,4
Control	V215-15_1	60	3	34,4
Control	V215-15_2	60	7	90,2
Control	V215-16_1	60	6	90,3
Control	V215-18_1	60	4	56,8
Control	V216-11_1	60	6	96,9
Control	V216-12_1	60	4	70,1
Control	V216-13_1	60	2	22,1
Control	V216-14_1	60	6	86
Control	V216-14_2	60	5	53
Control	V216-16_2	60	7	84,4
Control	V216-17_1	60	5	88,1
Control	V216-18_1	60	2	22,6
Control	V216-18_2	60	5	77
Control	V219-6_1	60	16	230,5
Control	V219-11_1	60	13	245,6
Control	V219-12_1	60	10	140,9
Control	V219-13_1	60	5	134,2
Control	V219-13_2	60	2	45,1
Control	V219-14_1	60	4	68,8
Control	V219-14_2	60	17	286
Control	V219-16_1	60	8	120,3
Control	V297-19_1	60	16	215,3
Control	V297-19_2	60	4	71,4
Control	V297-20_1	60	6	98
Control	V297-20_2	60	4	44
Control	V297-20_3	60	9	123
Control	V297-22_1	60	13	191,3
Control	V297-22_2	60	2	25,8
Control	V298-15_1	60	15	197
Control	V298-16_1	60	4	60,3
Control	V298-16_2	60	4	65,4
Control	V298-16_3	60	3	34,9
Control	V298-17_1	60	2	46,1
Control	V298-17_2	60	13	168,9
Control	V298-17_3	60	12	156,8
Control	V308-17_1	60	5	80,3
Control	V308-17_2	60	13	228,3
Control	V308-17_3	60	13	143,7
Control	V308-17_4	60	8	116,7
Control	V308-18_1	60	9	156,4
Control	V308-18_2	60	7	120,2

Control	V308-18_3	60	10	170,6
CIMT	V211-10_1	60	25	339,2
CIMT	V211-10_2	60	7	136
CIMT	V211-11_1	60	4	50,3
CIMT	V211-11_2	60	3	39
CIMT	V211-12_1	60	7	140,9
CIMT	V211-13_1	60	6	73,6
CIMT	V211-13_2	60	14	221
CIMT	V211-15_1	60	6	92
CIMT	V211-15_2	60	5	101,3
CIMT	V211-16_1	60	10	125
CIMT	V212-14_1	60	13	167,7
CIMT	V212-14_2	60	13	158,4
CIMT	V212-15_1	60	15	199,3
CIMT	V212-18_1	60	8	149,8
CIMT	V212-21_1	60	7	103,8
CIMT	V212-21_2	60	14	196,2
CIMT	V212-22_1	60	7	97,1
CIMT	V212-23_1	60	9	115,5
CIMT	V212-23_2	60	30	345,3
CIMT	V212-24_1	60	3	35
CIMT	V218-9_1	60	5	82,2
CIMT	V218-10_1	60	12	204,8
CIMT	V218-11_1	60	15	197,9
CIMT	V218-12_1	60	13	209
CIMT	V218-12_2	60	8	109,3
CIMT	V218-13_1	60	4	69,9
CIMT	V218-15_1	60	9	127,5
CIMT	V218-17_1	60	8	87,8
CIMT	V299-14_1	60	5	87,5
CIMT	V299-15_2	60	0	9,6
CIMT	V299-16_1	60	6	75,6
CIMT	V299-16_2	60	8	81,5
CIMT	V299-17_1	60	14	167,5
CIMT	V299-17_2	60	5	97,3
CIMT	V300-16_1	60	10	113,8
CIMT	V300-18_1	60	5	98,9
CIMT	V300-18_2	60	15	169
CIMT	V300-20_1	60	8	86,3
CIMT	V300-22_1	60	11	164,6
CIMT	V300-23_1	60	10	159
CIMT	V300-23_2	60	4	75,3
CIMT	V309-15_1	60	4	70,8
CIMT	V309-15_2	60	5	86,3
CIMT	V309-16_1	60	13	236,5
CIMT	V309-16_2	60	3	56,8
CIMT	V309-16_3	60	11	143,8
CIMT	V309-17_1	60	7	115,1
CIMT	V309-17_2	60	13	133,1
CIMT+G-CSF	V213-13_1	60	4	43,9
CIMT+G-CSF	V213-16_1	60	1	37,8
CIMT+G-CSF	V213-17_1	60	8	96,5
CIMT+G-CSF	V213-17_2	60	10	133,9
CIMT+G-CSF	V213-18_1	60	15	216,6
CIMT+G-CSF	V213-18_2	60	5	60,6
CIMT+G-CSF	V213-19_1	60	10	206,9

CIMT+G-CSF	V213-19_2	60	5	63,5
CIMT+G-CSF	V213-20_1	60	6	76,9
CIMT+G-CSF	V213-22_1	60	11	139,4
CIMT+G-CSF	V213-23_1	60	4	60,5
CIMT+G-CSF	V214-14_1	60	16	194,1
CIMT+G-CSF	V214-17_1	60	13	152,6
CIMT+G-CSF	V214-18_1	60	19	224,6
CIMT+G-CSF	V214-19_1	60	5	54,7
CIMT+G-CSF	V214-19_2	60	9	115,7
CIMT+G-CSF	V214-20_1	60	9	184
CIMT+G-CSF	V214-21_1	60	7	97,8
CIMT+G-CSF	V217-1_1	60	15	226
CIMT+G-CSF	V217-2_1	60	13	190,2
CIMT+G-CSF	V217-4_1	60	27	384,7
CIMT+G-CSF	V217-6_1	60	6	159,6
CIMT+G-CSF	V217-6_3	60	13	186
CIMT+G-CSF	V217-8_1	60	5	104,1
CIMT+G-CSF	V217-8_4	60	11	144,8
CIMT+G-CSF	V217-10_1	60	2	59,1
CIMT+G-CSF	V217-11_1	60	17	223,7
CIMT+G-CSF	V304-16_1	60	1	12,1
CIMT+G-CSF	V304-17_1	60	9	115,5
CIMT+G-CSF	V304-17_2	60	3	37
CIMT+G-CSF	V304-17_3	60	6	98
CIMT+G-CSF	V304-17_4	60	8	114,2
CIMT+G-CSF	V304-19_1	60	3	35,4
CIMT+G-CSF	V304-19_2	60	5	84,1
CIMT+G-CSF	V305-17_1	60	0	12,7
CIMT+G-CSF	V305-17_2	60	5	76,7
CIMT+G-CSF	V305-17_3	60	4	65,2
CIMT+G-CSF	V305-18_1	60	11	161
CIMT+G-CSF	V305-18_2	60	4	67,3
CIMT+G-CSF	V305-18_3	60	2	23,5
CIMT+G-CSF	V305-18_4	60	4	59,8
CIMT+G-CSF	V305-19_1	60	0	2,9
CIMT/G-CSF	V306-16_1	60	8	103,4
CIMT/G-CSF	V306-16_2	60	2	51,9
CIMT/G-CSF	V306-17_1	60	9	118,4
CIMT/G-CSF	V306-17_2	60	13	163,3
CIMT/G-CSF	V306-17_3	60	11	160,9
CIMT/G-CSF	V306-17_4	60	3	69,2
CIMT/G-CSF	V306-17_5	60	2	28,3
CIMT/G-CSF	V311-16_1	60	19	272,3
CIMT/G-CSF	V311-17_1	60	16	230,6
CIMT/G-CSF	V311-17_2	60	9	116,2
CIMT/G-CSF	V311-19_1	60	8	123,1
CIMT/G-CSF	V311-20_1	60	17	262,1
CIMT/G-CSF	V311-20_2	60	5	67,5
CIMT/G-CSF	V311-20_3	60	5	63,7
CIMT/G-CSF	V313-14_1	60	7	82,4
CIMT/G-CSF	V313-15_1	60	0	8,1
CIMT/G-CSF	V313-15_2	60	4	65,5
CIMT/G-CSF	V313-17_1	60	11	138,5
CIMT/G-CSF	V313-17_2	60	4	56,8
CIMT/G-CSF	V313-17_3	60	1	12,8
CIMT/G-CSF	V313-19_1	60	2	26,5

CIMT/G-CSF	V314-16_1	60	3	45,2
CIMT/G-CSF	V314-16_2	60	9	192
CIMT/G-CSF	V314-17_1	60	9	120,9
CIMT/G-CSF	V314-17_3	60	8	117,9
CIMT/G-CSF	V314-18_1	60	6	72,1
CIMT/G-CSF	V314-18_2	60	8	155,9
CIMT/G-CSF	V315-11_1	60	4	60,6
CIMT/G-CSF	V315-11_2	60	8	106,9
CIMT/G-CSF	V315-13_1	60	13	256,5
CIMT/G-CSF	V315-13_2	60	19	238,5
CIMT/G-CSF	V315-14_1	60	6	94,7
CIMT/G-CSF	V315-14_2	60	9	145,6
CIMT/G-CSF	V315-14_3	60	14	178,7
G-CSF	V220-9_1	60	13	175,5
G-CSF	V220-11_1	60	2	27,7
G-CSF	V220-12_1	60	6	100,6
G-CSF	V220-16_1	60	14	83,8
G-CSF	V220-16_2	60	3	72,6
G-CSF	V220-17_1	60	12	173,9
G-CSF	V220-18_1	60	8	104,8
G-CSF	V221-2_1	60	10	148,5
G-CSF	V221-4_1	60	0	7,7
G-CSF	V221-5_1	60	0	1,5
G-CSF	V221-6_1	60	9	171,9
G-CSF	V221-7_1	60	6	75,9
G-CSF	V221-7_2	60	19	243,4
G-CSF	V221-11_1	60	4	67
G-CSF	V221-13_1	60	25	332,9
G-CSF	V221-15_1	60	1	27
G-CSF	V221-20_1	60	8	140,6
G-CSF	V222-19_1	60	14	148,5
G-CSF	V222-19_2	60	4	53,7
G-CSF	V222-20_1	60	3	66,5
G-CSF	V222-20_2	60	12	147,8
G-CSF	V222-21_1	60	19	255,7
G-CSF	V222-22_1	60	7	156,6
G-CSF	V222-22_2	60	14	227,4
G-CSF	V222-23_1	60	12	152,6
G-CSF	V222-24_1	60	19	243,8
G-CSF	V301-18_1	60	5	77,8
G-CSF	V301-18_2	60	6	76,7
G-CSF	V301-18_3	60	4	63,9
G-CSF	V301-19_1	60	7	70,6
G-CSF	V301-19_2	60	5	63,5
G-CSF	V301-20_1	60	7	95
G-CSF	V301-20_2	60	6	110,6
G-CSF	V302-15_1	60	4	63,5
G-CSF	V302-15_2	60	5	80,6
G-CSF	V302-15_3	60	5	71,3
G-CSF	V302-16_1	60	2	23,2
G-CSF	V302-16_3	60	3	82,9
G-CSF	V302-16_4	60	7	83
G-CSF	V302-17_1	60	17	254,5
G-CSF	V302-17_2	60	6	86,9
G-CSF	V302-17_3	60	5	79,4
G-CSF	V303-15_1	60	2	63,7

G-CSF	V303-15_2	60	7	129,9
G-CSF	V303-15_3	60	11	162,3
G-CSF	V303-16_1	60	20	223,2
G-CSF	V303-16_2	60	10	120,8
G-CSF	V303-16_3	60	10	176,1
G-CSF	V303-17_1	60	17	220,9

basilar dendrite Sholl 70µm

Group	number	Radius(µm)	Intersections	Length(µm)
Control	V215-10_1	70	0	7,2
Control	V215-10_2	70	15	202,7
Control	V215-11_1	70	6	75,8
Control	V215-12_1	70	8	90,9
Control	V215-12_2	70	10	155,4
Control	V215-13_1	70	6	86,2
Control	V215-15_1	70	1	22,6
Control	V215-15_2	70	7	80,2
Control	V215-16_1	70	4	67,6
Control	V215-18_1	70	4	48,3
Control	V216-11_1	70	4	75,3
Control	V216-12_1	70	3	38,8
Control	V216-13_1	70	2	26,3
Control	V216-14_1	70	4	52,2
Control	V216-14_2	70	2	35,7
Control	V216-16_2	70	6	81,6
Control	V216-17_1	70	1	38,5
Control	V216-18_1	70	2	22,1
Control	V216-18_2	70	5	55,9
Control	V219-6_1	70	18	299,5
Control	V219-11_1	70	13	197,7
Control	V219-12_1	70	8	127,2
Control	V219-13_1	70	5	108,7
Control	V219-13_2	70	1	22,9
Control	V219-14_1	70	2	44,4
Control	V219-14_2	70	15	242,7
Control	V219-16_1	70	7	103,3
Control	V297-19_1	70	12	201
Control	V297-19_2	70	2	41
Control	V297-20_1	70	5	66,5
Control	V297-20_2	70	2	38,7
Control	V297-20_3	70	9	133
Control	V297-22_1	70	12	174
Control	V297-22_2	70	0	14,7
Control	V298-15_1	70	16	182
Control	V298-16_1	70	2	30,5
Control	V298-16_2	70	5	50,7
Control	V298-16_3	70	1	26,9
Control	V298-17_1	70	2	22,8
Control	V298-17_2	70	13	150,4
Control	V298-17_3	70	12	145,4
Control	V308-17_1	70	4	59,5
Control	V308-17_2	70	13	184,4
Control	V308-17_3	70	9	163,7
Control	V308-17_4	70	5	82

Control	V308-18_1	70	7	132,6
Control	V308-18_2	70	6	91,8
Control	V308-18_3	70	9	132,6
CIMT	V211-10_1	70	21	295
CIMT	V211-10_2	70	6	73,6
CIMT	V211-11_1	70	4	50,5
CIMT	V211-11_2	70	1	33,2
CIMT	V211-12_1	70	8	118,3
CIMT	V211-13_1	70	7	95,6
CIMT	V211-13_2	70	11	158,3
CIMT	V211-15_1	70	4	60,2
CIMT	V211-15_2	70	5	67
CIMT	V211-16_1	70	10	113,8
CIMT	V212-14_1	70	13	179
CIMT	V212-14_2	70	12	148,8
CIMT	V212-15_1	70	14	183,3
CIMT	V212-18_1	70	8	118,1
CIMT	V212-21_1	70	7	89,4
CIMT	V212-21_2	70	16	164,2
CIMT	V212-22_1	70	7	79,7
CIMT	V212-23_1	70	6	111,6
CIMT	V212-23_2	70	29	375,1
CIMT	V212-24_1	70	7	58,6
CIMT	V218-9_1	70	4	75,8
CIMT	V218-10_1	70	13	205,5
CIMT	V218-11_1	70	16	178,3
CIMT	V218-12_1	70	13	155,9
CIMT	V218-12_2	70	7	102,3
CIMT	V218-13_1	70	3	55,8
CIMT	V218-15_1	70	6	81,4
CIMT	V218-17_1	70	6	90,8
CIMT	V299-14_1	70	4	49,6
CIMT	V299-16_1	70	2	56,2
CIMT	V299-16_2	70	9	138
CIMT	V299-17_1	70	10	143,5
CIMT	V299-17_2	70	6	53,1
CIMT	V300-16_1	70	11	149,2
CIMT	V300-18_1	70	5	58,7
CIMT	V300-18_2	70	9	175,7
CIMT	V300-20_1	70	7	86,7
CIMT	V300-22_1	70	10	164,5
CIMT	V300-23_1	70	8	124,9
CIMT	V300-23_2	70	3	56,6
CIMT	V309-15_1	70	4	49,4
CIMT	V309-15_2	70	5	69,7
CIMT	V309-16_1	70	12	186,2
CIMT	V309-16_2	70	1	15,6
CIMT	V309-16_3	70	9	126,2
CIMT	V309-17_1	70	6	85,9
CIMT	V309-17_2	70	12	141,6
CIMT+G-CSF	V213-13_1	70	4	47,3
CIMT+G-CSF	V213-16_1	70	1	12,2
CIMT+G-CSF	V213-17_1	70	7	94,7
CIMT+G-CSF	V213-17_2	70	9	122,8
CIMT+G-CSF	V213-18_1	70	11	176,9
CIMT+G-CSF	V213-18_2	70	3	56,7

CIMT+G-CSF	V213-19_1	70	9	122,3
CIMT+G-CSF	V213-19_2	70	3	52,9
CIMT+G-CSF	V213-20_1	70	4	50,3
CIMT+G-CSF	V213-22_1	70	9	121,5
CIMT+G-CSF	V213-23_1	70	5	61,4
CIMT+G-CSF	V214-14_1	70	16	202,2
CIMT+G-CSF	V214-17_1	70	9	171,4
CIMT+G-CSF	V214-18_1	70	22	295,5
CIMT+G-CSF	V214-19_1	70	4	57,5
CIMT+G-CSF	V214-19_2	70	8	99,8
CIMT+G-CSF	V214-20_1	70	8	106,6
CIMT+G-CSF	V214-21_1	70	8	103,2
CIMT+G-CSF	V217-1_1	70	13	198,7
CIMT+G-CSF	V217-2_1	70	11	169,3
CIMT+G-CSF	V217-4_1	70	28	365,4
CIMT+G-CSF	V217-6_1	70	5	104,3
CIMT+G-CSF	V217-6_3	70	10	146,8
CIMT+G-CSF	V217-8_1	70	2	54,1
CIMT+G-CSF	V217-8_4	70	13	187,1
CIMT+G-CSF	V217-10_1	70	1	23
CIMT+G-CSF	V217-11_1	70	15	197,4
CIMT+G-CSF	V304-16_1	70	1	11,1
CIMT+G-CSF	V304-17_1	70	8	102,3
CIMT+G-CSF	V304-17_2	70	2	33,7
CIMT+G-CSF	V304-17_3	70	5	62,7
CIMT+G-CSF	V304-17_4	70	13	159,3
CIMT+G-CSF	V304-19_1	70	3	40,9
CIMT+G-CSF	V304-19_2	70	6	66,2
CIMT+G-CSF	V305-17_2	70	3	44,8
CIMT+G-CSF	V305-17_3	70	3	47,8
CIMT+G-CSF	V305-18_1	70	9	137,5
CIMT+G-CSF	V305-18_2	70	2	43,3
CIMT+G-CSF	V305-18_3	70	0	6,7
CIMT+G-CSF	V305-18_4	70	3	45,8
CIMT/G-CSF	V306-16_1	70	7	100,5
CIMT/G-CSF	V306-16_2	70	2	22,7
CIMT/G-CSF	V306-17_1	70	9	124,8
CIMT/G-CSF	V306-17_2	70	12	157
CIMT/G-CSF	V306-17_3	70	6	132,9
CIMT/G-CSF	V306-17_4	70	2	34,5
CIMT/G-CSF	V306-17_5	70	2	28,1
CIMT/G-CSF	V311-16_1	70	18	238,5
CIMT/G-CSF	V311-17_1	70	13	181,6
CIMT/G-CSF	V311-17_2	70	5	72,5
CIMT/G-CSF	V311-19_1	70	8	111,4
CIMT/G-CSF	V311-20_1	70	16	235,8
CIMT/G-CSF	V311-20_2	70	5	61,6
CIMT/G-CSF	V311-20_3	70	5	55,7
CIMT/G-CSF	V313-14_1	70	7	101,6
CIMT/G-CSF	V313-15_2	70	4	39,2
CIMT/G-CSF	V313-17_1	70	12	141
CIMT/G-CSF	V313-17_2	70	4	38,1
CIMT/G-CSF	V313-17_3	70	1	13,1
CIMT/G-CSF	V313-19_1	70	1	23,9
CIMT/G-CSF	V314-16_1	70	2	27,5
CIMT/G-CSF	V314-16_2	70	7	126

CIMT/G-CSF	V314-17_1	70	6	88,1
CIMT/G-CSF	V314-17_3	70	5	85,4
CIMT/G-CSF	V314-18_1	70	5	59,8
CIMT/G-CSF	V314-18_2	70	7	103,5
CIMT/G-CSF	V315-11_1	70	5	81,5
CIMT/G-CSF	V315-11_2	70	4	79,5
CIMT/G-CSF	V315-13_1	70	9	183,6
CIMT/G-CSF	V315-13_2	70	16	231,1
CIMT/G-CSF	V315-14_1	70	4	65,2
CIMT/G-CSF	V315-14_2	70	6	78
CIMT/G-CSF	V315-14_3	70	15	171,9
G-CSF	V220-9_1	70	11	138,3
G-CSF	V220-11_1	70	0	7,8
G-CSF	V220-12_1	70	5	110,6
G-CSF	V220-16_1	70	13	213,2
G-CSF	V220-16_2	70	2	32,4
G-CSF	V220-17_1	70	13	213,2
G-CSF	V220-18_1	70	7	106,8
G-CSF	V221-2_1	70	10	136,7
G-CSF	V221-6_1	70	6	94,7
G-CSF	V221-7_1	70	4	64,3
G-CSF	V221-7_2	70	14	223,3
G-CSF	V221-11_1	70	3	37,7
G-CSF	V221-13_1	70	23	298,9
G-CSF	V221-15_1	70	1	10,7
G-CSF	V221-20_1	70	6	77,9
G-CSF	V222-19_1	70	13	193,2
G-CSF	V222-19_2	70	3	46,7
G-CSF	V222-20_1	70	19	174,5
G-CSF	V222-20_2	70	9	127,3
G-CSF	V222-21_1	70	16	220,3
G-CSF	V222-22_1	70	8	120,3
G-CSF	V222-22_2	70	9	147,9
G-CSF	V222-23_1	70	13	171,8
G-CSF	V222-24_1	70	13	209
G-CSF	V301-18_1	70	4	60,3
G-CSF	V301-18_2	70	5	67,4
G-CSF	V301-18_3	70	4	61,2
G-CSF	V301-19_1	70	6	78,4
G-CSF	V301-19_2	70	4	72,8
G-CSF	V301-20_1	70	7	89,4
G-CSF	V301-20_2	70	5	70,3
G-CSF	V302-15_1	70	3	41,2
G-CSF	V302-15_2	70	5	64,3
G-CSF	V302-15_3	70	5	54,9
G-CSF	V302-16_1	70	1	21,7
G-CSF	V302-16_3	70	3	33,9
G-CSF	V302-16_4	70	7	79,4
G-CSF	V302-17_1	70	16	252,8
G-CSF	V302-17_2	70	4	56,5
G-CSF	V302-17_3	70	4	48,7
G-CSF	V303-15_1	70	1	15,3
G-CSF	V303-15_2	70	5	83
G-CSF	V303-15_3	70	9	121,3
G-CSF	V303-16_1	70	15	268,8
G-CSF	V303-16_2	70	9	107,1

G-CSF	V303-16_3	70	7	118,5
G-CSF	V303-17_1	70	16	219,3

basilar dendrite Sholl 80µm

Group	number	Radius(µm)	Intersections	Length(µm)
Control	V215-10_2	80	15	185,4
Control	V215-11_1	80	4	63,8
Control	V215-12_1	80	7	98,3
Control	V215-12_2	80	9	116,4
Control	V215-13_1	80	6	78,8
Control	V215-15_1	80	1	10,9
Control	V215-15_2	80	6	93,9
Control	V215-16_1	80	4	44,8
Control	V215-18_1	80	2	32,2
Control	V216-11_1	80	3	33,1
Control	V216-12_1	80	2	33,9
Control	V216-13_1	80	2	20,9
Control	V216-14_1	80	3	39,2
Control	V216-14_2	80	1	15,4
Control	V216-16_2	80	8	74,2
Control	V216-17_1	80	1	12,6
Control	V216-18_1	80	1	17
Control	V216-18_2	80	5	68
Control	V219-6_1	80	22	317,6
Control	V219-11_1	80	10	183,6
Control	V219-12_1	80	8	105,7
Control	V219-13_1	80	2	69,4
Control	V219-13_2	80	1	16,3
Control	V219-14_1	80	1	31,5
Control	V219-14_2	80	15	208,6
Control	V219-16_1	80	6	97,6
Control	V297-19_1	80	7	123,5
Control	V297-19_2	80	3	44,3
Control	V297-20_1	80	1	25,8
Control	V297-20_2	80	2	25
Control	V297-20_3	80	8	99,2
Control	V297-22_1	80	12	144,3
Control	V298-15_1	80	13	201,4
Control	V298-16_1	80	1	24,4
Control	V298-16_2	80	6	70,2
Control	V298-16_3	80	1	10,8
Control	V298-17_1	80	2	22,9
Control	V298-17_2	80	14	164,6
Control	V298-17_3	80	12	160,6
Control	V308-17_1	80	3	46,8
Control	V308-17_2	80	15	198,2
Control	V308-17_3	80	7	127,6
Control	V308-17_4	80	7	84,8
Control	V308-18_1	80	3	68,5
Control	V308-18_2	80	5	86,4
Control	V308-18_3	80	9	133,3
CIMT	V211-10_1	80	19	275
CIMT	V211-10_2	80	8	79,3
CIMT	V211-11_1	80	3	45

CIMT	V211-11_2	80	1	11,7
CIMT	V211-12_1	80	6	85,2
CIMT	V211-13_1	80	7	68,2
CIMT	V211-13_2	80	8	114,5
CIMT	V211-15_1	80	2	32,5
CIMT	V211-15_2	80	5	78,4
CIMT	V211-16_1	80	9	119,1
CIMT	V212-14_1	80	11	147,2
CIMT	V212-14_2	80	11	154,1
CIMT	V212-15_1	80	16	180,9
CIMT	V212-18_1	80	4	81
CIMT	V212-21_1	80	6	81,4
CIMT	V212-21_2	80	16	244,6
CIMT	V212-22_1	80	5	76,5
CIMT	V212-23_1	80	6	72,3
CIMT	V212-23_2	80	25	347,8
CIMT	V212-24_1	80	6	157,4
CIMT	V218-9_1	80	3	52,4
CIMT	V218-10_1	80	10	154,5
CIMT	V218-11_1	80	14	167,3
CIMT	V218-12_1	80	10	173,5
CIMT	V218-12_2	80	6	106
CIMT	V218-13_1	80	0	9,7
CIMT	V218-15_1	80	3	74,4
CIMT	V218-17_1	80	6	73
CIMT	V299-14_1	80	3	41,3
CIMT	V299-16_1	80	1	14,3
CIMT	V299-16_2	80	7	105,4
CIMT	V299-17_1	80	9	127,7
CIMT	V299-17_2	80	5	89,5
CIMT	V300-16_1	80	9	130,8
CIMT	V300-18_1	80	5	57,9
CIMT	V300-18_2	80	8	99,4
CIMT	V300-20_1	80	6	73,5
CIMT	V300-22_1	80	8	100,8
CIMT	V300-23_1	80	3	53,5
CIMT	V300-23_2	80	3	35,1
CIMT	V309-15_1	80	3	43
CIMT	V309-15_2	80	3	47,8
CIMT	V309-16_1	80	8	155,7
CIMT	V309-16_2	80	0	4
CIMT	V309-16_3	80	8	127,3
CIMT	V309-17_1	80	4	51,4
CIMT	V309-17_2	80	11	141
CIMT+G-CSF	V213-13_1	80	3	36,2
CIMT+G-CSF	V213-16_1	80	1	10,2
CIMT+G-CSF	V213-17_1	80	5	82,1
CIMT+G-CSF	V213-17_2	80	7	92,8
CIMT+G-CSF	V213-18_1	80	7	107,9
CIMT+G-CSF	V213-18_2	80	3	37,2
CIMT+G-CSF	V213-19_1	80	9	125,9
CIMT+G-CSF	V213-19_2	80	3	38,5
CIMT+G-CSF	V213-20_1	80	4	53
CIMT+G-CSF	V213-22_1	80	8	93,9
CIMT+G-CSF	V213-23_1	80	4	53,1
CIMT+G-CSF	V214-14_1	80	13	216,6

CIMT+G-CSF	V214-17_1	80	7	110,9
CIMT+G-CSF	V214-18_1	80	22	286,8
CIMT+G-CSF	V214-19_1	80	4	53,2
CIMT+G-CSF	V214-19_2	80	8	104,7
CIMT+G-CSF	V214-20_1	80	9	100,4
CIMT+G-CSF	V214-21_1	80	8	97,9
CIMT+G-CSF	V217-1_1	80	14	174,9
CIMT+G-CSF	V217-2_1	80	7	124
CIMT+G-CSF	V217-4_1	80	26	357,4
CIMT+G-CSF	V217-6_1	80	3	60,4
CIMT+G-CSF	V217-6_3	80	9	127,7
CIMT+G-CSF	V217-8_1	80	0	8
CIMT+G-CSF	V217-8_4	80	9	196,5
CIMT+G-CSF	V217-10_1	80	1	12,3
CIMT+G-CSF	V217-11_1	80	12	181,2
CIMT+G-CSF	V304-16_1	80	1	11,6
CIMT+G-CSF	V304-17_1	80	5	129,4
CIMT+G-CSF	V304-17_2	80	2	21,6
CIMT+G-CSF	V304-17_3	80	4	51,2
CIMT+G-CSF	V304-17_4	80	11	158,9
CIMT+G-CSF	V304-19_1	80	2	32,6
CIMT+G-CSF	V304-19_2	80	4	47,8
CIMT+G-CSF	V305-17_2	80	0	19,4
CIMT+G-CSF	V305-17_3	80	1	30,7
CIMT+G-CSF	V305-18_1	80	7	96
CIMT+G-CSF	V305-18_2	80	1	15,7
CIMT+G-CSF	V305-18_4	80	2	30,6
CIMT/G-CSF	V306-16_1	80	6	103,7
CIMT/G-CSF	V306-16_2	80	1	12,2
CIMT/G-CSF	V306-17_1	80	5	98,3
CIMT/G-CSF	V306-17_2	80	9	145,7
CIMT/G-CSF	V306-17_3	80	3	56,6
CIMT/G-CSF	V306-17_4	80	2	29,2
CIMT/G-CSF	V306-17_5	80	1	23,6
CIMT/G-CSF	V311-16_1	80	16	210,9
CIMT/G-CSF	V311-17_1	80	13	160,9
CIMT/G-CSF	V311-17_2	80	4	66,7
CIMT/G-CSF	V311-19_1	80	8	107,2
CIMT/G-CSF	V311-20_1	80	17	208,2
CIMT/G-CSF	V311-20_2	80	5	63,7
CIMT/G-CSF	V311-20_3	80	5	57,1
CIMT/G-CSF	V313-14_1	80	4	65,1
CIMT/G-CSF	V313-15_2	80	1	29
CIMT/G-CSF	V313-17_1	80	10	117,3
CIMT/G-CSF	V313-17_2	80	1	37,5
CIMT/G-CSF	V313-17_3	80	1	11,8
CIMT/G-CSF	V313-19_1	80	1	10,7
CIMT/G-CSF	V314-16_1	80	0	5,9
CIMT/G-CSF	V314-16_2	80	9	120
CIMT/G-CSF	V314-17_1	80	6	73
CIMT/G-CSF	V314-17_3	80	1	46,2
CIMT/G-CSF	V314-18_1	80	3	51,1
CIMT/G-CSF	V314-18_2	80	5	88
CIMT/G-CSF	V315-11_1	80	4	83,2
CIMT/G-CSF	V315-11_2	80	2	33,8
CIMT/G-CSF	V315-13_1	80	5	116,8

CIMT/G-CSF	V315-13_2	80	18	205,5
CIMT/G-CSF	V315-14_1	80	4	45,3
CIMT/G-CSF	V315-14_2	80	6	68,5
CIMT/G-CSF	V315-14_3	80	17	190,3
G-CSF	V220-9_1	80	9	134,6
G-CSF	V220-12_1	80	4	64,8
G-CSF	V220-16_1	80	10	187,9
G-CSF	V220-16_2	80	1	11
G-CSF	V220-17_1	80	10	190,7
G-CSF	V220-18_1	80	6	83,6
G-CSF	V221-2_1	80	8	140,1
G-CSF	V221-6_1	80	7	78,1
G-CSF	V221-7_1	80	4	46
G-CSF	V221-7_2	80	13	158
G-CSF	V221-11_1	80	2	35,1
G-CSF	V221-13_1	80	23	322,8
G-CSF	V221-15_1	80	1	10,2
G-CSF	V221-20_1	80	5	60,7
G-CSF	V222-19_1	80	14	184,4
G-CSF	V222-19_2	80	2	29,2
G-CSF	V222-20_1	80	13	214,9
G-CSF	V222-20_2	80	7	97,1
G-CSF	V222-21_1	80	12	179,7
G-CSF	V222-22_1	80	9	148,4
G-CSF	V222-22_2	80	7	93,3
G-CSF	V222-23_1	80	12	162,9
G-CSF	V222-24_1	80	11	162,1
G-CSF	V301-18_1	80	2	30,6
G-CSF	V301-18_2	80	4	45,4
G-CSF	V301-18_3	80	1	32,7
G-CSF	V301-19_1	80	4	86,6
G-CSF	V301-19_2	80	4	45,3
G-CSF	V301-20_1	80	4	71
G-CSF	V301-20_2	80	4	59,9
G-CSF	V302-15_1	80	2	29,3
G-CSF	V302-15_2	80	4	62,7
G-CSF	V302-15_3	80	4	49,6
G-CSF	V302-16_1	80	1	10,4
G-CSF	V302-16_3	80	2	31,7
G-CSF	V302-16_4	80	6	66,9
G-CSF	V302-17_1	80	17	213,6
G-CSF	V302-17_2	80	3	47,2
G-CSF	V302-17_3	80	2	41,1
G-CSF	V303-15_1	80	0	5,9
G-CSF	V303-15_2	80	5	72,5
G-CSF	V303-15_3	80	8	105,6
G-CSF	V303-16_1	80	14	178,2
G-CSF	V303-16_2	80	8	94,7
G-CSF	V303-16_3	80	3	91,7
G-CSF	V303-17_1	80	15	199,6

basilar dendrite Sholl 90μm

Group	number	Radius(μm)	Intersections	Length(μm)
Control	V215-10_2	90	14	179,5
Control	V215-11_1	90	2	45,2
Control	V215-12_1	90	4	77,7
Control	V215-12_2	90	8	98,6
Control	V215-13_1	90	5	77,3
Control	V215-15_1	90	0	3,7
Control	V215-15_2	90	5	77,6
Control	V215-16_1	90	4	44,2
Control	V215-18_1	90	2	26
Control	V216-11_1	90	3	33,4
Control	V216-12_1	90	0	7,6
Control	V216-13_1	90	2	21
Control	V216-14_1	90	1	22,2
Control	V216-14_2	90	0	2,3
Control	V216-16_2	90	6	68,3
Control	V216-17_1	90	1	12,5
Control	V216-18_1	90	0	7
Control	V216-18_2	90	4	60,8
Control	V219-6_1	90	22	324,1
Control	V219-11_1	90	11	153,7
Control	V219-12_1	90	9	111,5
Control	V219-13_1	90	1	34,2
Control	V219-13_2	90	0	6,6
Control	V219-14_1	90	0	5,8
Control	V219-14_2	90	6	115,6
Control	V219-16_1	90	4	77,9
Control	V297-19_1	90	7	84,3
Control	V297-19_2	90	1	16,1
Control	V297-20_1	90	0	3,8
Control	V297-20_2	90	2	25,7
Control	V297-20_3	90	7	150,9
Control	V297-22_1	90	11	137,3
Control	V298-15_1	90	16	196
Control	V298-16_1	90	0	1,7
Control	V298-16_2	90	4	60,9
Control	V298-16_3	90	0	8,2
Control	V298-17_1	90	2	23,1
Control	V298-17_2	90	12	159,7
Control	V298-17_3	90	11	136,4
Control	V308-17_1	90	3	41,6
Control	V308-17_2	90	10	162,6
Control	V308-17_3	90	7	88,2
Control	V308-17_4	90	6	118,7
Control	V308-18_1	90	1	28,3
Control	V308-18_2	90	5	65,2
Control	V308-18_3	90	9	112,5
CIMT	V211-10_1	90	16	231,6
CIMT	V211-10_2	90	9	101,6
CIMT	V211-11_1	90	2	37,6
CIMT	V211-11_2	90	1	10,7
CIMT	V211-12_1	90	3	73,3
CIMT	V211-13_1	90	3	51,7
CIMT	V211-13_2	90	6	84

CIMT	V211-15_1	90	0	7,8
CIMT	V211-15_2	90	2	48,4
CIMT	V211-16_1	90	6	127,4
CIMT	V212-14_1	90	9	120,5
CIMT	V212-14_2	90	10	149,7
CIMT	V212-15_1	90	12	146,6
CIMT	V212-18_1	90	3	45,1
CIMT	V212-21_1	90	5	61,1
CIMT	V212-21_2	90	17	207,4
CIMT	V212-22_1	90	5	109,8
CIMT	V212-23_1	90	4	74,6
CIMT	V212-23_2	90	22	351,8
CIMT	V212-24_1	90	7	130,9
CIMT	V218-9_1	90	1	43
CIMT	V218-10_1	90	11	133,1
CIMT	V218-11_1	90	11	161,1
CIMT	V218-12_1	90	7	104,9
CIMT	V218-12_2	90	4	83,7
CIMT	V218-15_1	90	1	40,5
CIMT	V218-17_1	90	6	70,2
CIMT	V299-14_1	90	2	35,2
CIMT	V299-16_1	90	0	8,5
CIMT	V299-16_2	90	7	142,1
CIMT	V299-17_1	90	7	108,1
CIMT	V299-17_2	90	4	51,1
CIMT	V300-16_1	90	9	96,3
CIMT	V300-18_1	90	2	42,4
CIMT	V300-18_2	90	5	74,1
CIMT	V300-20_1	90	4	50,3
CIMT	V300-22_1	90	8	105,3
CIMT	V300-23_1	90	1	24,1
CIMT	V300-23_2	90	1	18,1
CIMT	V309-15_1	90	1	59,2
CIMT	V309-15_2	90	3	34
CIMT	V309-16_1	90	4	77,4
CIMT	V309-16_3	90	7	90,3
CIMT	V309-17_1	90	7	67,4
CIMT	V309-17_2	90	10	118,4
CIMT+G-CSF	V213-13_1	90	3	31,8
CIMT+G-CSF	V213-16_1	90	0	7,9
CIMT+G-CSF	V213-17_1	90	5	73,1
CIMT+G-CSF	V213-17_2	90	6	72,1
CIMT+G-CSF	V213-18_1	90	6	84,2
CIMT+G-CSF	V213-18_2	90	4	38,3
CIMT+G-CSF	V213-19_1	90	8	107,3
CIMT+G-CSF	V213-19_2	90	2	25,5
CIMT+G-CSF	V213-20_1	90	2	29
CIMT+G-CSF	V213-22_1	90	8	87,2
CIMT+G-CSF	V213-23_1	90	1	33,4
CIMT+G-CSF	V214-14_1	90	12	177
CIMT+G-CSF	V214-17_1	90	6	79,5
CIMT+G-CSF	V214-18_1	90	19	310,7
CIMT+G-CSF	V214-19_1	90	4	47,2
CIMT+G-CSF	V214-19_2	90	7	99
CIMT+G-CSF	V214-20_1	90	9	102
CIMT+G-CSF	V214-21_1	90	6	90,2

CIMT+G-CSF	V217-1_1	90	15	211,7
CIMT+G-CSF	V217-2_1	90	5	87,4
CIMT+G-CSF	V217-4_1	90	25	372,4
CIMT+G-CSF	V217-6_1	90	3	43,7
CIMT+G-CSF	V217-6_3	90	6	126,4
CIMT+G-CSF	V217-8_4	90	9	117,5
CIMT+G-CSF	V217-10_1	90	1	12,4
CIMT+G-CSF	V217-11_1	90	10	134,6
CIMT+G-CSF	V304-16_1	90	0	4,5
CIMT+G-CSF	V304-17_1	90	3	53,3
CIMT+G-CSF	V304-17_2	90	3	29,9
CIMT+G-CSF	V304-17_3	90	4	41,7
CIMT+G-CSF	V304-17_4	90	6	128,8
CIMT+G-CSF	V304-19_1	90	1	25,1
CIMT+G-CSF	V304-19_2	90	4	44,5
CIMT+G-CSF	V305-17_3	90	0	4,3
CIMT+G-CSF	V305-18_1	90	4	54,7
CIMT+G-CSF	V305-18_2	90	1	10,2
CIMT+G-CSF	V305-18_4	90	1	18,3
CIMT/G-CSF	V306-16_1	90	4	59,5
CIMT/G-CSF	V306-16_2	90	1	11,3
CIMT/G-CSF	V306-17_1	90	5	68,7
CIMT/G-CSF	V306-17_2	90	9	124,9
CIMT/G-CSF	V306-17_3	90	3	33,4
CIMT/G-CSF	V306-17_4	90	2	21,1
CIMT/G-CSF	V306-17_5	90	0	0,4
CIMT/G-CSF	V311-16_1	90	13	190,3
CIMT/G-CSF	V311-17_1	90	7	134,7
CIMT/G-CSF	V311-17_2	90	4	49,9
CIMT/G-CSF	V311-19_1	90	7	91,4
CIMT/G-CSF	V311-20_1	90	13	218
CIMT/G-CSF	V311-20_2	90	4	45,1
CIMT/G-CSF	V311-20_3	90	5	58,1
CIMT/G-CSF	V313-14_1	90	5	62,2
CIMT/G-CSF	V313-15_2	90	1	23,5
CIMT/G-CSF	V313-17_1	90	8	108,7
CIMT/G-CSF	V313-17_2	90	1	11,1
CIMT/G-CSF	V313-17_3	90	0	0
CIMT/G-CSF	V313-19_1	90	1	10,8
CIMT/G-CSF	V314-16_2	90	5	118,1
CIMT/G-CSF	V314-17_1	90	2	37,4
CIMT/G-CSF	V314-17_3	90	1	16,4
CIMT/G-CSF	V314-18_1	90	1	21,9
CIMT/G-CSF	V314-18_2	90	5	72,5
CIMT/G-CSF	V315-11_1	90	4	50,5
CIMT/G-CSF	V315-11_2	90	2	22
CIMT/G-CSF	V315-13_1	90	2	56,1
CIMT/G-CSF	V315-13_2	90	15	203
CIMT/G-CSF	V315-14_1	90	5	66,3
CIMT/G-CSF	V315-14_2	90	5	62,8
CIMT/G-CSF	V315-14_3	90	16	257,7
G-CSF	V220-9_1	90	8	87,6
G-CSF	V220-12_1	90	4	47,1
G-CSF	V220-16_1	90	15	186,2
G-CSF	V220-16_2	90	1	13,2
G-CSF	V220-17_1	90	9	141

G-CSF	V220-18_1	90	3	62,4
G-CSF	V221-2_1	90	6	75,1
G-CSF	V221-6_1	90	5	74,8
G-CSF	V221-7_1	90	5	57
G-CSF	V221-7_2	90	10	134,2
G-CSF	V221-11_1	90	2	20,8
G-CSF	V221-13_1	90	19	253,6
G-CSF	V221-15_1	90	1	10,1
G-CSF	V221-20_1	90	3	37,2
G-CSF	V222-19_1	90	13	158,5
G-CSF	V222-19_2	90	1	22,3
G-CSF	V222-20_1	90	16	240,9
G-CSF	V222-20_2	90	7	80,1
G-CSF	V222-21_1	90	11	134,7
G-CSF	V222-22_1	90	6	98,1
G-CSF	V222-22_2	90	7	87,9
G-CSF	V222-23_1	90	11	150,2
G-CSF	V222-24_1	90	8	117,3
G-CSF	V301-18_1	90	1	23,2
G-CSF	V301-18_2	90	4	52,1
G-CSF	V301-18_3	90	1	13,2
G-CSF	V301-19_1	90	4	47,7
G-CSF	V301-19_2	90	3	46,4
G-CSF	V301-20_1	90	4	45,9
G-CSF	V301-20_2	90	5	95
G-CSF	V302-15_1	90	1	12,5
G-CSF	V302-15_2	90	2	35,8
G-CSF	V302-15_3	90	2	39,2
G-CSF	V302-16_1	90	1	10,2
G-CSF	V302-16_3	90	0	15,5
G-CSF	V302-16_4	90	7	71,4
G-CSF	V302-17_1	90	15	234
G-CSF	V302-17_2	90	2	24,7
G-CSF	V302-17_3	90	1	14,9
G-CSF	V303-15_2	90	2	47,8
G-CSF	V303-15_3	90	7	92,5
G-CSF	V303-16_1	90	13	179,9
G-CSF	V303-16_2	90	4	70,7
G-CSF	V303-16_3	90	2	30,8
G-CSF	V303-17_1	90	14	184,9

basilar dendrite Sholl 100µm

Group	number	Radius(µm)	Intersections	Length(µm)
Control	V215-10_2	100	14	167,6
Control	V215-11_1	100	0	7,9
Control	V215-12_1	100	3	36,6
Control	V215-12_2	100	7	88,2
Control	V215-13_1	100	3	78
Control	V215-15_2	100	2	77,7
Control	V215-16_1	100	4	49,7
Control	V215-18_1	100	1	23,9
Control	V216-11_1	100	2	28,4
Control	V216-13_1	100	0	2,8
Control	V216-14_1	100	1	10,1

Control	V216-16_2	100	6	66,4
Control	V216-17_1	100	1	17,7
Control	V216-18_2	100	4	50
Control	V219-6_1	100	23	297,5
Control	V219-11_1	100	8	130,5
Control	V219-12_1	100	7	115,2
Control	V219-13_1	100	3	22,3
Control	V219-14_2	100	4	56
Control	V219-16_1	100	2	40,2
Control	V297-19_1	100	6	98,4
Control	V297-19_2	100	0	0,6
Control	V297-20_2	100	1	14,8
Control	V297-20_3	100	7	89,7
Control	V297-22_1	100	11	132,9
Control	V298-15_1	100	11	190,1
Control	V298-16_2	100	3	42,4
Control	V298-17_1	100	1	11,7
Control	V298-17_2	100	8	128,7
Control	V298-17_3	100	11	146,3
Control	V308-17_1	100	3	37,1
Control	V308-17_2	100	8	129,4
Control	V308-17_3	100	8	87,9
Control	V308-17_4	100	5	98,1
Control	V308-18_1	100	0	6,5
Control	V308-18_2	100	1	58,4
Control	V308-18_3	100	8	115,6
CIMT	V211-10_1	100	16	190
CIMT	V211-10_2	100	10	110,9
CIMT	V211-11_1	100	2	26,5
CIMT	V211-11_2	100	1	10,5
CIMT	V211-12_1	100	1	27,2
CIMT	V211-13_1	100	3	32,6
CIMT	V211-13_2	100	5	59,1
CIMT	V211-15_2	100	1	19,2
CIMT	V211-16_1	100	4	57,4
CIMT	V212-14_1	100	7	101,5
CIMT	V212-14_2	100	10	123,4
CIMT	V212-15_1	100	11	137,7
CIMT	V212-18_1	100	3	35,7
CIMT	V212-21_1	100	5	52,4
CIMT	V212-21_2	100	18	238,4
CIMT	V212-22_1	100	4	60,9
CIMT	V212-23_1	100	3	42,4
CIMT	V212-23_2	100	24	368,2
CIMT	V212-24_1	100	4	62,8
CIMT	V218-9_1	100	1	10
CIMT	V218-10_1	100	7	155,7
CIMT	V218-11_1	100	11	144,1
CIMT	V218-12_1	100	11	110,1
CIMT	V218-12_2	100	2	34,2
CIMT	V218-15_1	100	1	10,1
CIMT	V218-17_1	100	5	63,9
CIMT	V299-14_1	100	2	25,2
CIMT	V299-16_2	100	10	154,9
CIMT	V299-17_1	100	7	101,4
CIMT	V299-17_2	100	2	37,2

CIMT	V300-16_1	100	8	100,9
CIMT	V300-18_1	100	2	21
CIMT	V300-18_2	100	7	63,9
CIMT	V300-20_1	100	4	47,1
CIMT	V300-22_1	100	7	98,5
CIMT	V300-23_1	100	1	10,8
CIMT	V300-23_2	100	1	14,8
CIMT	V309-15_1	100	1	11,3
CIMT	V309-15_2	100	3	34
CIMT	V309-16_1	100	0	24,1
CIMT	V309-16_3	100	7	98
CIMT	V309-17_1	100	3	138,7
CIMT	V309-17_2	100	10	112,3
CIMT+G-CSF	V213-13_1	100	3	32,2
CIMT+G-CSF	V213-17_1	100	3	53,8
CIMT+G-CSF	V213-17_2	100	6	68,7
CIMT+G-CSF	V213-18_1	100	6	134,1
CIMT+G-CSF	V213-18_2	100	4	47,6
CIMT+G-CSF	V213-19_1	100	5	84,1
CIMT+G-CSF	V213-19_2	100	1	21,4
CIMT+G-CSF	V213-20_1	100	0	9
CIMT+G-CSF	V213-22_1	100	8	88,9
CIMT+G-CSF	V213-23_1	100	1	11,5
CIMT+G-CSF	V214-14_1	100	8	125,8
CIMT+G-CSF	V214-17_1	100	6	81,5
CIMT+G-CSF	V214-18_1	100	14	232
CIMT+G-CSF	V214-19_1	100	1	24,1
CIMT+G-CSF	V214-19_2	100	6	75,5
CIMT+G-CSF	V214-20_1	100	8	92,2
CIMT+G-CSF	V214-21_1	100	6	70,2
CIMT+G-CSF	V217-1_1	100	13	169,1
CIMT+G-CSF	V217-2_1	100	5	68,8
CIMT+G-CSF	V217-4_1	100	23	322,3
CIMT+G-CSF	V217-6_1	100	1	25,4
CIMT+G-CSF	V217-6_3	100	3	89,2
CIMT+G-CSF	V217-8_4	100	8	106,2
CIMT+G-CSF	V217-10_1	100	1	14,6
CIMT+G-CSF	V217-11_1	100	11	143,5
CIMT+G-CSF	V304-17_1	100	1	12,2
CIMT+G-CSF	V304-17_2	100	2	26,8
CIMT+G-CSF	V304-17_3	100	3	43,8
CIMT+G-CSF	V304-17_4	100	5	68,9
CIMT+G-CSF	V304-19_1	100	1	10,8
CIMT+G-CSF	V304-19_2	100	3	36,5
CIMT+G-CSF	V305-18_1	100	3	45,5
CIMT+G-CSF	V305-18_2	100	1	10,3
CIMT+G-CSF	V305-18_4	100	1	12,5
CIMT/G-CSF	V306-16_1	100	1	24,4
CIMT/G-CSF	V306-16_2	100	1	13,6
CIMT/G-CSF	V306-17_1	100	5	63,1
CIMT/G-CSF	V306-17_2	100	5	69,7
CIMT/G-CSF	V306-17_3	100	3	34,6
CIMT/G-CSF	V306-17_4	100	1	14,1
CIMT/G-CSF	V311-16_1	100	13	157,2
CIMT/G-CSF	V311-17_1	100	6	86,1
CIMT/G-CSF	V311-17_2	100	4	51,7

CIMT/G-CSF	V311-19_1	100	6	98,4
CIMT/G-CSF	V311-20_1	100	11	148,9
CIMT/G-CSF	V311-20_2	100	4	41,7
CIMT/G-CSF	V311-20_3	100	4	56,6
CIMT/G-CSF	V313-14_1	100	5	73
CIMT/G-CSF	V313-15_2	100	0	9,7
CIMT/G-CSF	V313-17_1	100	8	108,3
CIMT/G-CSF	V313-17_2	100	1	15,1
CIMT/G-CSF	V313-19_1	100	0	2,6
CIMT/G-CSF	V314-16_2	100	5	95,8
CIMT/G-CSF	V314-17_1	100	2	23,3
CIMT/G-CSF	V314-17_3	100	1	20
CIMT/G-CSF	V314-18_1	100	1	13,9
CIMT/G-CSF	V314-18_2	100	6	86,1
CIMT/G-CSF	V315-11_1	100	3	42,9
CIMT/G-CSF	V315-11_2	100	0	18
CIMT/G-CSF	V315-13_1	100	1	19,2
CIMT/G-CSF	V315-13_2	100	12	158
CIMT/G-CSF	V315-14_1	100	3	50,6
CIMT/G-CSF	V315-14_2	100	3	48,8
CIMT/G-CSF	V315-14_3	100	14	213,7
G-CSF	V220-9_1	100	6	139,9
G-CSF	V220-12_1	100	2	36,4
G-CSF	V220-16_1	100	11	194,3
G-CSF	V220-16_2	100	1	11,4
G-CSF	V220-17_1	100	8	140,5
G-CSF	V220-18_1	100	2	23,4
G-CSF	V221-2_1	100	5	59,4
G-CSF	V221-6_1	100	3	41,4
G-CSF	V221-7_1	100	3	39,5
G-CSF	V221-7_2	100	6	112,1
G-CSF	V221-11_1	100	0	2,8
G-CSF	V221-13_1	100	15	205,2
G-CSF	V221-15_1	100	1	10,1
G-CSF	V221-20_1	100	2	24
G-CSF	V222-19_1	100	12	155
G-CSF	V222-19_2	100	0	2,5
G-CSF	V222-20_1	100	10	220,8
G-CSF	V222-20_2	100	5	67,6
G-CSF	V222-21_1	100	10	142,5
G-CSF	V222-22_1	100	4	85,3
G-CSF	V222-22_2	100	6	76,8
G-CSF	V222-23_1	100	11	136,3
G-CSF	V222-24_1	100	8	104,1
G-CSF	V301-18_1	100	0	6,7
G-CSF	V301-18_2	100	3	34,2
G-CSF	V301-18_3	100	1	11
G-CSF	V301-19_1	100	2	28,3
G-CSF	V301-19_2	100	1	33
G-CSF	V301-20_1	100	2	30,7
G-CSF	V301-20_2	100	1	28,2
G-CSF	V302-15_1	100	1	10,6
G-CSF	V302-15_2	100	2	23,7
G-CSF	V302-15_3	100	3	35,2
G-CSF	V302-16_1	100	1	10,4
G-CSF	V302-16_4	100	5	64,3

G-CSF	V302-17_1	100	13	201,5
G-CSF	V302-17_2	100	1	21,9
G-CSF	V302-17_3	100	0	4,3
G-CSF	V303-15_2	100	2	25,6
G-CSF	V303-15_3	100	6	87,9
G-CSF	V303-16_1	100	12	150,6
G-CSF	V303-16_2	100	4	48,8
G-CSF	V303-16_3	100	2	27,8
G-CSF	V303-17_1	100	12	162,4

basilar dendrite Sholl 110µm

Group	number	Radius(µm)	Intersections	Length(µm)
Control	V215-10_2	110	10	129
Control	V215-12_1	110	3	46,2
Control	V215-12_2	110	4	72,1
Control	V215-13_1	110	2	50,4
Control	V215-15_2	110	1	11,5
Control	V215-16_1	110	3	43,5
Control	V215-18_1	110	0	8,4
Control	V216-11_1	110	2	20,3
Control	V216-14_1	110	1	11,4
Control	V216-16_2	110	3	61,9
Control	V216-17_1	110	1	14,7
Control	V216-18_2	110	3	41,4
Control	V219-6_1	110	14	273,7
Control	V219-11_1	110	6	77,3
Control	V219-12_1	110	7	96,2
Control	V219-13_1	110	3	20
Control	V219-14_2	110	5	47,8
Control	V219-16_1	110	1	20,3
Control	V297-19_1	110	4	80,4
Control	V297-20_2	110	0	2,3
Control	V297-20_3	110	6	87,2
Control	V297-22_1	110	11	128,8
Control	V298-15_1	110	9	115
Control	V298-16_2	110	1	24,5
Control	V298-17_1	110	0	14,1
Control	V298-17_2	110	7	85,5
Control	V298-17_3	110	9	121,8
Control	V308-17_1	110	1	33,9
Control	V308-17_2	110	9	98,2
Control	V308-17_3	110	6	76,6
Control	V308-17_4	110	3	62,4
Control	V308-18_2	110	1	17,4
Control	V308-18_3	110	8	122
CIMT	V211-10_1	110	14	168,6
CIMT	V211-10_2	110	8	131,3
CIMT	V211-11_1	110	3	26,8
CIMT	V211-11_2	110	0	12,1
CIMT	V211-12_1	110	1	12,4
CIMT	V211-13_1	110	2	34,4
CIMT	V211-13_2	110	4	60,1
CIMT	V211-15_2	110	1	11
CIMT	V211-16_1	110	2	35,2

CIMT	V212-14_1	110	8	85,9
CIMT	V212-14_2	110	10	121,9
CIMT	V212-15_1	110	9	128,4
CIMT	V212-18_1	110	3	36,8
CIMT	V212-21_1	110	4	50
CIMT	V212-21_2	110	15	232,9
CIMT	V212-22_1	110	4	56,5
CIMT	V212-23_1	110	2	23,4
CIMT	V212-23_2	110	25	361,1
CIMT	V212-24_1	110	3	45,2
CIMT	V218-9_1	110	0	6,5
CIMT	V218-10_1	110	6	73,7
CIMT	V218-11_1	110	11	150,3
CIMT	V218-12_1	110	11	199,2
CIMT	V218-12_2	110	2	25
CIMT	V218-15_1	110	0	4,3
CIMT	V218-17_1	110	3	55,1
CIMT	V299-14_1	110	1	23
CIMT	V299-16_2	110	6	112
CIMT	V299-17_1	110	6	73
CIMT	V299-17_2	110	2	21,3
CIMT	V300-16_1	110	9	115,1
CIMT	V300-18_1	110	1	17,8
CIMT	V300-18_2	110	6	72,1
CIMT	V300-20_1	110	3	49,8
CIMT	V300-22_1	110	7	76,5
CIMT	V300-23_1	110	1	12
CIMT	V300-23_2	110	0	5
CIMT	V309-15_1	110	1	10,7
CIMT	V309-15_2	110	3	36,6
CIMT	V309-16_3	110	5	79,2
CIMT	V309-17_1	110	3	26,6
CIMT	V309-17_2	110	10	118,7
CIMT+G-CSF	V213-13_1	110	3	31
CIMT+G-CSF	V213-17_1	110	2	51,7
CIMT+G-CSF	V213-17_2	110	5	63,1
CIMT+G-CSF	V213-18_1	110	4	78,7
CIMT+G-CSF	V213-18_2	110	4	48,1
CIMT+G-CSF	V213-19_1	110	5	68,3
CIMT+G-CSF	V213-19_2	110	1	11
CIMT+G-CSF	V213-22_1	110	5	73,6
CIMT+G-CSF	V213-23_1	110	1	12,1
CIMT+G-CSF	V214-14_1	110	7	120,5
CIMT+G-CSF	V214-17_1	110	6	84,6
CIMT+G-CSF	V214-18_1	110	12	154,1
CIMT+G-CSF	V214-19_1	110	1	10,3
CIMT+G-CSF	V214-19_2	110	5	71,6
CIMT+G-CSF	V214-20_1	110	8	106,8
CIMT+G-CSF	V214-21_1	110	4	53,1
CIMT+G-CSF	V217-1_1	110	13	175,3
CIMT+G-CSF	V217-2_1	110	4	66,4
CIMT+G-CSF	V217-4_1	110	20	275,6
CIMT+G-CSF	V217-6_1	110	0	3,2
CIMT+G-CSF	V217-6_3	110	2	32,4
CIMT+G-CSF	V217-8_4	110	4	67,6
CIMT+G-CSF	V217-10_1	110	1	14,3

CIMT+G-CSF	V217-11_1	110	11	170,8
CIMT+G-CSF	V304-17_1	110	0	1,1
CIMT+G-CSF	V304-17_2	110	2	21,4
CIMT+G-CSF	V304-17_3	110	3	35,4
CIMT+G-CSF	V304-17_4	110	6	60,8
CIMT+G-CSF	V304-19_1	110	1	11,6
CIMT+G-CSF	V304-19_2	110	2	33,9
CIMT+G-CSF	V305-18_1	110	3	34,9
CIMT+G-CSF	V305-18_2	110	0	11,8
CIMT+G-CSF	V305-18_4	110	1	14,1
CIMT/G-CSF	V306-16_1	110	1	11
CIMT/G-CSF	V306-16_2	110	0	2,1
CIMT/G-CSF	V306-17_1	110	5	56,7
CIMT/G-CSF	V306-17_2	110	3	58,1
CIMT/G-CSF	V306-17_3	110	1	20,7
CIMT/G-CSF	V306-17_4	110	1	12,5
CIMT/G-CSF	V311-16_1	110	13	161,2
CIMT/G-CSF	V311-17_1	110	4	56,6
CIMT/G-CSF	V311-17_2	110	4	47,1
CIMT/G-CSF	V311-19_1	110	4	73,2
CIMT/G-CSF	V311-20_1	110	8	102,9
CIMT/G-CSF	V311-20_2	110	4	43,9
CIMT/G-CSF	V311-20_3	110	3	46,8
CIMT/G-CSF	V313-14_1	110	5	65,2
CIMT/G-CSF	V313-17_1	110	6	80,3
CIMT/G-CSF	V313-17_2	110	1	10,9
CIMT/G-CSF	V314-16_2	110	3	53,2
CIMT/G-CSF	V314-17_1	110	1	20
CIMT/G-CSF	V314-17_3	110	0	7,3
CIMT/G-CSF	V314-18_1	110	1	19,3
CIMT/G-CSF	V314-18_2	110	4	91,2
CIMT/G-CSF	V315-11_1	110	3	36,6
CIMT/G-CSF	V315-13_1	110	1	11,6
CIMT/G-CSF	V315-13_2	110	9	128,2
CIMT/G-CSF	V315-14_1	110	4	59,8
CIMT/G-CSF	V315-14_2	110	2	28,7
CIMT/G-CSF	V315-14_3	110	13	176
G-CSF	V220-9_1	110	6	112,8
G-CSF	V220-12_1	110	1	12,3
G-CSF	V220-16_1	110	11	134,7
G-CSF	V220-16_2	110	2	32,3
G-CSF	V220-17_1	110	5	87,4
G-CSF	V220-18_1	110	2	38,9
G-CSF	V221-2_1	110	5	57,7
G-CSF	V221-6_1	110	2	30,3
G-CSF	V221-7_1	110	2	29,7
G-CSF	V221-7_2	110	4	59,3
G-CSF	V221-13_1	110	12	169,8
G-CSF	V221-15_1	110	1	10
G-CSF	V221-20_1	110	2	21
G-CSF	V222-19_1	110	10	123,4
G-CSF	V222-20_1	110	7	134,4
G-CSF	V222-20_2	110	5	59,7
G-CSF	V222-21_1	110	7	118
G-CSF	V222-22_1	110	3	44,4
G-CSF	V222-22_2	110	6	81,4

G-CSF	V222-23_1	110	11	127,3
G-CSF	V222-24_1	110	7	101,9
G-CSF	V301-18_2	110	3	36,4
G-CSF	V301-18_3	110	0	8,4
G-CSF	V301-19_1	110	1	23,5
G-CSF	V301-19_2	110	0	14,1
G-CSF	V301-20_1	110	0	11,2
G-CSF	V301-20_2	110	1	13
G-CSF	V302-15_1	110	0	11,2
G-CSF	V302-15_2	110	1	22,9
G-CSF	V302-15_3	110	3	42,4
G-CSF	V302-16_1	110	1	10,7
G-CSF	V302-16_4	110	1	32,8
G-CSF	V302-17_1	110	7	97,8
G-CSF	V302-17_2	110	0	3,8
G-CSF	V303-15_2	110	1	18,8
G-CSF	V303-15_3	110	3	75,5
G-CSF	V303-16_1	110	11	133
G-CSF	V303-16_2	110	4	43,7
G-CSF	V303-16_3	110	1	19,5
G-CSF	V303-17_1	110	10	112,5

basilar dendrite Sholl 120µm

Group	number	Radius(µm)	Intersections	Length(µm)
Control	V215-10_2	120	5	110,9
Control	V215-12_1	120	4	35,5
Control	V215-12_2	120	2	34,6
Control	V215-13_1	120	0	12
Control	V215-15_2	120	1	10,2
Control	V215-16_1	120	3	33,8
Control	V216-11_1	120	0	16,8
Control	V216-14_1	120	0	1,2
Control	V216-16_2	120	3	33,5
Control	V216-17_1	120	1	11,5
Control	V216-18_2	120	2	26,3
Control	V219-6_1	120	12	179
Control	V219-11_1	120	4	74,6
Control	V219-12_1	120	5	88,9
Control	V219-13_1	120	0	21,9
Control	V219-14_2	120	3	71,8
Control	V219-16_1	120	1	12
Control	V297-19_1	120	2	30,6
Control	V297-20_3	120	4	57,6
Control	V297-22_1	120	10	129,2
Control	V298-15_1	120	8	93,9
Control	V298-16_2	120	1	10,3
Control	V298-17_2	120	6	83,8
Control	V298-17_3	120	8	108,4
Control	V308-17_1	120	1	13,1
Control	V308-17_2	120	6	91,6
Control	V308-17_3	120	4	66,1
Control	V308-17_4	120	0	19,3
Control	V308-18_2	120	1	19,5
Control	V308-18_3	120	9	120,1

CIMT	V211-10_1	120	14	173,9
CIMT	V211-10_2	120	6	80,9
CIMT	V211-11_1	120	1	31,3
CIMT	V211-12_1	120	0	7,4
CIMT	V211-13_1	120	2	22,1
CIMT	V211-13_2	120	3	45
CIMT	V211-15_2	120	0	2,8
CIMT	V211-16_1	120	1	10,9
CIMT	V212-14_1	120	7	101,5
CIMT	V212-14_2	120	7	110
CIMT	V212-15_1	120	7	102,4
CIMT	V212-18_1	120	2	31,5
CIMT	V212-21_1	120	5	81,1
CIMT	V212-21_2	120	13	166,6
CIMT	V212-22_1	120	2	37
CIMT	V212-23_1	120	2	31,7
CIMT	V212-23_2	120	22	366,7
CIMT	V212-24_1	120	1	35,5
CIMT	V218-10_1	120	2	52,4
CIMT	V218-11_1	120	9	135,3
CIMT	V218-12_1	120	3	88,5
CIMT	V218-12_2	120	2	31,3
CIMT	V218-17_1	120	1	41,9
CIMT	V299-14_1	120	1	11,5
CIMT	V299-16_2	120	2	50,1
CIMT	V299-17_1	120	5	61,4
CIMT	V299-17_2	120	1	18,1
CIMT	V300-16_1	120	5	94,8
CIMT	V300-18_1	120	0	8,2
CIMT	V300-18_2	120	6	78,8
CIMT	V300-20_1	120	3	35,5
CIMT	V300-22_1	120	6	96,2
CIMT	V300-23_1	120	0	3,9
CIMT	V309-15_1	120	1	11,1
CIMT	V309-15_2	120	2	33,1
CIMT	V309-16_3	120	2	46,6
CIMT	V309-17_1	120	0	7,6
CIMT	V309-17_2	120	9	108,7
CIMT+G-CSF	V213-13_1	120	0	63,4
CIMT+G-CSF	V213-17_1	120	1	22
CIMT+G-CSF	V213-17_2	120	3	42,9
CIMT+G-CSF	V213-18_1	120	2	39,5
CIMT+G-CSF	V213-18_2	120	4	42,9
CIMT+G-CSF	V213-19_1	120	3	60,2
CIMT+G-CSF	V213-19_2	120	0	10,3
CIMT+G-CSF	V213-22_1	120	5	55,1
CIMT+G-CSF	V213-23_1	120	1	11,3
CIMT+G-CSF	V214-14_1	120	1	58,6
CIMT+G-CSF	V214-17_1	120	4	66,2
CIMT+G-CSF	V214-18_1	120	11	143,5
CIMT+G-CSF	V214-19_1	120	1	11,6
CIMT+G-CSF	V214-19_2	120	5	58,7
CIMT+G-CSF	V214-20_1	120	7	82,7
CIMT+G-CSF	V214-21_1	120	3	44,8
CIMT+G-CSF	V217-1_1	120	11	154,1
CIMT+G-CSF	V217-2_1	120	2	35,6

CIMT+G-CSF	V217-4_1	120	17	234,6
CIMT+G-CSF	V217-6_3	120	2	26,1
CIMT+G-CSF	V217-8_4	120	3	49,8
CIMT+G-CSF	V217-10_1	120	1	11,7
CIMT+G-CSF	V217-11_1	120	11	174,6
CIMT+G-CSF	V304-17_2	120	2	22,2
CIMT+G-CSF	V304-17_3	120	1	29,4
CIMT+G-CSF	V304-17_4	120	5	64,8
CIMT+G-CSF	V304-19_1	120	1	13,7
CIMT+G-CSF	V304-19_2	120	0	14,1
CIMT+G-CSF	V305-18_1	120	2	30,8
CIMT+G-CSF	V305-18_4	120	1	13
CIMT/G-CSF	V306-16_1	120	0	7,3
CIMT/G-CSF	V306-17_1	120	3	44,5
CIMT/G-CSF	V306-17_2	120	3	50,3
CIMT/G-CSF	V306-17_3	120	1	13,3
CIMT/G-CSF	V306-17_4	120	0	6,1
CIMT/G-CSF	V311-16_1	120	12	144,1
CIMT/G-CSF	V311-17_1	120	2	40,8
CIMT/G-CSF	V311-17_2	120	3	31,6
CIMT/G-CSF	V311-19_1	120	7	73,6
CIMT/G-CSF	V311-20_1	120	8	98,9
CIMT/G-CSF	V311-20_2	120	2	56,4
CIMT/G-CSF	V311-20_3	120	3	32,8
CIMT/G-CSF	V313-14_1	120	5	63,8
CIMT/G-CSF	V313-17_1	120	6	79
CIMT/G-CSF	V313-17_2	120	0	2,6
CIMT/G-CSF	V314-16_2	120	3	41,3
CIMT/G-CSF	V314-17_1	120	1	10,1
CIMT/G-CSF	V314-18_1	120	1	10,4
CIMT/G-CSF	V314-18_2	120	3	43,9
CIMT/G-CSF	V315-11_1	120	3	43,7
CIMT/G-CSF	V315-13_1	120	1	13,8
CIMT/G-CSF	V315-13_2	120	6	85
CIMT/G-CSF	V315-14_1	120	3	61,6
CIMT/G-CSF	V315-14_2	120	2	25,5
CIMT/G-CSF	V315-14_3	120	10	154,3
G-CSF	V220-9_1	120	4	79,5
G-CSF	V220-12_1	120	1	10,3
G-CSF	V220-16_1	120	4	71,6
G-CSF	V220-16_2	120	1	33,8
G-CSF	V220-17_1	120	6	68,1
G-CSF	V220-18_1	120	1	22
G-CSF	V221-2_1	120	3	39,6
G-CSF	V221-6_1	120	1	12,9
G-CSF	V221-7_1	120	2	20,3
G-CSF	V221-7_2	120	4	46
G-CSF	V221-13_1	120	9	128,4
G-CSF	V221-15_1	120	1	10,2
G-CSF	V221-20_1	120	2	20,7
G-CSF	V222-19_1	120	10	126,1
G-CSF	V222-20_1	120	6	95,6
G-CSF	V222-20_2	120	5	60,6
G-CSF	V222-21_1	120	7	91,2
G-CSF	V222-22_1	120	2	32
G-CSF	V222-22_2	120	5	62,4

G-CSF	V222-23_1	120	12	159,5
G-CSF	V222-24_1	120	6	76,3
G-CSF	V301-18_2	120	3	34,8
G-CSF	V301-19_1	120	0	9,1
G-CSF	V301-20_2	120	1	20,5
G-CSF	V302-15_2	120	1	10,5
G-CSF	V302-15_3	120	1	31,8
G-CSF	V302-16_1	120	1	10,1
G-CSF	V302-16_4	120	1	10,7
G-CSF	V302-17_1	120	5	72,7
G-CSF	V303-15_2	120	1	12,2
G-CSF	V303-15_3	120	3	34,4
G-CSF	V303-16_1	120	8	117
G-CSF	V303-16_2	120	4	47,2
G-CSF	V303-16_3	120	1	14,2
G-CSF	V303-17_1	120	10	116

basilar dendrite Sholl 130µm

Group	number	Radius(µm)	Intersections	Length(µm)
Control	V215-10_2	130	5	62,8
Control	V215-12_1	130	2	37,4
Control	V215-12_2	130	1	30,2
Control	V215-15_2	130	0	25,8
Control	V215-16_1	130	1	18,9
Control	V216-16_2	130	3	35,7
Control	V216-17_1	130	1	11,2
Control	V216-18_2	130	2	21,8
Control	V219-6_1	130	9	162,8
Control	V219-11_1	130	4	48,5
Control	V219-12_1	130	4	44,6
Control	V219-14_2	130	2	31,4
Control	V219-16_1	130	1	12,5
Control	V297-19_1	130	2	32,2
Control	V297-20_3	130	4	45
Control	V297-22_1	130	10	116,7
Control	V298-15_1	130	9	97
Control	V298-16_2	130	1	12
Control	V298-17_2	130	5	68,3
Control	V298-17_3	130	7	84,4
Control	V308-17_1	130	0	2,5
Control	V308-17_2	130	5	75
Control	V308-17_3	130	2	38,1
Control	V308-18_2	130	0	3
Control	V308-18_3	130	6	172,9
CIMT	V211-10_1	130	12	148
CIMT	V211-10_2	130	6	67,9
CIMT	V211-11_1	130	1	10,4
CIMT	V211-13_1	130	3	25,2
CIMT	V211-13_2	130	2	36,8
CIMT	V211-16_1	130	1	12,6
CIMT	V212-14_1	130	7	99,8
CIMT	V212-14_2	130	5	74
CIMT	V212-15_1	130	7	88,2
CIMT	V212-18_1	130	2	24

CIMT	V212-21_1	130	7	64,9
CIMT	V212-21_2	130	13	148,9
CIMT	V212-22_1	130	0	5,4
CIMT	V212-23_1	130	2	33,1
CIMT	V212-23_2	130	19	296,8
CIMT	V212-24_1	130	1	12,2
CIMT	V218-10_1	130	2	26,9
CIMT	V218-11_1	130	9	120,4
CIMT	V218-12_1	130	2	34,4
CIMT	V218-12_2	130	2	21,9
CIMT	V218-17_1	130	0	0,8
CIMT	V299-14_1	130	0	0,7
CIMT	V299-16_2	130	2	23,1
CIMT	V299-17_1	130	2	63,9
CIMT	V299-17_2	130	1	10,5
CIMT	V300-16_1	130	5	80,4
CIMT	V300-18_2	130	4	67,6
CIMT	V300-20_1	130	3	36,2
CIMT	V300-22_1	130	3	51,1
CIMT	V309-15_1	130	1	10,9
CIMT	V309-15_2	130	0	10,2
CIMT	V309-16_3	130	0	5,1
CIMT	V309-17_2	130	10	111,9
CIMT+G-CSF	V213-17_1	130	1	11,9
CIMT+G-CSF	V213-17_2	130	2	28,1
CIMT+G-CSF	V213-18_1	130	2	23,7
CIMT+G-CSF	V213-18_2	130	4	50,2
CIMT+G-CSF	V213-19_1	130	2	34,2
CIMT+G-CSF	V213-22_1	130	3	53,5
CIMT+G-CSF	V213-23_1	130	1	11,7
CIMT+G-CSF	V214-14_1	130	0	5,5
CIMT+G-CSF	V214-17_1	130	2	54,6
CIMT+G-CSF	V214-18_1	130	11	120,9
CIMT+G-CSF	V214-19_1	130	1	11,1
CIMT+G-CSF	V214-19_2	130	4	58,2
CIMT+G-CSF	V214-20_1	130	6	67,9
CIMT+G-CSF	V214-21_1	130	3	35,4
CIMT+G-CSF	V217-1_1	130	8	131
CIMT+G-CSF	V217-2_1	130	1	20,6
CIMT+G-CSF	V217-4_1	130	15	174,6
CIMT+G-CSF	V217-6_3	130	1	18,1
CIMT+G-CSF	V217-8_4	130	3	35,6
CIMT+G-CSF	V217-10_1	130	1	12,1
CIMT+G-CSF	V217-11_1	130	9	126,1
CIMT+G-CSF	V304-17_2	130	0	9,8
CIMT+G-CSF	V304-17_3	130	1	11
CIMT+G-CSF	V304-17_4	130	1	41,7
CIMT+G-CSF	V304-19_1	130	1	14,1
CIMT+G-CSF	V305-18_1	130	1	15,5
CIMT+G-CSF	V305-18_4	130	1	12,7
CIMT/G-CSF	V306-17_1	130	4	58
CIMT/G-CSF	V306-17_2	130	2	52,1
CIMT/G-CSF	V306-17_3	130	1	10,7
CIMT/G-CSF	V311-16_1	130	7	110,6
CIMT/G-CSF	V311-17_1	130	1	22,4
CIMT/G-CSF	V311-17_2	130	2	28,1

CIMT/G-CSF	V311-19_1	130	2	85,3
CIMT/G-CSF	V311-20_1	130	9	103,1
CIMT/G-CSF	V311-20_2	130	2	24,7
CIMT/G-CSF	V311-20_3	130	2	23,7
CIMT/G-CSF	V313-14_1	130	5	71,6
CIMT/G-CSF	V313-17_1	130	5	61,2
CIMT/G-CSF	V314-16_2	130	2	30,4
CIMT/G-CSF	V314-17_1	130	1	10,1
CIMT/G-CSF	V314-18_1	130	0	0,8
CIMT/G-CSF	V314-18_2	130	1	23,1
CIMT/G-CSF	V315-11_1	130	0	12,2
CIMT/G-CSF	V315-13_1	130	2	16,3
CIMT/G-CSF	V315-13_2	130	2	45
CIMT/G-CSF	V315-14_1	130	2	37,4
CIMT/G-CSF	V315-14_2	130	1	20,2
CIMT/G-CSF	V315-14_3	130	6	118,3
G-CSF	V220-9_1	130	3	43,7
G-CSF	V220-12_1	130	1	11,8
G-CSF	V220-16_1	130	3	67,4
G-CSF	V220-16_2	130	1	12,9
G-CSF	V220-17_1	130	2	64,2
G-CSF	V220-18_1	130	1	12
G-CSF	V221-2_1	130	0	15,2
G-CSF	V221-6_1	130	0	0,4
G-CSF	V221-7_1	130	2	21,8
G-CSF	V221-7_2	130	3	32,8
G-CSF	V221-13_1	130	7	81,3
G-CSF	V221-15_1	130	1	10,3
G-CSF	V221-20_1	130	2	21,1
G-CSF	V222-19_1	130	9	176,7
G-CSF	V222-20_1	130	4	91,7
G-CSF	V222-20_2	130	3	45,3
G-CSF	V222-21_1	130	7	98,7
G-CSF	V222-22_1	130	2	41,9
G-CSF	V222-22_2	130	3	41,8
G-CSF	V222-23_1	130	11	163,3
G-CSF	V222-24_1	130	6	66,9
G-CSF	V301-18_2	130	2	31,1
G-CSF	V301-20_2	130	1	12
G-CSF	V302-15_2	130	1	11
G-CSF	V302-15_3	130	0	6,9
G-CSF	V302-16_1	130	0	7
G-CSF	V302-16_4	130	1	10,5
G-CSF	V302-17_1	130	4	58,3
G-CSF	V303-15_2	130	0	7,5
G-CSF	V303-15_3	130	3	33,8
G-CSF	V303-16_1	130	5	89,2
G-CSF	V303-16_2	130	3	36,9
G-CSF	V303-16_3	130	0	2
G-CSF	V303-17_1	130	10	134,9

basilar dendrite Sholl 140µm

Group	number	Radius(µm)	Intersections	Length(µm)
Control	V215-10_2	140	5	67,6
Control	V215-12_1	140	1	21
Control	V215-12_2	140	0	21,3
Control	V215-16_1	140	0	9,7
Control	V216-16_2	140	2	24,5
Control	V216-17_1	140	0	8,2
Control	V216-18_2	140	2	24
Control	V219-6_1	140	9	124,5
Control	V219-11_1	140	3	66,2
Control	V219-12_1	140	2	37,5
Control	V219-14_2	140	1	17,4
Control	V219-16_1	140	1	10,7
Control	V297-19_1	140	1	17,3
Control	V297-20_3	140	3	44,6
Control	V297-22_1	140	9	142
Control	V298-15_1	140	8	110,8
Control	V298-16_2	140	0	11,9
Control	V298-17_2	140	4	67,8
Control	V298-17_3	140	5	68,3
Control	V308-17_2	140	2	44,7
Control	V308-17_3	140	1	12,3
Control	V308-18_3	140	6	76,1
CIMT	V211-10_1	140	13	176,3
CIMT	V211-10_2	140	5	75,3
CIMT	V211-11_1	140	0	9,1
CIMT	V211-13_1	140	2	29,1
CIMT	V211-13_2	140	0	35
CIMT	V211-16_1	140	1	10,8
CIMT	V212-14_1	140	6	78,6
CIMT	V212-14_2	140	10	91,5
CIMT	V212-15_1	140	6	82,4
CIMT	V212-18_1	140	2	26,7
CIMT	V212-21_1	140	3	98,9
CIMT	V212-21_2	140	9	178,1
CIMT	V212-23_1	140	1	12,2
CIMT	V212-23_2	140	19	248,1
CIMT	V212-24_1	140	0	9,9
CIMT	V218-10_1	140	1	18,3
CIMT	V218-11_1	140	8	98,1
CIMT	V218-12_1	140	2	33,3
CIMT	V218-12_2	140	2	22,9
CIMT	V299-16_2	140	2	21,5
CIMT	V299-17_1	140	1	20
CIMT	V299-17_2	140	1	10,3
CIMT	V300-16_1	140	4	49,3
CIMT	V300-18_2	140	3	41,5
CIMT	V300-20_1	140	2	29,5
CIMT	V300-22_1	140	2	24,6
CIMT	V309-15_1	140	1	11
CIMT	V309-17_2	140	8	116,3
CIMT+G-CSF	V213-17_1	140	1	10,2
CIMT+G-CSF	V213-17_2	140	2	21,2
CIMT+G-CSF	V213-18_1	140	2	23,8

CIMT+G-CSF	V213-18_2	140	1	35,9
CIMT+G-CSF	V213-19_1	140	1	16,2
CIMT+G-CSF	V213-22_1	140	3	43,6
CIMT+G-CSF	V213-23_1	140	1	10,4
CIMT+G-CSF	V214-17_1	140	2	23,1
CIMT+G-CSF	V214-18_1	140	10	119
CIMT+G-CSF	V214-19_1	140	0	0
CIMT+G-CSF	V214-19_2	140	3	40,1
CIMT+G-CSF	V214-20_1	140	5	56,7
CIMT+G-CSF	V214-21_1	140	3	33,5
CIMT+G-CSF	V217-1_1	140	6	81,1
CIMT+G-CSF	V217-2_1	140	0	7,7
CIMT+G-CSF	V217-4_1	140	14	164,6
CIMT+G-CSF	V217-6_3	140	1	13,5
CIMT+G-CSF	V217-8_4	140	2	43,4
CIMT+G-CSF	V217-10_1	140	0	9,1
CIMT+G-CSF	V217-11_1	140	8	159,8
CIMT+G-CSF	V304-17_3	140	1	12,4
CIMT+G-CSF	V304-17_4	140	0	6,6
CIMT+G-CSF	V304-19_1	140	1	11,3
CIMT+G-CSF	V305-18_1	140	1	11,1
CIMT+G-CSF	V305-18_4	140	0	8,4
CIMT/G-CSF	V306-17_1	140	3	54,4
CIMT/G-CSF	V306-17_2	140	1	31,5
CIMT/G-CSF	V306-17_3	140	1	10,4
CIMT/G-CSF	V311-16_1	140	6	89,7
CIMT/G-CSF	V311-17_1	140	1	12,8
CIMT/G-CSF	V311-17_2	140	1	15
CIMT/G-CSF	V311-19_1	140	1	29,3
CIMT/G-CSF	V311-20_1	140	7	123
CIMT/G-CSF	V311-20_2	140	1	23,4
CIMT/G-CSF	V311-20_3	140	2	22,7
CIMT/G-CSF	V313-14_1	140	4	67,6
CIMT/G-CSF	V313-17_1	140	3	52,3
CIMT/G-CSF	V314-16_2	140	1	18,7
CIMT/G-CSF	V314-17_1	140	1	10,1
CIMT/G-CSF	V314-18_2	140	0	1,7
CIMT/G-CSF	V315-13_1	140	2	26,6
CIMT/G-CSF	V315-13_2	140	2	22,1
CIMT/G-CSF	V315-14_1	140	0	16,6
CIMT/G-CSF	V315-14_2	140	1	11
CIMT/G-CSF	V315-14_3	140	6	71,8
G-CSF	V220-9_1	140	2	42,2
G-CSF	V220-12_1	140	1	16,4
G-CSF	V220-16_1	140	4	71,2
G-CSF	V220-16_2	140	1	11,5
G-CSF	V220-17_1	140	2	26
G-CSF	V220-18_1	140	0	2,6
G-CSF	V221-7_1	140	2	23,7
G-CSF	V221-7_2	140	2	32,3
G-CSF	V221-13_1	140	5	61,1
G-CSF	V221-15_1	140	1	10
G-CSF	V221-20_1	140	2	35,2
G-CSF	V222-19_1	140	7	97,1
G-CSF	V222-20_1	140	3	45,8
G-CSF	V222-20_2	140	0	11,3

G-CSF	V222-21_1	140	4	81,6
G-CSF	V222-22_1	140	0	10
G-CSF	V222-22_2	140	1	13,8
G-CSF	V222-23_1	140	7	141,7
G-CSF	V222-24_1	140	2	39,7
G-CSF	V301-18_2	140	2	28,6
G-CSF	V301-20_2	140	0	2,5
G-CSF	V302-15_2	140	1	10,7
G-CSF	V302-16_4	140	1	10,5
G-CSF	V302-17_1	140	3	36,9
G-CSF	V303-15_3	140	3	40,1
G-CSF	V303-16_1	140	4	54,8
G-CSF	V303-16_2	140	3	31,8
G-CSF	V303-17_1	140	5	82,8

basilar dendrite Sholl 150μm

Group	number	Radius(μm)	Intersections	Length(μm)
Control	V215-10_2	150	6	82,1
Control	V215-12_1	150	1	10,4
Control	V216-16_2	150	2	31,3
Control	V216-18_2	150	1	17,2
Control	V219-6_1	150	8	120,5
Control	V219-11_1	150	3	39,1
Control	V219-12_1	150	2	28,2
Control	V219-14_2	150	0	1,1
Control	V219-16_1	150	1	11,1
Control	V297-19_1	150	0	5,4
Control	V297-20_3	150	3	43,3
Control	V297-22_1	150	2	75,1
Control	V298-15_1	150	7	82,8
Control	V298-17_2	150	3	42,6
Control	V298-17_3	150	5	55,1
Control	V308-17_2	150	1	19,8
Control	V308-17_3	150	0	10,1
Control	V308-18_3	150	5	78,5
CIMT	V211-10_1	150	12	155,3
CIMT	V211-10_2	150	7	63,5
CIMT	V211-13_1	150	1	14,4
CIMT	V211-16_1	150	1	10,4
CIMT	V212-14_1	150	4	62,7
CIMT	V212-14_2	150	9	221,2
CIMT	V212-15_1	150	3	67,1
CIMT	V212-18_1	150	1	15,8
CIMT	V212-21_1	150	2	30
CIMT	V212-21_2	150	8	95,7
CIMT	V212-23_1	150	1	13,6
CIMT	V212-23_2	150	12	254
CIMT	V218-10_1	150	0	3,6
CIMT	V218-11_1	150	5	79,4
CIMT	V218-12_1	150	0	13,4
CIMT	V218-12_2	150	1	21,5
CIMT	V299-16_2	150	1	14
CIMT	V299-17_1	150	1	13
CIMT	V299-17_2	150	1	11,2

CIMT	V300-16_1	150	5	60,6
CIMT	V300-18_2	150	2	32,6
CIMT	V300-20_1	150	1	12,9
CIMT	V300-22_1	150	2	24,6
CIMT	V309-15_1	150	0	11,9
CIMT	V309-17_2	150	7	93,9
CIMT+G-CSF	V213-17_1	150	0	7,3
CIMT+G-CSF	V213-17_2	150	2	22,2
CIMT+G-CSF	V213-18_1	150	1	44,3
CIMT+G-CSF	V213-18_2	150	1	10,5
CIMT+G-CSF	V213-19_1	150	1	16,3
CIMT+G-CSF	V213-22_1	150	2	29,5
CIMT+G-CSF	V213-23_1	150	1	10,3
CIMT+G-CSF	V214-17_1	150	2	23,8
CIMT+G-CSF	V214-18_1	150	10	116,3
CIMT+G-CSF	V214-19_2	150	2	29,6
CIMT+G-CSF	V214-20_1	150	2	38,7
CIMT+G-CSF	V214-21_1	150	2	27,5
CIMT+G-CSF	V217-1_1	150	6	129,1
CIMT+G-CSF	V217-4_1	150	12	157,9
CIMT+G-CSF	V217-6_3	150	1	14
CIMT+G-CSF	V217-8_4	150	2	23,2
CIMT+G-CSF	V217-11_1	150	7	93,7
CIMT+G-CSF	V304-17_3	150	1	10,1
CIMT+G-CSF	V304-19_1	150	0	1,5
CIMT+G-CSF	V305-18_1	150	1	11,6
CIMT/G-CSF	V306-17_1	150	2	27,2
CIMT/G-CSF	V306-17_2	150	1	16,7
CIMT/G-CSF	V306-17_3	150	1	13,9
CIMT/G-CSF	V311-16_1	150	6	68,1
CIMT/G-CSF	V311-17_1	150	1	10,3
CIMT/G-CSF	V311-17_2	150	1	10,3
CIMT/G-CSF	V311-19_1	150	0	2,6
CIMT/G-CSF	V311-20_1	150	7	79
CIMT/G-CSF	V311-20_2	150	0	6,7
CIMT/G-CSF	V311-20_3	150	1	22,6
CIMT/G-CSF	V313-14_1	150	4	54,4
CIMT/G-CSF	V313-17_1	150	2	30,5
CIMT/G-CSF	V314-16_2	150	0	2,3
CIMT/G-CSF	V314-17_1	150	1	10,2
CIMT/G-CSF	V315-13_1	150	0	12,1
CIMT/G-CSF	V315-13_2	150	1	16,6
CIMT/G-CSF	V315-14_2	150	1	14,6
CIMT/G-CSF	V315-14_3	150	4	57
G-CSF	V220-9_1	150	2	22,9
G-CSF	V220-12_1	150	0	0
G-CSF	V220-16_1	150	3	104,8
G-CSF	V220-16_2	150	1	10,1
G-CSF	V220-17_1	150	2	24,4
G-CSF	V221-7_1	150	2	30,2
G-CSF	V221-7_2	150	1	17,3
G-CSF	V221-13_1	150	5	54,9
G-CSF	V221-15_1	150	1	10,1
G-CSF	V221-20_1	150	2	20,8
G-CSF	V222-19_1	150	4	58,1
G-CSF	V222-20_1	150	2	25

G-CSF	V222-21_1	150	3	35,4
G-CSF	V222-22_2	150	1	29,3
G-CSF	V222-23_1	150	4	71,8
G-CSF	V222-24_1	150	1	14
G-CSF	V301-18_2	150	1	12,4
G-CSF	V302-15_2	150	1	13,7
G-CSF	V302-16_4	150	1	10,8
G-CSF	V302-17_1	150	2	29,4
G-CSF	V303-15_3	150	3	35,9
G-CSF	V303-16_1	150	4	47,8
G-CSF	V303-16_2	150	2	30,9
G-CSF	V303-17_1	150	4	54,3
